# Supplementary material for: Rh(III)-Catalyzed Annulation of Boc-Protected Benzamides with Diazo Compounds: Approach to Isocoumarins
Source: Molecules. 2019 Mar 7;24(5):937. doi: 10.3390/molecules24050937 (PMC6429621; doi:10.3390/molecules24050937)
Supplement: Supplementary file 1 [file molecules-24-00937-s001.pdf]

Supporting Information for

Rh(III)-Catalyzed Annulation of Boc-protected benzamides with Diazo Compounds: Approach to isocoumarins

Guangyu Dong [1, 2, 3], Chunpu Li [1] and Hong Liu [1, 2, 3*]

[1] State Key Laboratory of Drug Research, Shanghai Institute of Materia Medica, Chinese Academy of Sciences, 555 Zuchongzhi Road, Shanghai 201203, China;18115120903@163.com (G.D.); lcp1681993@163.com (C.L.)

[2] University of Chinese Academy of Sciences, No.19A Yuquan Road, Beijing 100049, China

[3] School of Life Science and Technology, ShanghaiTech University,100 haike Road,Shanghai 201210,China

* Correspondence: hliu@simm.ac.cn; Tel.: +86-21-50807042

Contents

1. X-ray single crystal structure of compound **3ga** 2
2. ^{1}H and ^{13}C NMR Spectra of benzamides 6
3. ^{1}H and ^{13}C NMR Spectra of Products 26
4. ^{1}H Spectra of Compete experiments 61
5. MS Spectra of benzamides 64
6. MS Spectra of Products 74

1. X-ray single crystal structure of compound 3ga.

These data can be obtained free of charge via https://www.ccdc.cam.ac.uk（CCDC: 1891024）

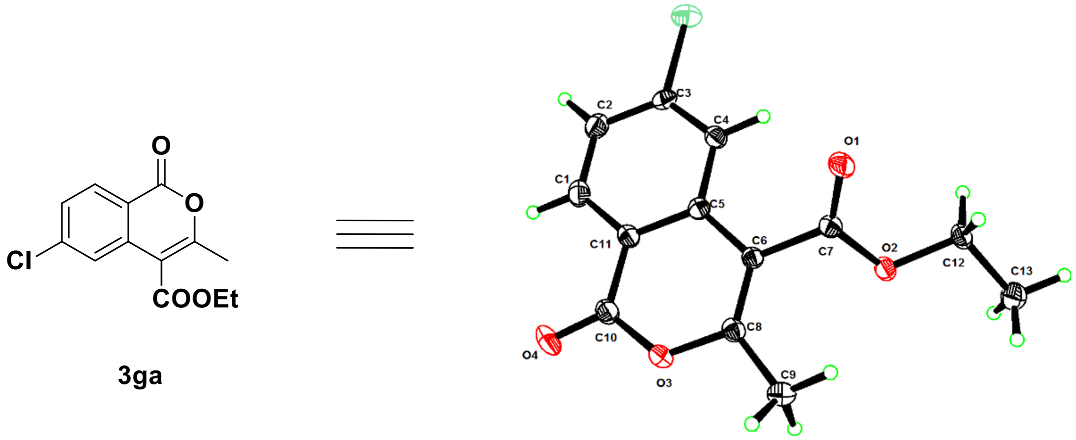

Scheme S1. X-ray crystallography data of 3ga

Table1. Crystal data and structure refinement for **3ga.**

Identification code	mo_20180573-b6-ff-61-1_0m
Empirical formula	$C_{13}H_{11}ClO_4$
Formula weight	266.67
Temperature/K	173
Crystal system	monoclinic
Space group	$P2_1/c$
a/Å	3.878(3)
b/Å	30.07(2)
c/Å	10.204(7)
α/°	90
β/°	95.477(15)
γ/°	90
Volume/Å^3	1184.2(14)
Z	4
ρ_{calc}g/cm^3	1.496
μ/mm^{-1}	0.326
F(000)	552.0
Crystal size/mm^3	0.15 × 0.08 × 0.03
Radiation	MoKα (λ = 0.71073)
2Θ range for data collection/°	4.232 to 52.798
Index ranges	$-4 \leq h \leq 4$, $-37 \leq k \leq 37$, $-12 \leq l \leq 7$
Reflections collected	6521
Independent reflections	2354 [R_{int} = 0.0623, R_{sigma} = 0.0768]
Data/restraints/parameters	2354/0/165

Goodness-of-fit on F^2	1.000
Final R indexes [I>=2σ (I)]	R_1 = 0.0600, wR_2 = 0.1591
Final R indexes [all data]	R_1 = 0.0902, wR_2 = 0.1747
Largest diff. peak/hole / e $Å^{-3}$	0.28/-0.30

Table2. Fractional Atomic Coordinates (×10^4) and Equivalent Isotropic Displacement Parameters ($Å^2$×10^3) for **3ga**. U_{eq} is defined as 1/3 of of the trace of the orthogonalised U_{IJ} tensor.

**Atom**	***x***	***y***	***z***	**U(eq)**
Cl1	361(2)	5944.3(3)	675.3(9)	45.6(3)
O2	6315(5)	5728.7(6)	6585(2)	31.6(6)
O3	8445(6)	7080.0(6)	5700(2)	34.4(6)
O1	8611(6)	5573.9(7)	4706(2)	37.8(6)
O4	6997(7)	7636.2(7)	4356(3)	52.0(8)
C11	5345(7)	6915.9(9)	3583(3)	28.3(7)
C5	5529(7)	6460.6(9)	3887(3)	25.7(7)
C6	7188(7)	6326.9(9)	5153(3)	24.8(7)
C8	8569(8)	6631.3(9)	6017(3)	28.5(7)
C7	7488(7)	5838.8(9)	5454(3)	27.5(7)
C4	3936(8)	6160.9(10)	2958(3)	28.9(7)
C3	2329(7)	6321.5(10)	1804(3)	28.9(7)
C12	6101(8)	5252.9(9)	6849(3)	31.5(7)
C10	6913(8)	7241.6(10)	4517(4)	33.9(8)
C9	10303(8)	6569.4(10)	7356(3)	35.4(8)
C2	2179(9)	6770.3(11)	1493(4)	36.8(8)
C1	3702(9)	7064.3(10)	2389(4)	36.0(8)
C13	5217(10)	5202.7(11)	8240(4)	41.9(9)

Table3. Anisotropic Displacement Parameters ($Å^2$×10^3) for **3ga**. The Anisotropic displacement factor exponent takes the form: $-2\pi^2[h^2a^{*2}U_{11}+2hka^*b^*U_{12}+\ldots]$.

**Atom**	**U_{11}**	**U_{22}**	**U_{33}**	**U_{23}**	**U_{13}**	**U_{12}**
Cl1	48.0(5)	48.9(6)	38.6(6)	-11.6(4)	-3.1(4)	-2.8(4)
O2	42.3(12)	22.4(11)	32.4(14)	-0.2(9)	14.6(10)	-0.4(8)
O3	43.6(13)	24.6(11)	34.9(15)	-4.6(9)	4.4(11)	-5.0(8)
O1	48.9(13)	27.3(11)	39.3(15)	-3.3(10)	15.6(12)	6.5(9)
O4	71.5(18)	22.3(12)	61(2)	0.1(11)	2.2(15)	-8.0(11)
C11	28.2(15)	24.4(14)	34(2)	-2.2(13)	10.7(14)	-0.8(11)
C5	25.1(15)	26.1(15)	27.4(19)	-0.9(12)	9.7(13)	1.0(10)
C6	26.6(14)	22.7(14)	26.0(18)	-1.5(12)	7.5(13)	0.8(10)
C8	29.7(16)	24.4(15)	33(2)	-2.0(13)	9.2(14)	1.9(11)
C7	27.1(15)	26.7(15)	28.6(19)	-1.4(13)	2.5(13)	-1.3(11)
C4	30.9(16)	24.6(15)	32(2)	-1.7(13)	7.4(14)	-0.6(11)
C3	27.6(15)	37.2(17)	22.6(18)	-4.2(13)	5.9(13)	-0.8(12)
C12	37.9(17)	21.5(15)	36(2)	2.0(13)	8.6(15)	-1.7(11)

C10	38.4(18)	27.4(16)	37(2)	-1.9(14)	10.1(16)	-3.8(12)
C9	37.2(18)	37.0(18)	32(2)	-7.1(15)	3.2(16)	0.5(13)
C2	40.8(19)	38.7(18)	31(2)	4.3(15)	3.8(16)	3.5(13)
C1	39.7(18)	30.8(17)	38(2)	5.9(15)	8.6(16)	0.9(12)
C13	50(2)	37.8(19)	39(2)	3.3(16)	11.3(18)	-5.8(15)

Table4. Bond Lengths for **3ga**.

**Atom**	**Atom**	**Length/Å**	**Atom**	**Atom**	**Length/Å**
Cl1	C3	1.740(3)	C5	C6	1.444(4)
O2	C7	1.322(4)	C5	C4	1.408(4)
O2	C12	1.459(4)	C6	C8	1.345(4)
O3	C8	1.387(4)	C6	C7	1.502(4)
O3	C10	1.381(4)	C8	C9	1.476(5)
O1	C7	1.212(4)	C4	C3	1.367(4)
O4	C10	1.199(4)	C3	C2	1.386(4)
C11	C5	1.404(4)	C12	C13	1.500(5)
C11	C10	1.458(4)	C2	C1	1.365(5)
C11	C1	1.393(5)			

Table5. Bond Angles for **3ga**.

Atom	Atom	Atom	Angle/°	Atom	Atom	Atom	Angle/°
C7	O2	C12	115.9(2)	O2	C7	C6	113.4(3)
C10	O3	C8	123.2(2)	O1	C7	O2	124.0(3)
C5	C11	C10	120.1(3)	O1	C7	C6	122.6(3)
C1	C11	C5	121.0(3)	C3	C4	C5	119.3(3)
C1	C11	C10	118.9(3)	C4	C3	Cl1	118.4(2)
C11	C5	C6	118.6(3)	C4	C3	C2	123.1(3)
C11	C5	C4	117.7(3)	C2	C3	Cl1	118.5(3)
C4	C5	C6	123.7(3)	O2	C12	C13	107.2(3)
C5	C6	C7	118.3(2)	O3	C10	C11	116.9(3)
C8	C6	C5	120.8(3)	O4	C10	O3	116.9(3)
C8	C6	C7	120.9(3)	O4	C10	C11	126.2(3)
O3	C8	C9	109.9(3)	C1	C2	C3	118.1(3)
C6	C8	O3	120.4(3)	C2	C1	C11	120.7(3)
C6	C8	C9	129.6(3)				

Table6. Hydrogen Atom Coordinates (Å×10^4) and Isotropic Displacement Parameters (Å^2×10^3) for **3ga**.

**Atom**	***x***	***y***	***z***	**U(eq)**
H4	3978	5850	3130	35
H12A	8345	5107	6738	38

H12B	4290	5114	6232	38
H9A	12405	6752	7463	53
H9B	10923	6256	7489	53
H9C	8732	6660	8005	53
H2	1049	6870	680	44
H1	3641	7373	2197	43
H13A	3109	5372	8358	63
H13B	7133	5316	8845	63
H13C	4832	4888	8426	63

Crystal structure determination of 3ga.

Crystal Data for $C_{13}H_{11}ClO_4$ (*M* =266.67 g/mol): monoclinic, space group $P2_1/c$ (no. 14), a = 3.878(3) Å, b = 30.07(2) Å, c = 10.204(7) Å, β = 95.477(15)°, V = 1184.2(14) Å^3, Z = 4, T = 173 K, μ(MoKα) = 0.326 mm^{-1}, *Dcalc* = 1.496 g/cm^3, 6521 reflections measured (4.232° ≤ 2Θ ≤ 52.798°), 2354 unique (R_{int} = 0.0623, R_{sigma} = 0.0768) which were used in all calculations. The final R_1 was 0.0600 (I > 2σ(I)) and wR_2 was 0.1747 (all data).

6.¹H and ¹³C NMR Spectra of benzamides

tert-Butyl (4-ethylbenzoyl)carbamate (1c).

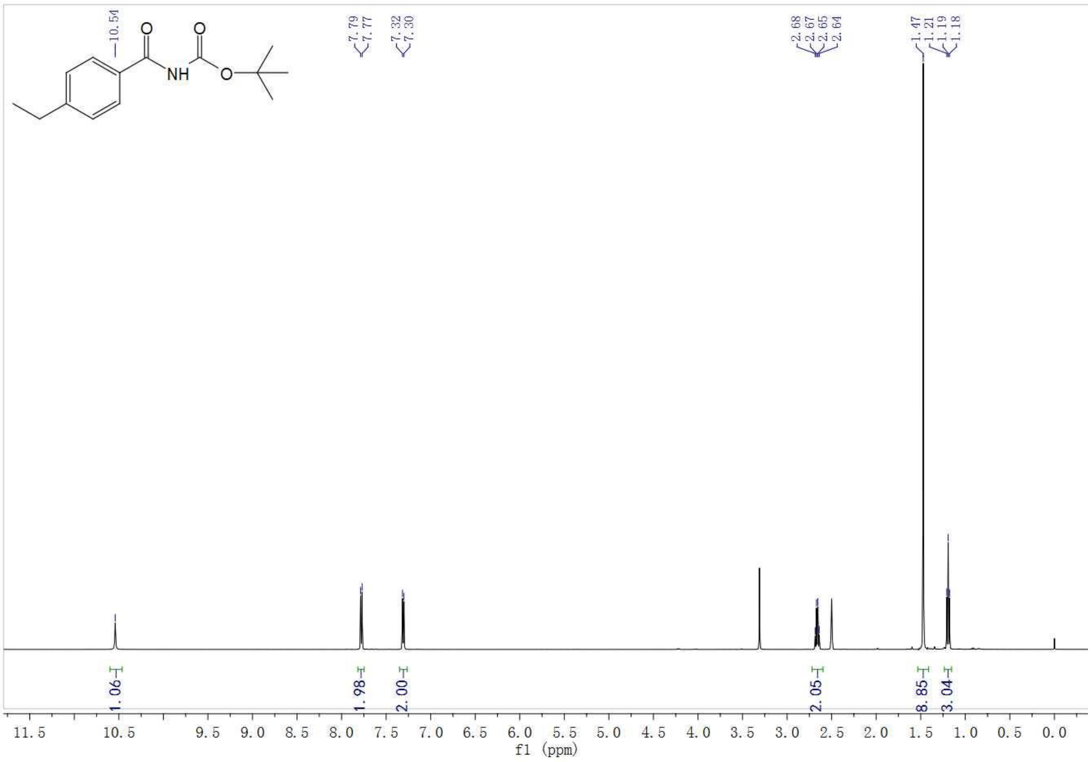

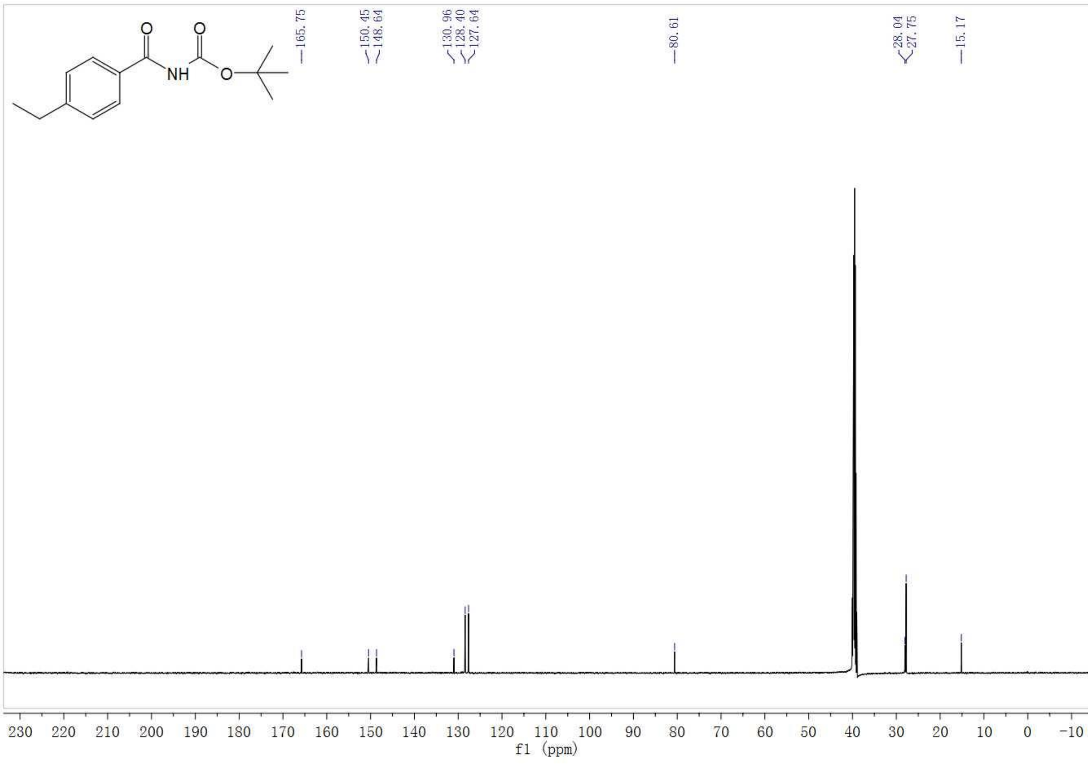

tert-Butyl (4-isopropylbenzoyl)carbamate (1d).

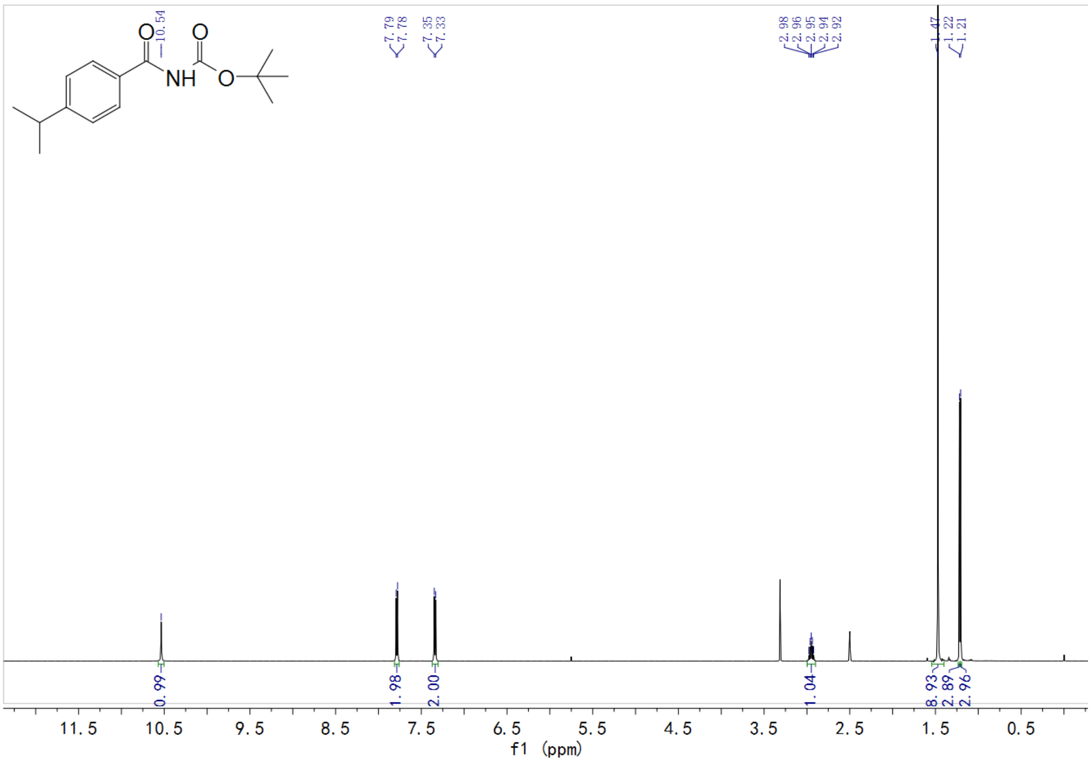

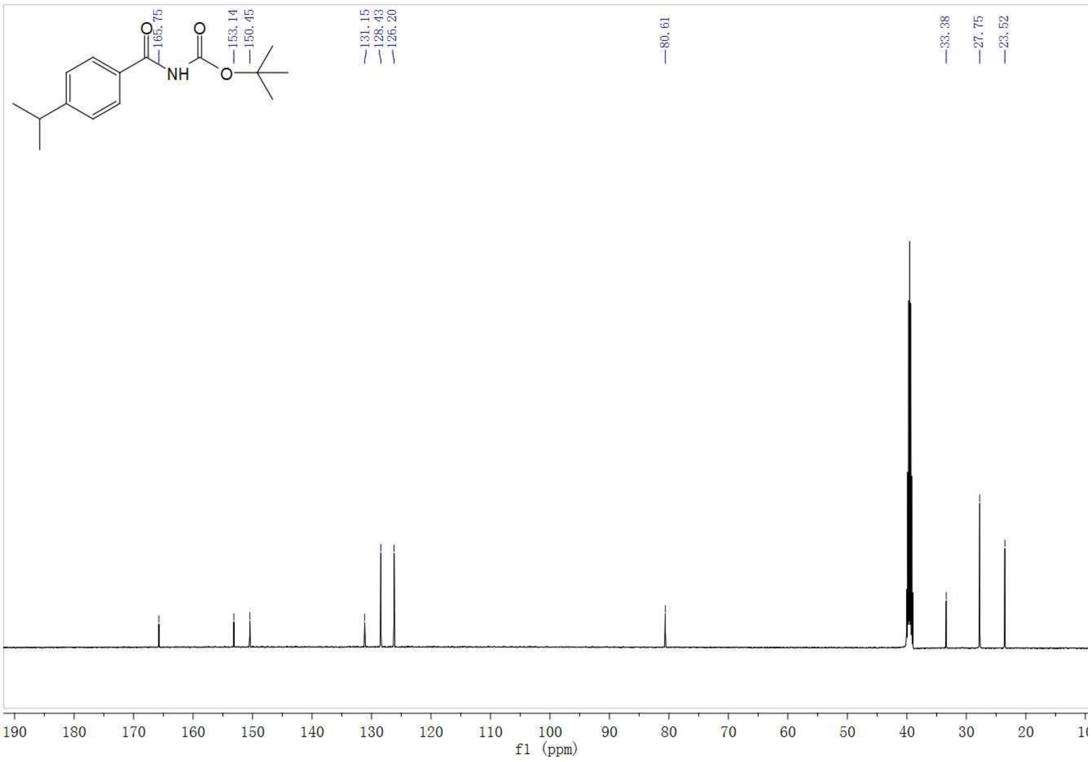

***tetert*-Butyl (4-(tert-butyl)benzoyl)carbamate (1e).**

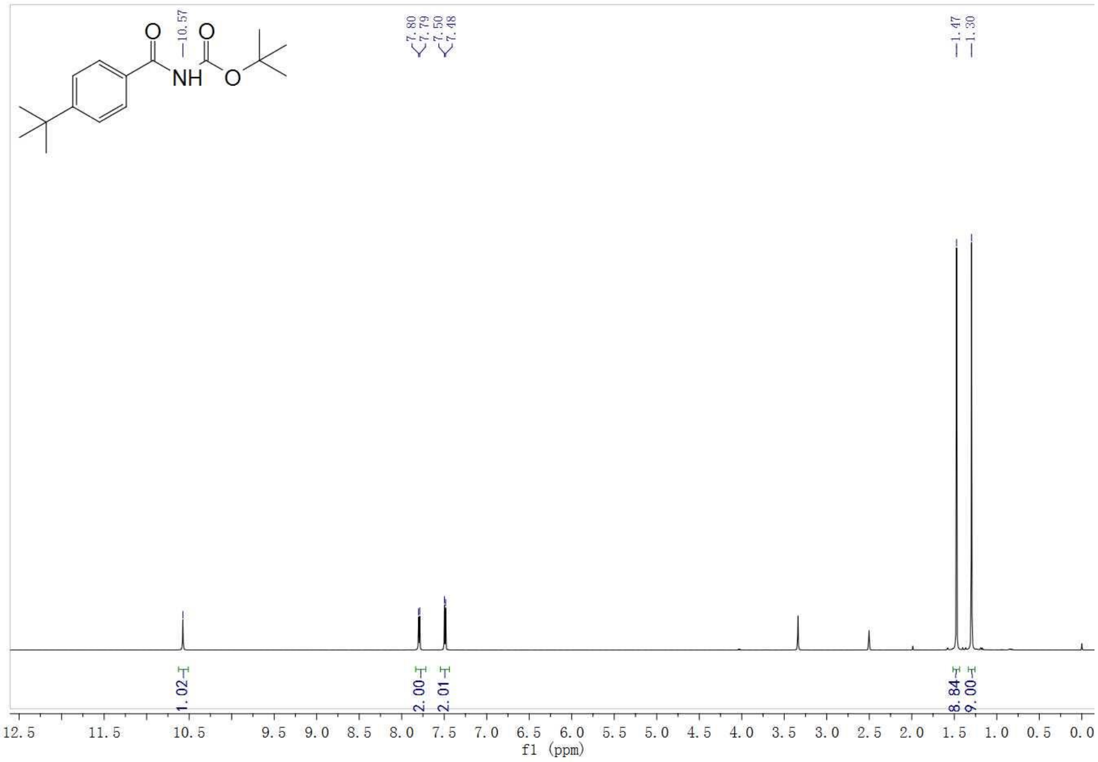

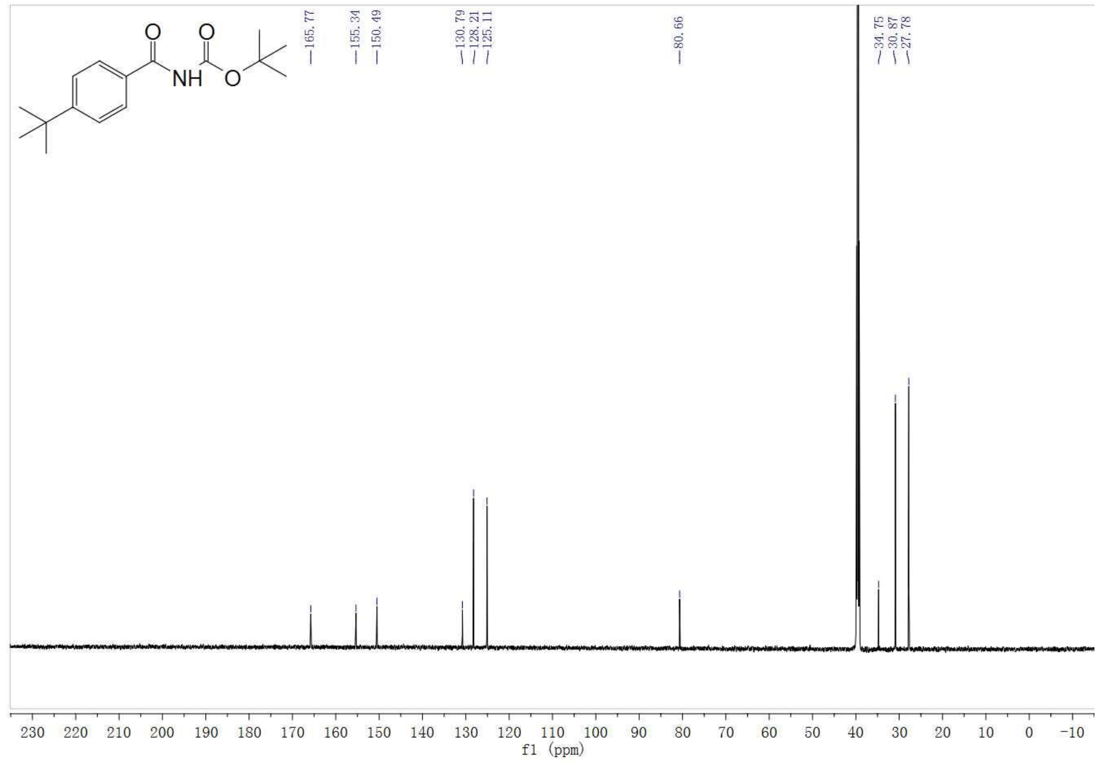

tert-butyl (4-fluorobenzoyl)carbamate (1h).

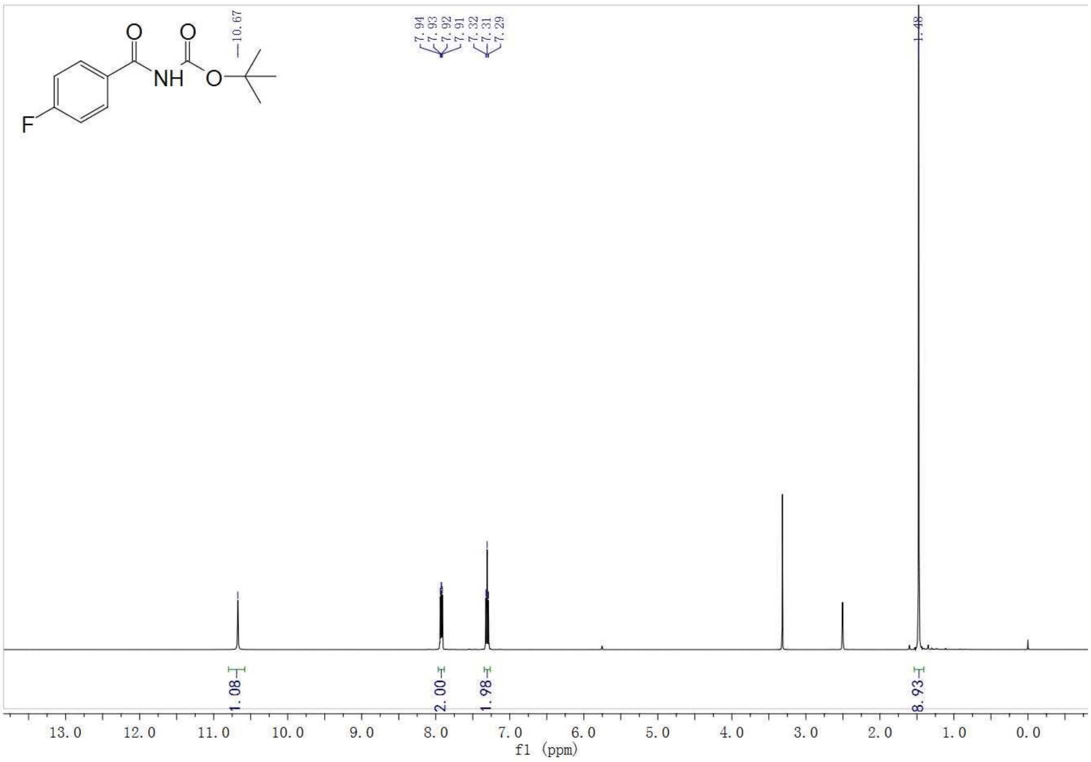

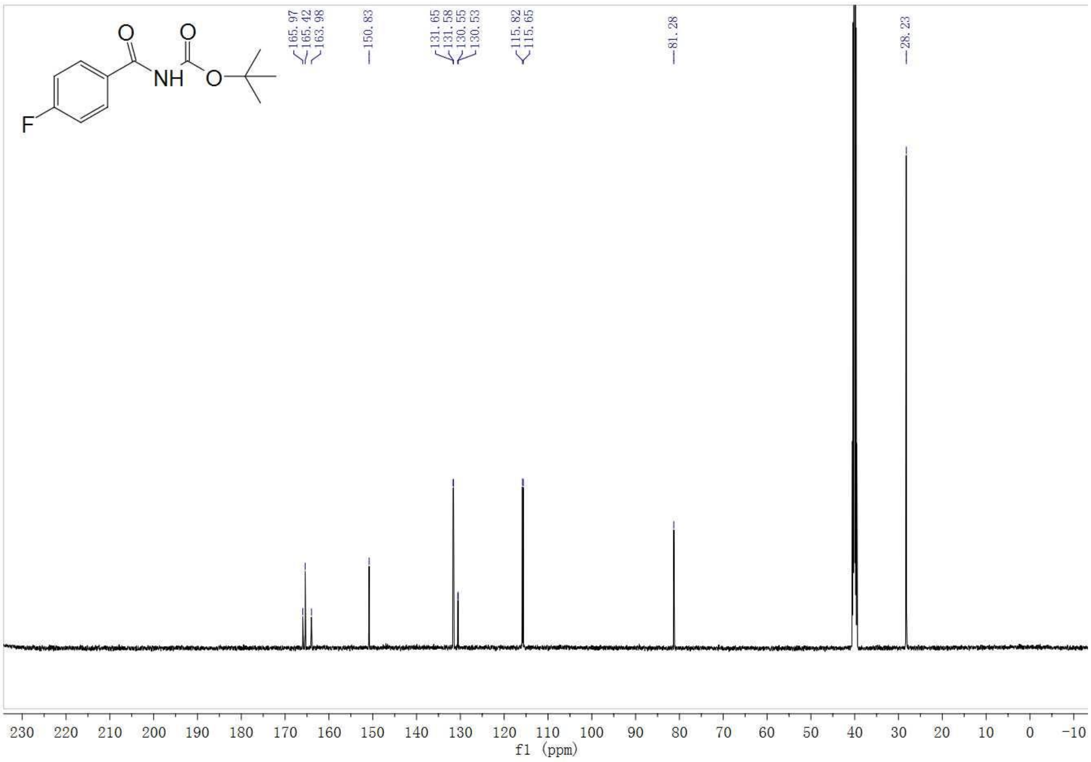

***tert*-butyl (4-chlorobenzoyl)carbamate (1g).**

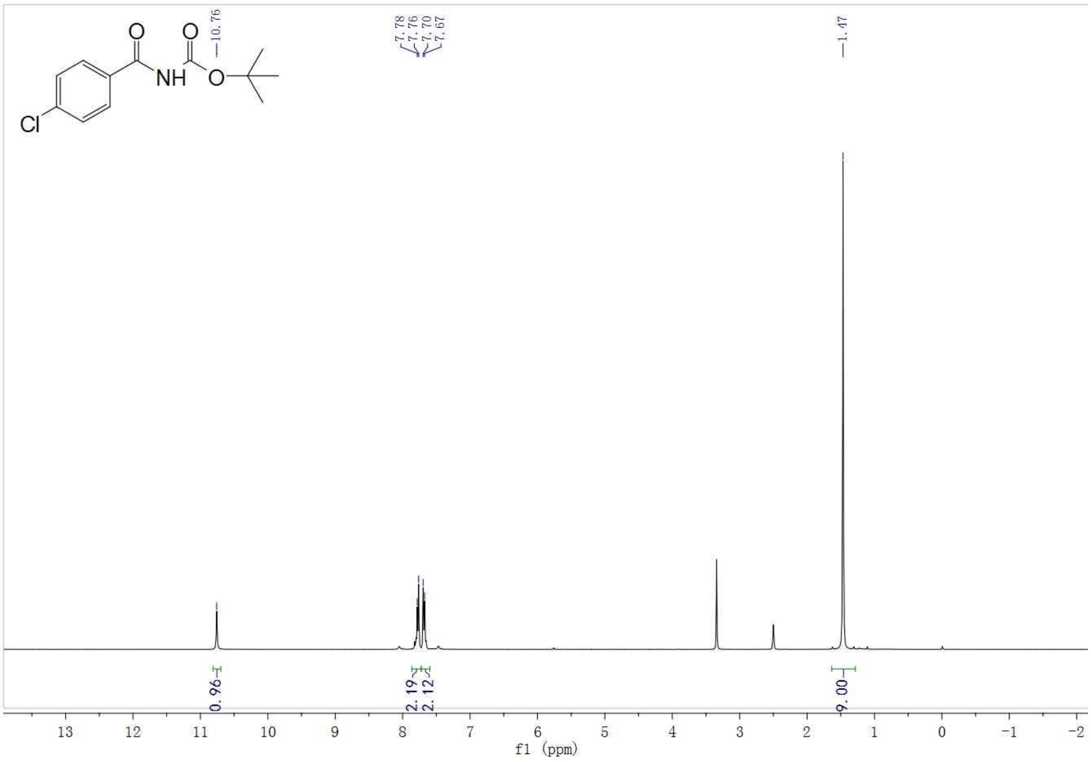

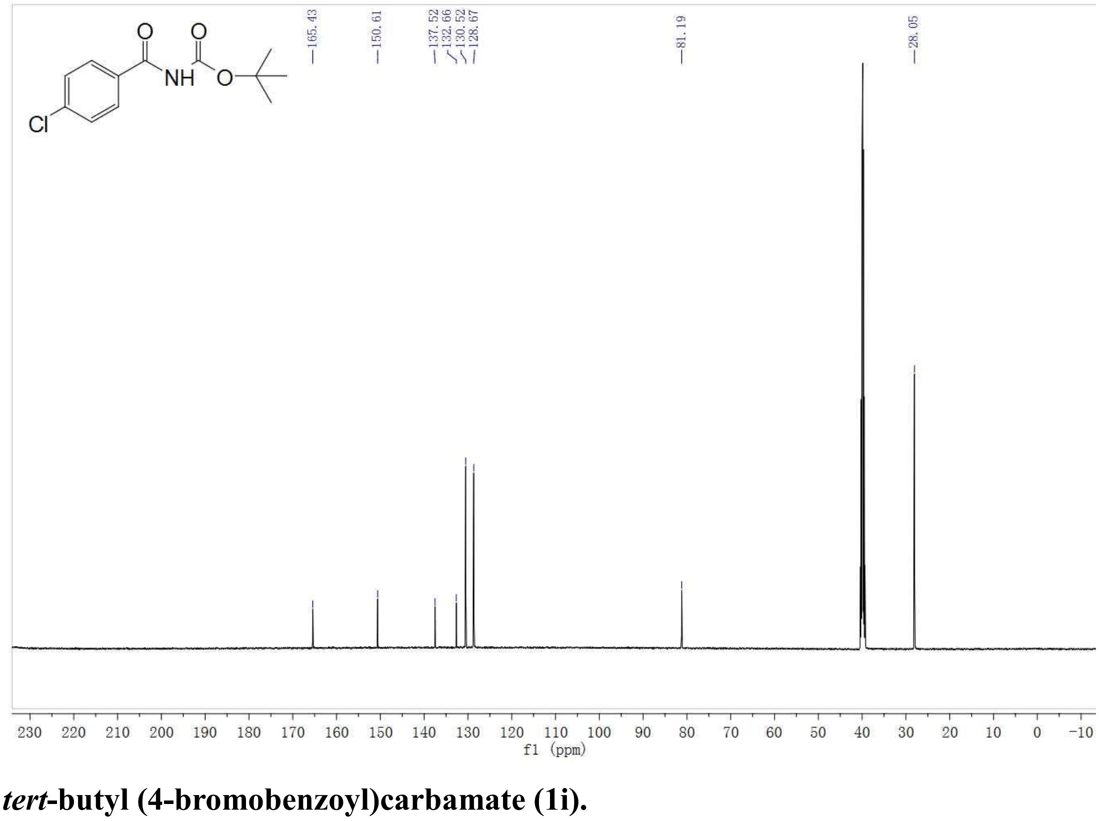

***tert*-butyl (4-bromobenzoyl)carbamate (1i).**

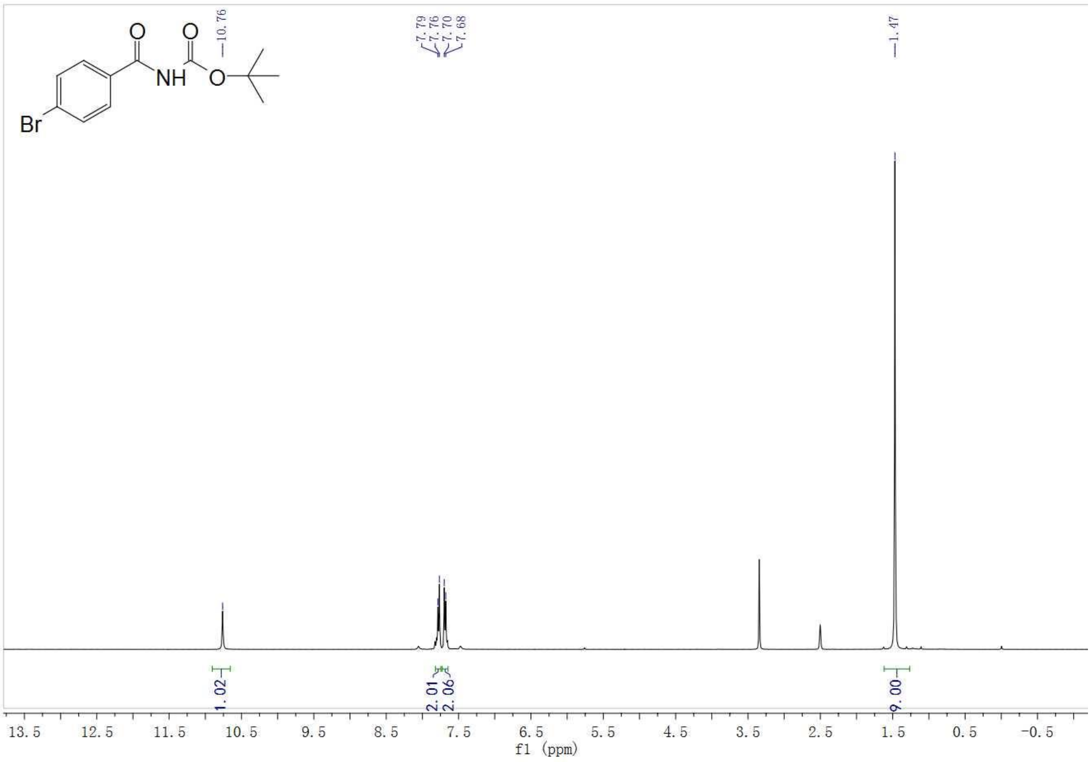

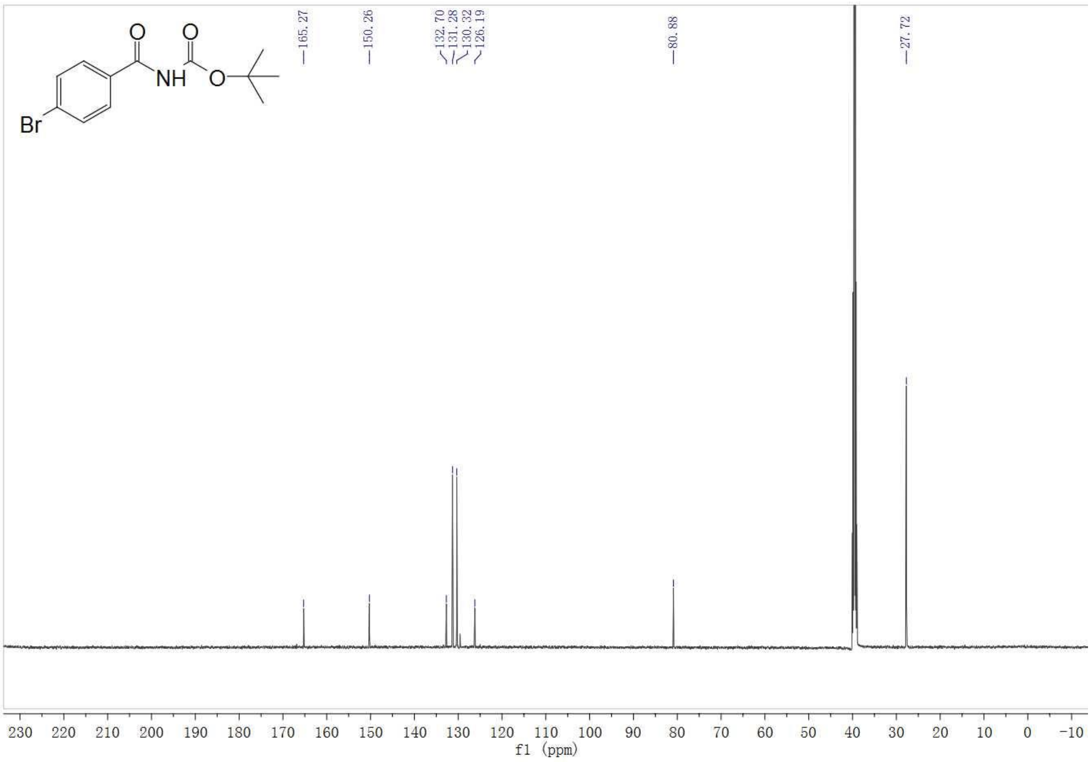

***tert*-Butyl (4-iodobenzoyl)carbamate (1j).**

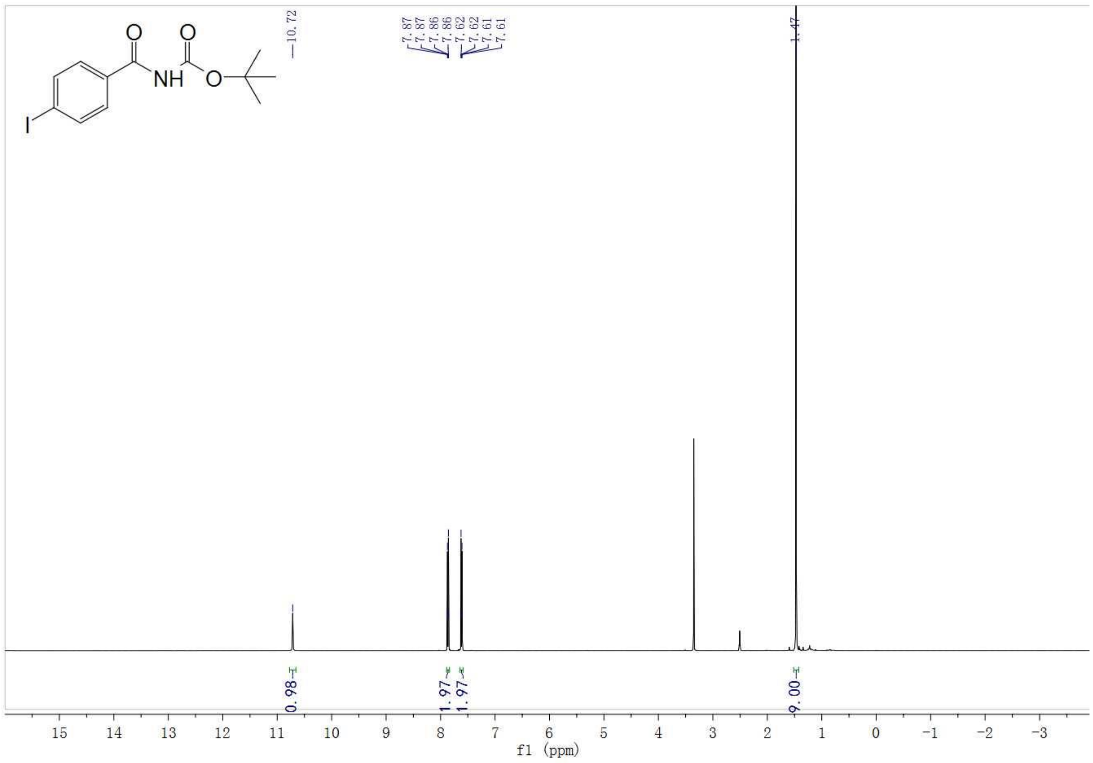

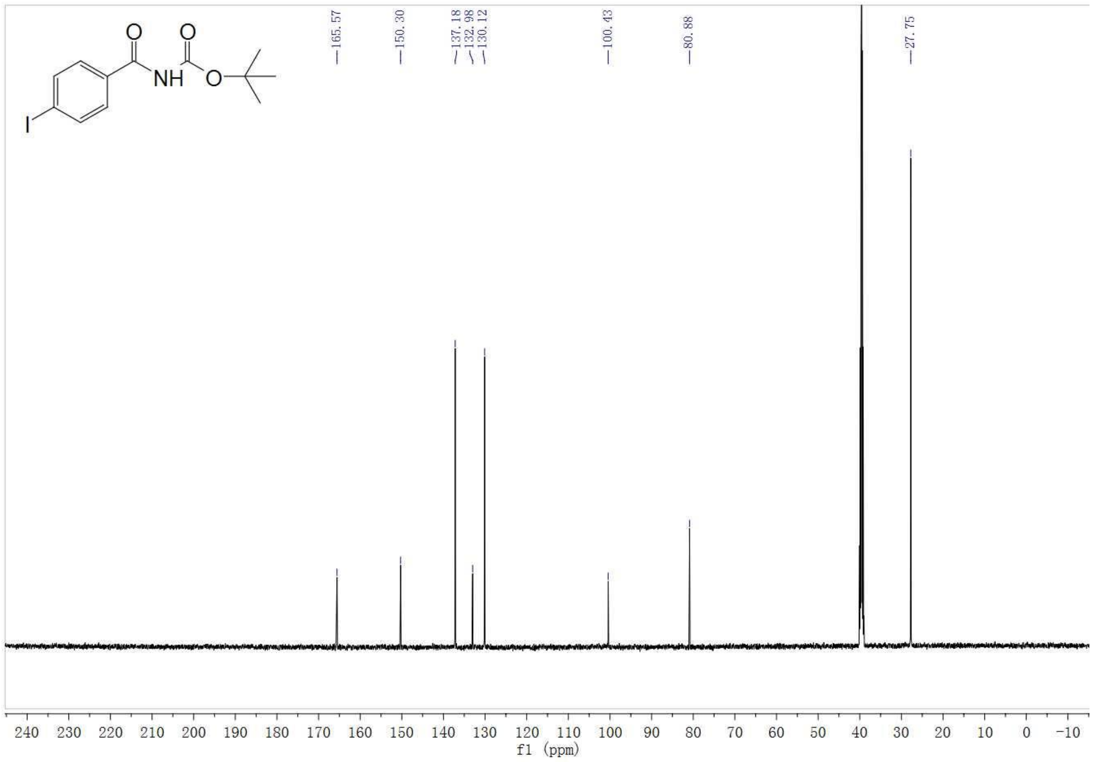

***tert*-Butyl (4-(trifluoromethyl)benzoyl)carbamate (1k).**

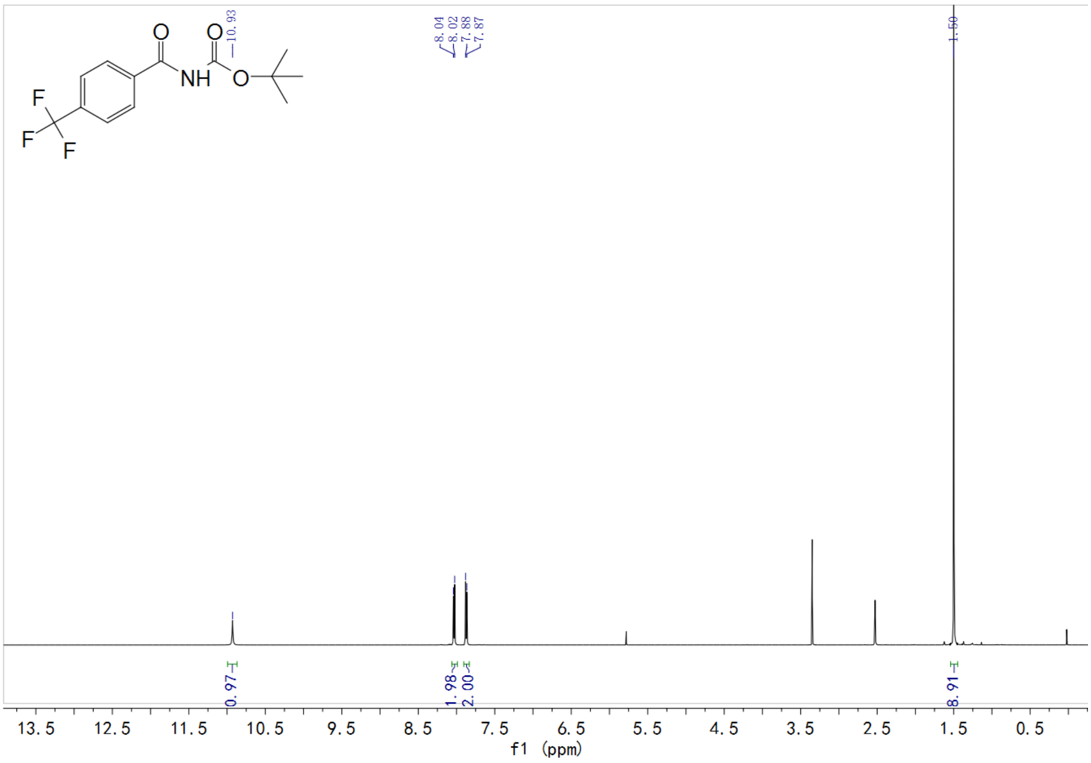

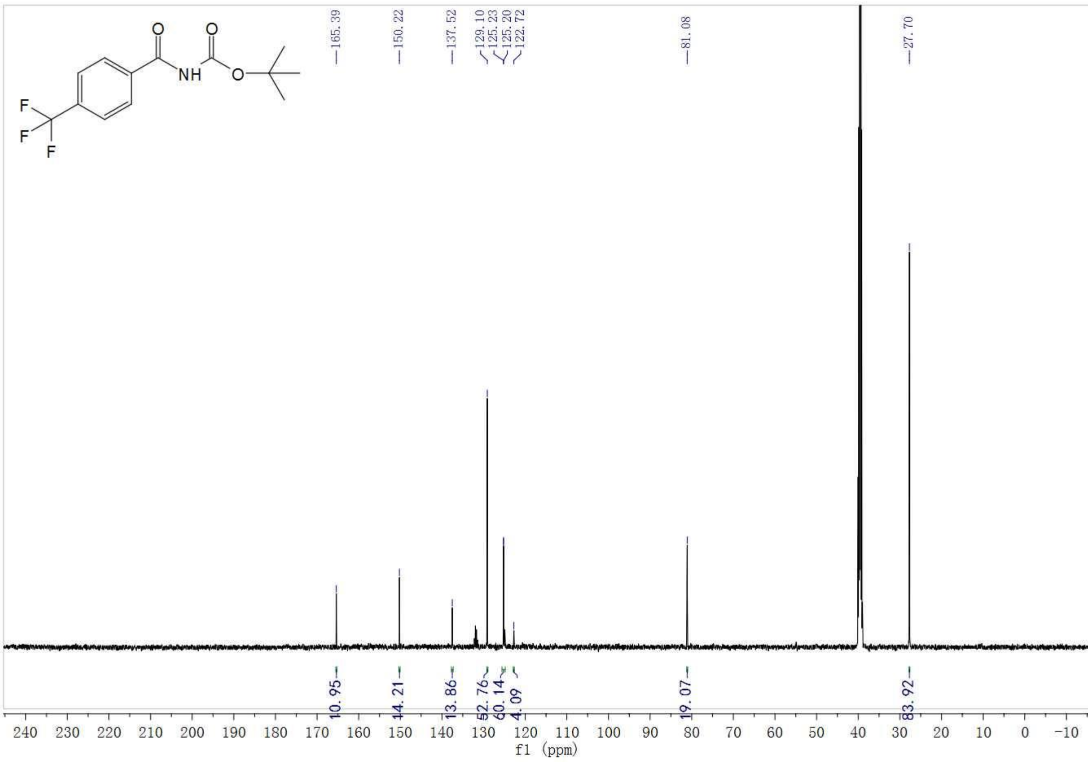

***tert*-Butyl (4-nitrobenzoyl)carbamate (1l).**

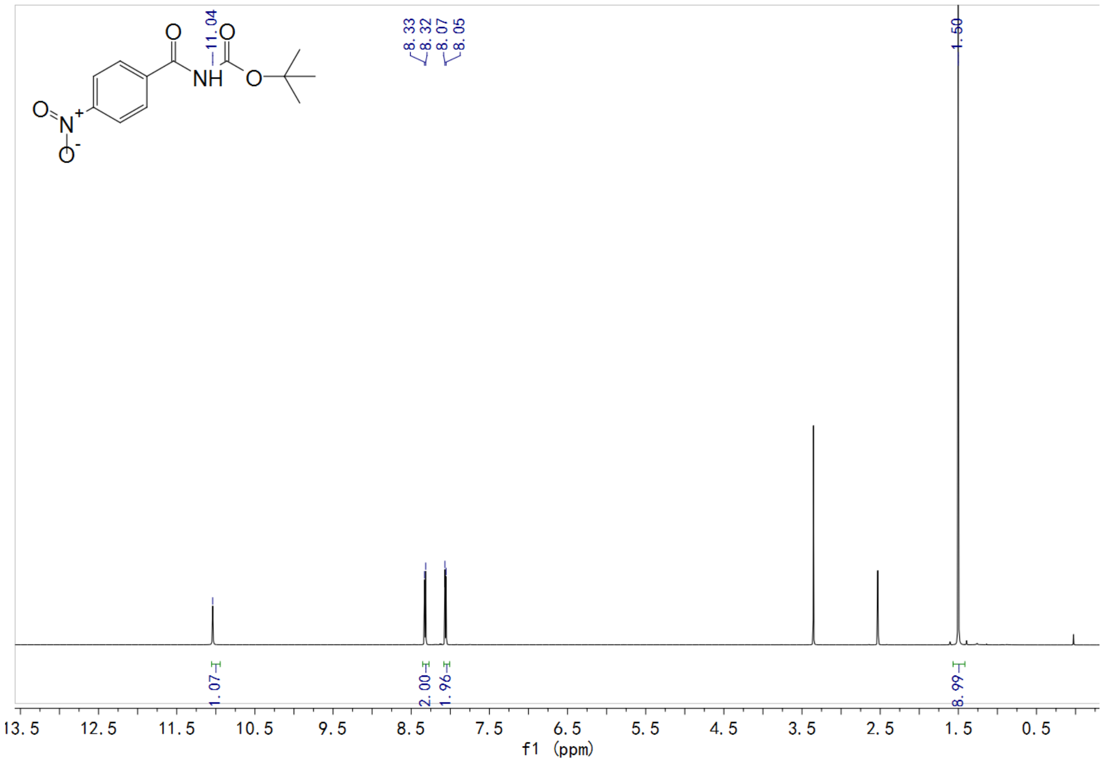

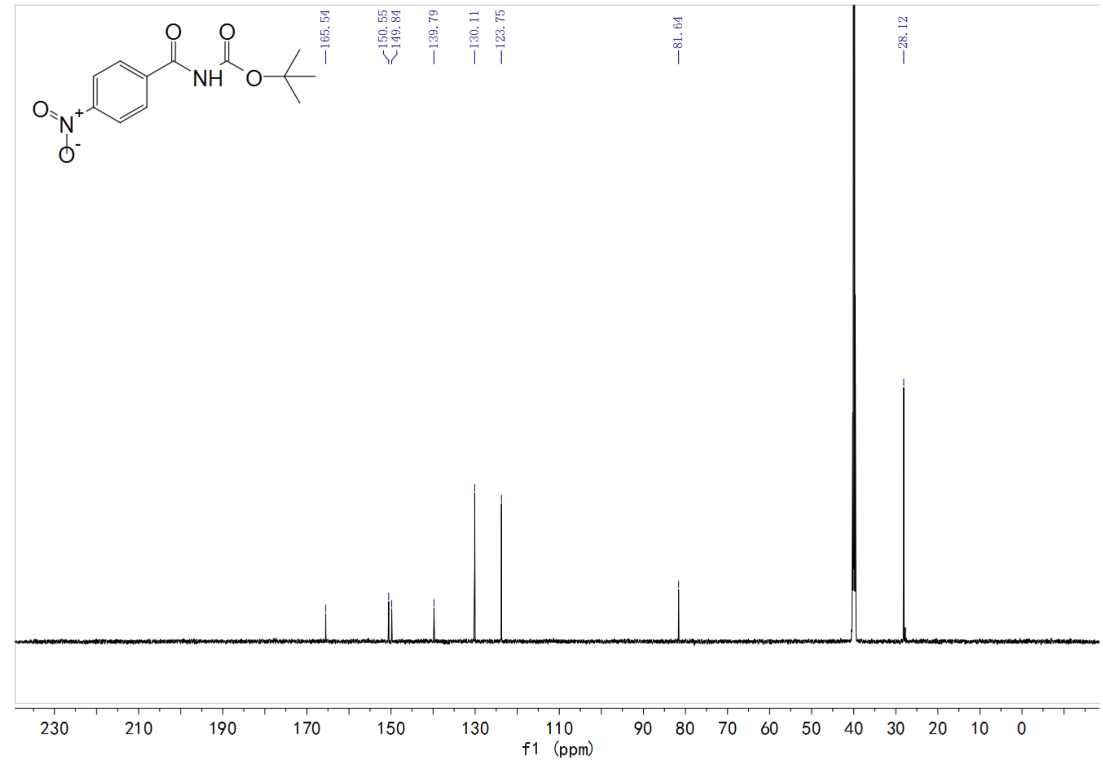

tert-Butyl (4-(benzyloxy)benzoyl)carbamate (1m).

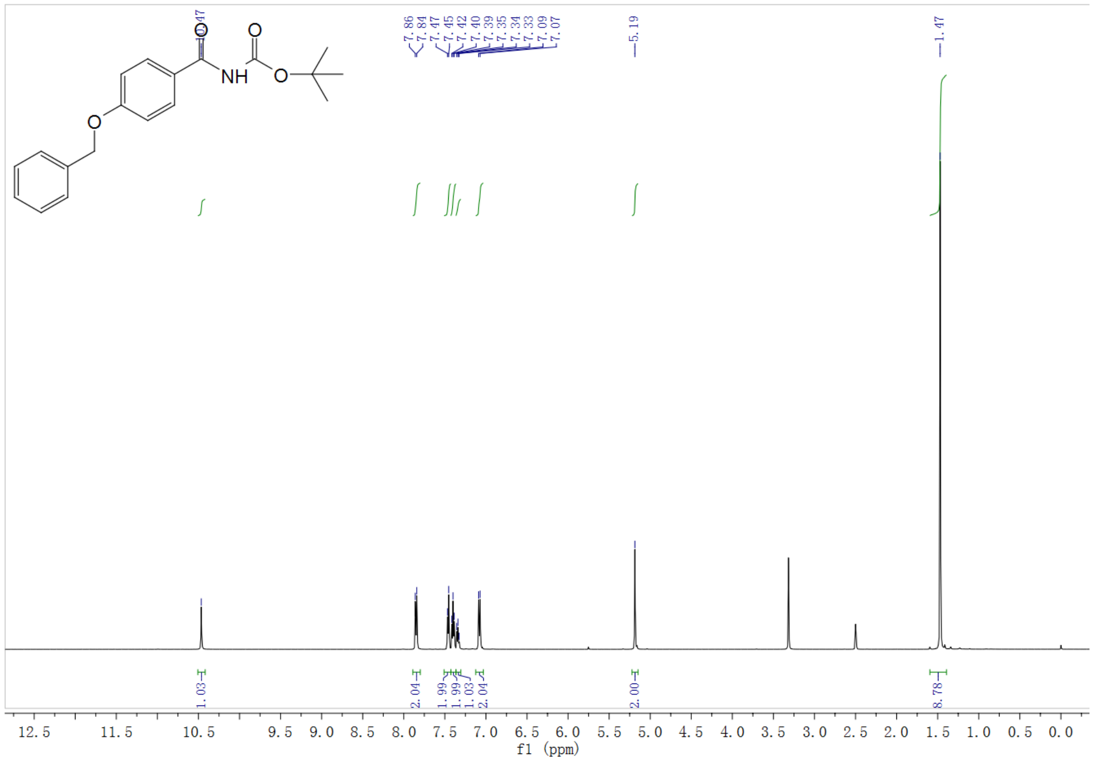

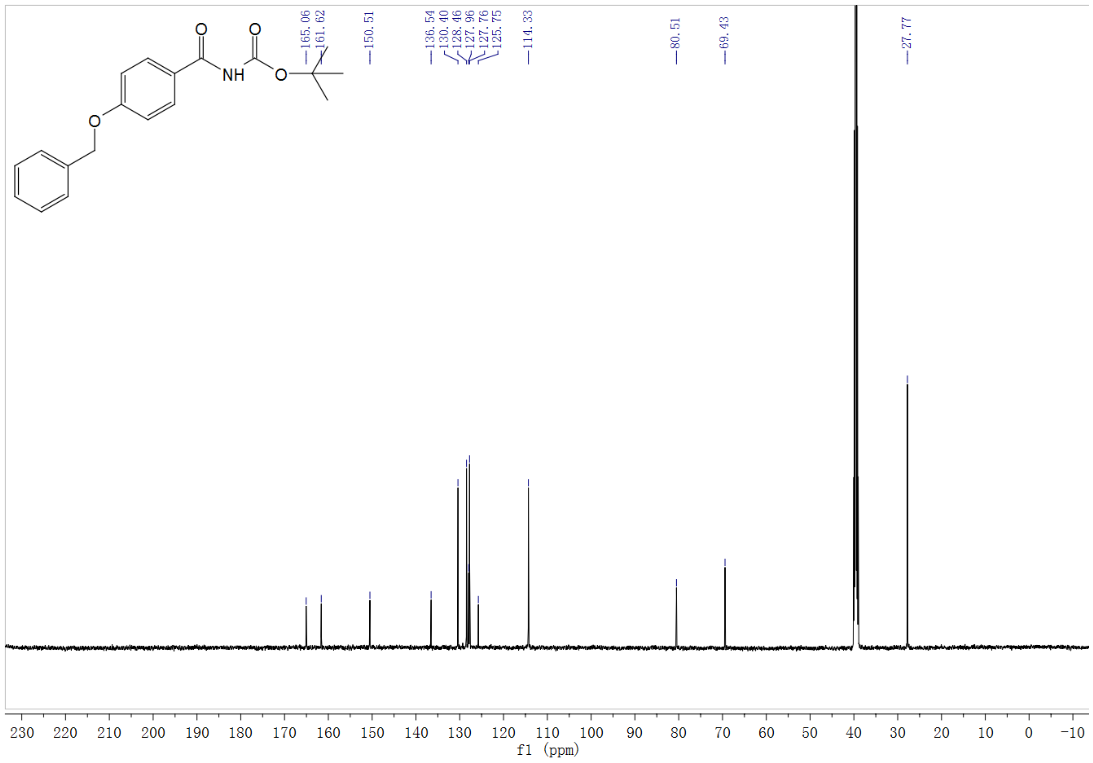

***tert*-Butyl (4-(dimethylamino)benzoyl)carbamate (1n).**

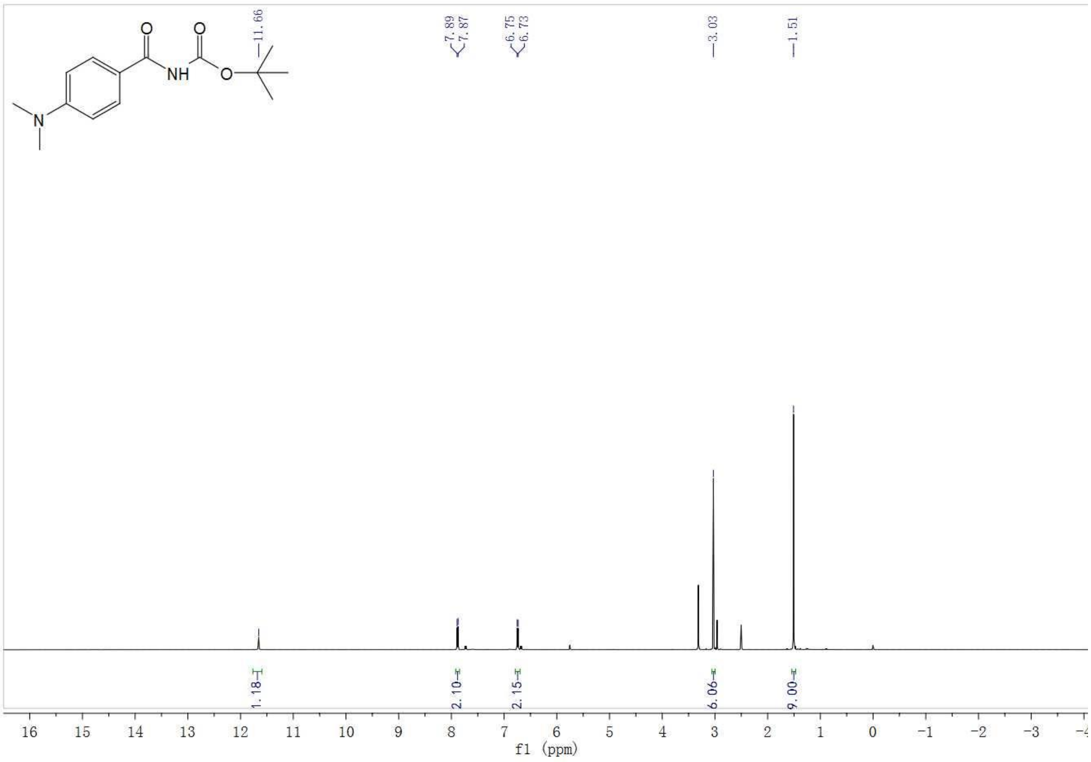

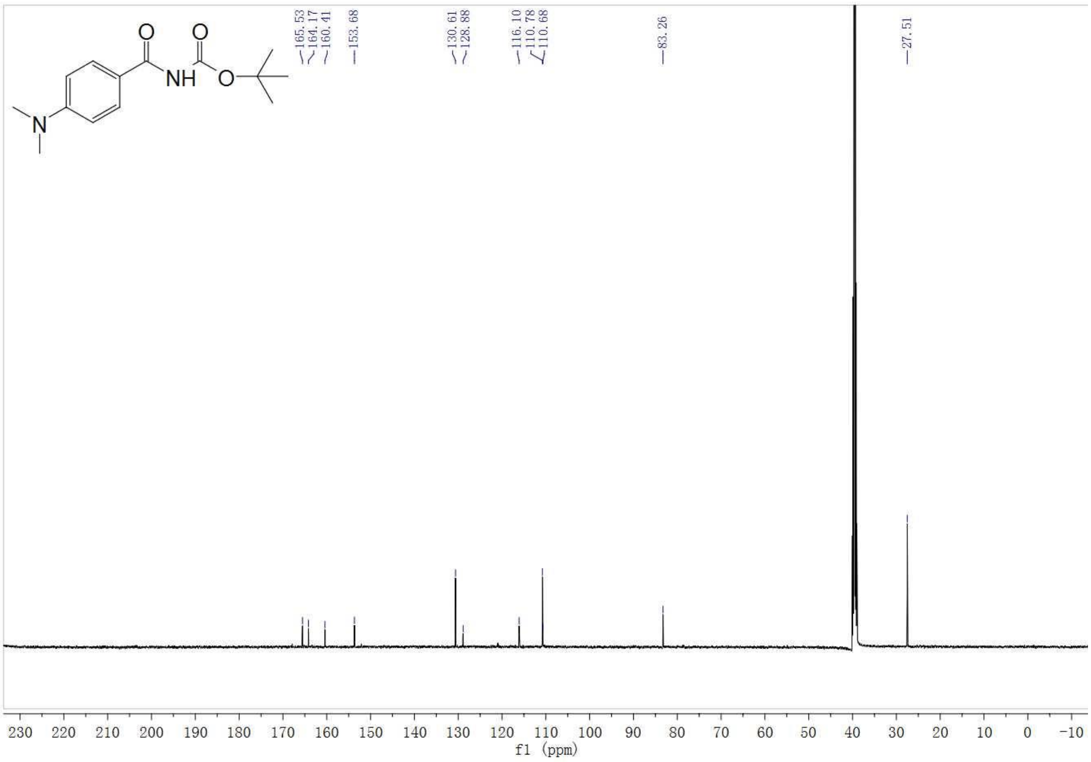

***tert*-Butyl [1,1'-biphenyl]-4-carbonylcarbamate (1o).**

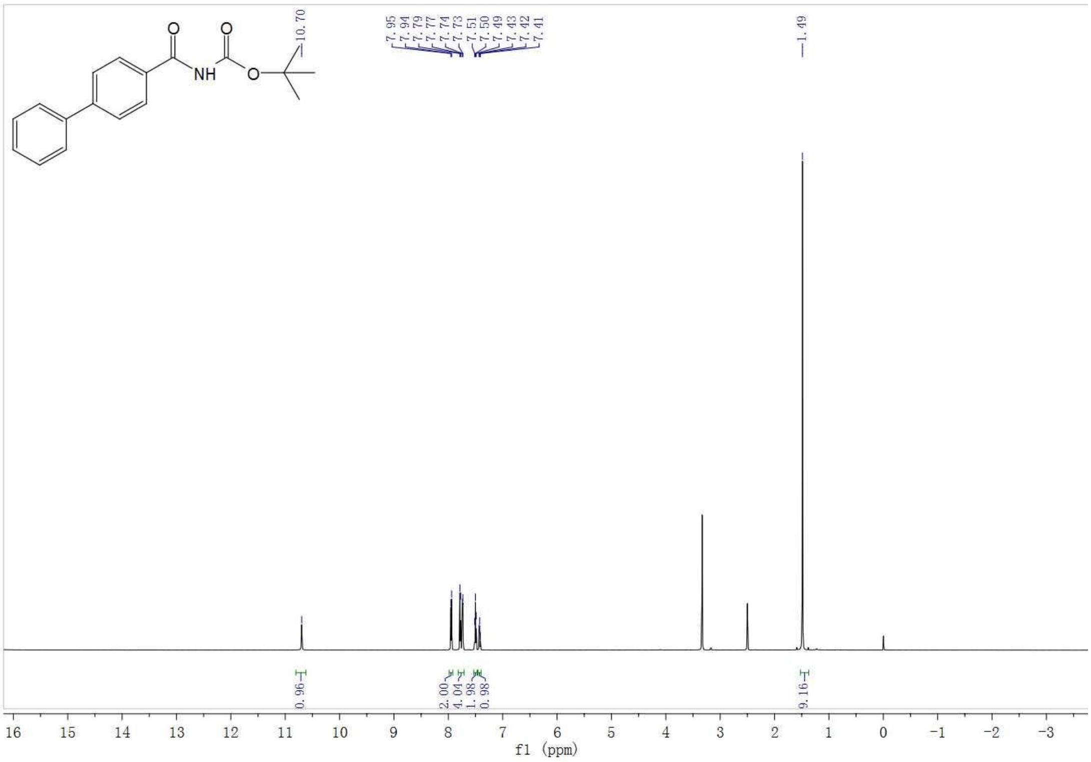

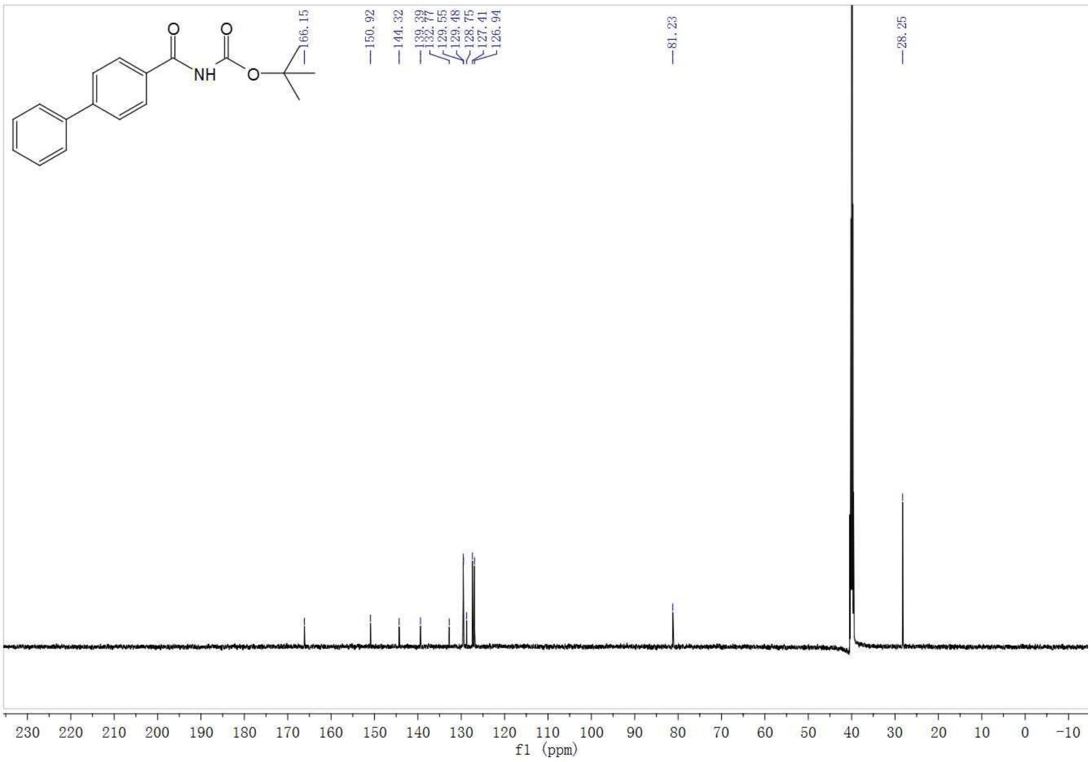

***tert*-Butyl (3-methylbenzoyl)carbamate (1p).**

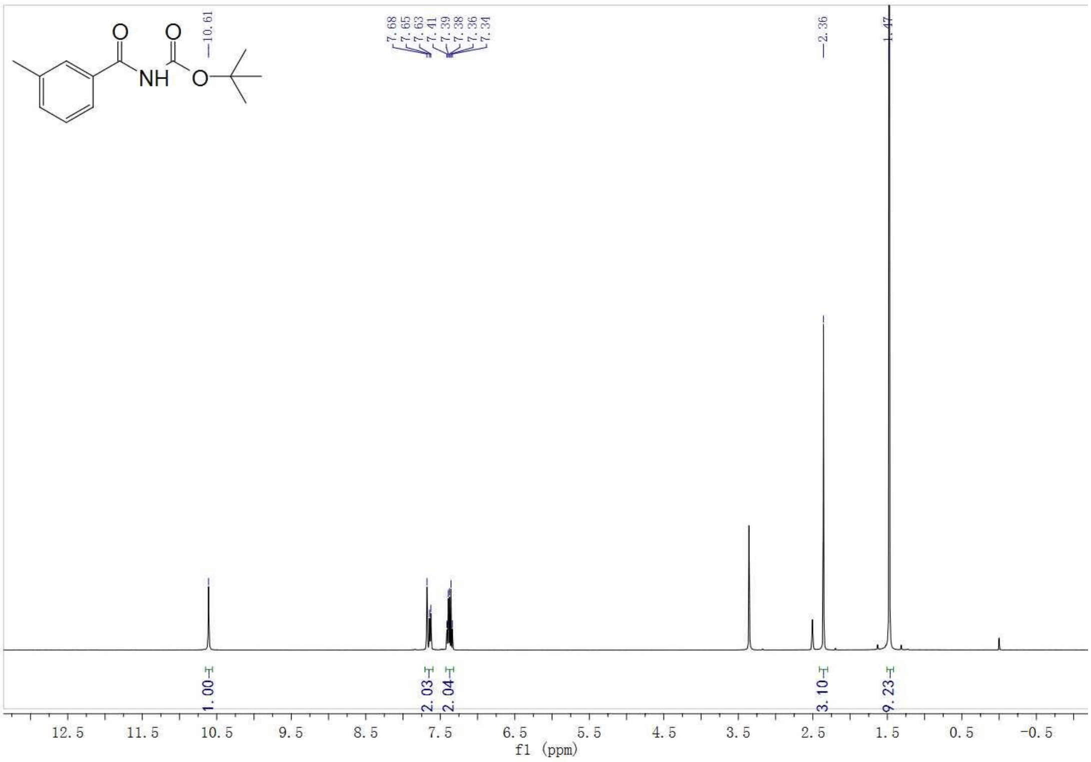

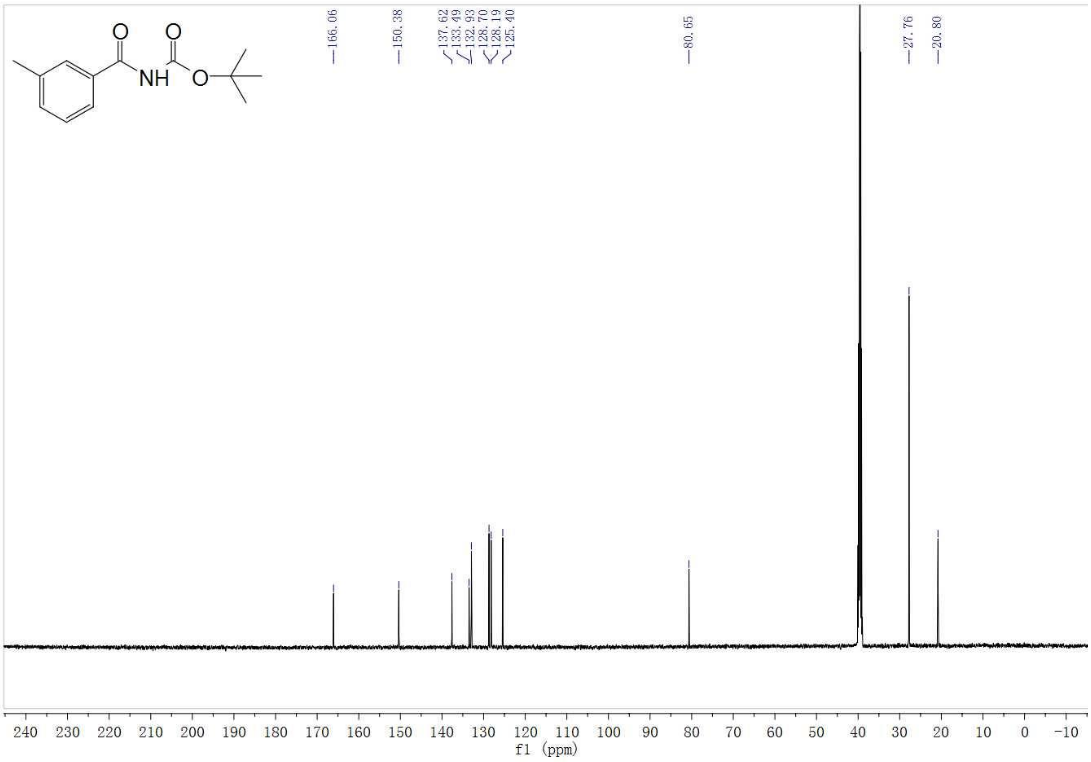

***tert*-Butyl (3-chlorobenzoyl)carbamate (1q).**

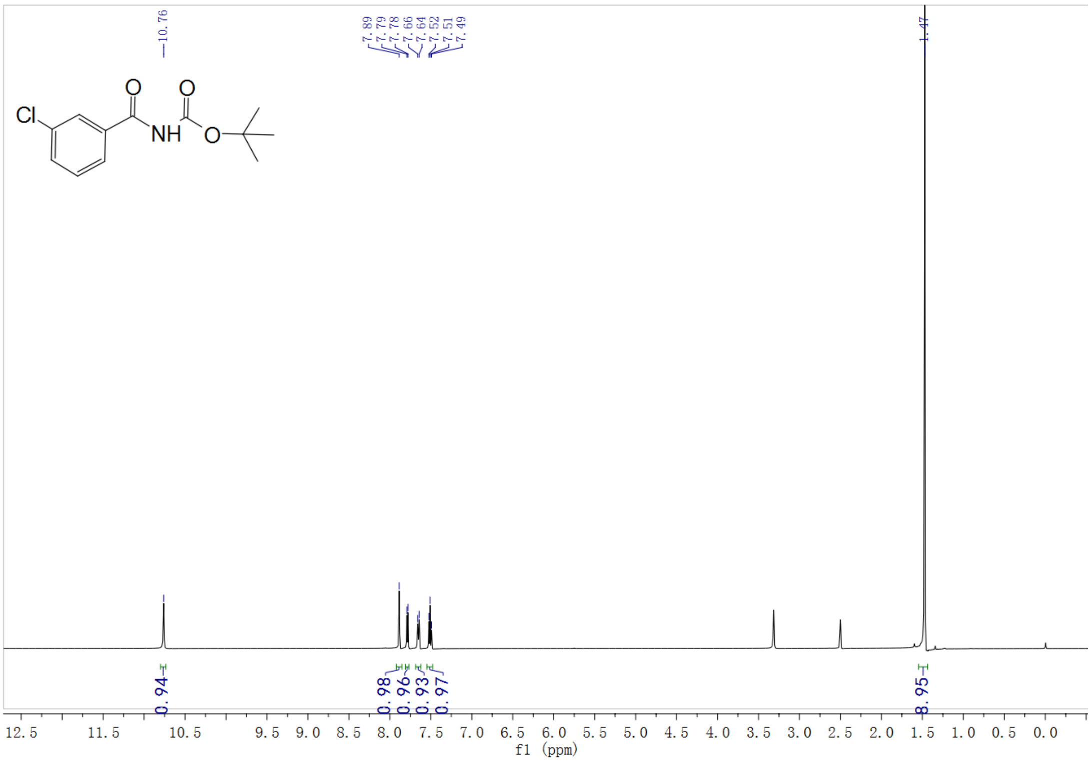

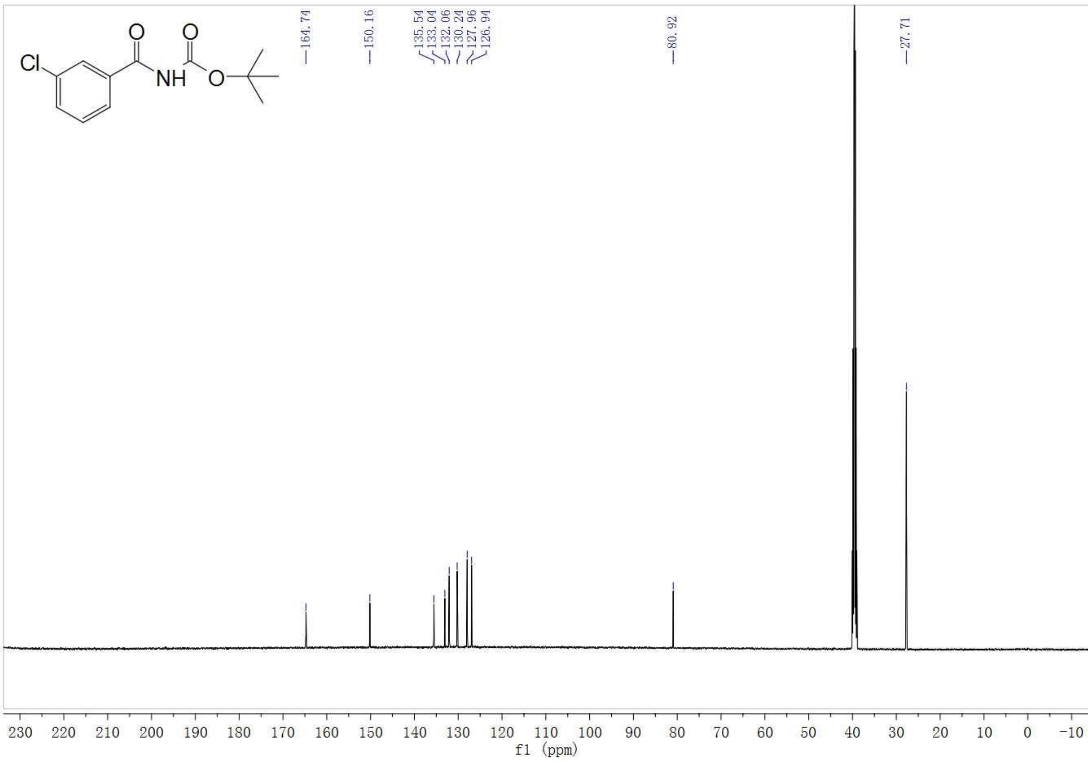

tert-Butyl (2-methylbenzoyl)carbamate (1r).

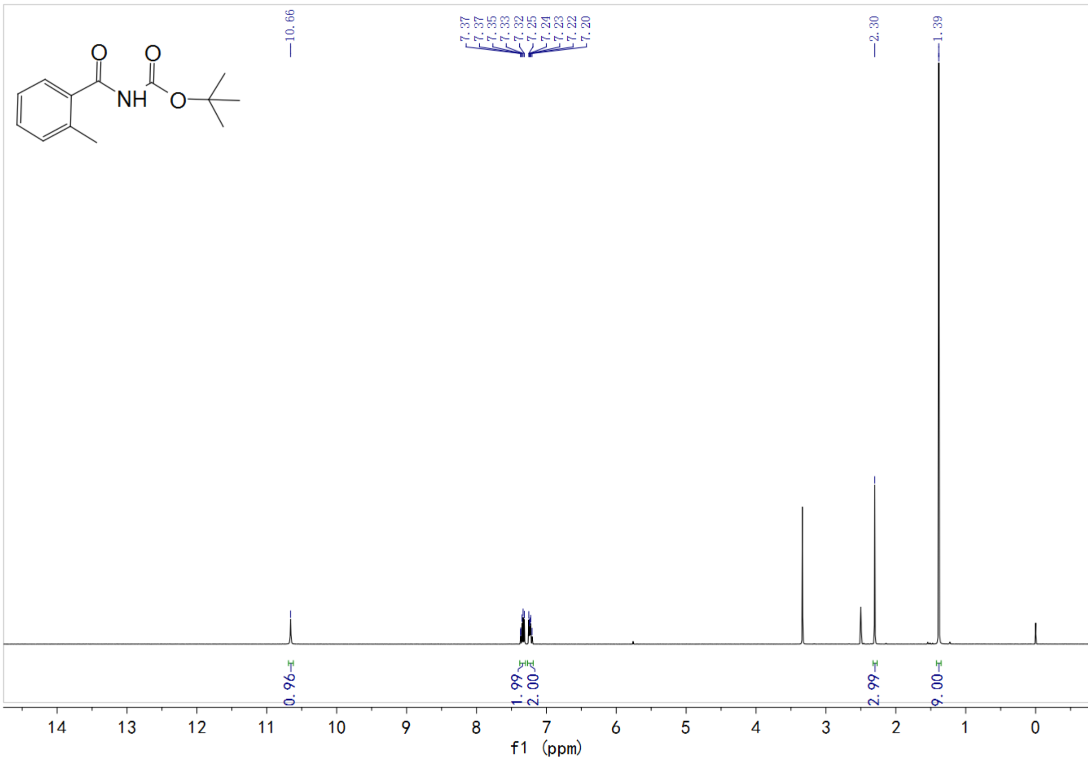

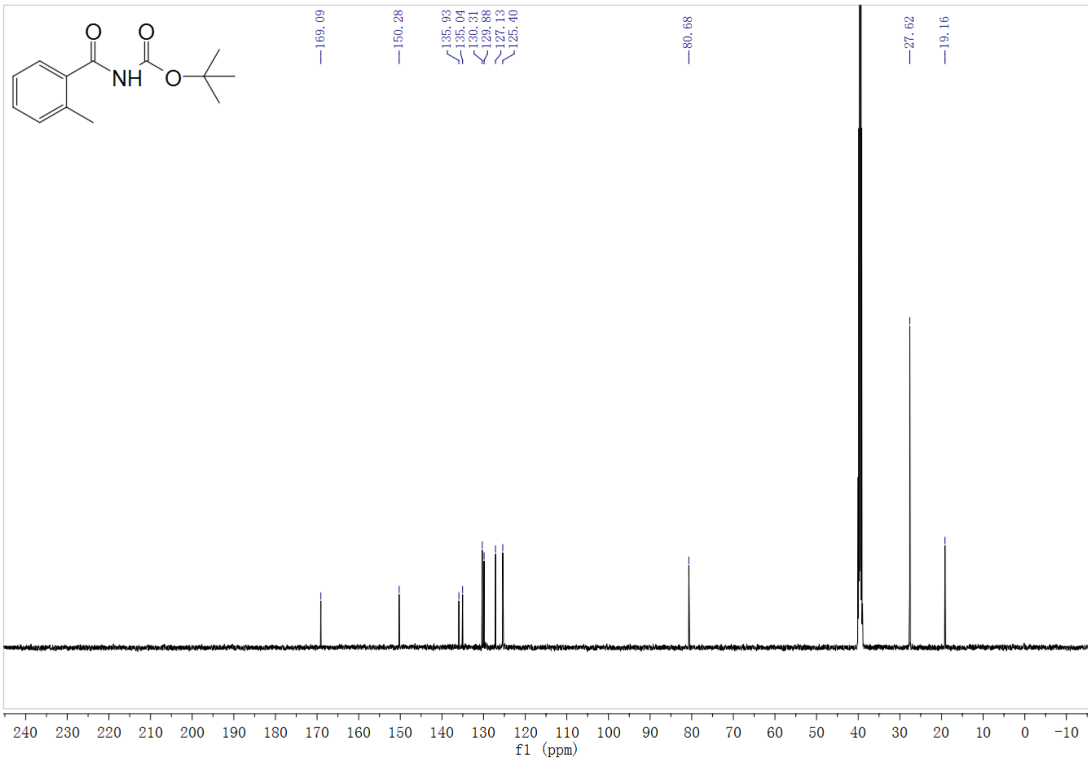

***tert*-Butyl (2-chlorobenzoyl)carbamate(1s).**

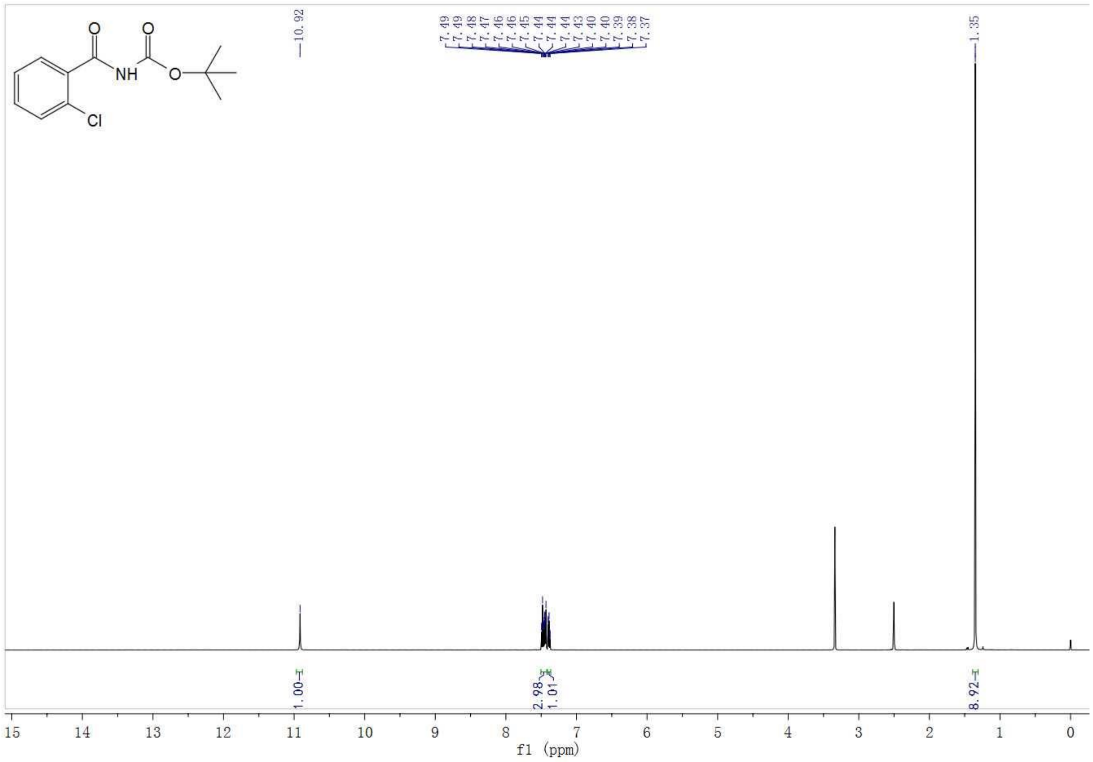

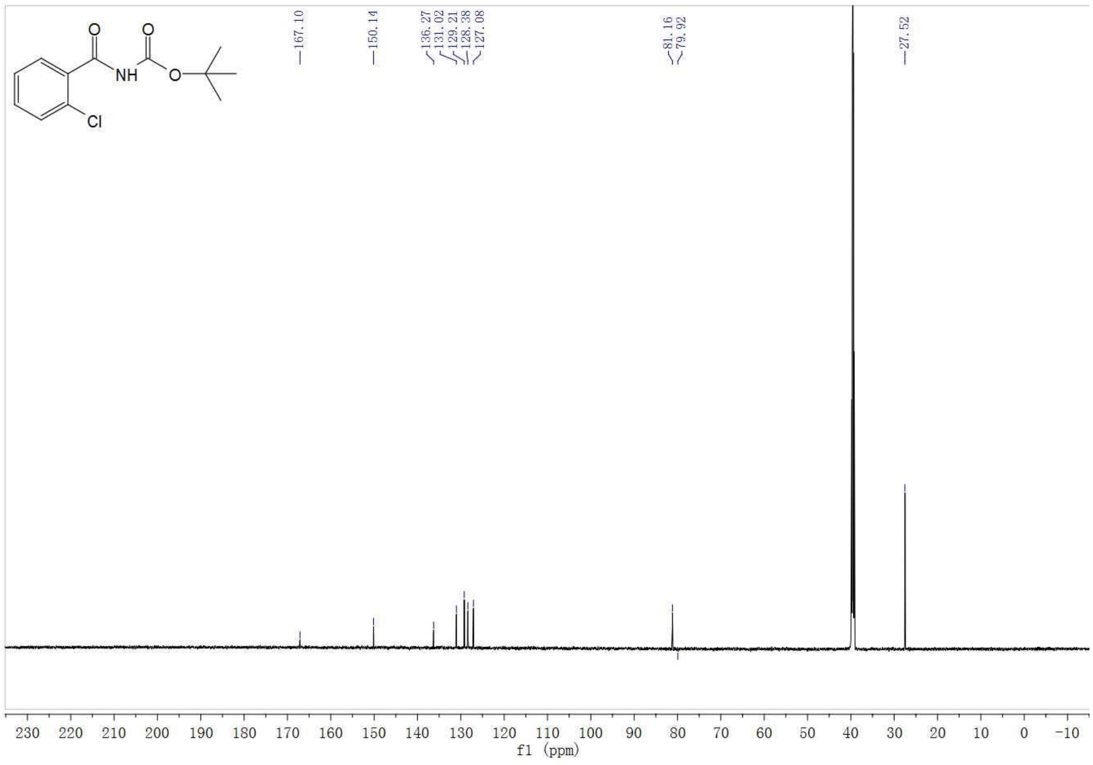

tert-Butyl (3,4-dimethylbenzoyl)carbamate(1t).

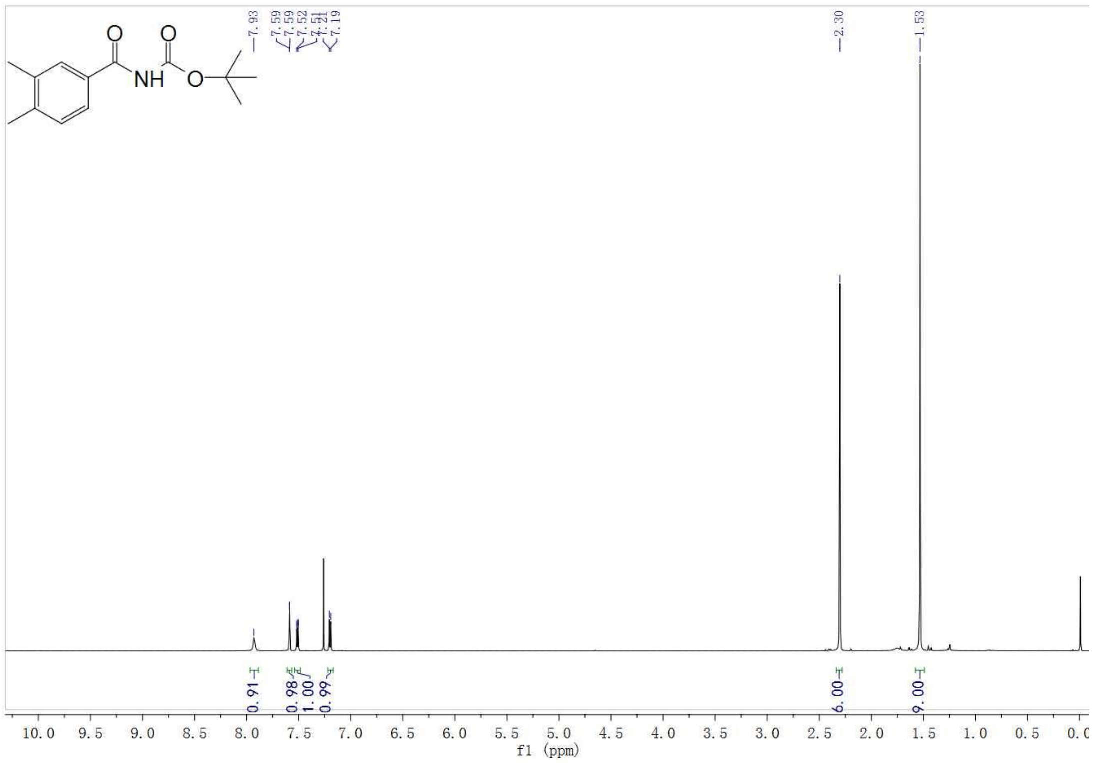

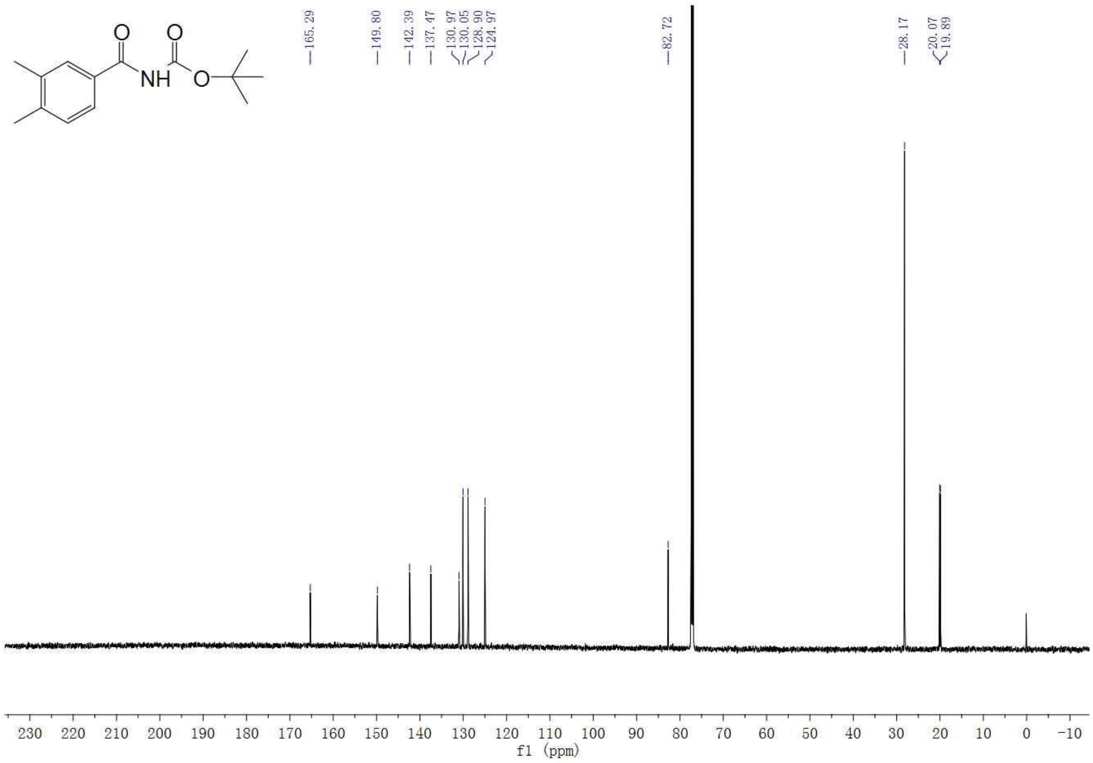

tert-Butyl 2-naphthoylcarbamate (1u).

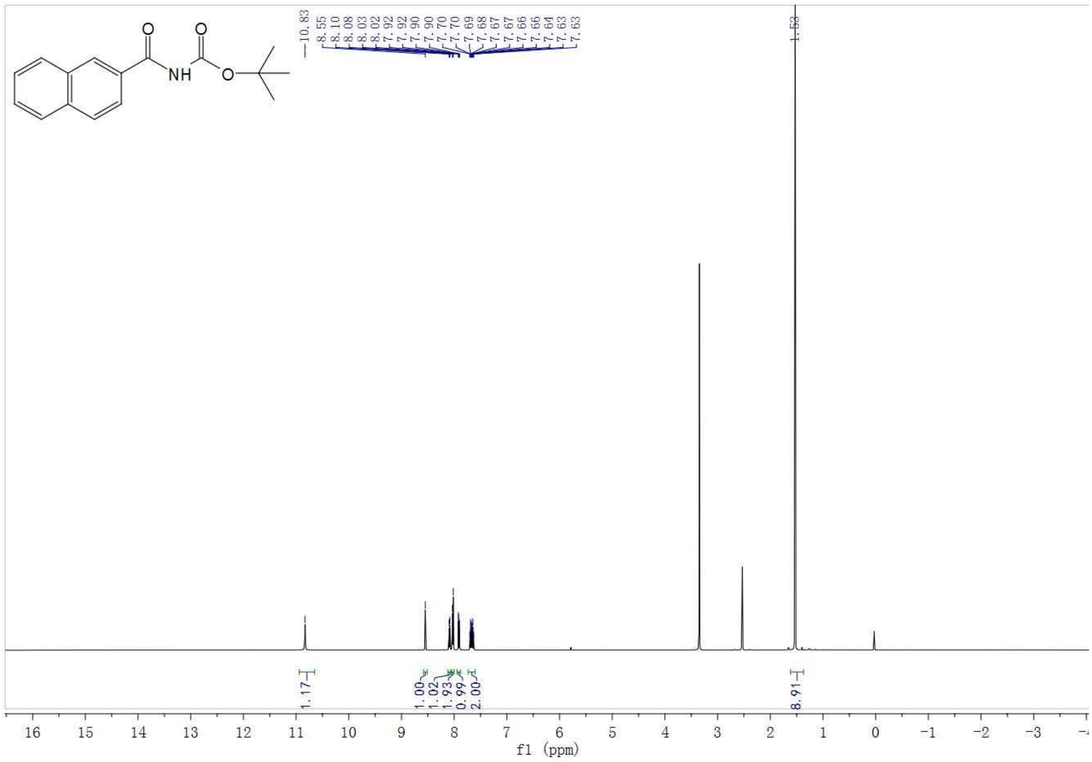

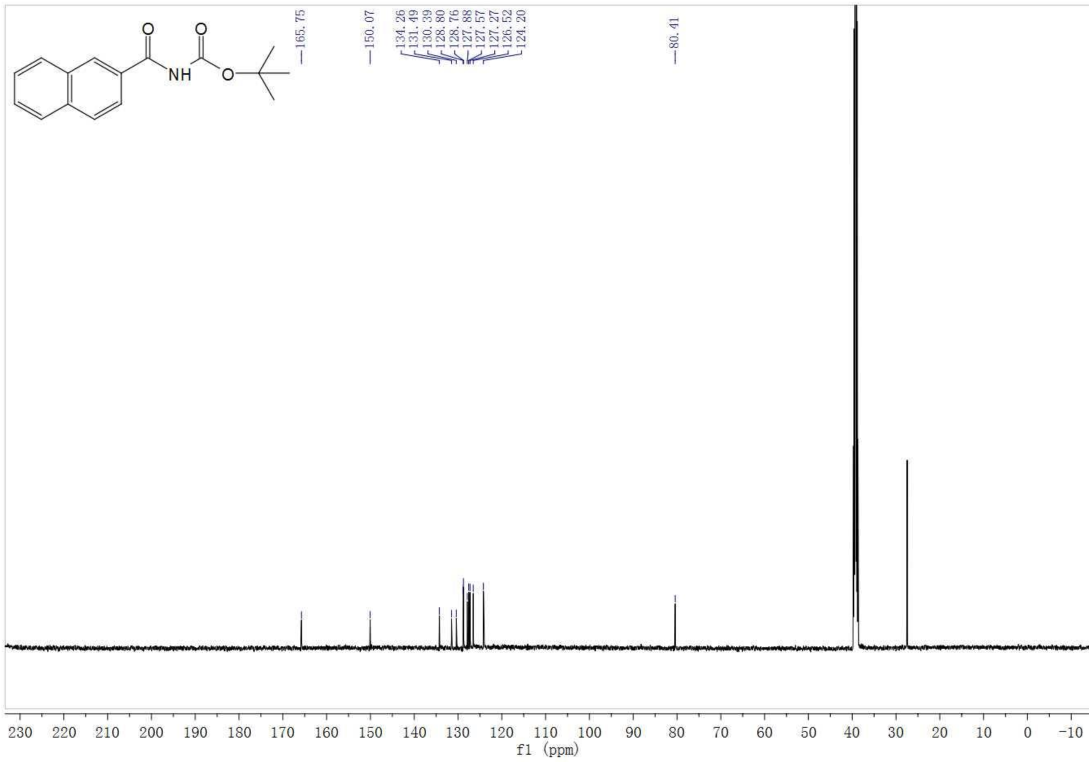

***tert*-Butyl thiophene-2-carbonylcarbamate (1v).**

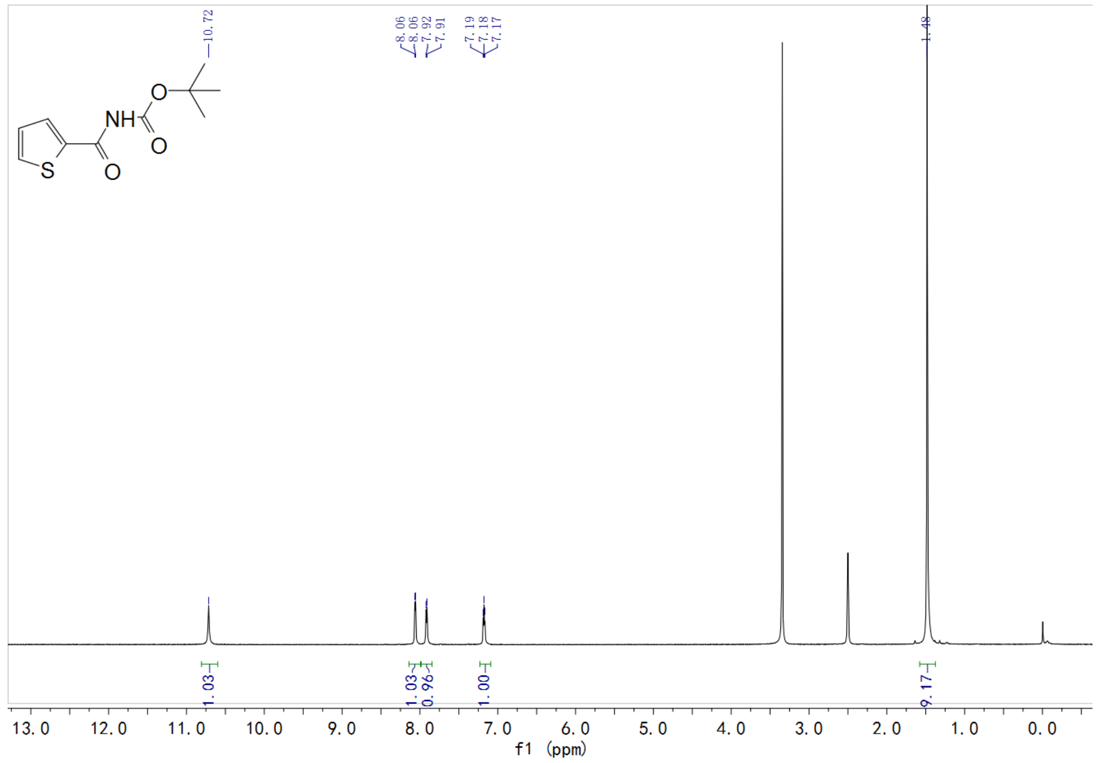

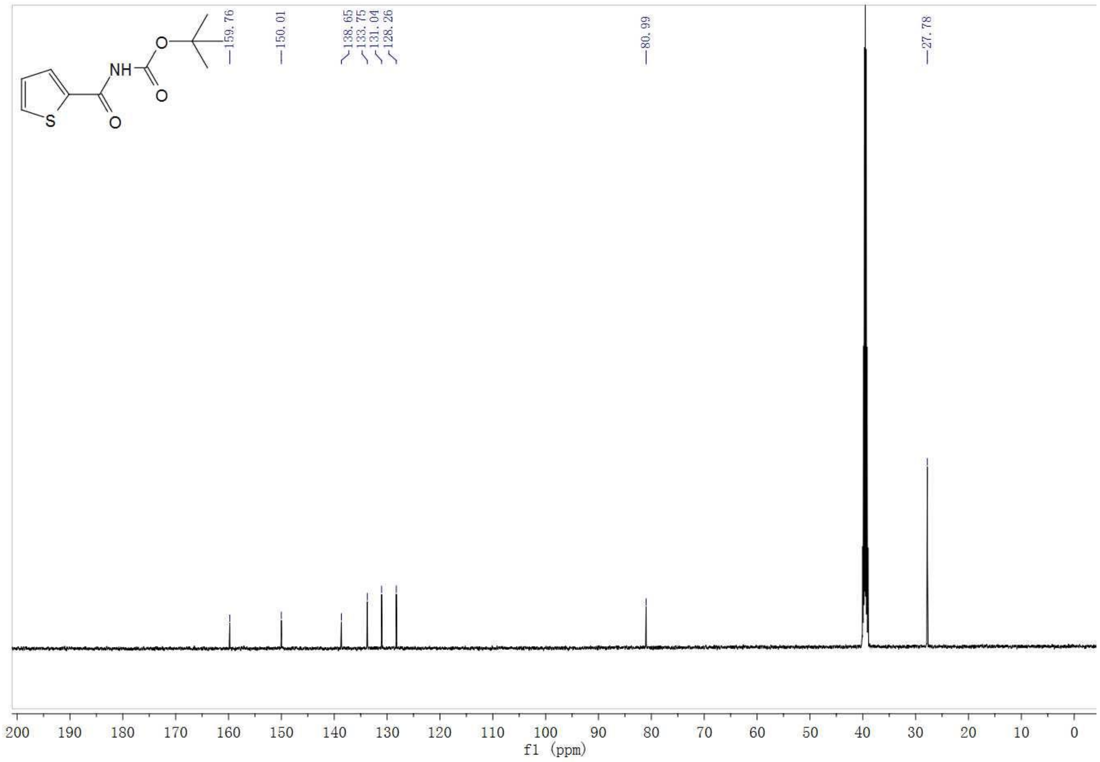

1a-d_5

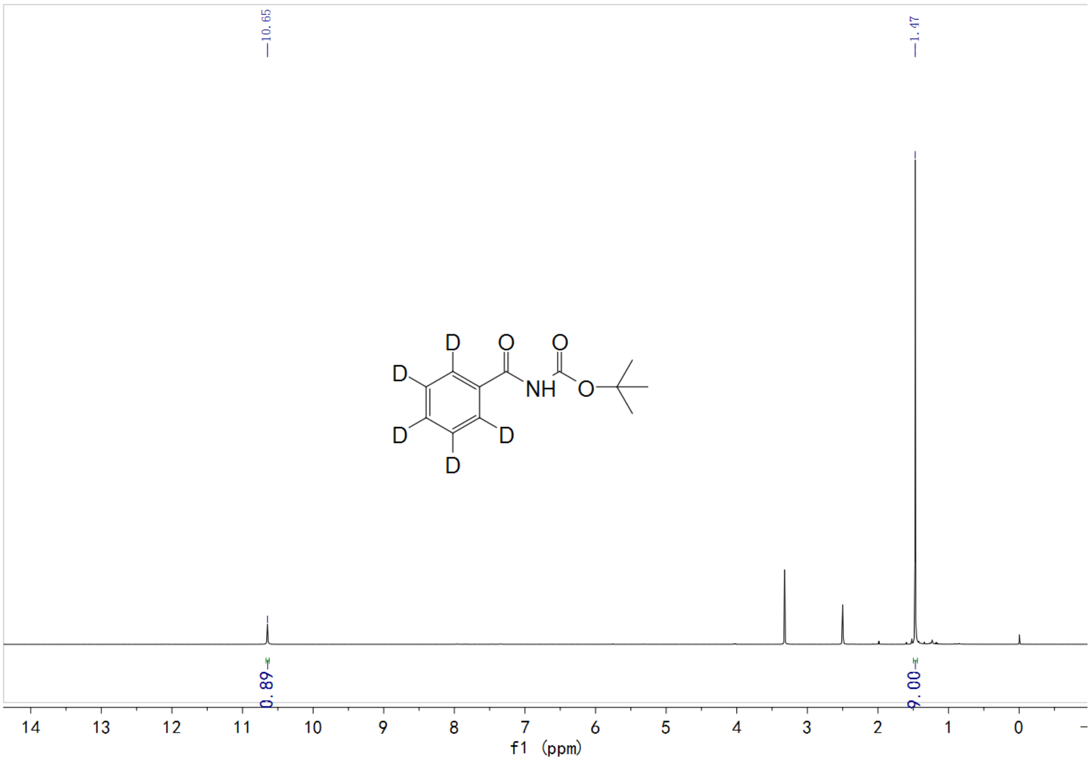
—10.65
—1.47
D
D
D
D
D
O
O
NH
O
0.89
9.00
14
13
12
11
10
9
8
7
6
5
4
3
2
1
0
f1 (ppm)

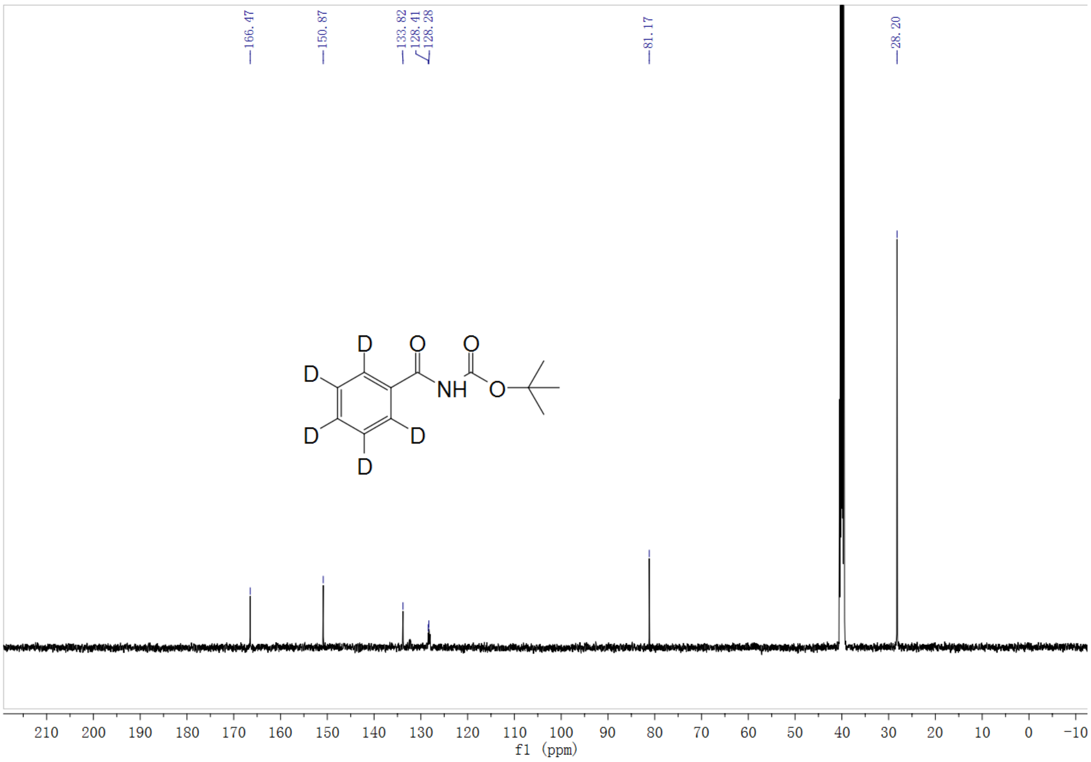
—166.47
—150.87
133.82
128.41
128.28
—81.17
—28.20
D
D
D
D
D
O
O
NH
O
210
200
190
180
170
160
150
140
130
120
110
100
90
80
70
60
50
40
30
20
10
0
−10
f1 (ppm)

7. ^{1}H and ^{13}C NMR Spectra of Products

Ethyl 3-methyl-1-oxo-1*H*-isochromene-4-carboxylate (3aa).

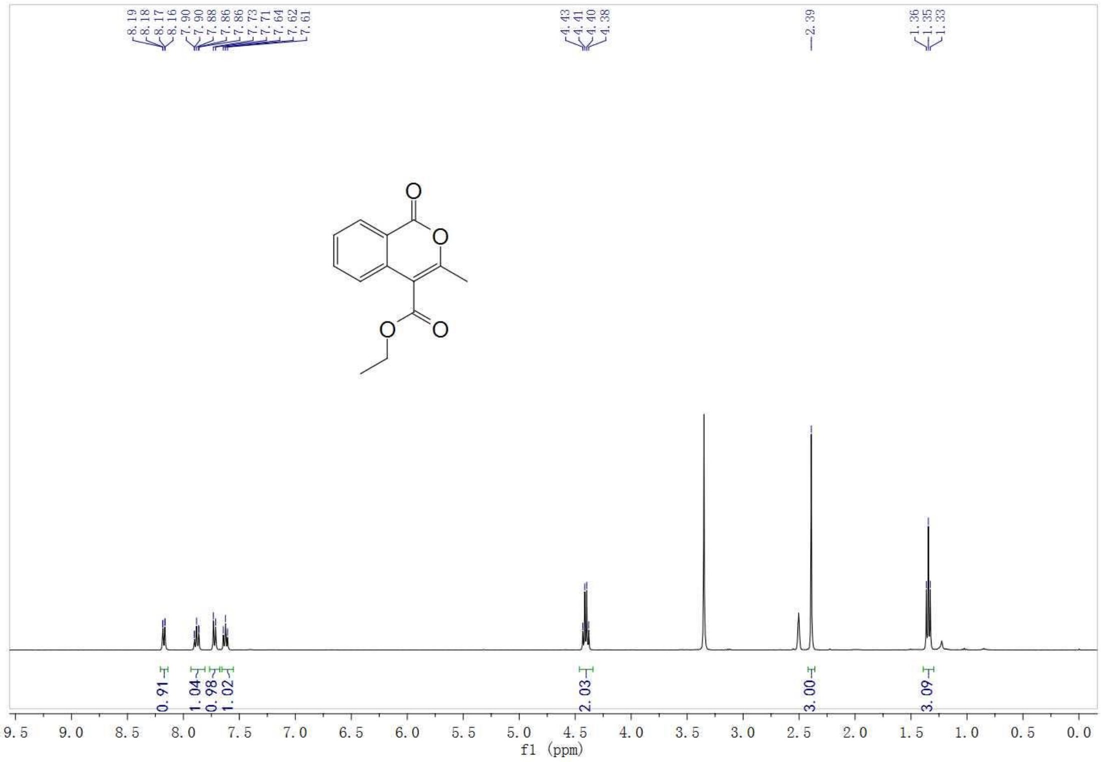

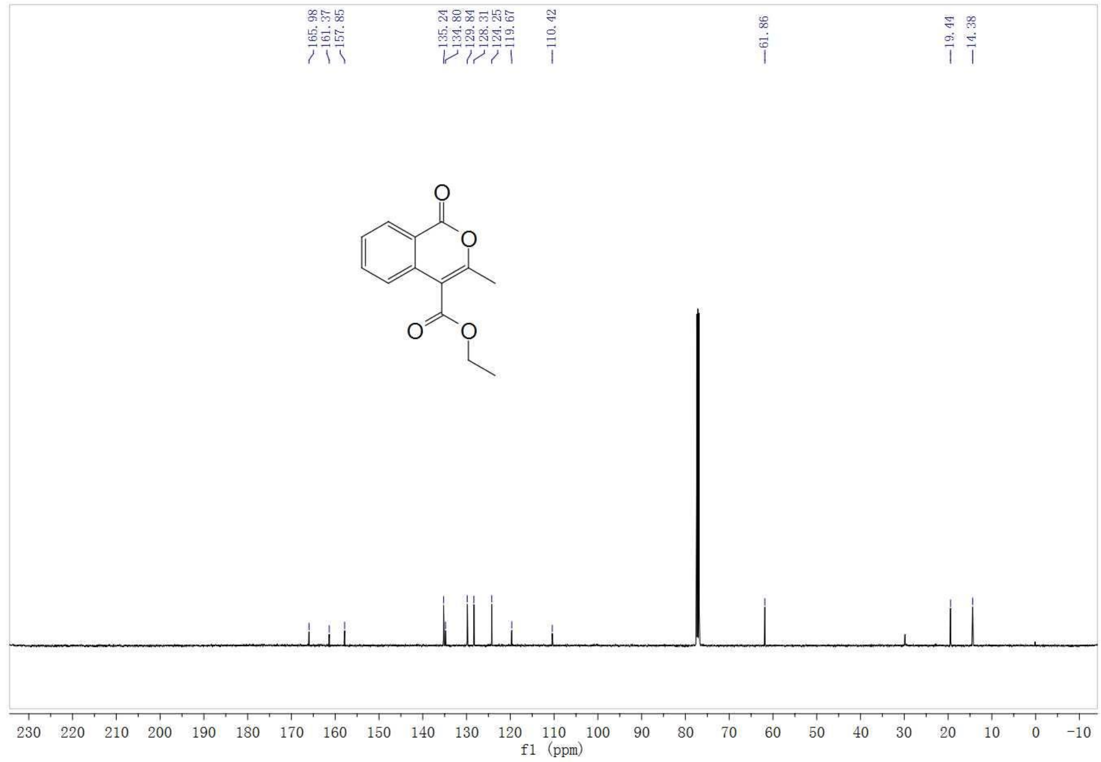

Ethyl 3,6-dimethyl-1-oxo-1*H*-isochromene-4-carboxylate (3ba).

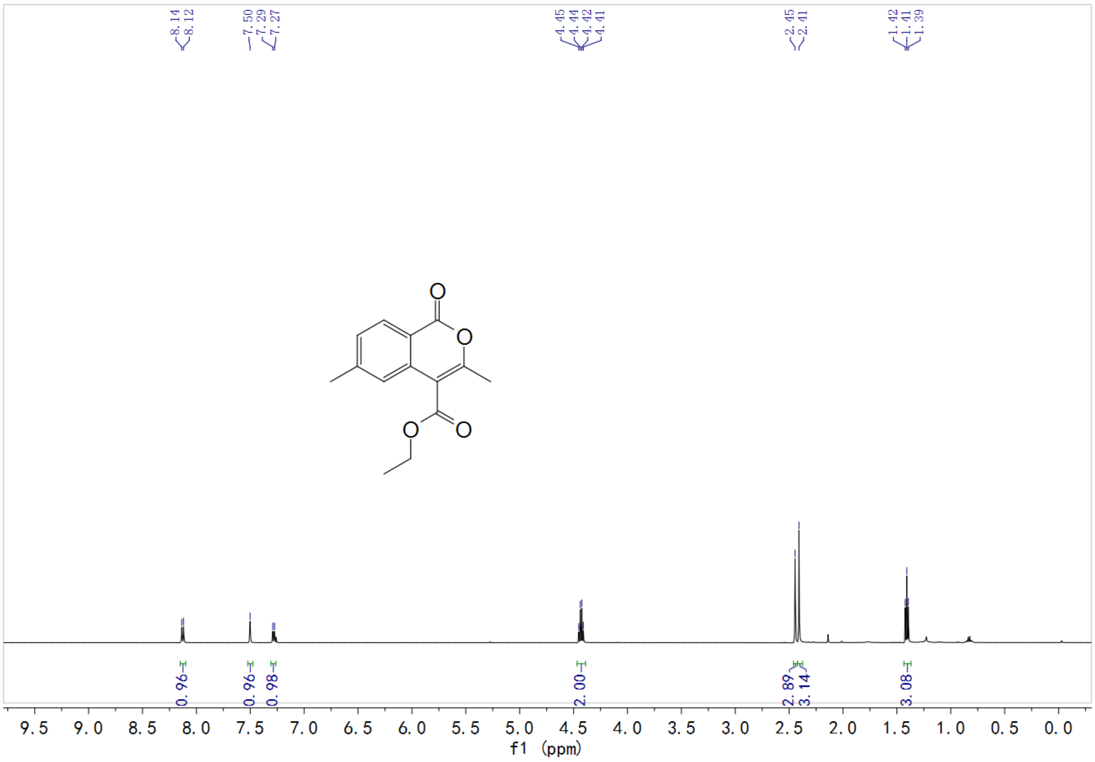

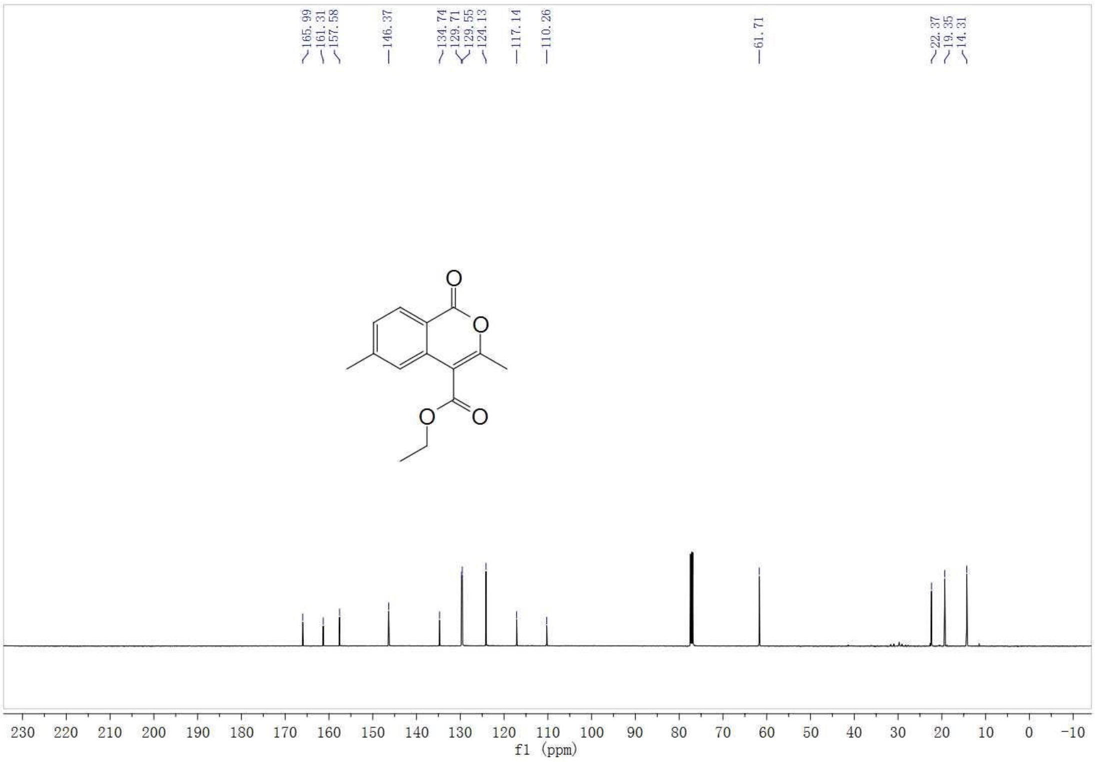

Ethyl 6-ethyl-3-methyl-1-oxo-1H-isochromene-4-carboxylate (3ca).

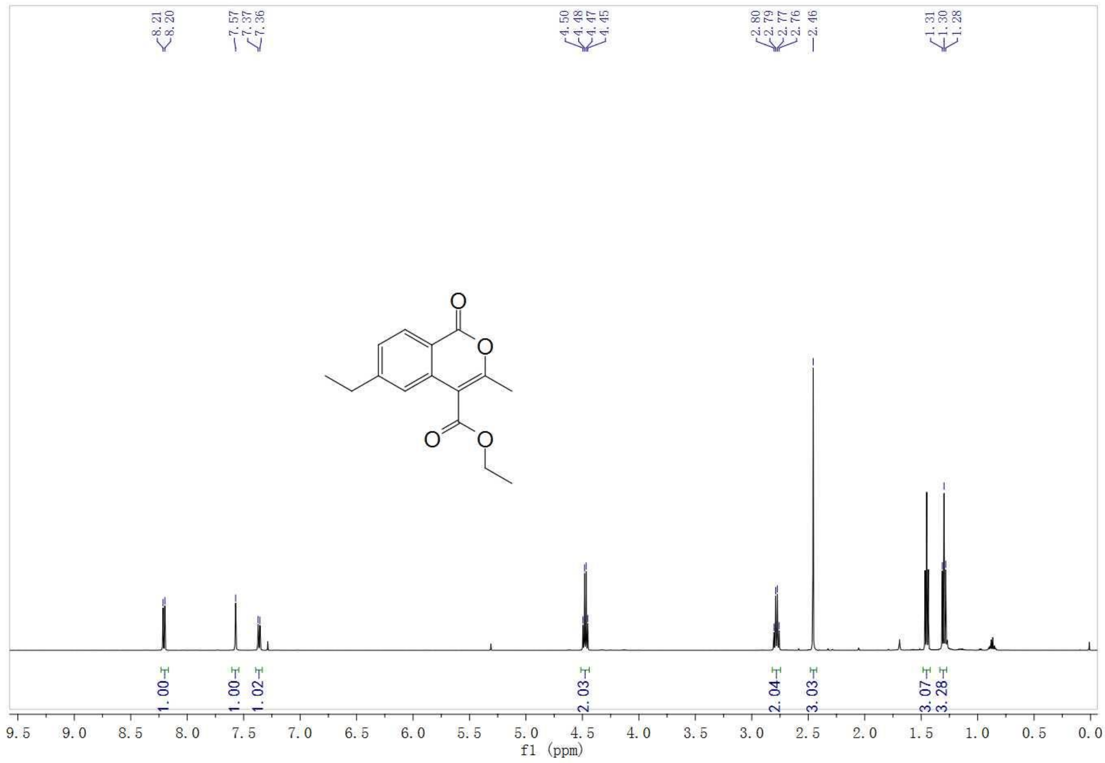

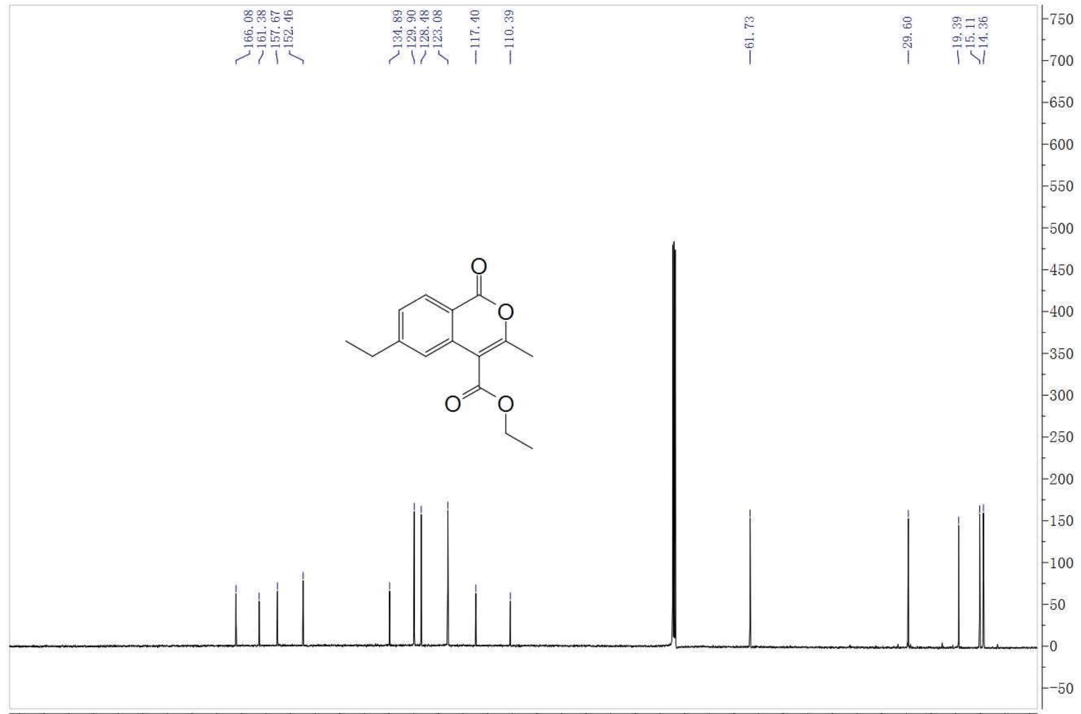

Ethyl 6-isopropyl-3-methyl-1-oxo-1*H*-isochromene-4-carboxylate(3da).

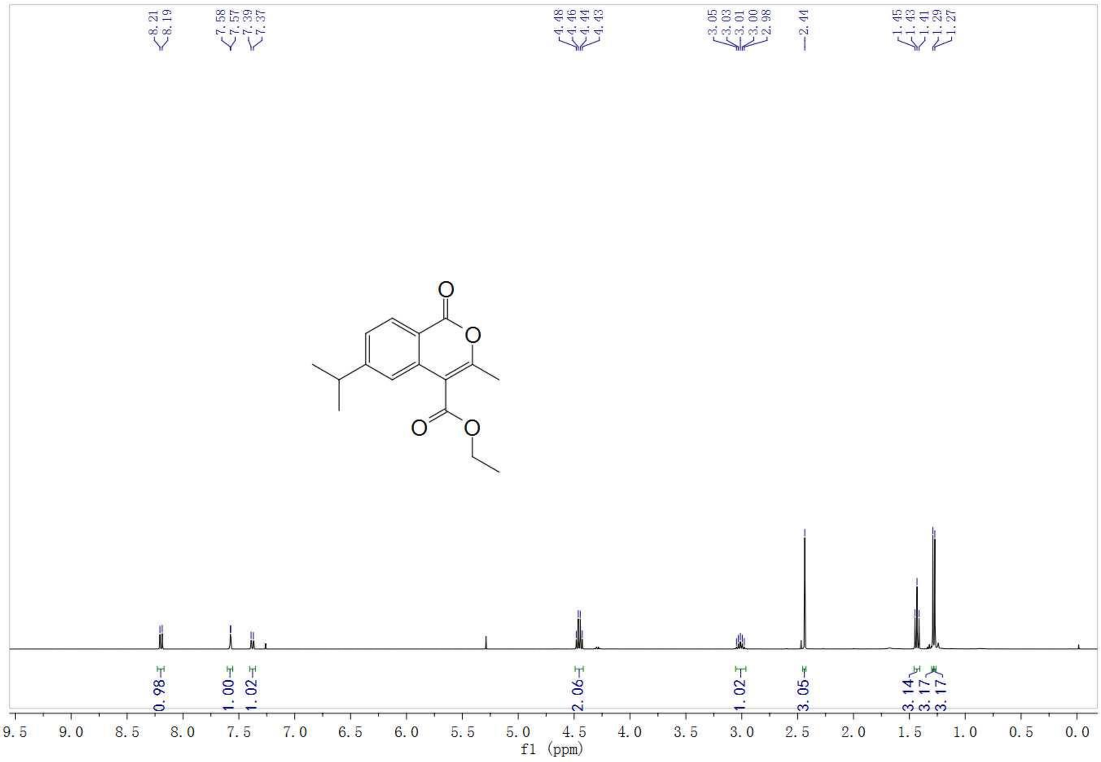

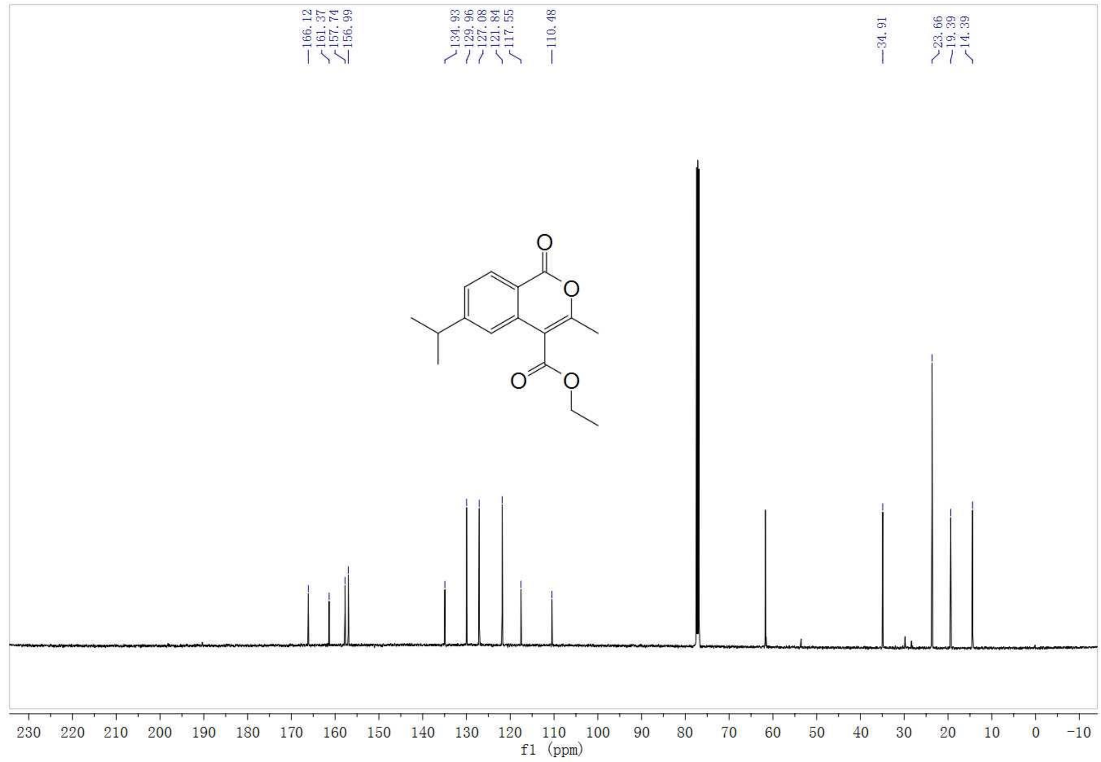

Ethyl 6-(*tert*-butyl)-3-methyl-1-oxo-1*H*-isochromene-4-carboxylate (3ea).

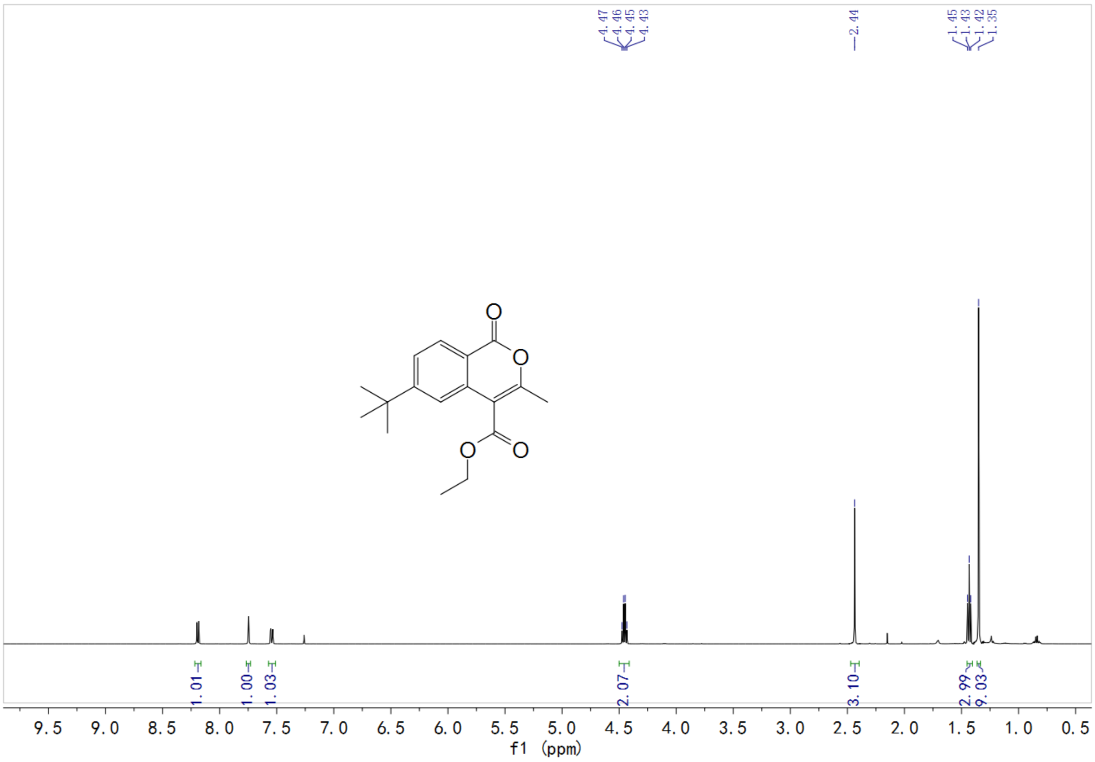

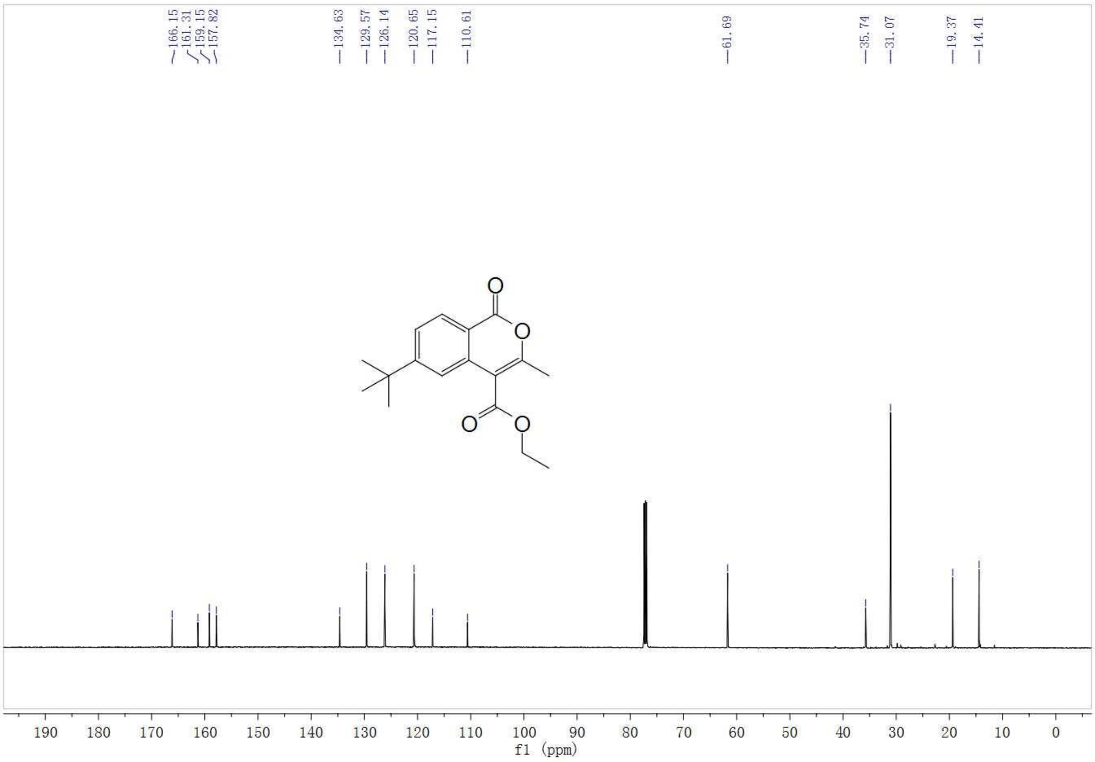

Ethyl 6-methoxy-3-methyl-1-oxo-1*H*-isochromene-4-carboxylate (3fa).

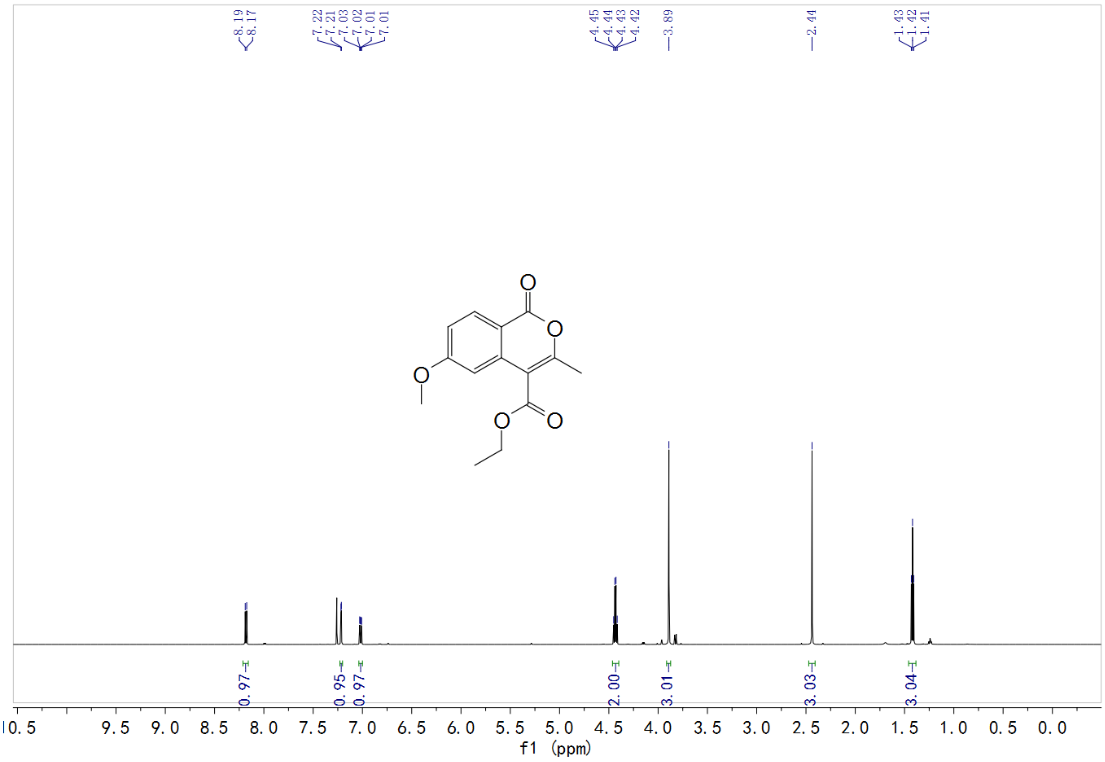

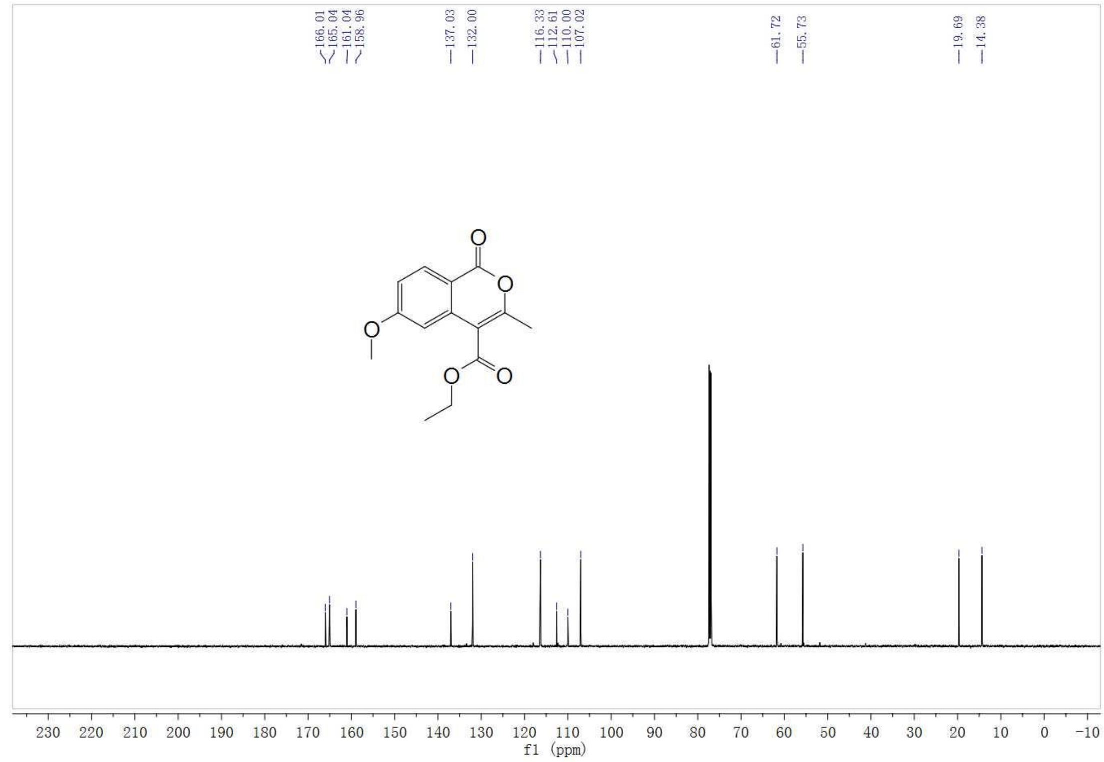

Ethyl 6-fluoro-3-methyl-1-oxo-1*H*-isochromene-4-carboxylate (3ga)

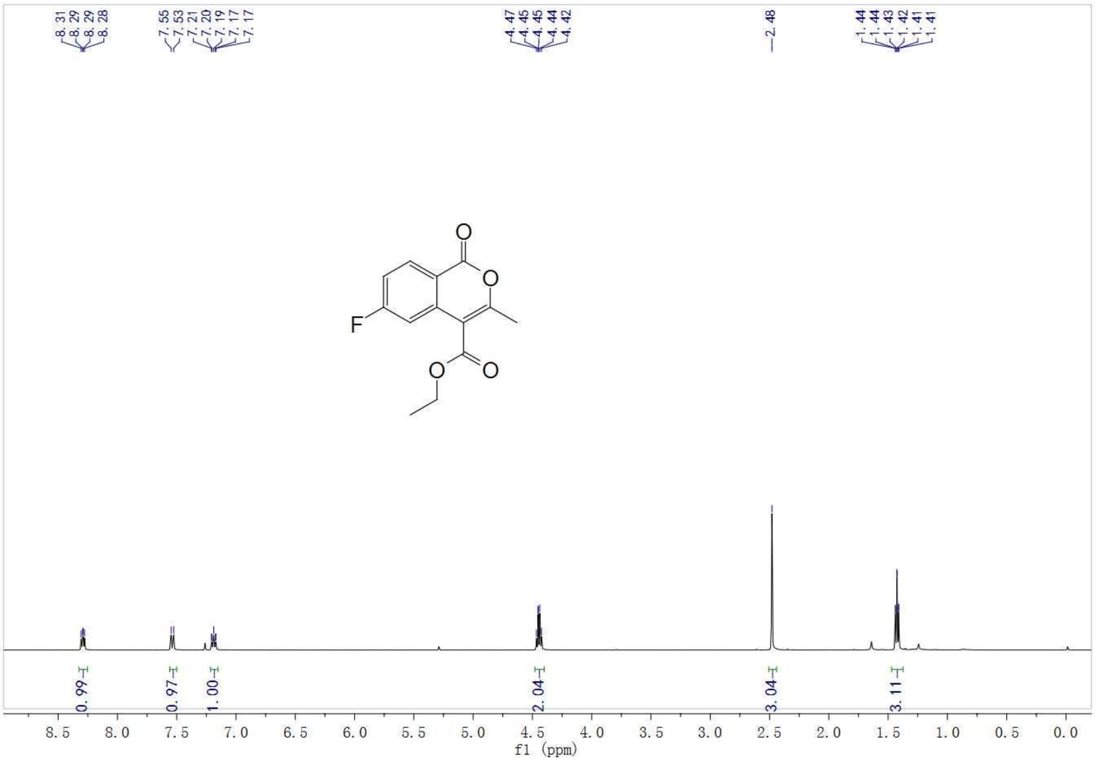

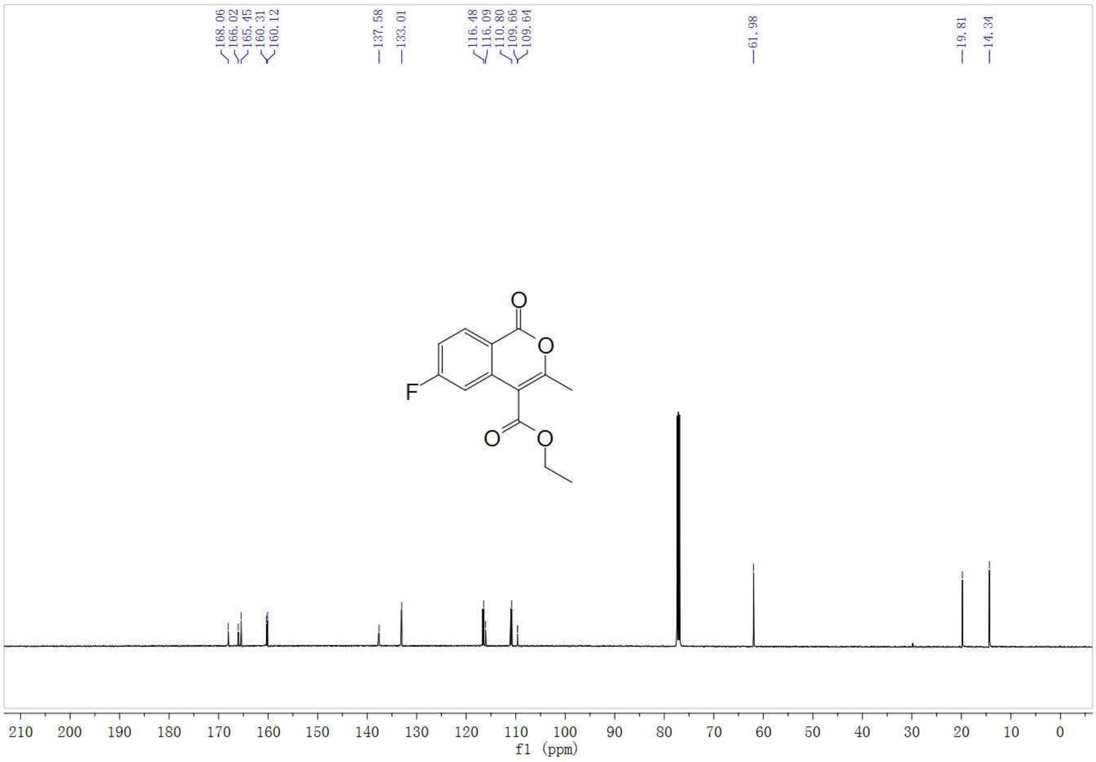

Ethyl 6-chloro-3-methyl-1-oxo-1*H*-isochromene-4-carboxylate (3ha).

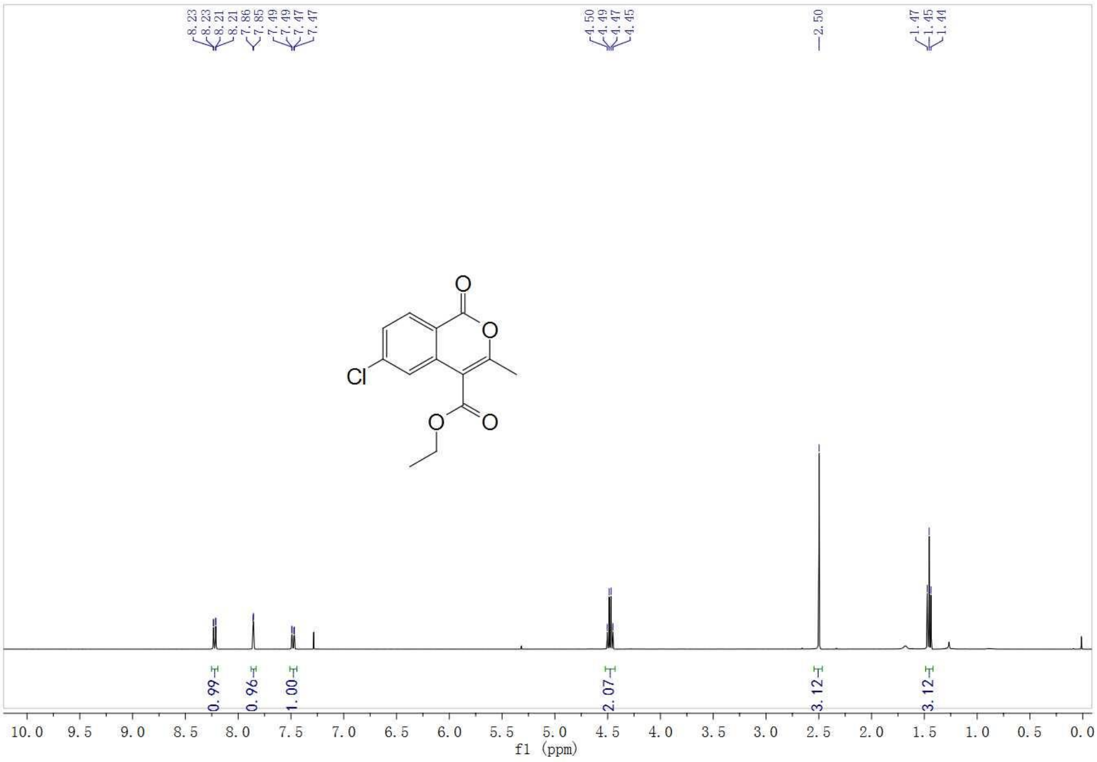

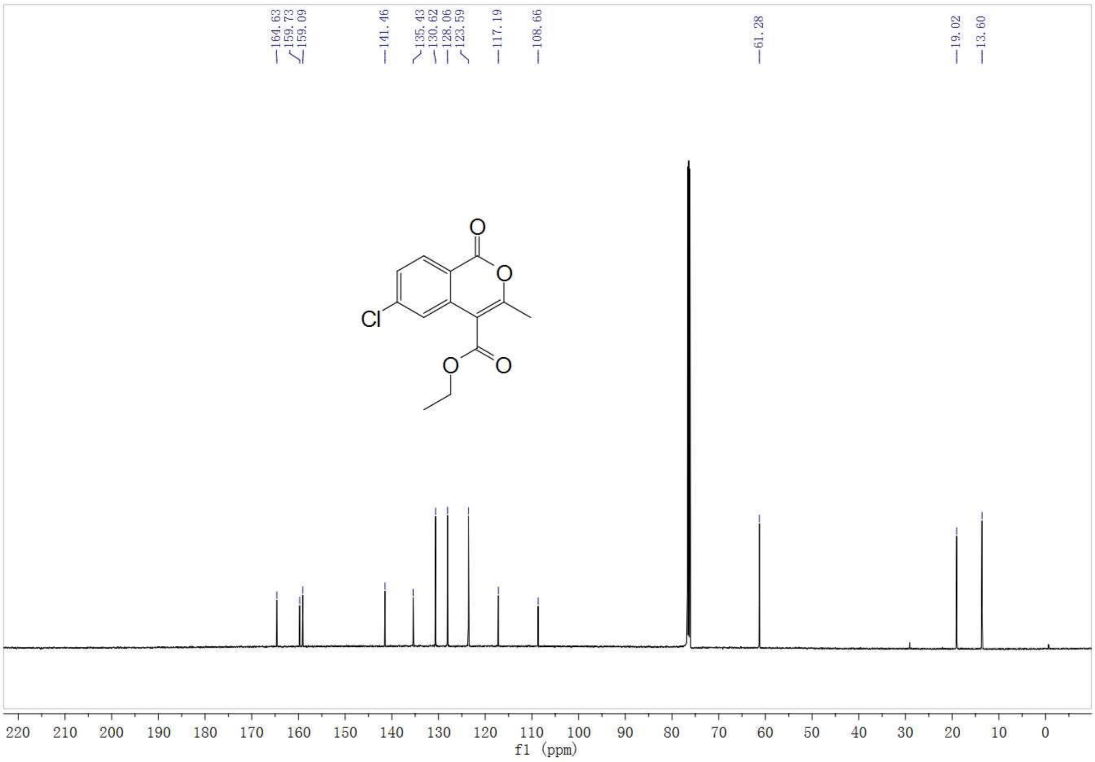

Ethyl 6-bromo-3-methyl-1-oxo-1*H*-isochromene-4-carboxylate (3ia).

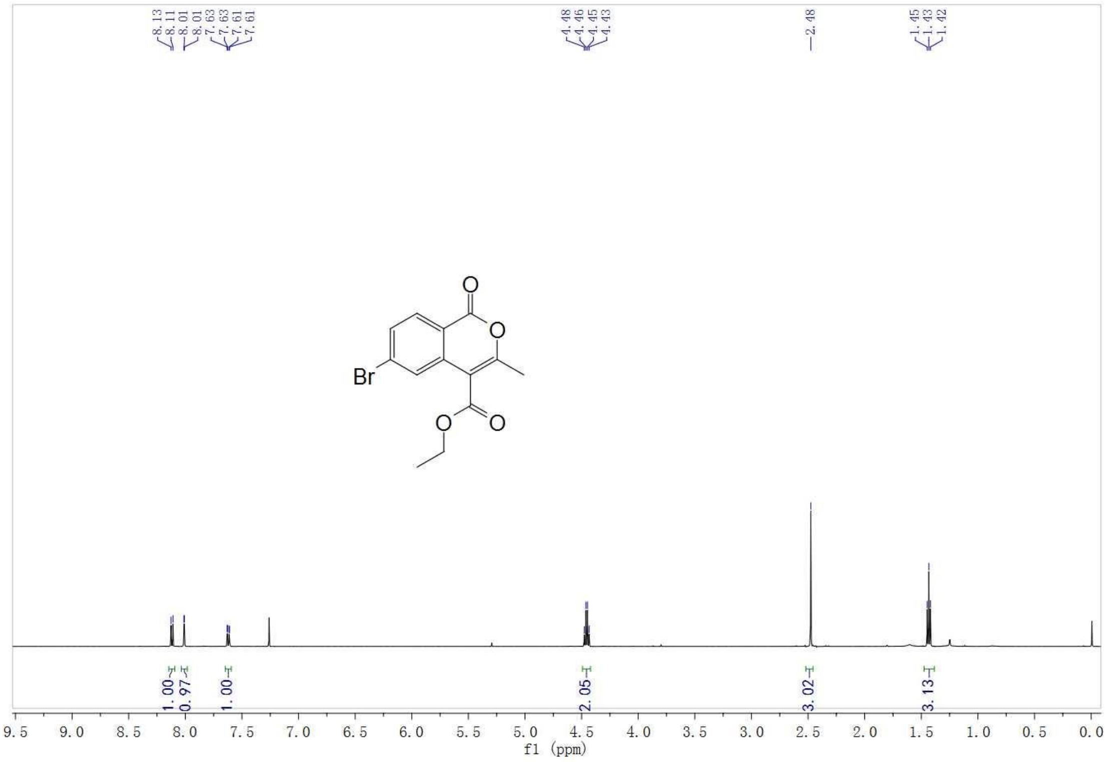

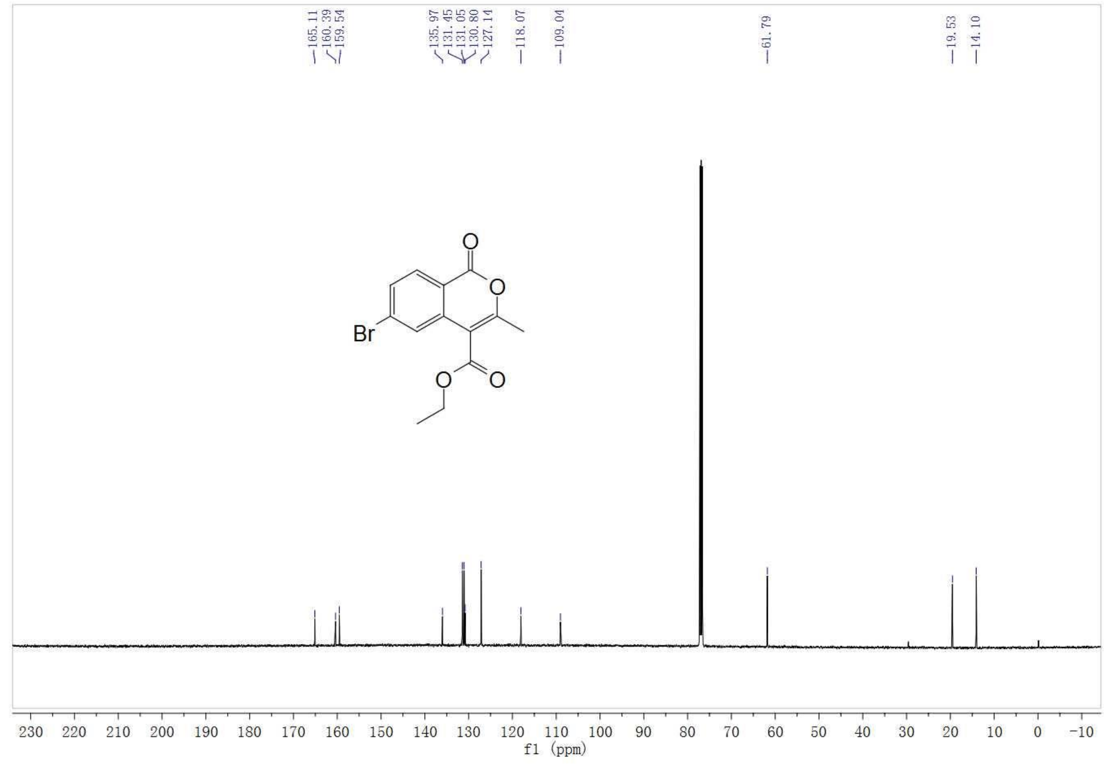

Ethyl 6-iodo-3-methyl-1-oxo-1*H*-isochromene-4-carboxylate (3ja).

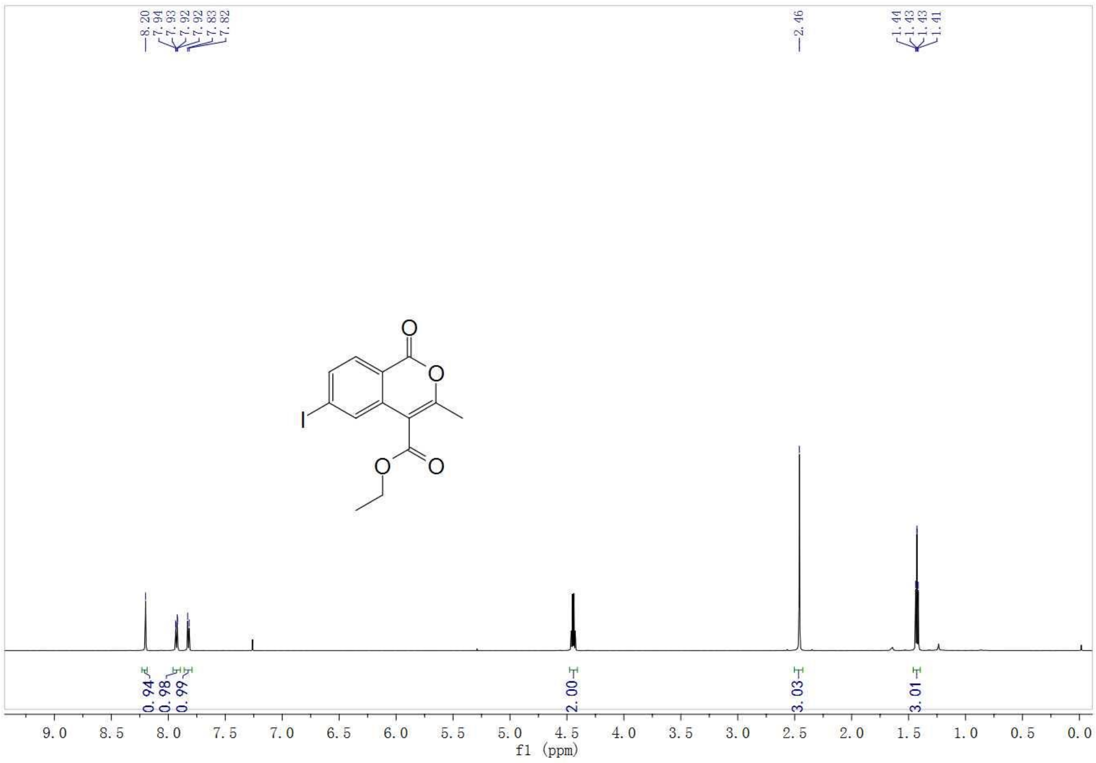

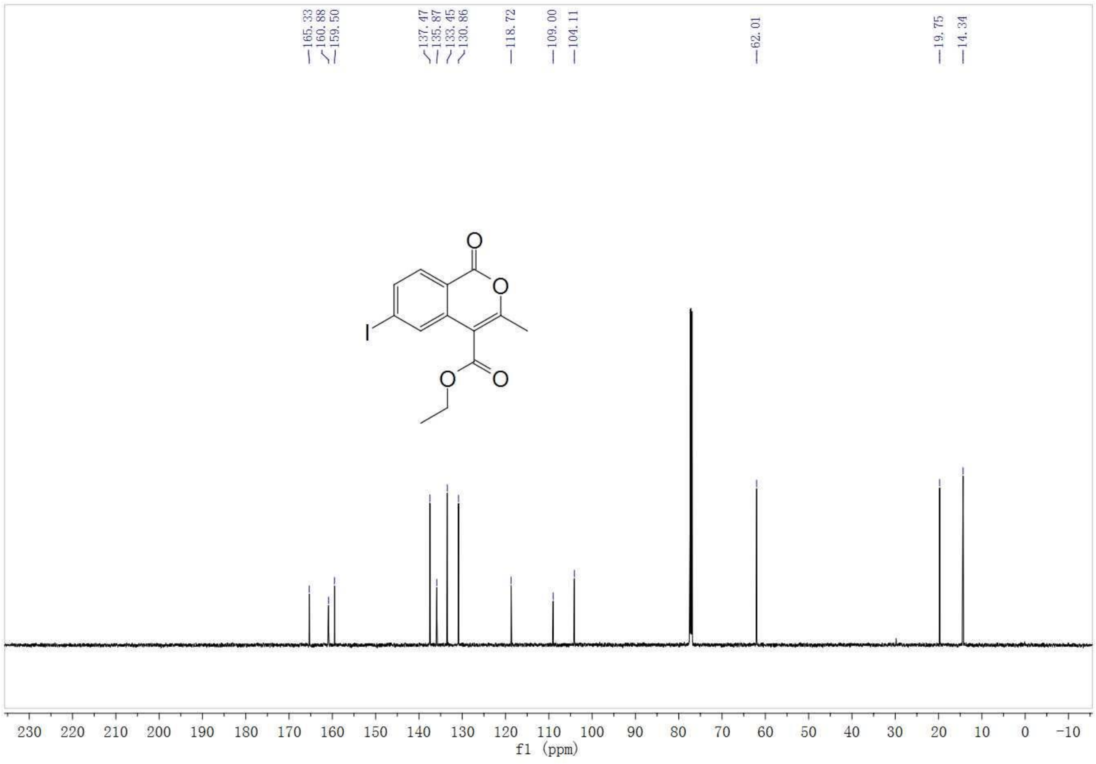

Ethyl 3-methyl-1-oxo-6-(trifluoromethyl)-1H-isochromene-4-carboxylate (3ka).

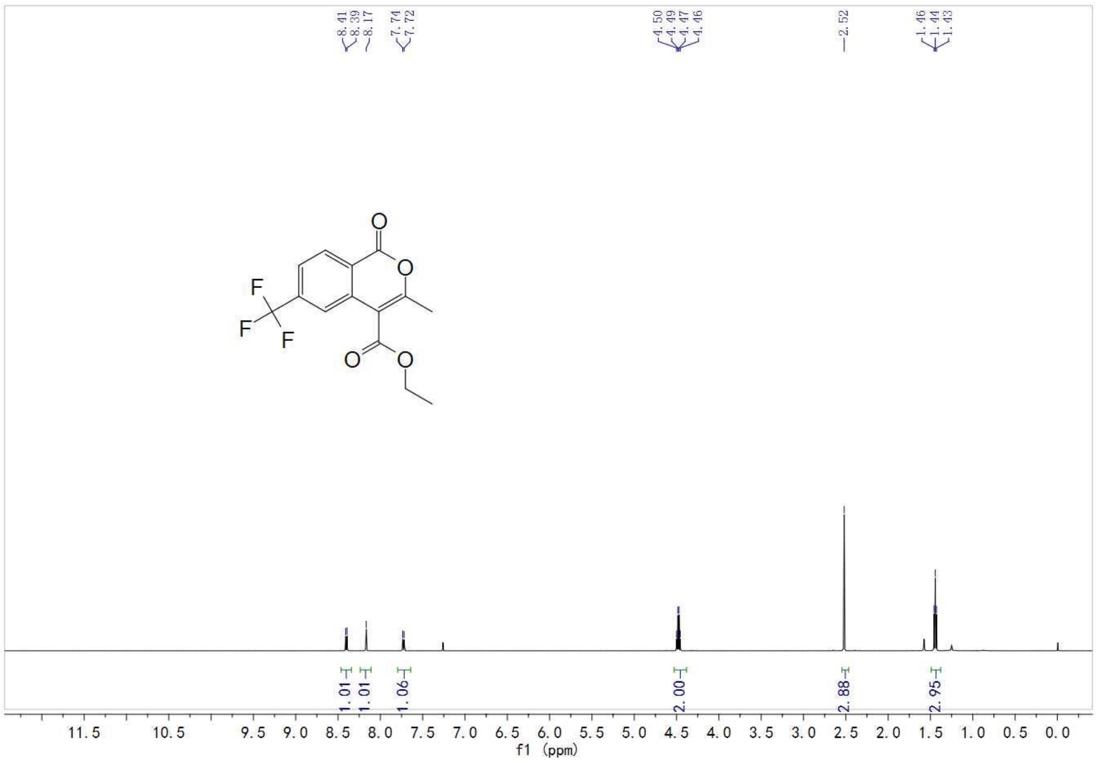

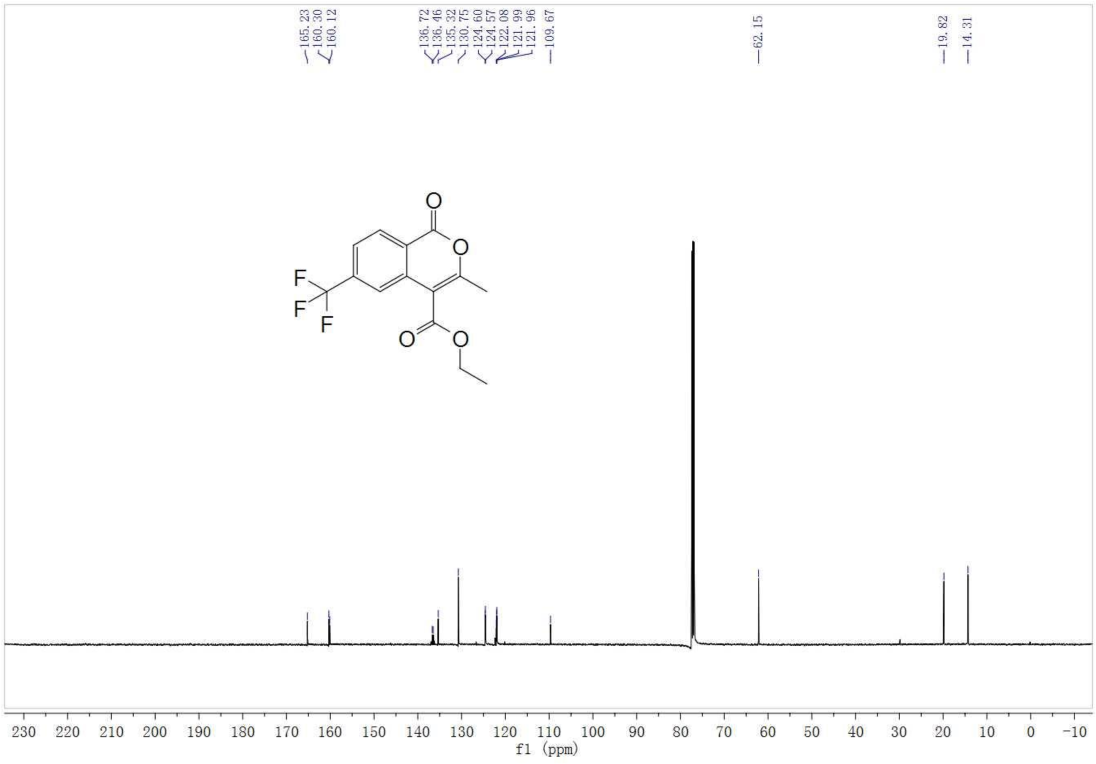

Ethyl 6-(benzyloxy)-3-methyl-1-oxo-1H-isochromene-4-carboxylate (3ma).

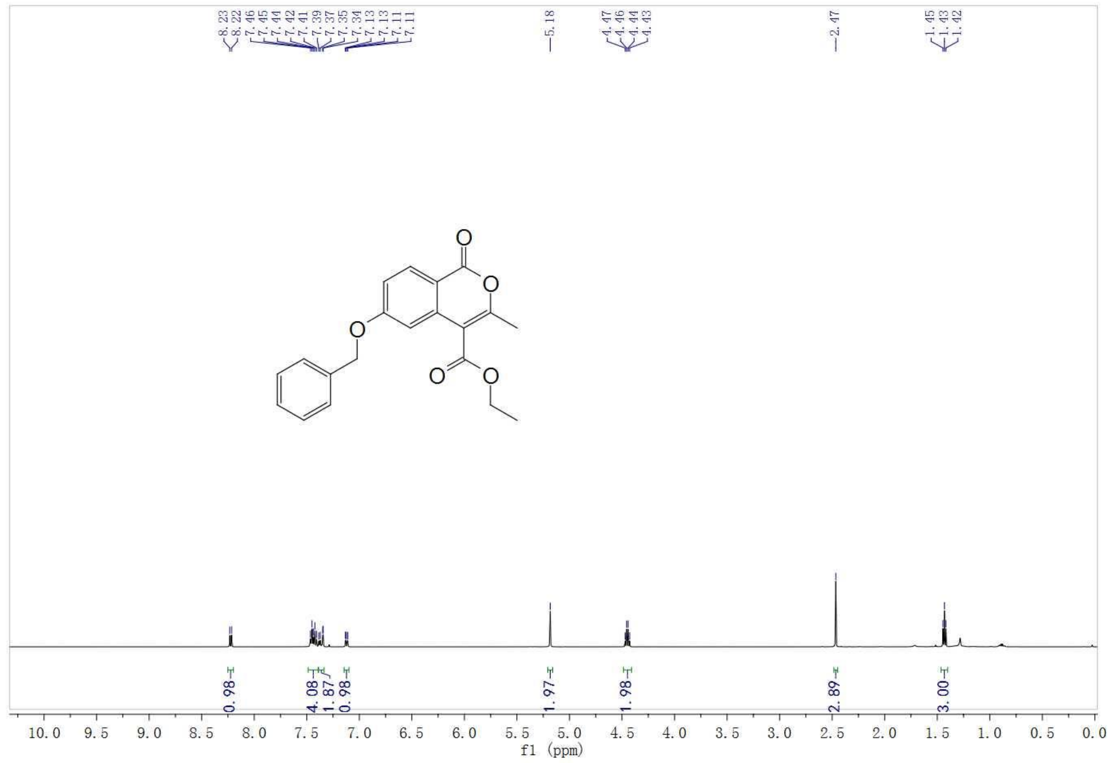

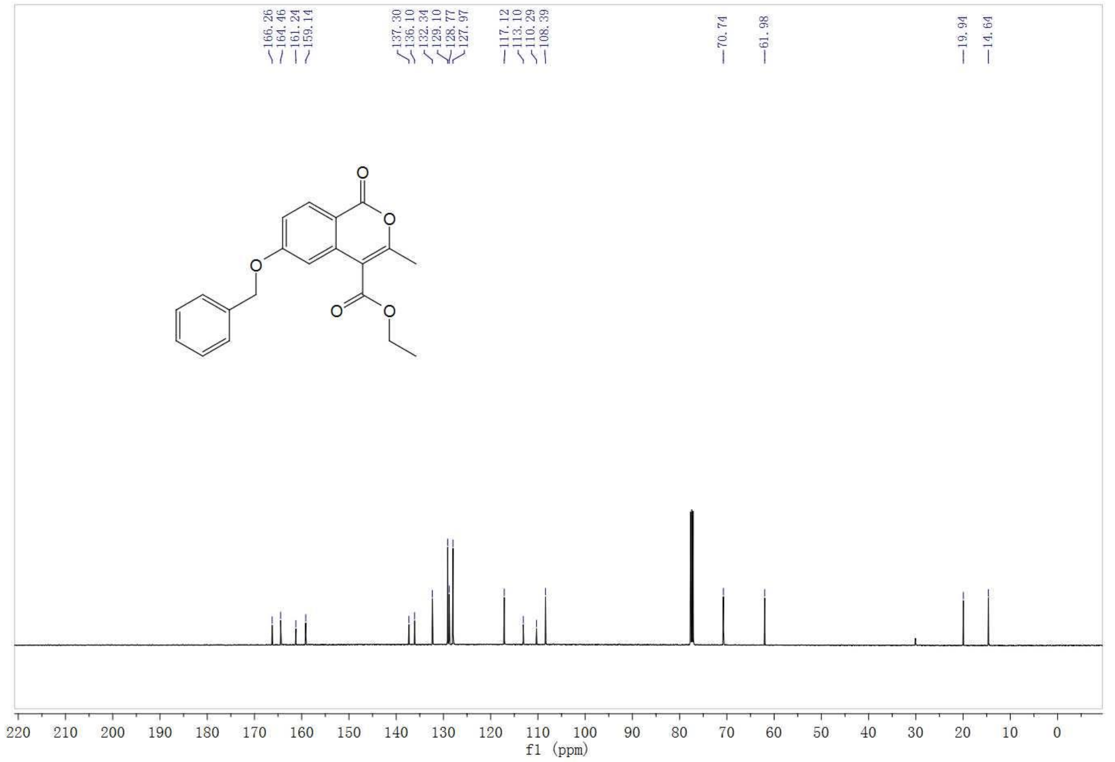

Ethyl 6-(dimethylamino)-3-methyl-1-oxo-1*H*-isochromene-4-carboxylate (3na).

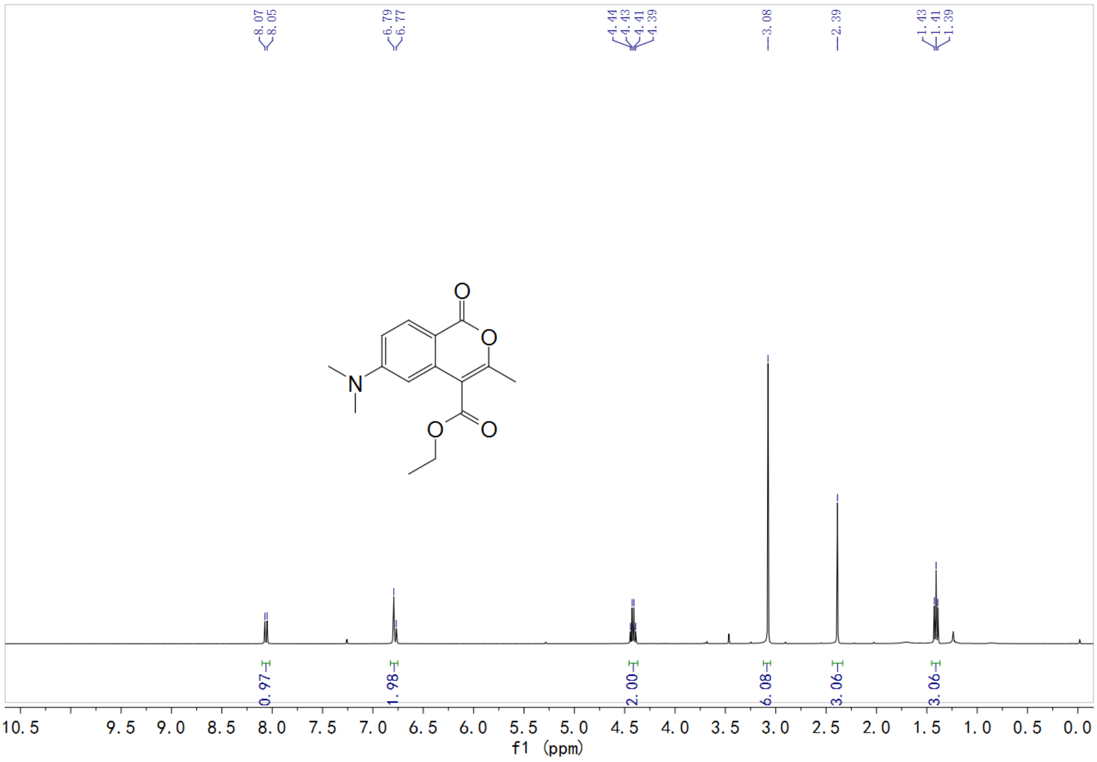

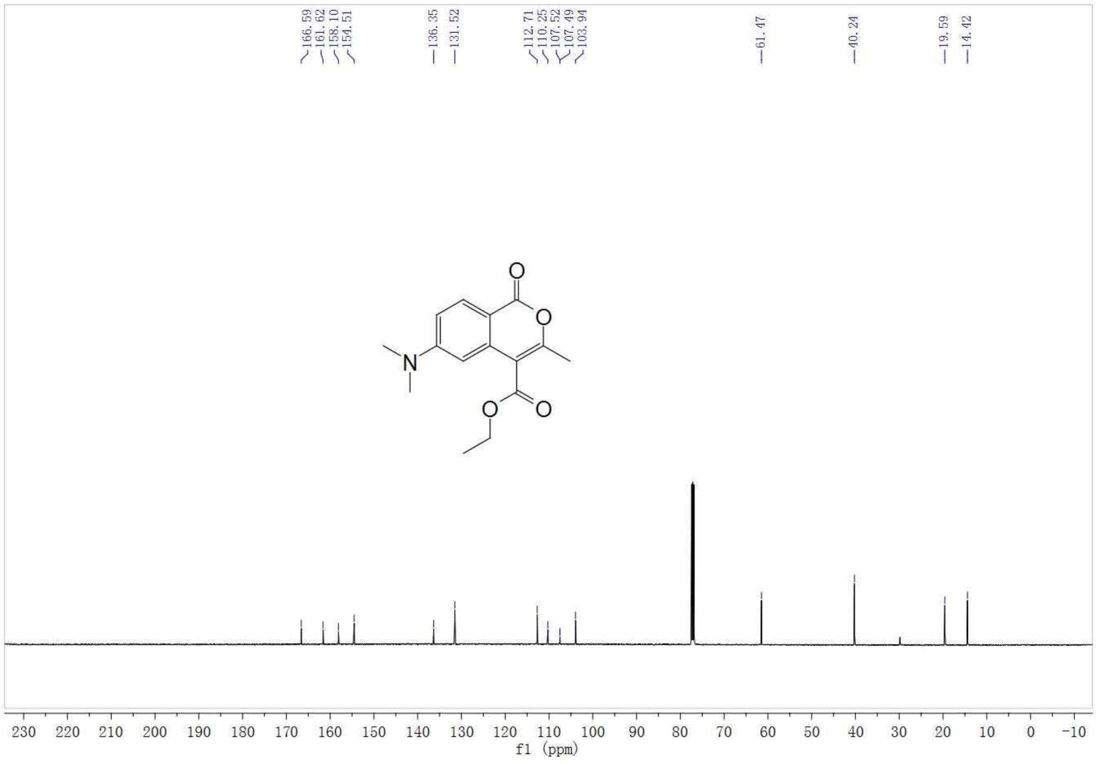

Ethyl 3-methyl-1-oxo-6-phenyl-1*H*-isochromene-4-carboxylate (3oa).

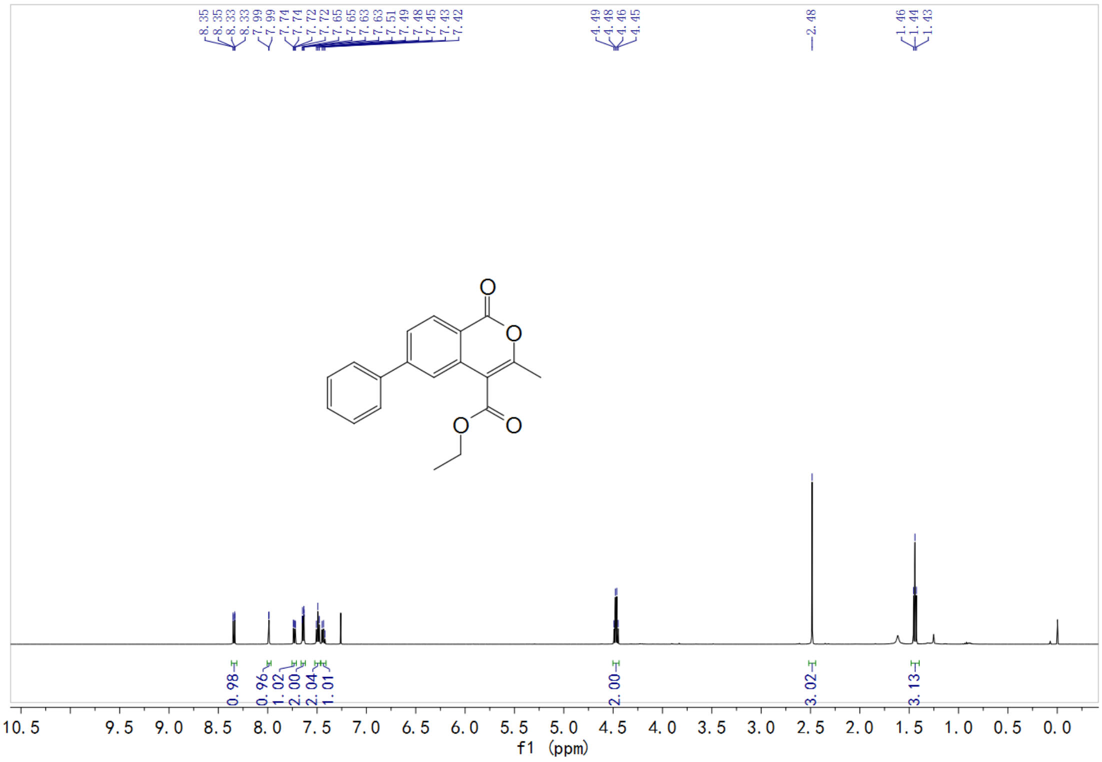

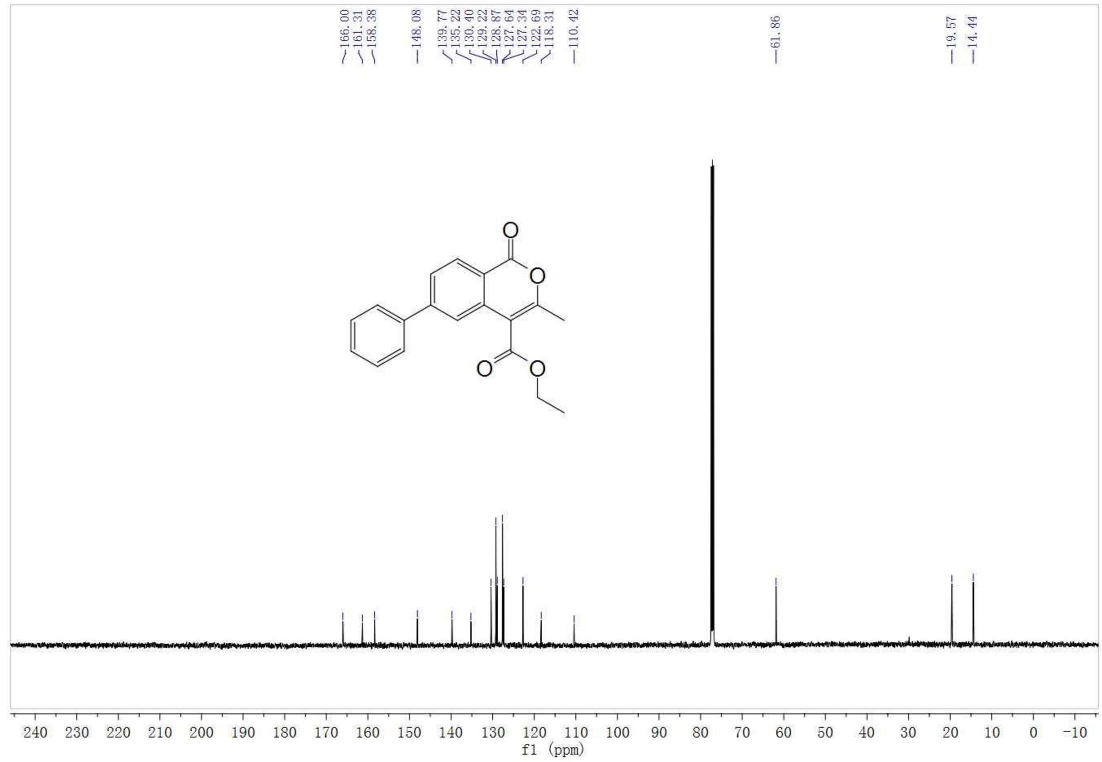

Ethyl 3,7-dimethyl-1-oxo-1*H*-isochromene-4-carboxylate (3pa).

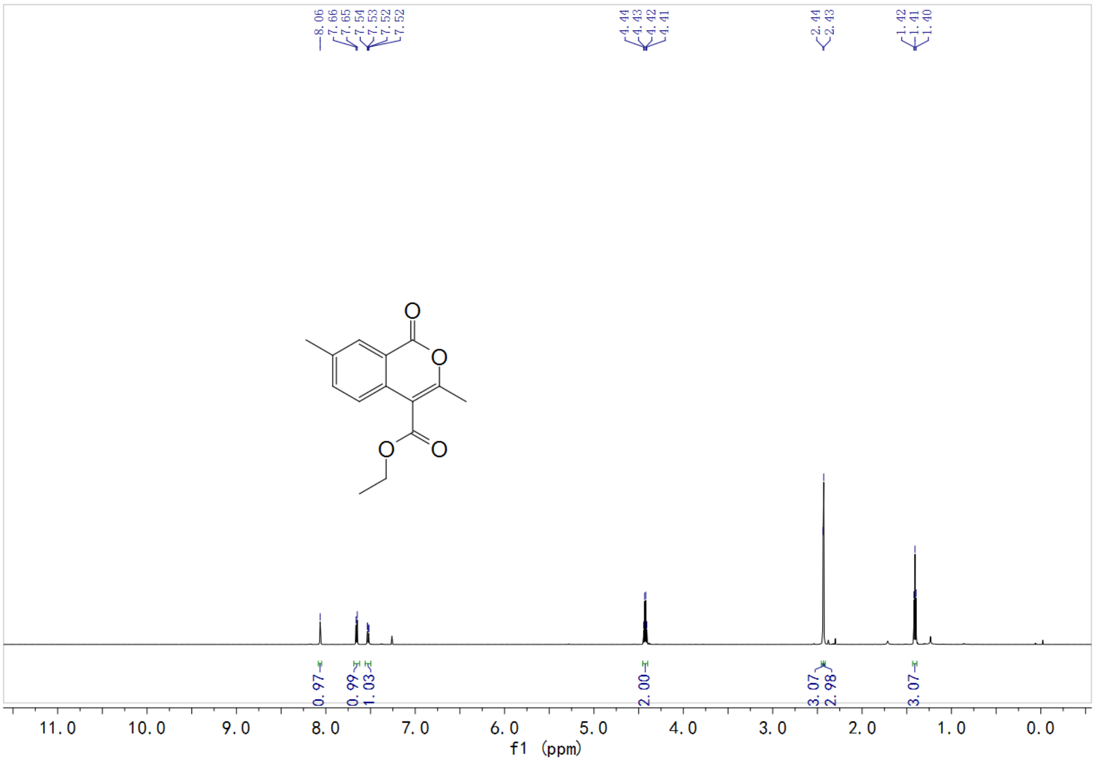

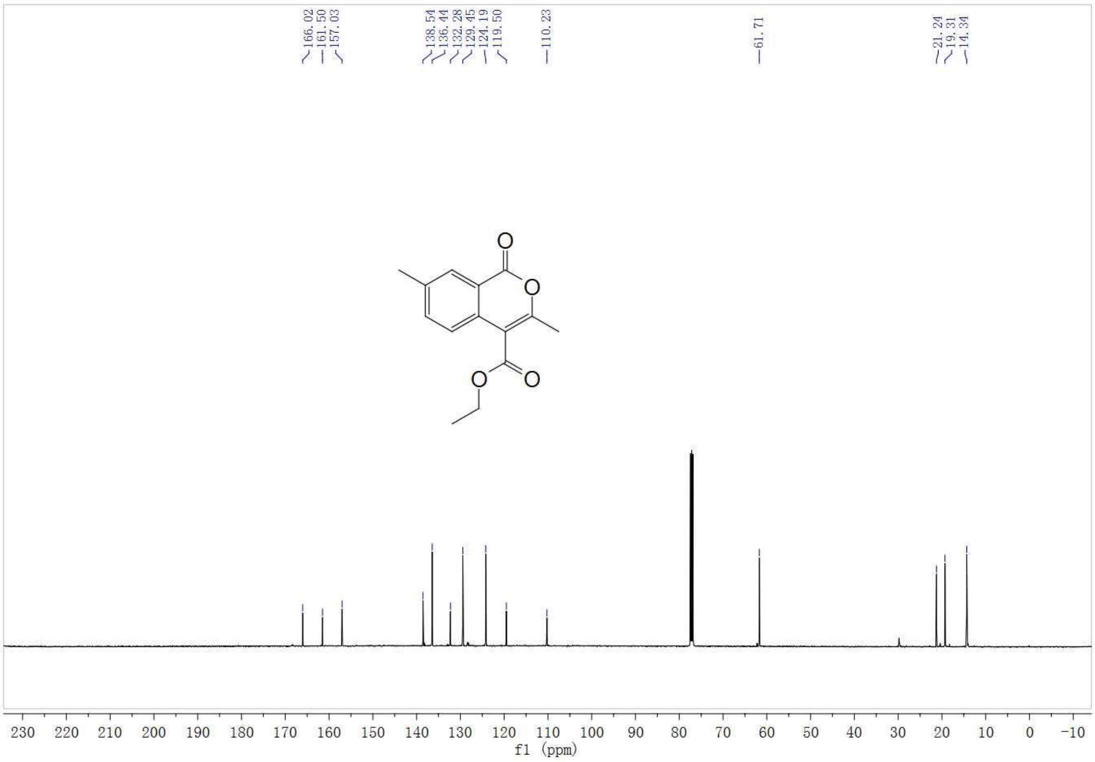

Ethyl 7-chloro-3-methyl-1-oxo-1*H*-isochromene-4-carboxylate (3qa).

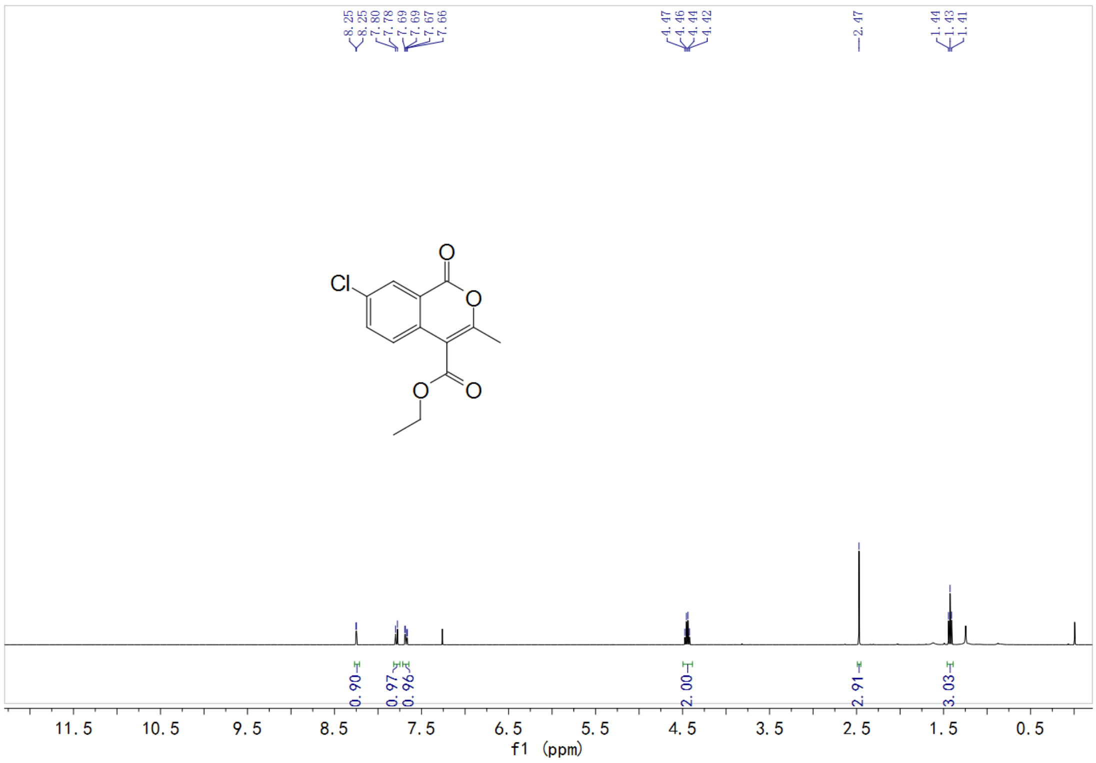

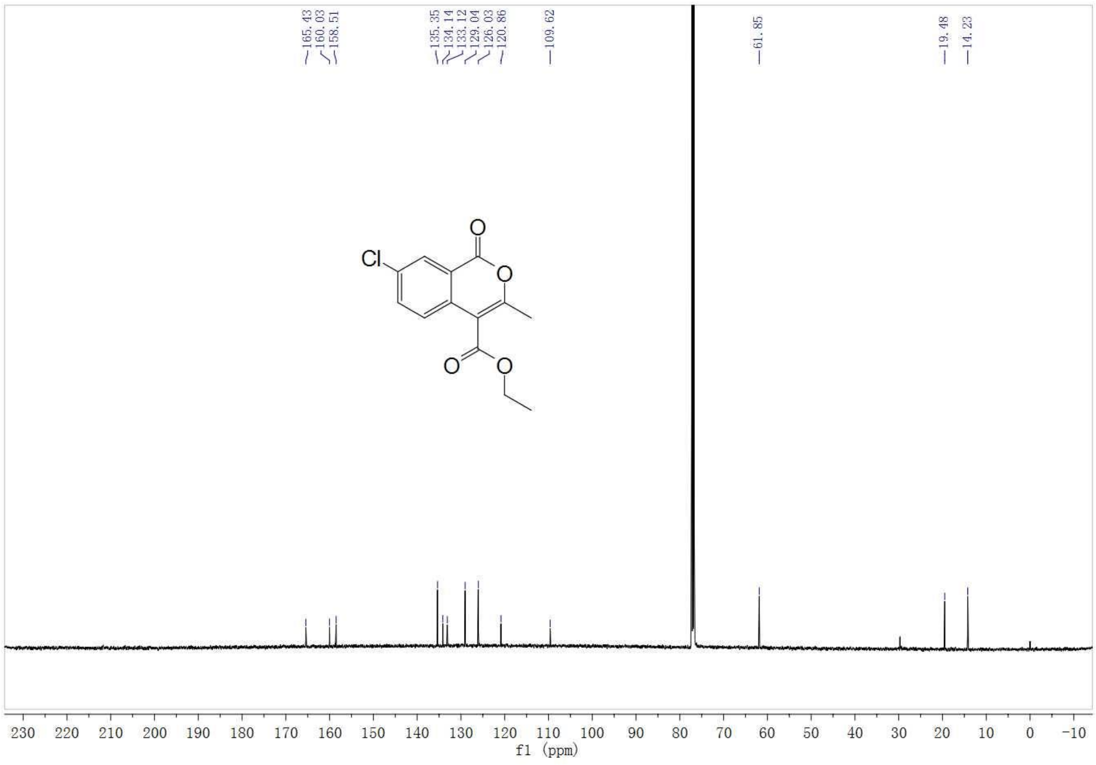

Ethyl 3,5-dimethyl-1-oxo-1*H*-isochromene-4-carboxylate (3ra).

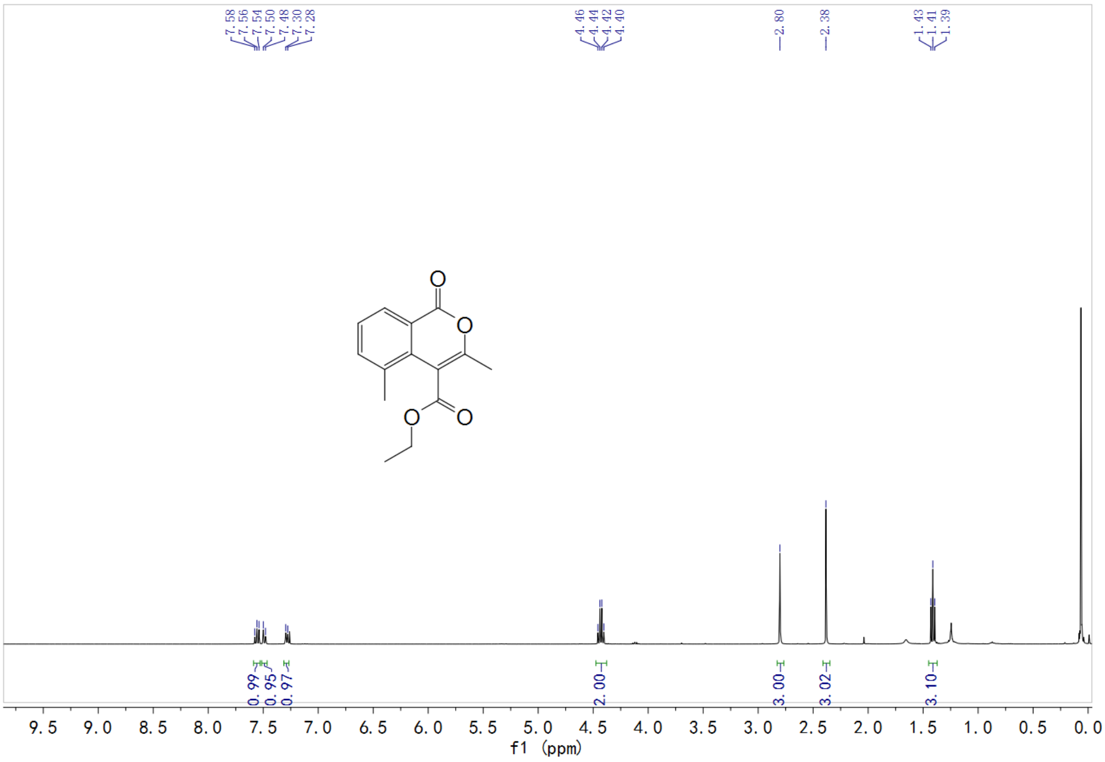

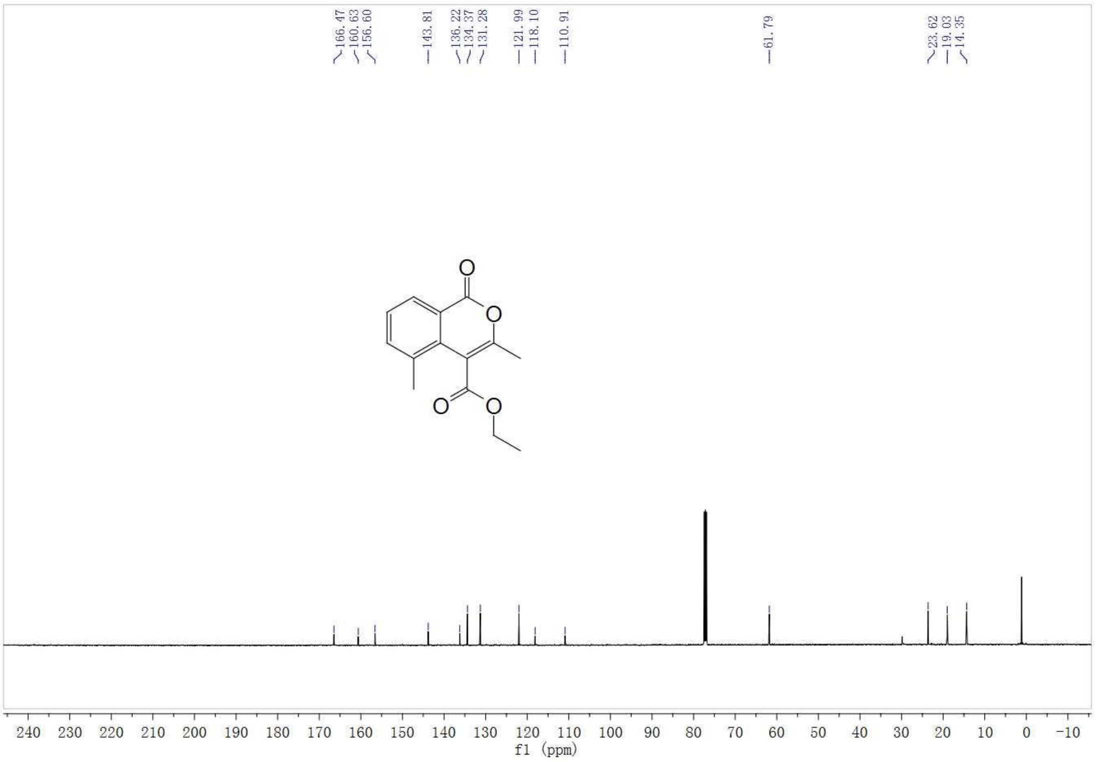

Ethyl 8-chloro-3-methyl-1-oxo-1*H*-isochromene-4-carboxylate (3sa).

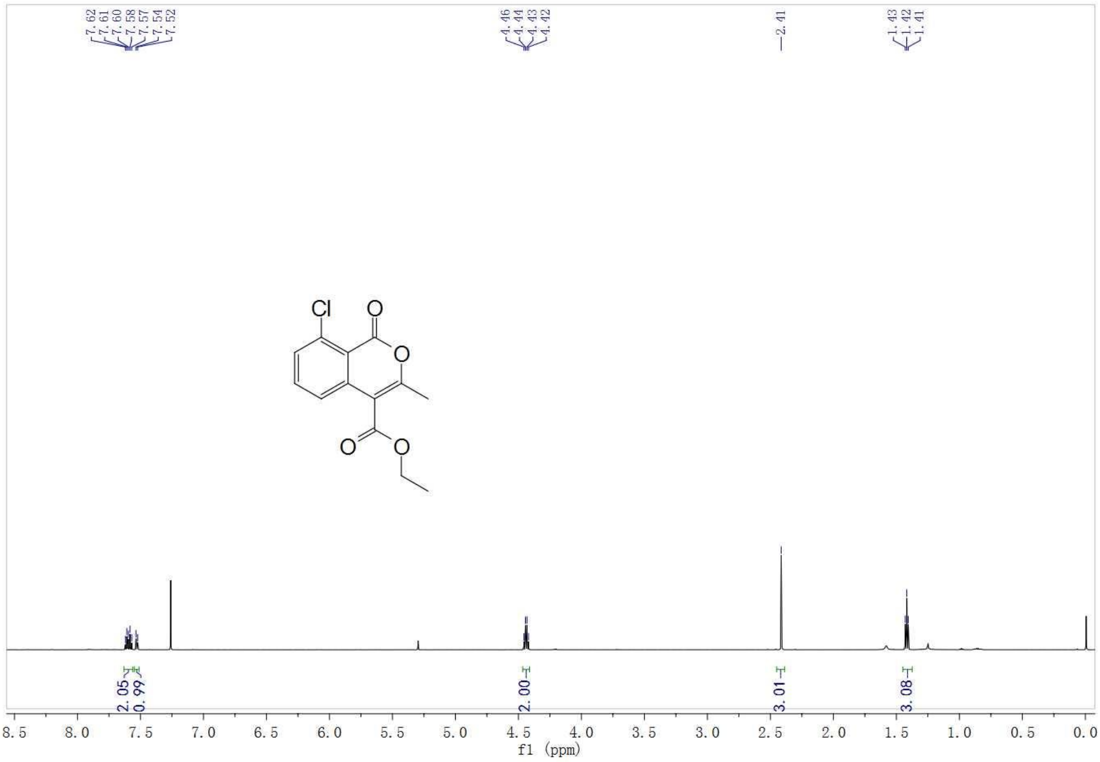

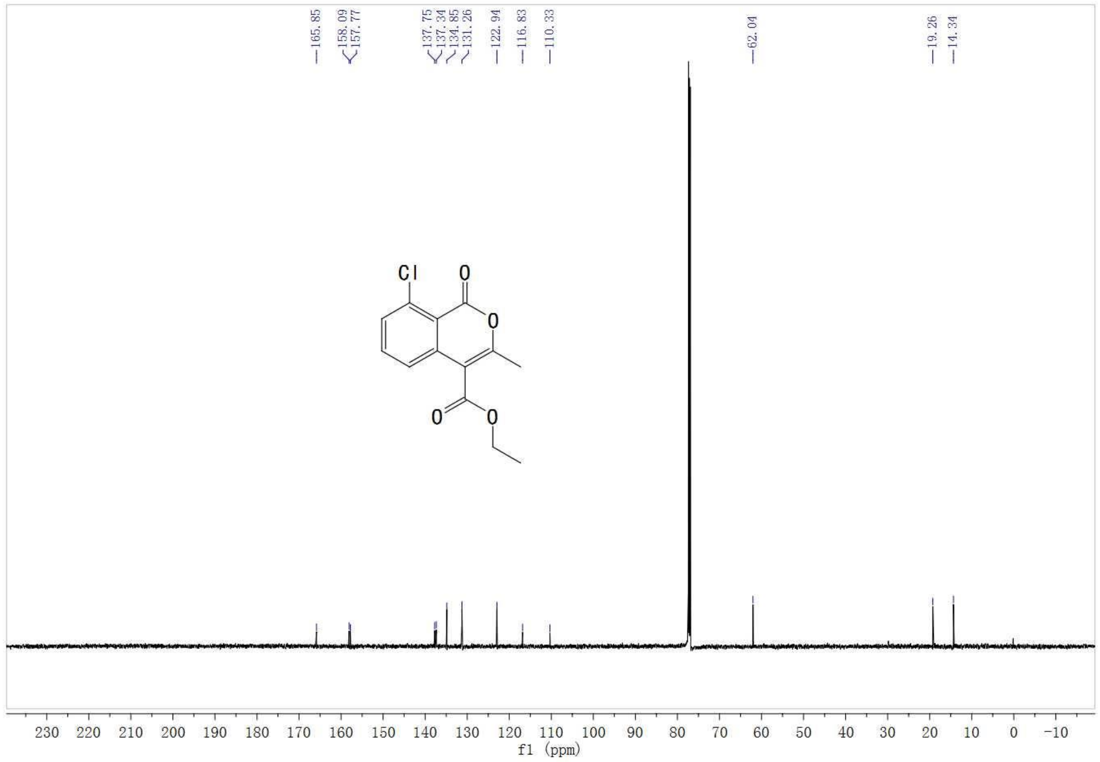

Ethyl 3,6,7-trimethyl-1-oxo-1*H*-isochromene-4-carboxylate (3ta).

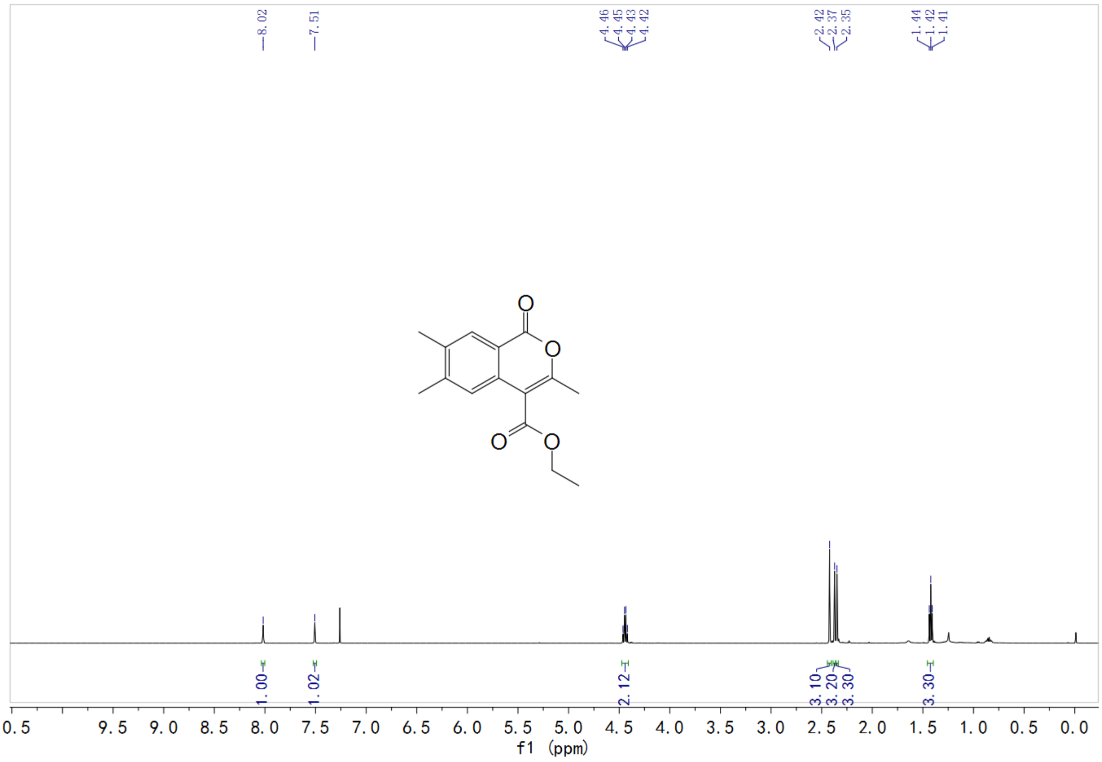

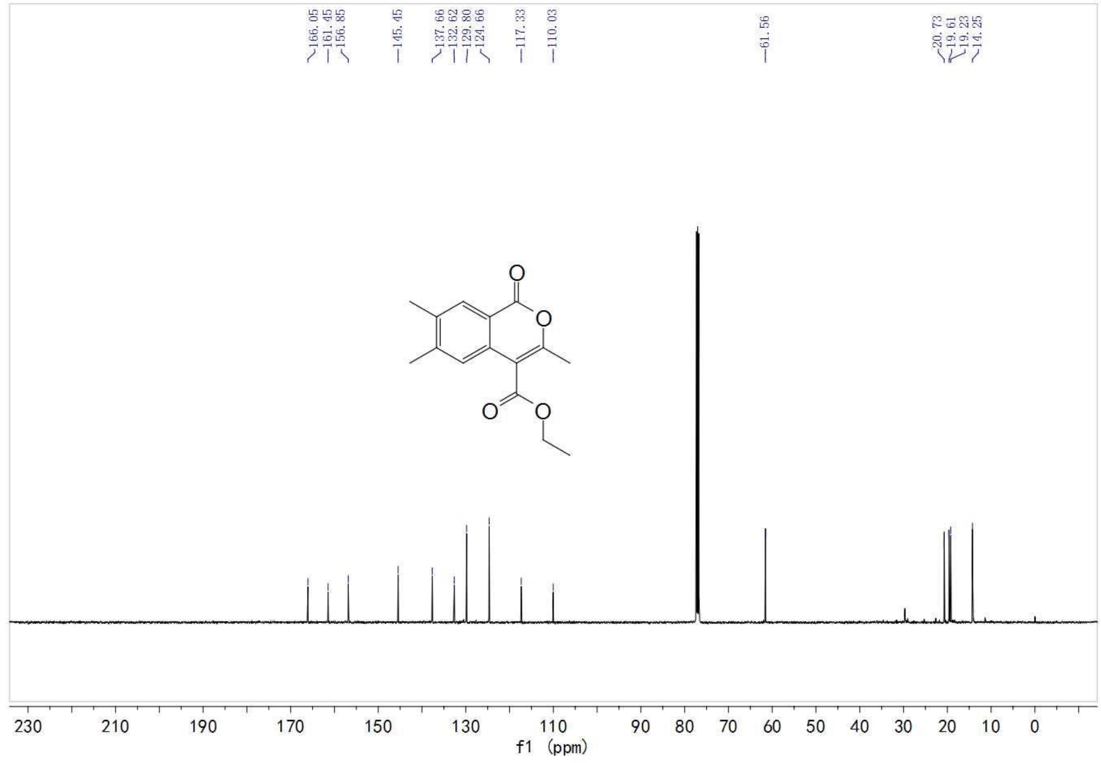

Ethyl 3-methyl-1-oxo-1*H*-benzo[*g*]isochromene-4-carboxylate (3ua).

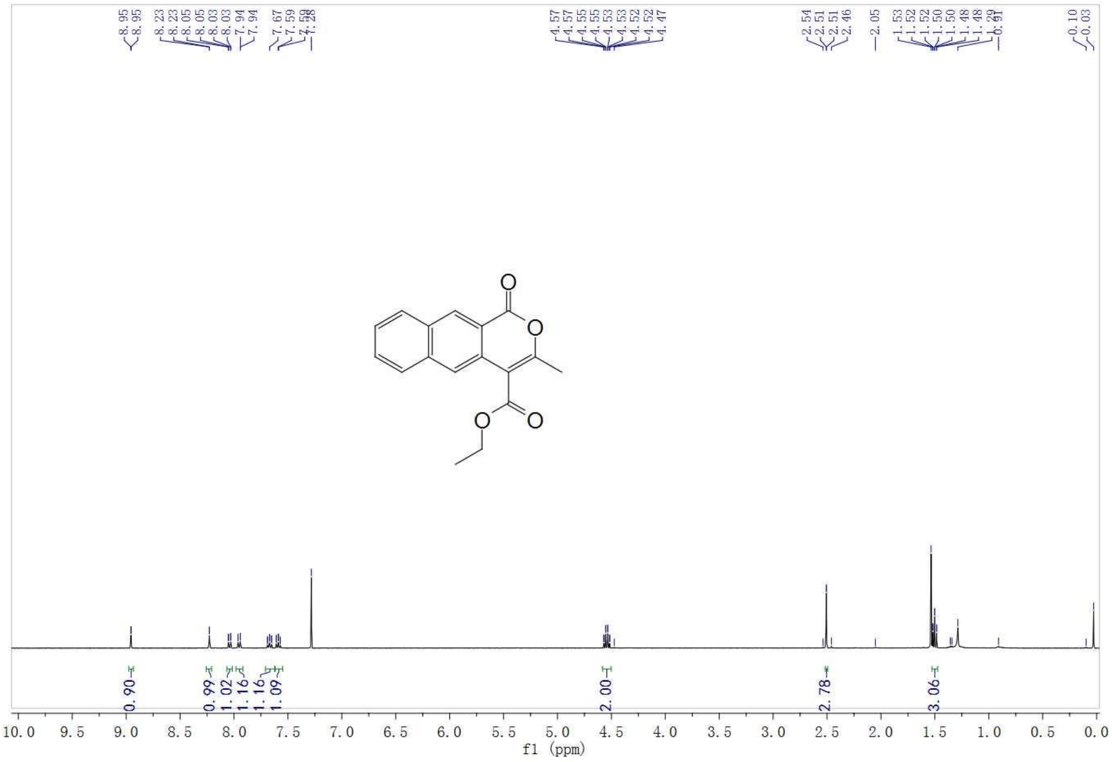

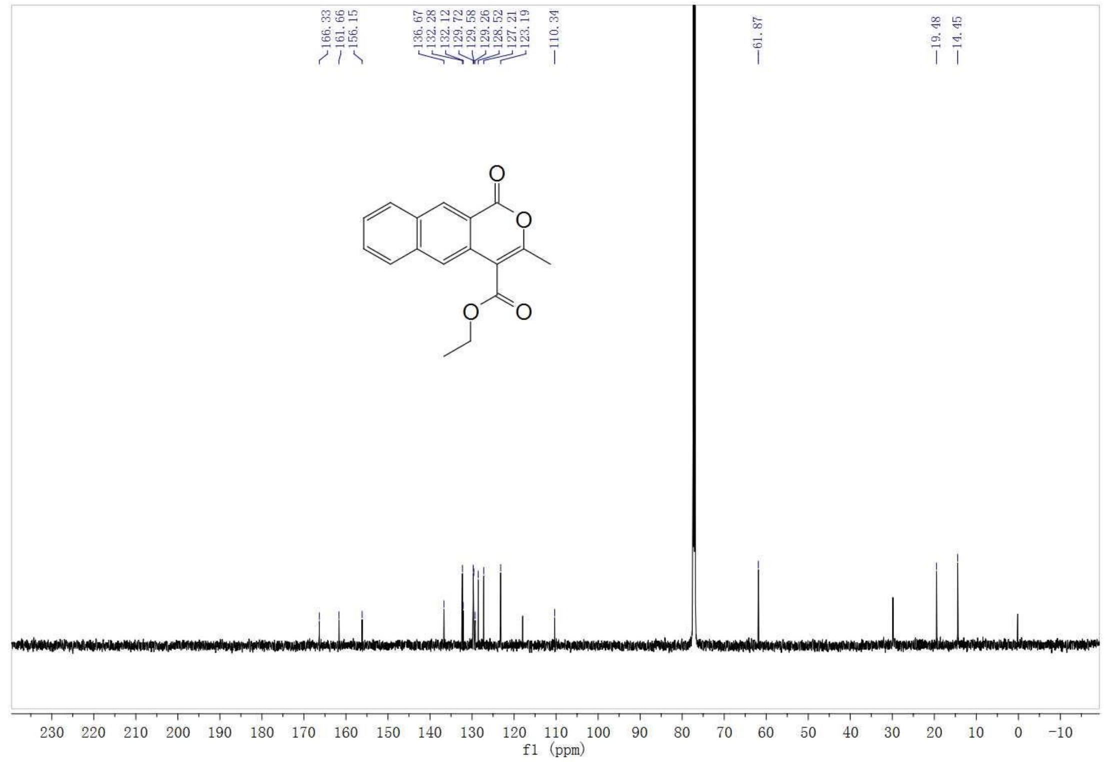

Ethyl 5-methyl-7-oxo-7*H*-thieno[2,3-c]pyran-4-carboxylate (3vq).

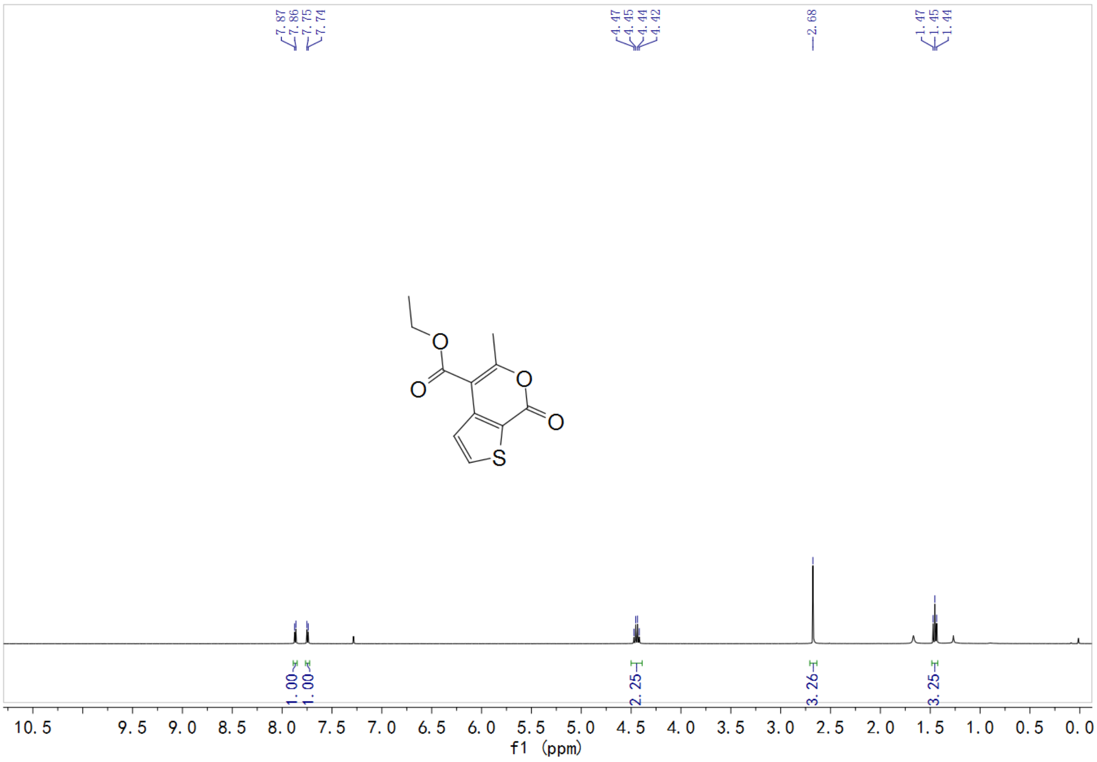

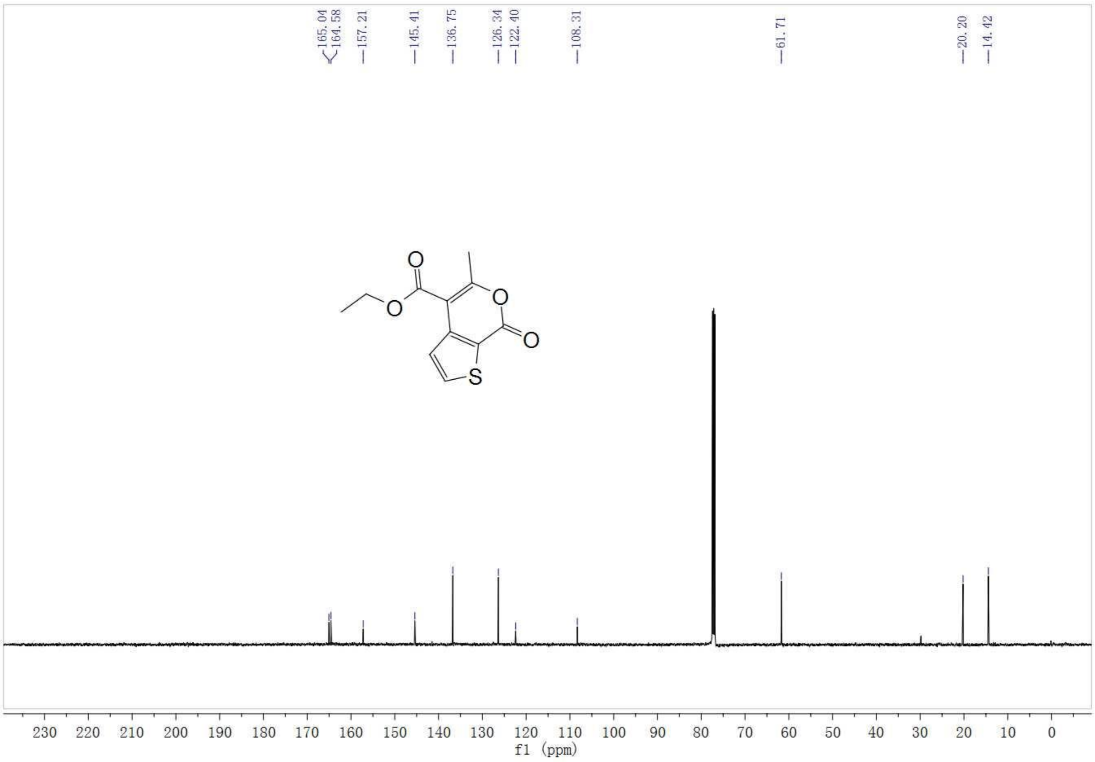

Ethyl 1-oxo-3-phenyl-1*H*-isochromene-4-carboxylate (3ab).

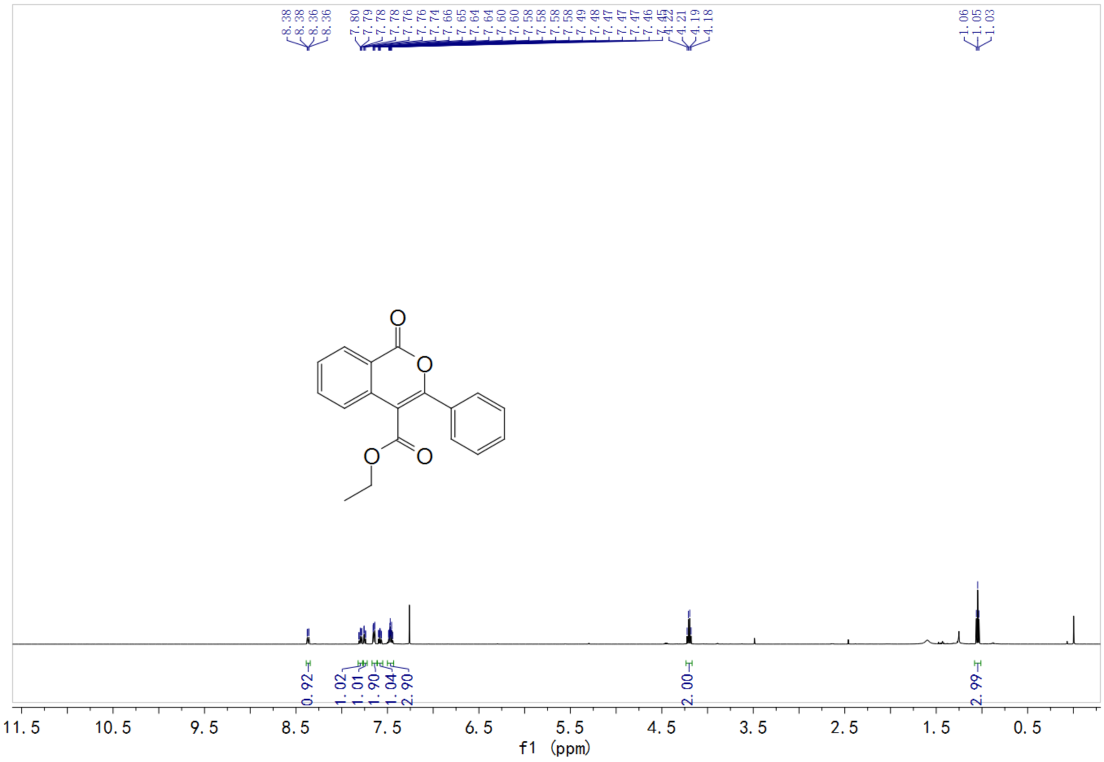

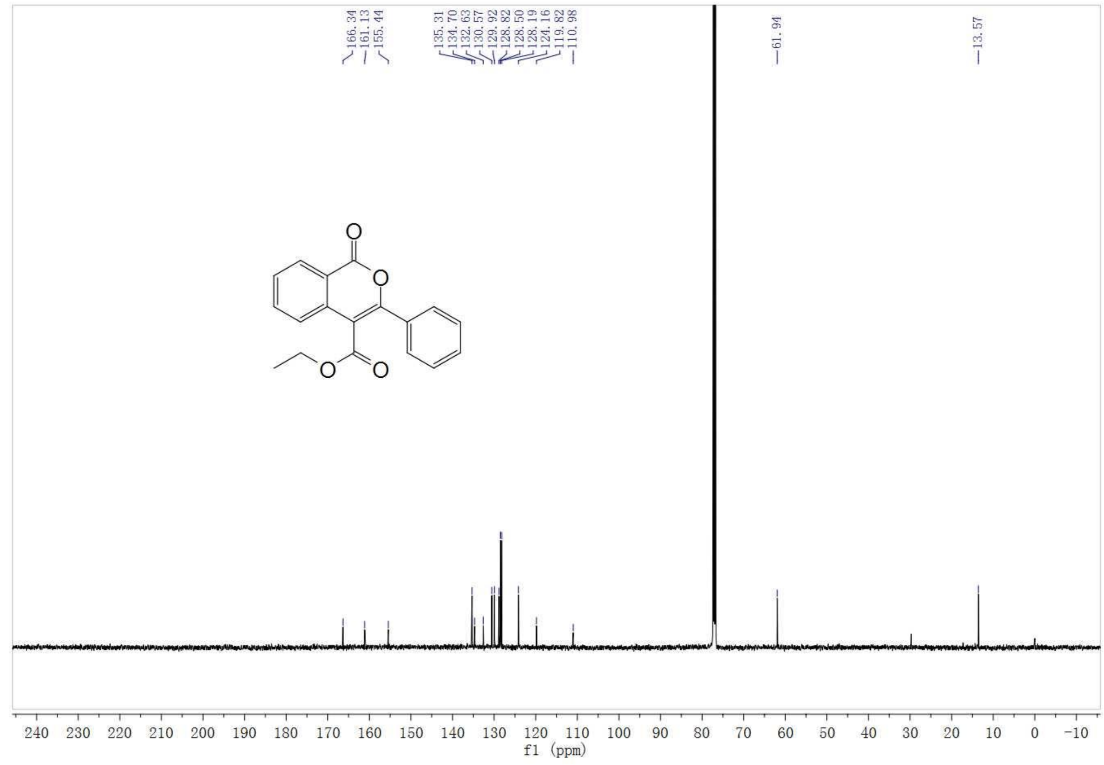

Ethyl 3-(4-methoxyphenyl)-1-oxo-1*H*-isochromene-4-carboxylate (3ac).

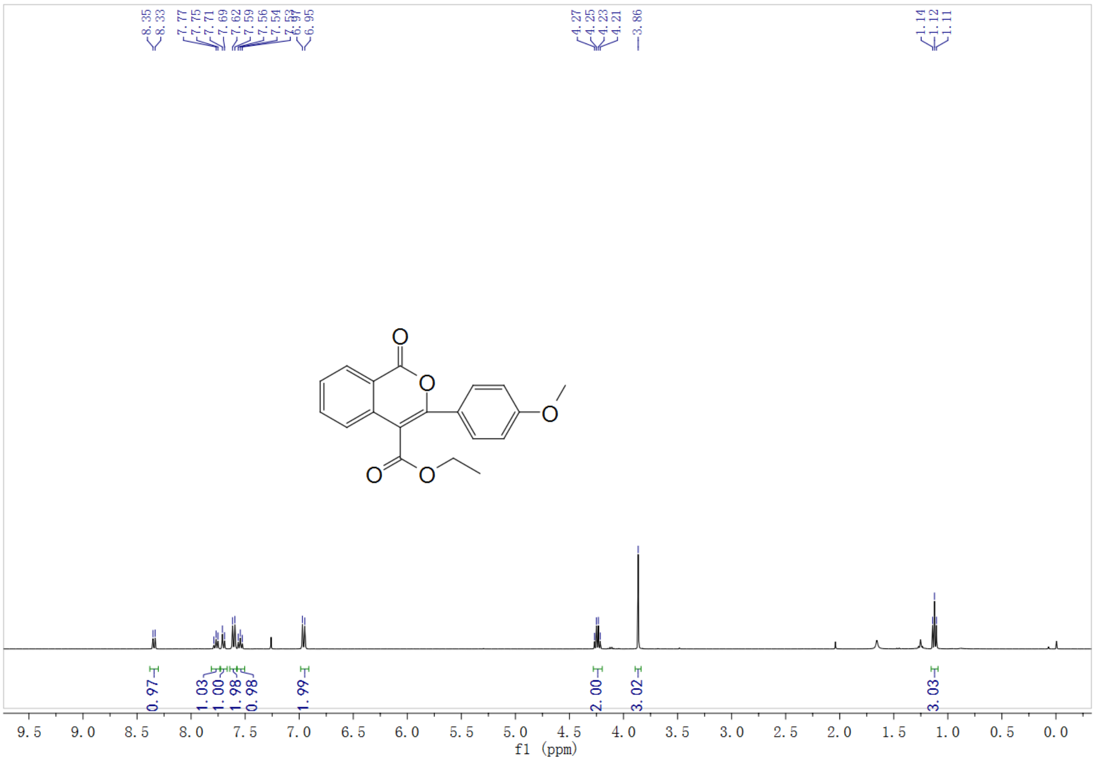

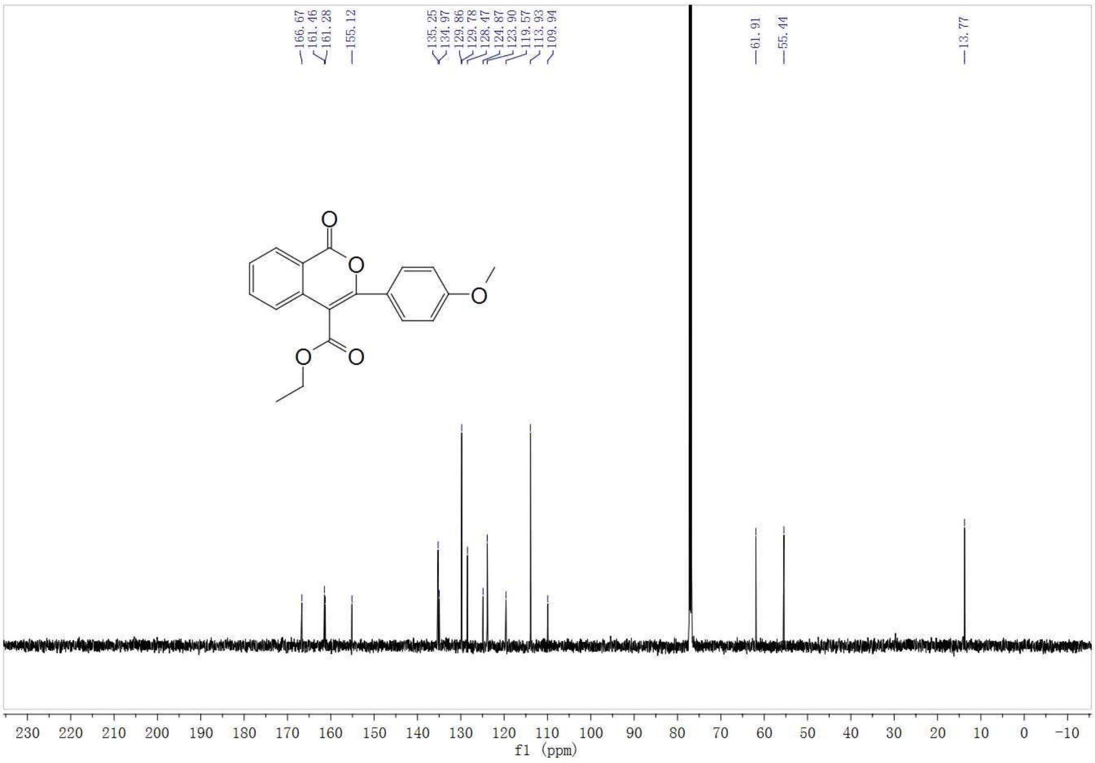

Ethyl 3-(4-chlorophenyl)-1-oxo-1*H*-isochromene-4-carboxylate(3ad).

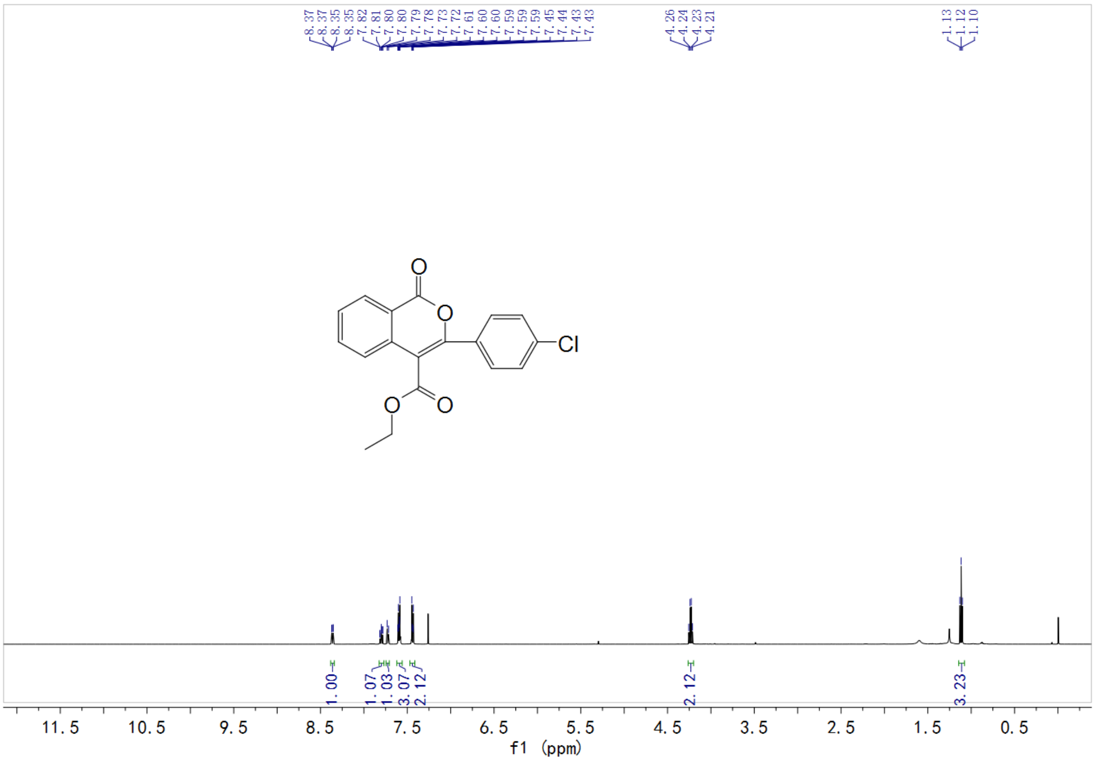

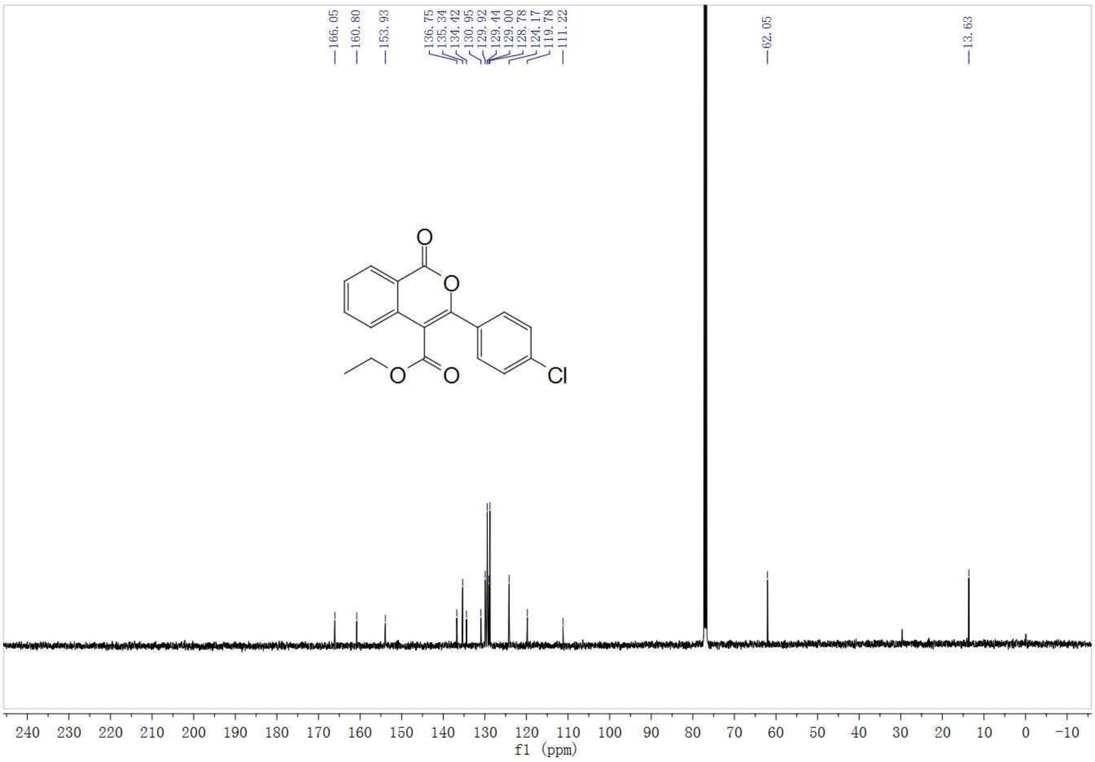

Ethyl 3-(naphthalen-2-yl)-1-oxo-1*H*-isochromene-4-carboxylate (3ae).

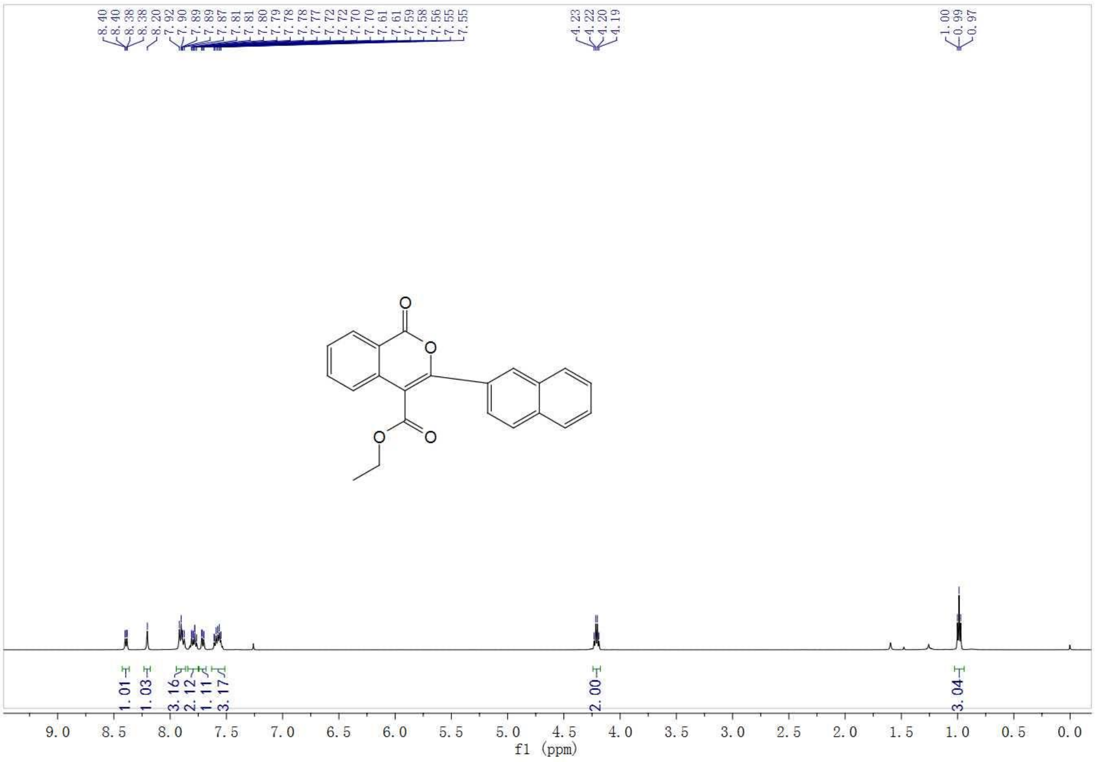

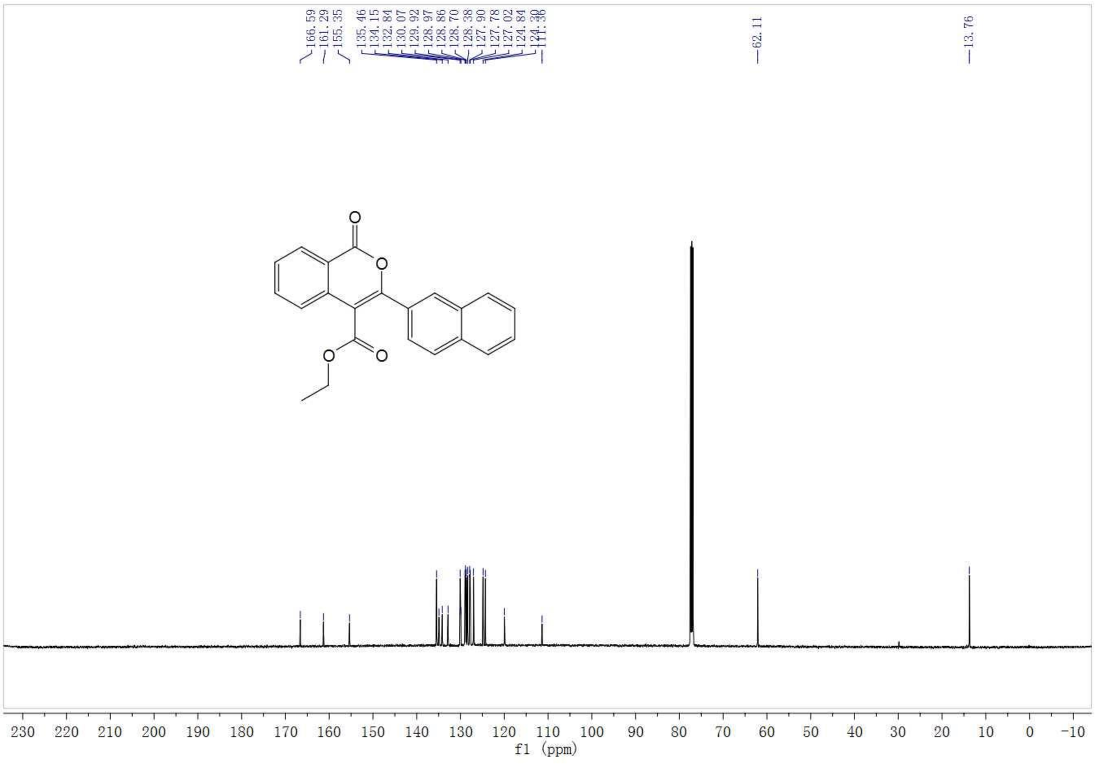

Ethyl 3-cyclopropyl-1-oxo-1*H*-isochromene-4-carboxylate (3af).

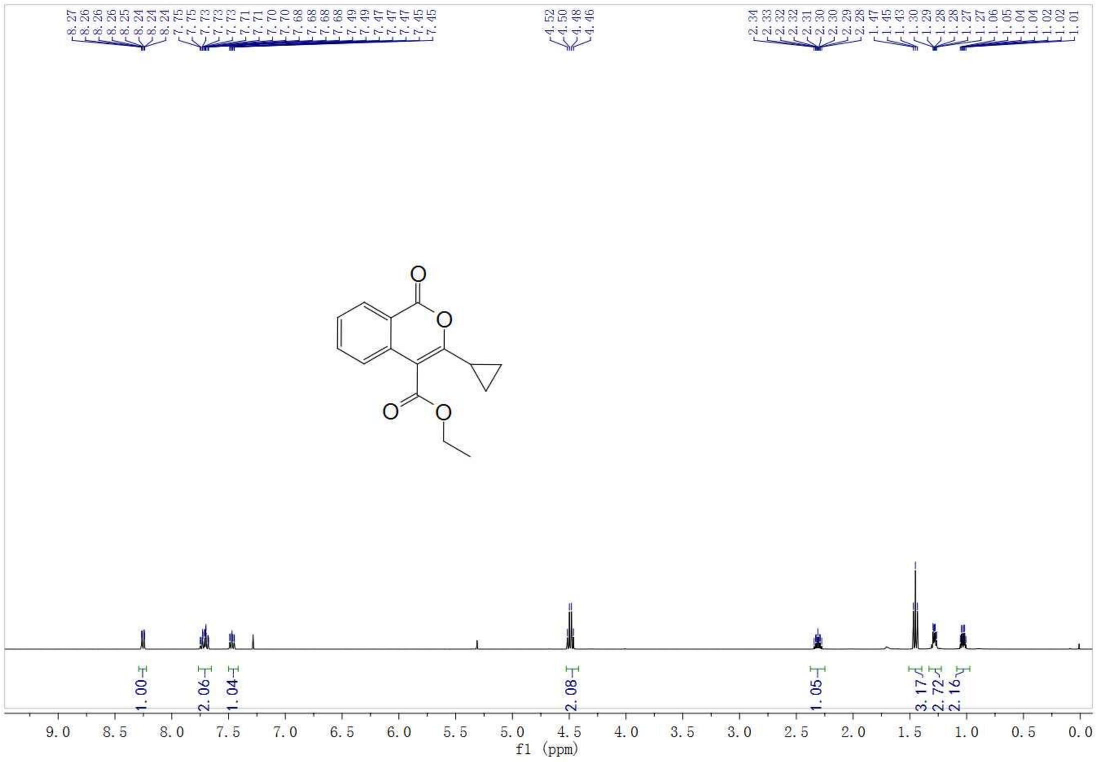

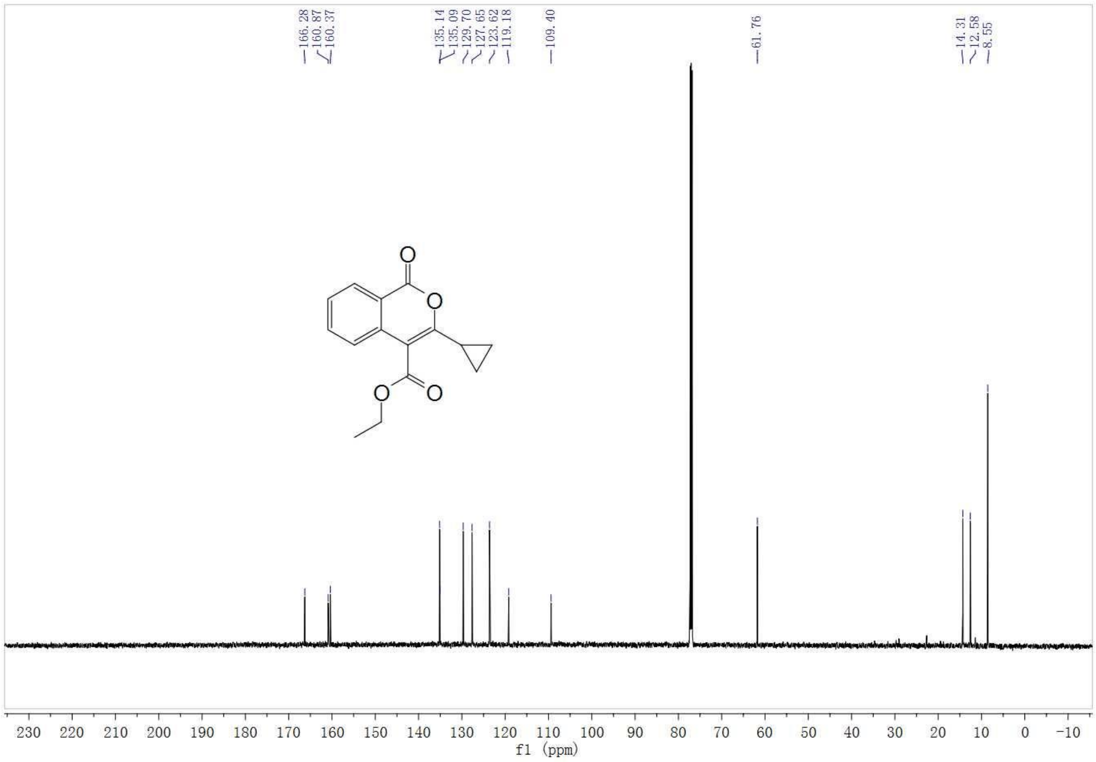

Ethyl 3-cyclohexyl-1-oxo-1*H*-isochromene-4-carboxylate (3ag).

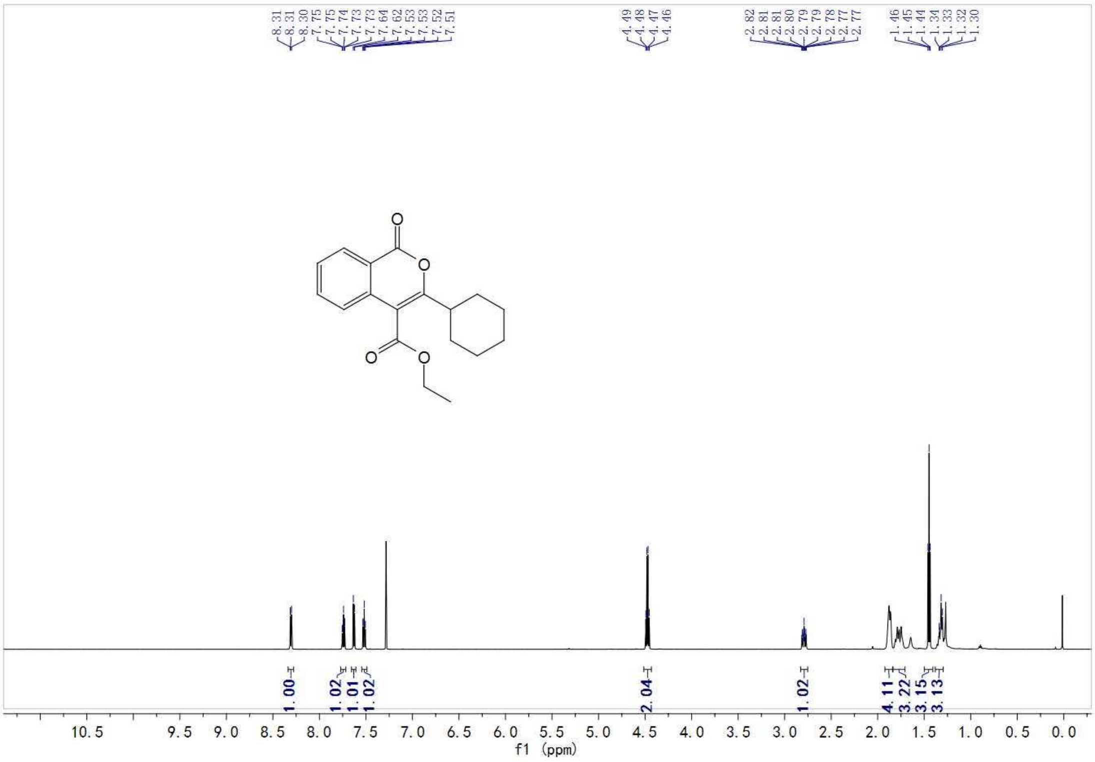

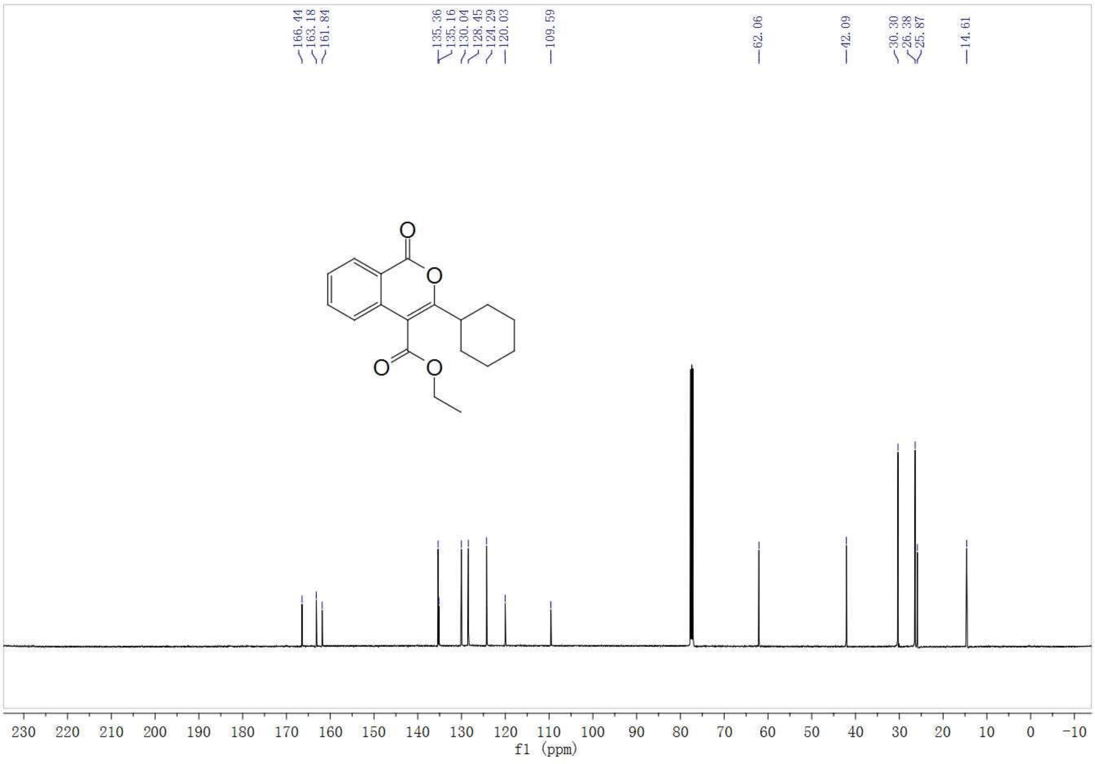

7,8,9,10-tetrahydrocyclohepta[c]isochromene-5,11-dione (3ah).

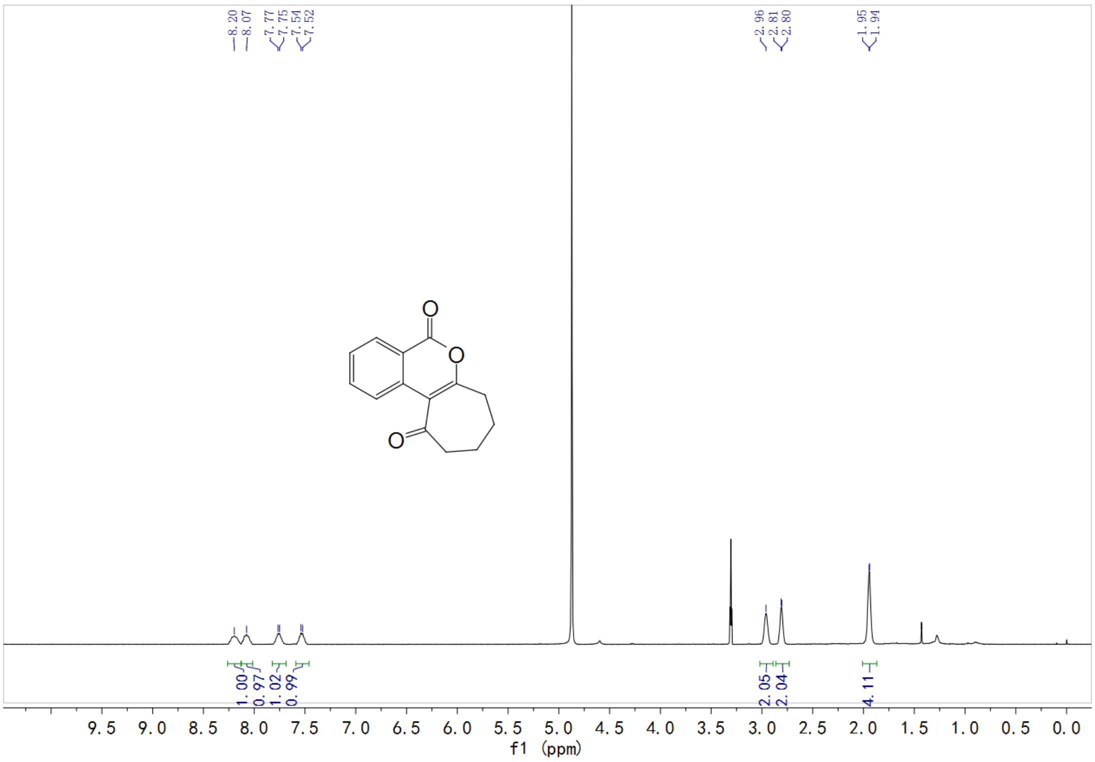

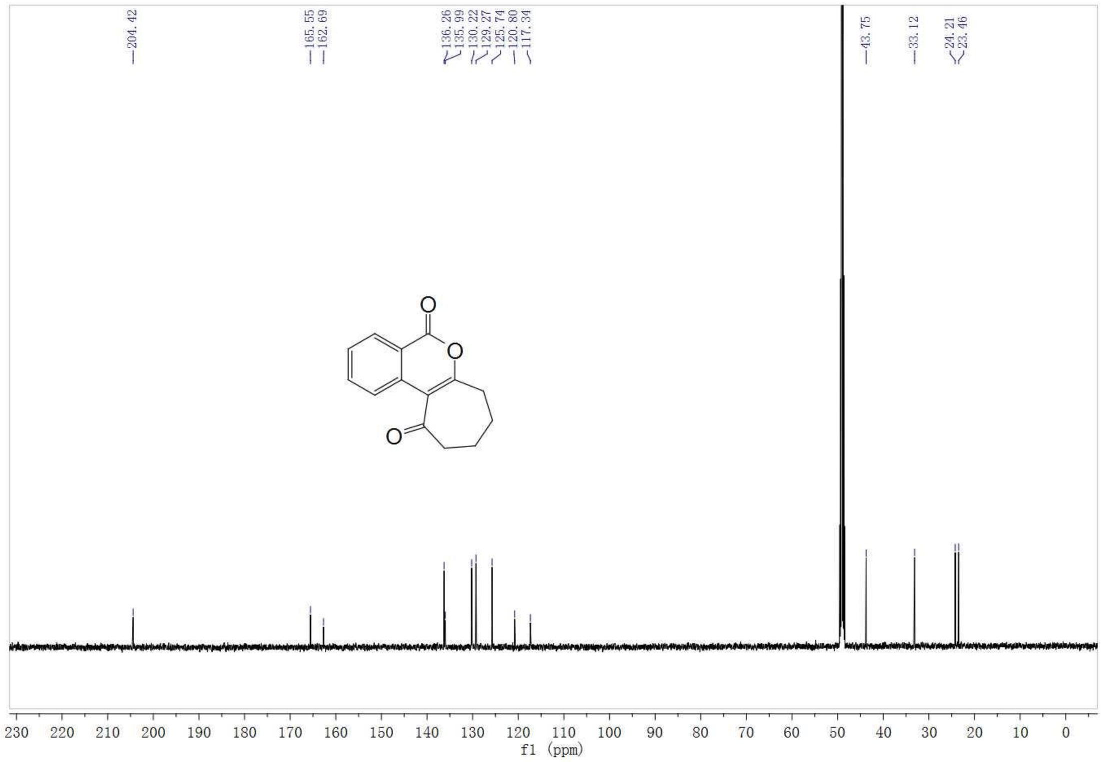

Ethyl 3-isopropyl-1-oxo-1*H*-isochromene-4-carboxylate (3ai).

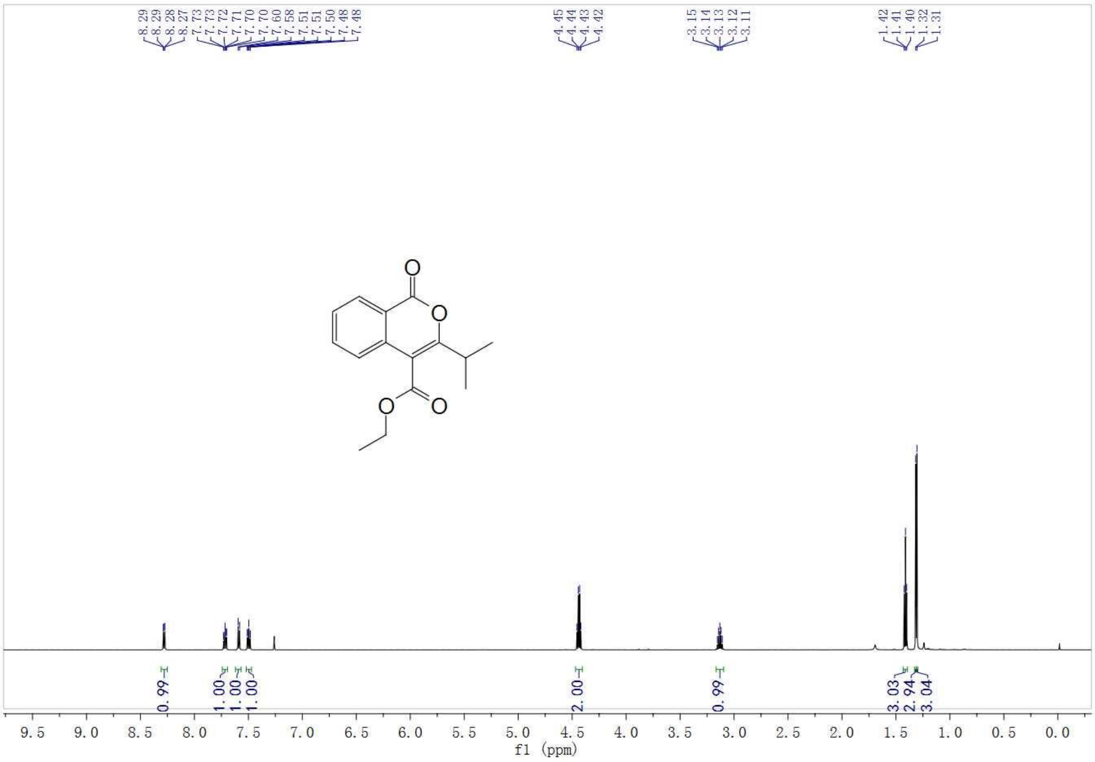

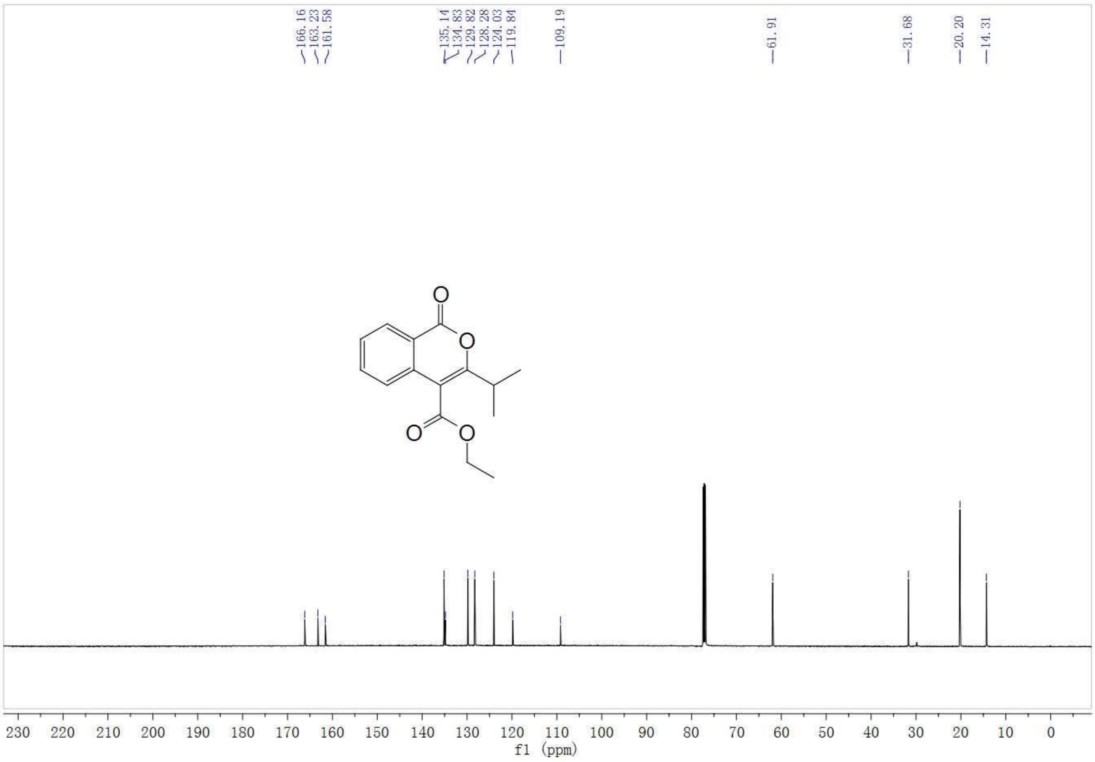

Ethyl 3-ethyl-1-oxo-1H-isochromene-4-carboxylate (3aj).

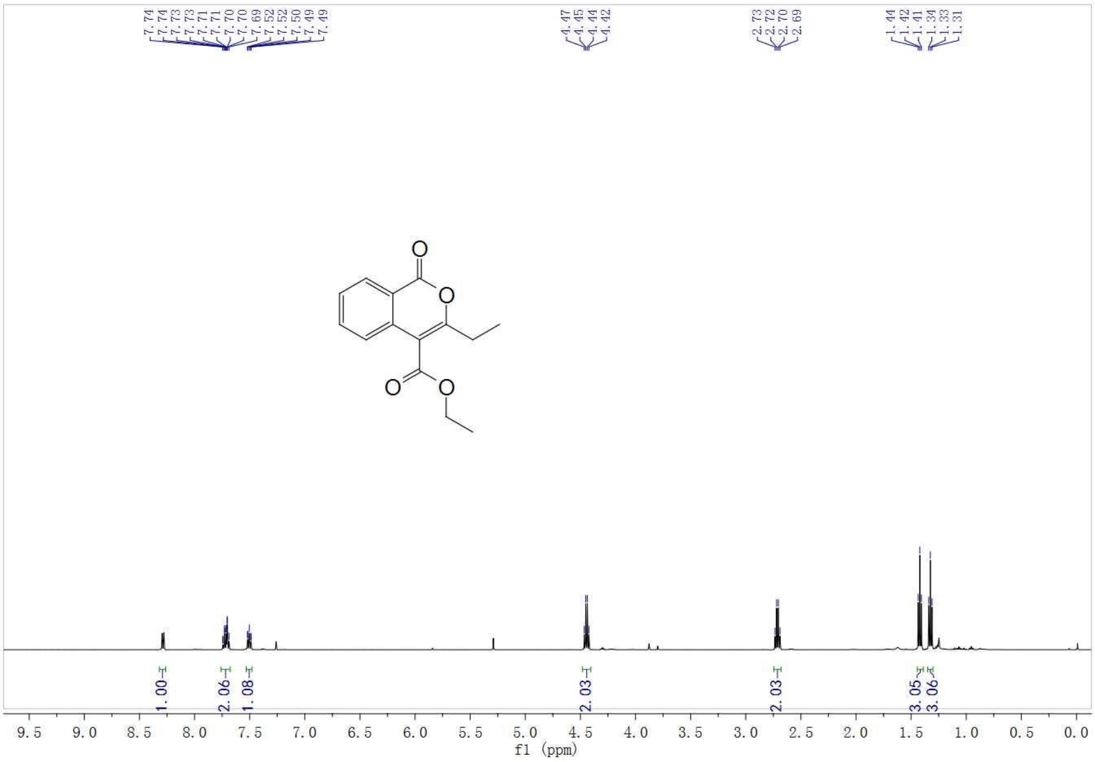

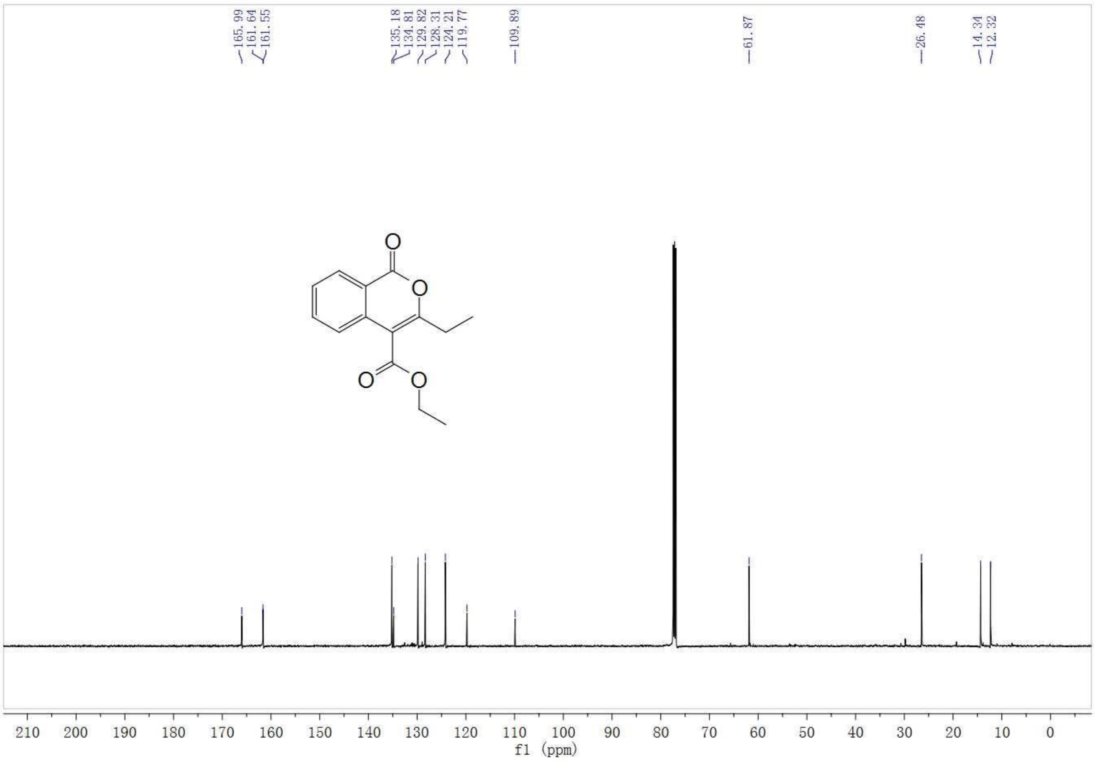

***ftert*-Butyl 3-methyl-1-oxo-1*H*-isochromene-4-carboxylate(3ak).**

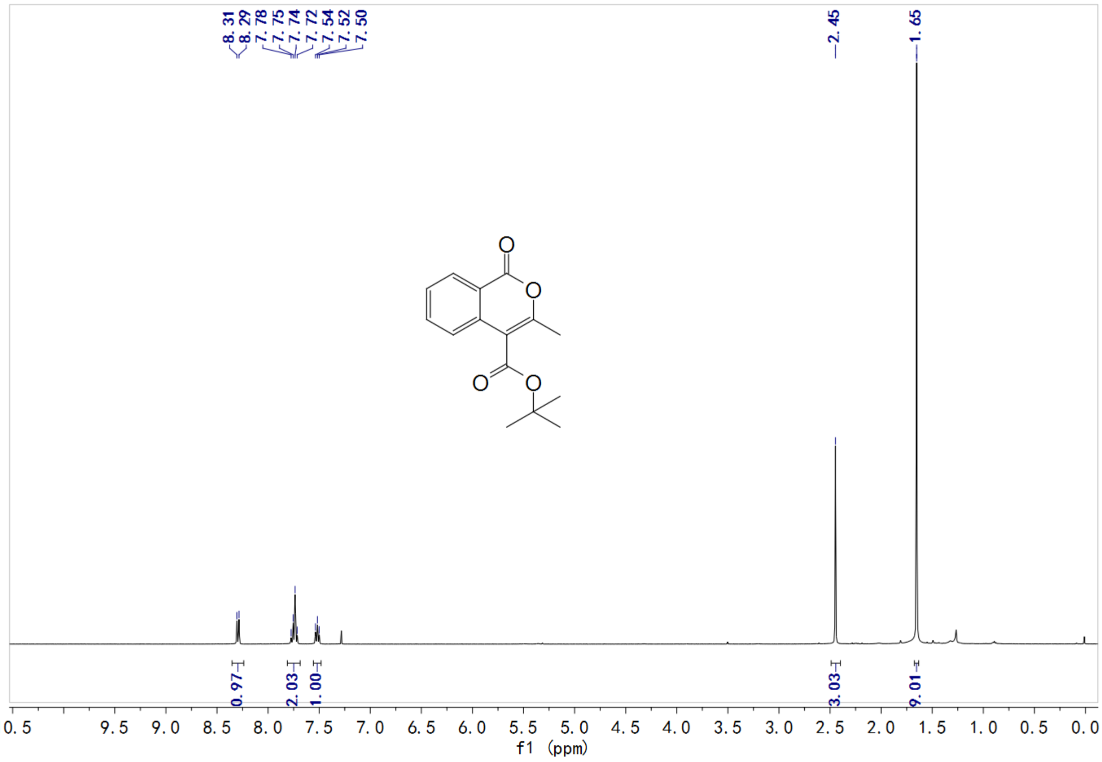

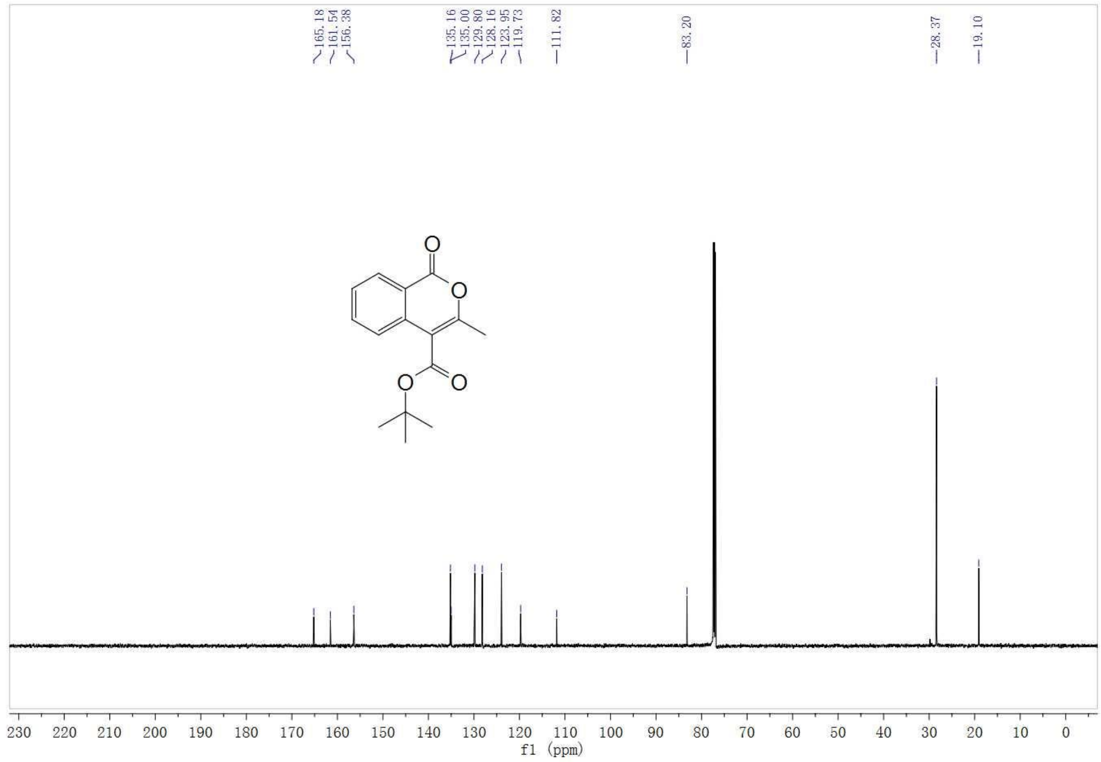

Benzyl 3-methyl-1-oxo-1*H*-isochromene-4-carboxylate(3al).

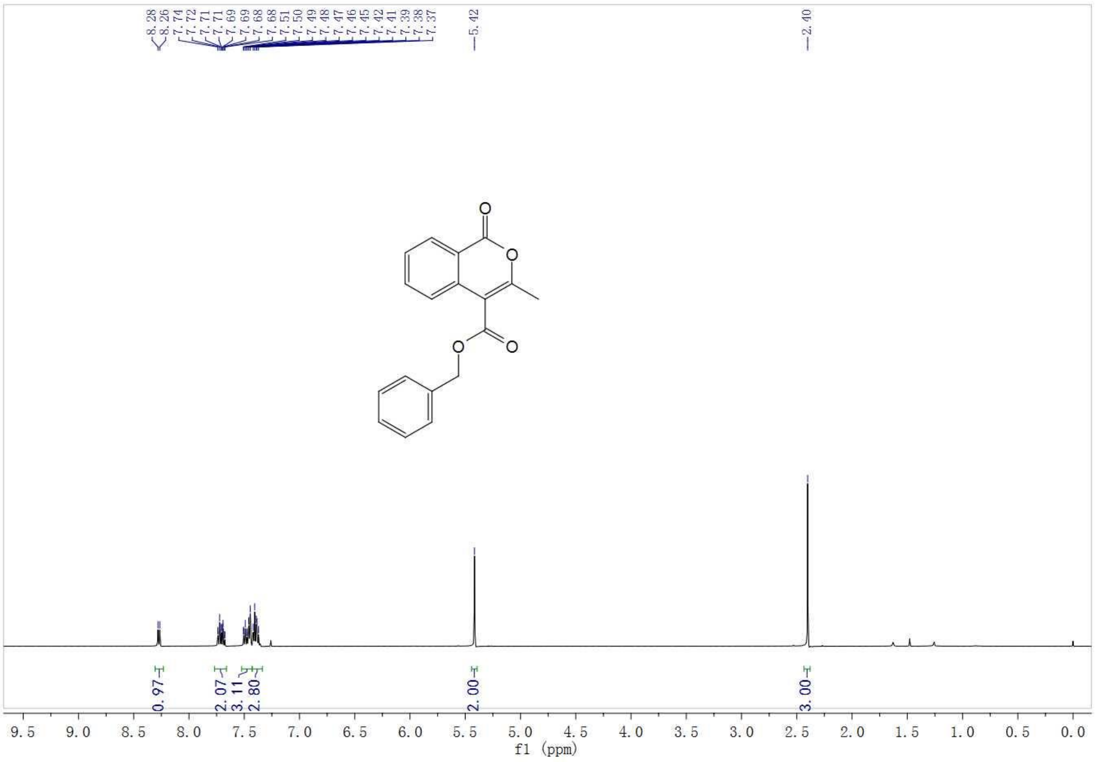

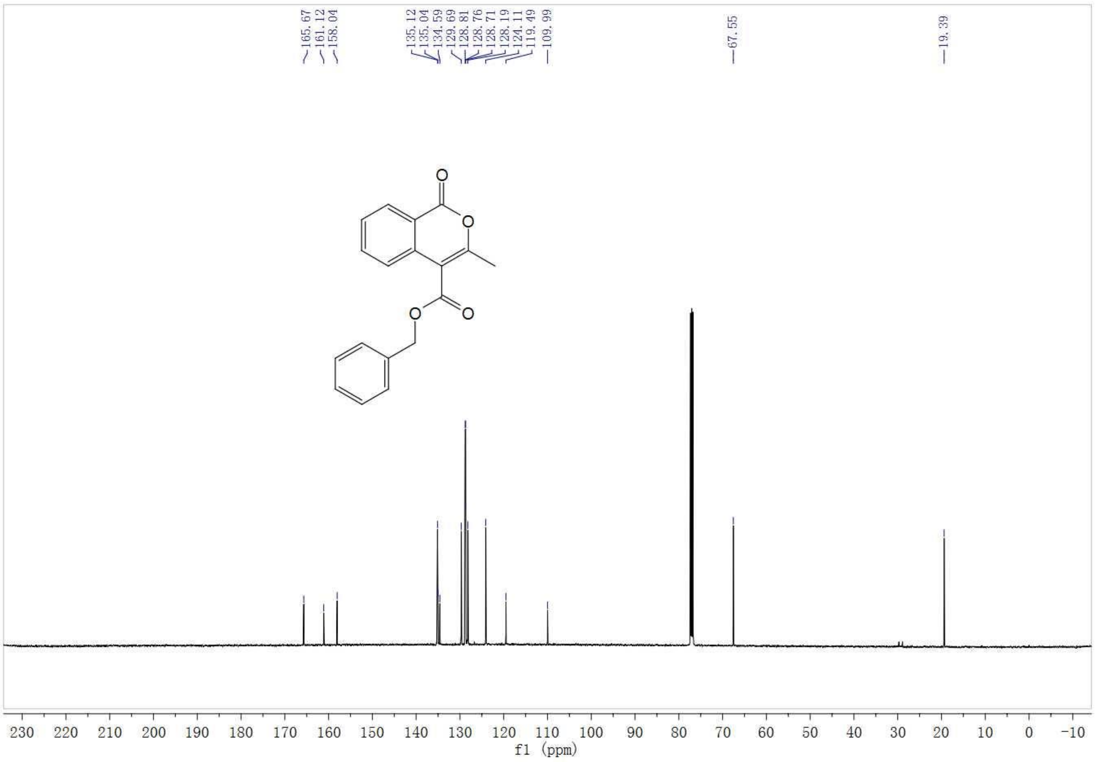

Allyl 3-methyl-1-oxo-1*H*-isochromene-4-carboxylate(3am).

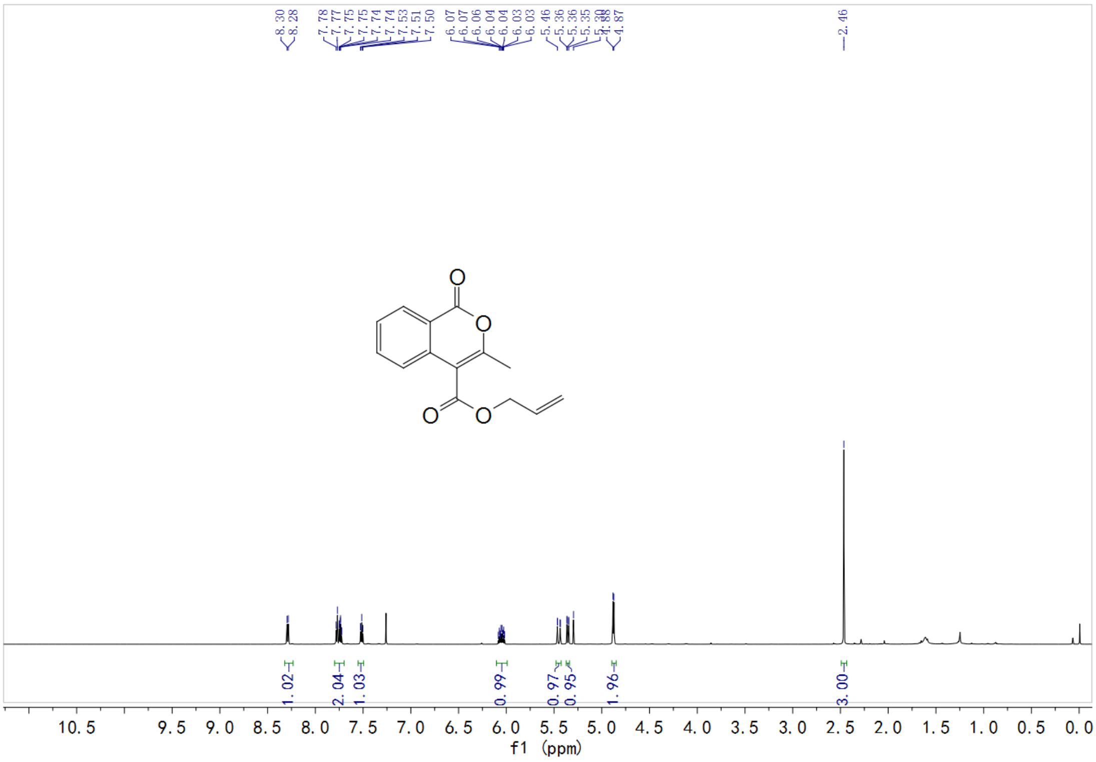

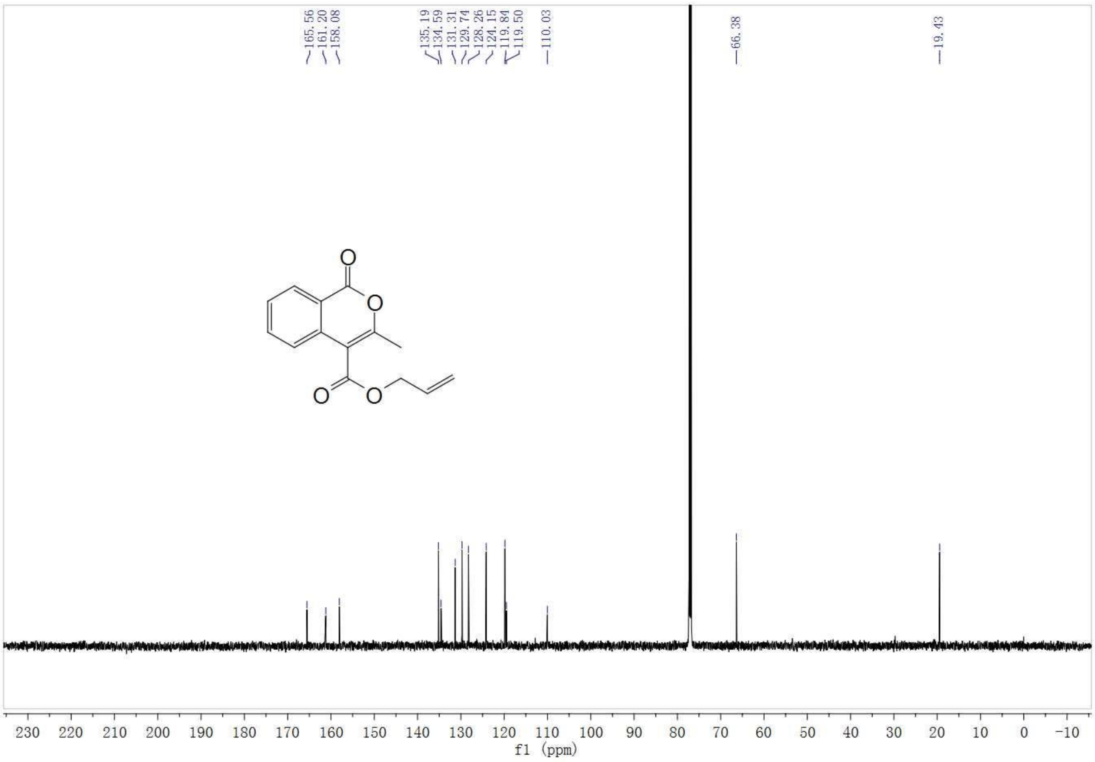

Methyl 3-methyl-1-oxo-1*H*-isochromene-4-carboxylate (3an).

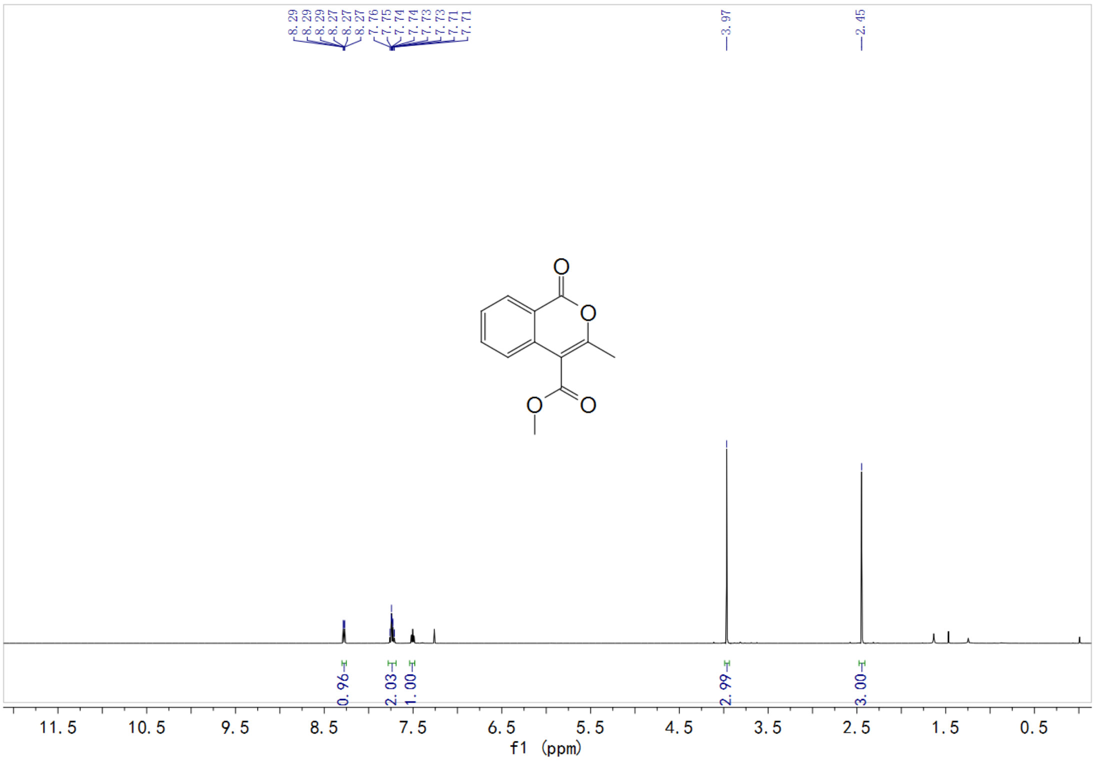

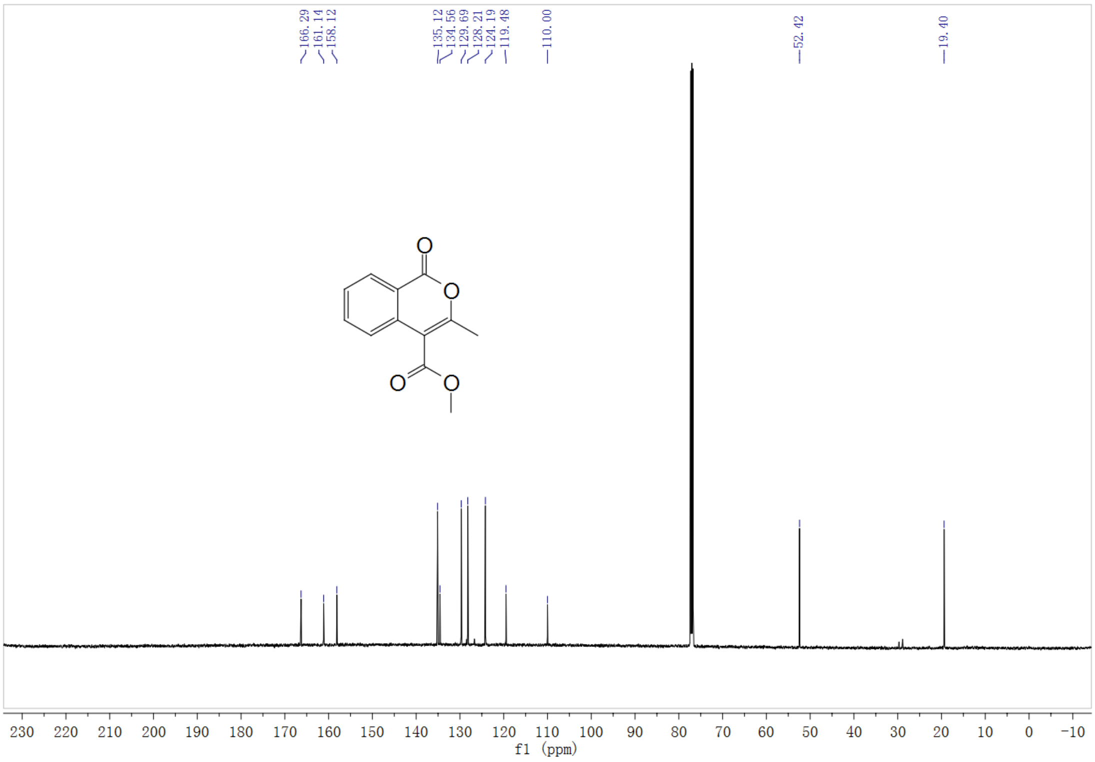

Propyl 3-methyl-1-oxo-1*H*-isochromene-4-carboxylate (3ao).

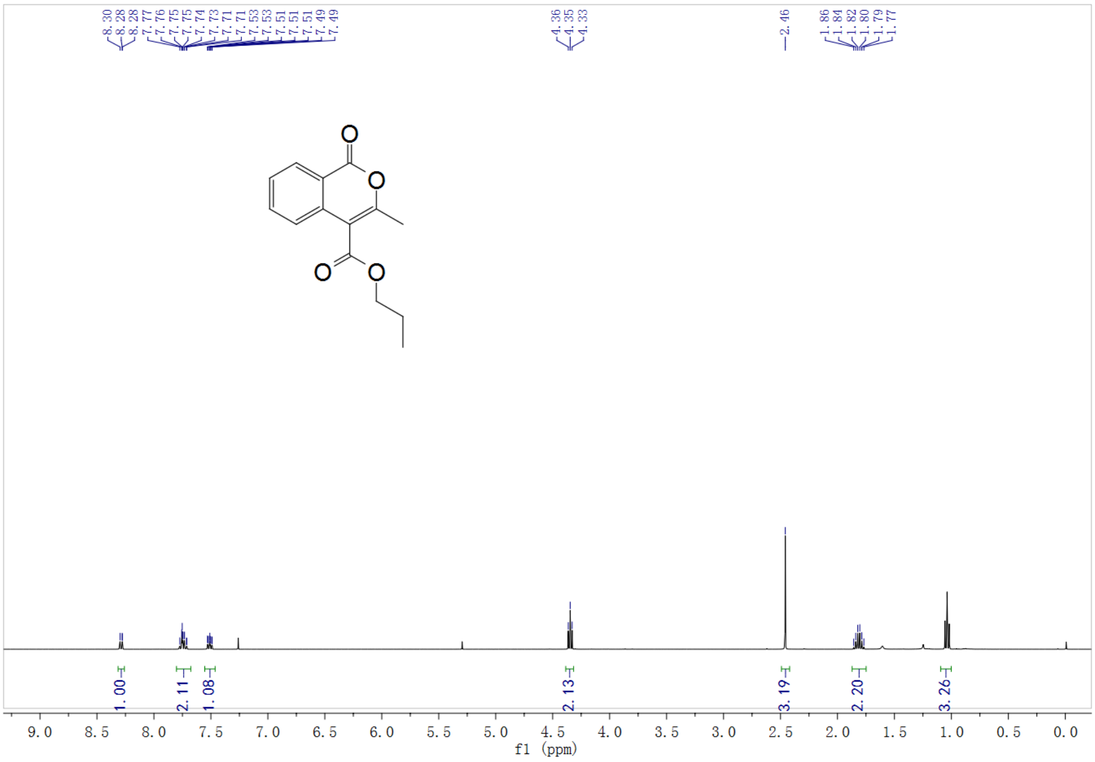

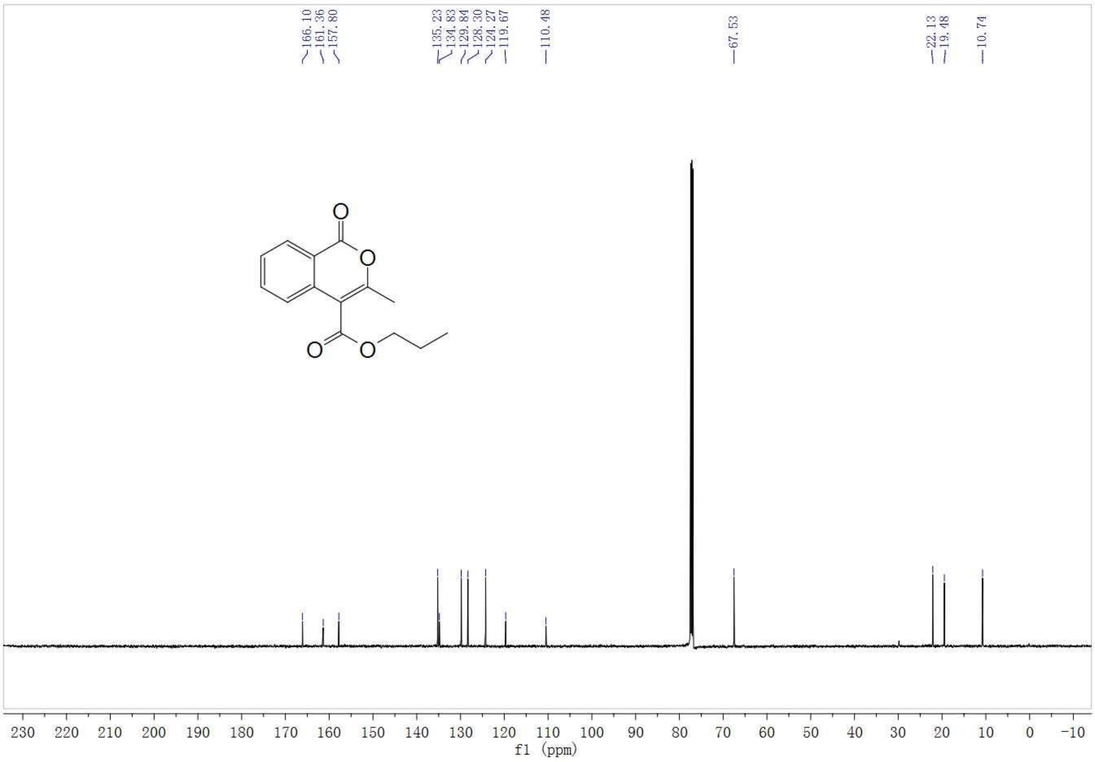

Ethyl 3-methyl-1-oxo-1,2-dihydroisoquinoline-4-carboxylate (4)

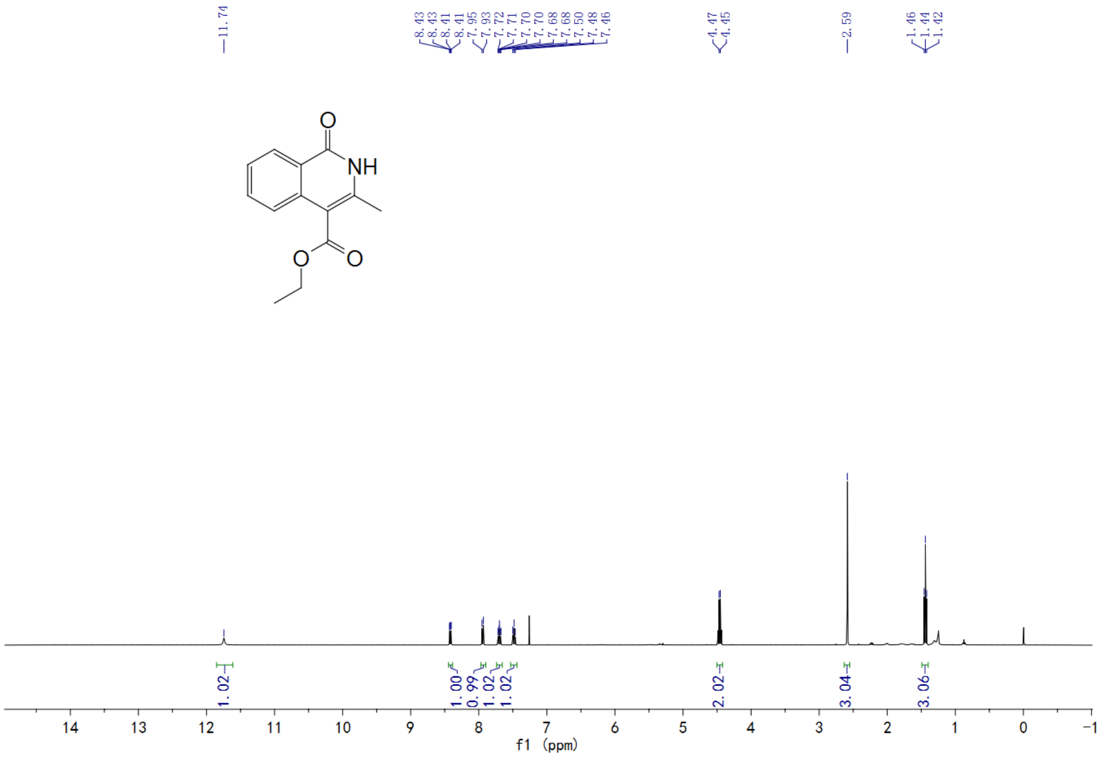

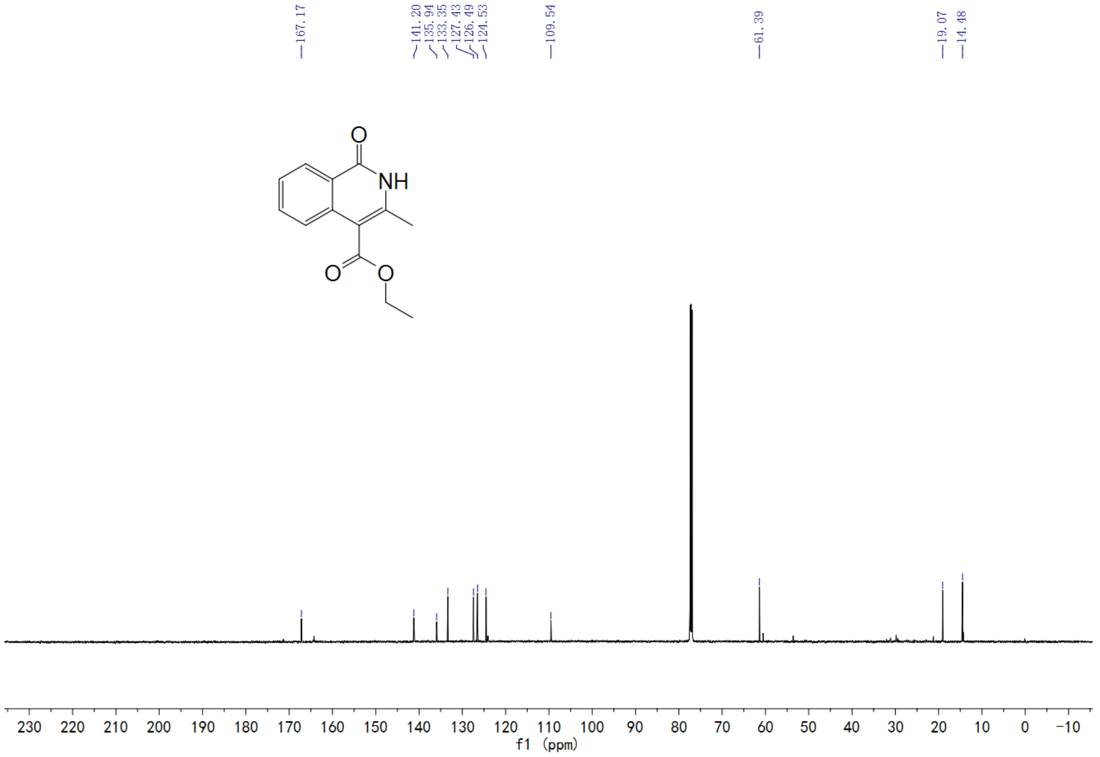

3-methyl-1H-isochromen-1-one (5)

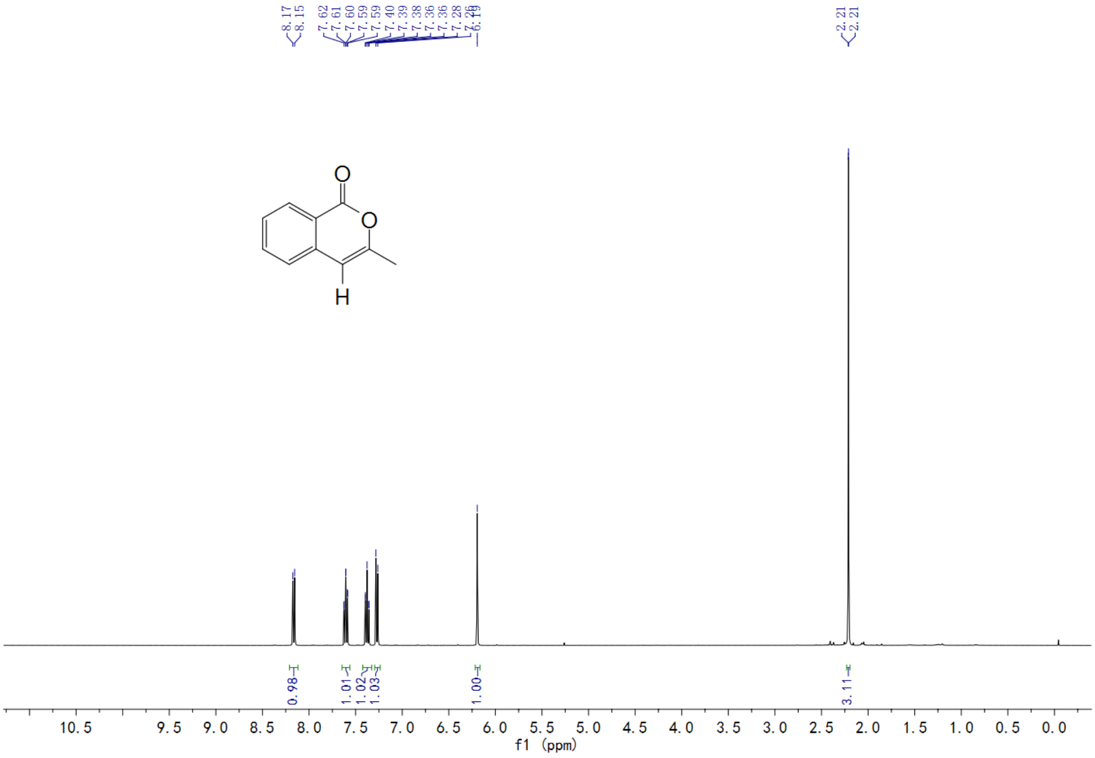
8.17
8.15
7.62
7.61
7.60
7.59
7.59
7.40
7.39
7.38
7.36
7.36
7.28
7.26
6.19
2.21
2.21
O
O
H
0.98
1.01
1.02
1.03
1.00
3.11
10.5
9.5
9.0
8.5
8.0
7.5
7.0
6.5
6.0
5.5
5.0
4.5
4.0
3.5
3.0
2.5
2.0
1.5
1.0
0.5
0.0
f1 (ppm)

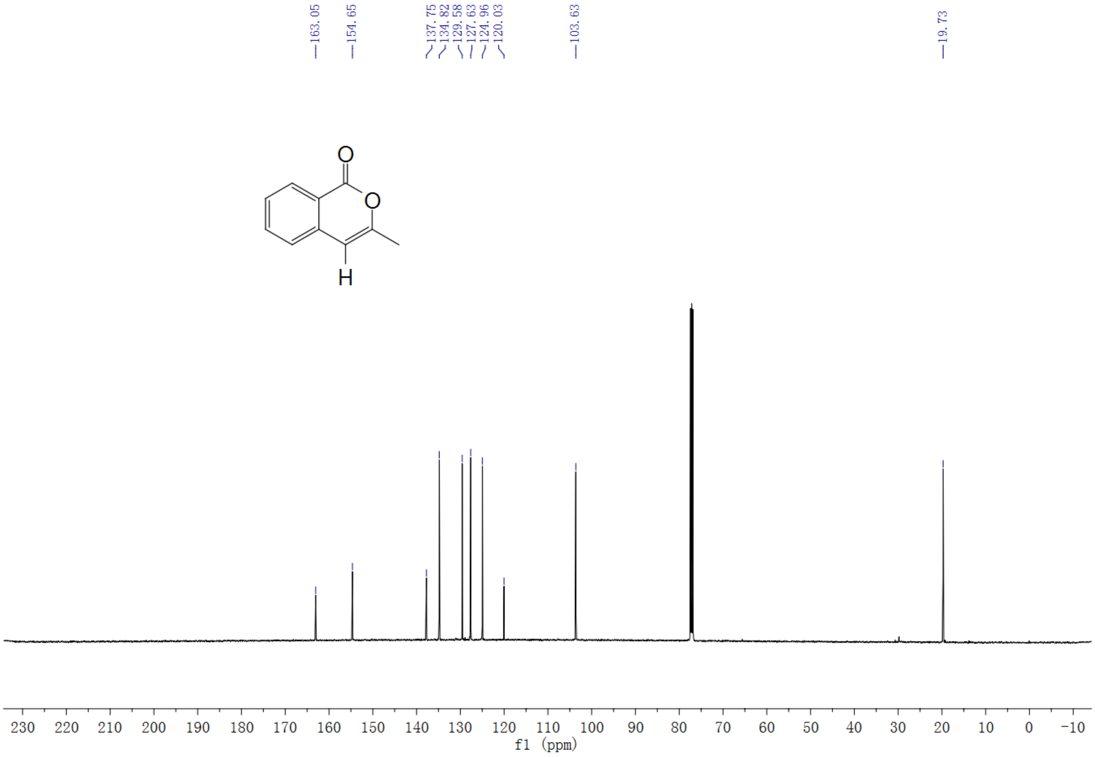
163.05
154.65
137.75
134.82
129.58
127.63
124.96
120.03
103.63
19.73
O
O
H
230
220
210
200
190
180
170
160
150
140
130
120
110
100
90
80
70
60
50
40
30
20
10
0
-10
f1 (ppm)

Scheme 3b

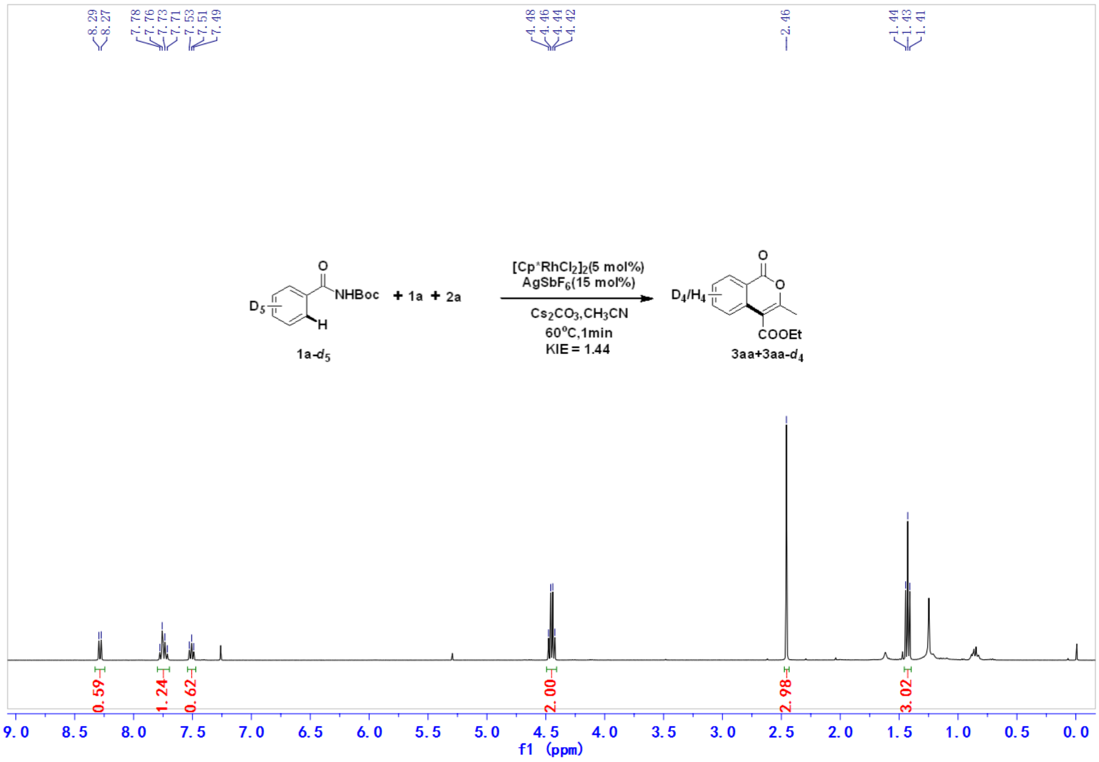
8.29
8.27
7.78
7.76
7.73
7.71
7.53
7.51
7.49
4.48
4.46
4.44
4.42
2.46
1.44
1.43
1.41
1a-d_5
NHBoc
+ 1a + 2a
$[Cp^*RhCl_2]_2$(5 mol%)
$AgSbF_6$(15 mol%)
Cs_2CO_3,CH_3CN
60°C,1min
KIE = 1.44
D_4/H_4
COOEt
3aa+3aa-d_4
0.59
1.24
0.62
2.00
2.98
3.02
9.0
8.5
8.0
7.5
7.0
6.5
6.0
5.5
5.0
4.5
4.0
3.5
3.0
2.5
2.0
1.5
1.0
0.5
0.0
f1 (ppm)

5. MS Spectra of benzamides.

3c

**Data Filename**	ESIH_20181012_LH_DGY_09.d	**Sample Name**	B6-DW-37
**Sample Type**	Sample	**Position**	P1-B5
**Instrument Name**	Agilent G6520 Q-TOF	**Acq Method**	20160322_MS_ESIH_POS_1min.m
**Acquired Time**	10/12/2018 14:25:47	**IRM Calibration Status**	Success
**DA Method**	small molecular data analysis method.m	**Comment**	ESIH by ZZY

User Spectra

Fragmentor Voltage	Collision Energy	Ionization Mode
175	0	ESI

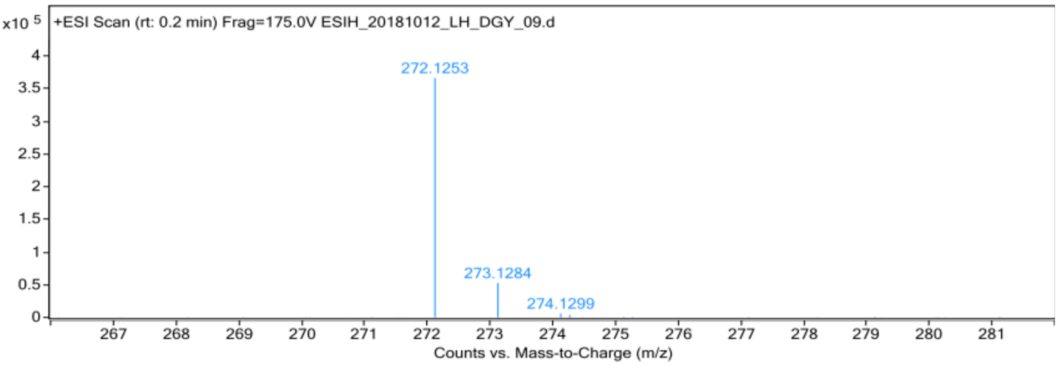

Formula Calculator Results

m/z	Calc m/z	Diff (mDa)	Diff (ppm)	Ion Formula	Ion
272.1253	272.1257	0.46	1.69	C14 H19 N Na O3	(M+Na)+

3d

**Data Filename**	ESIH_20181012_LH_DGY_08.d	**Sample Name**	B6-DW-35
**Sample Type**	Sample	**Position**	P1-B4
**Instrument Name**	Agilent G6520 Q-TOF	**Acq Method**	20160322_MS_ESIH_POS_1min.m
**Acquired Time**	10/12/2018 14:23:56	**IRM Calibration Status**	Success
**DA Method**	small molecular data analysis method.m	**Comment**	ESIH by ZZY

User Spectra

Fragmentor Voltage	Collision Energy	Ionization Mode
175	0	ESI

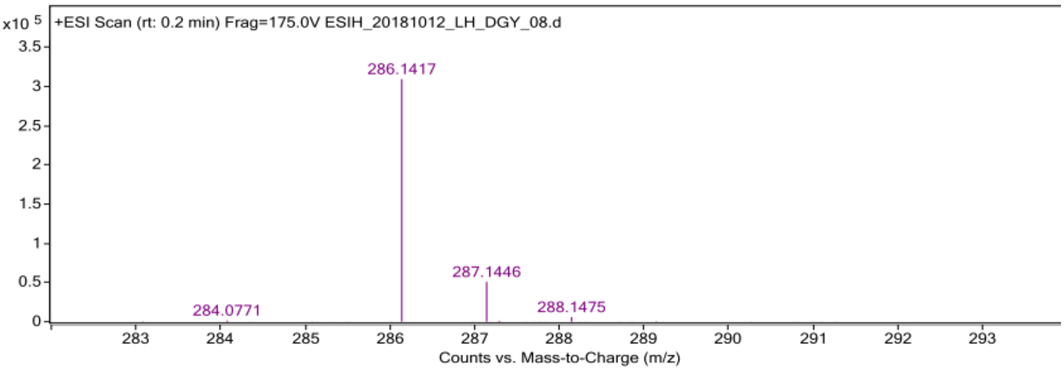

Formula Calculator Results

m/z	Calc m/z	Diff (mDa)	Diff (ppm)	Ion Formula	Ion
286.1417	286.1414	-0.29	-1.01	C15 H21 N Na O3	(M+Na)+

3e

**Data Filename**	ESIH_20180929_LH_DGY_21.d	**Sample Name**	B6-0929-DW28
**Sample Type**	Sample	**Position**	P1-B3
**Instrument Name**	Agilent G6520 Q-TOF	**Acq Method**	20160324_MS_ESIH_NEG_1min.m
**Acquired Time**	9/29/2018 15:11:07	**IRM Calibration Status**	Success
**DA Method**	small molecular data analysis method.m	**Comment**	ESIH by ZZY

User Spectra

Fragmentor Voltage	Collision Energy	Ionization Mode
175	0	ESI

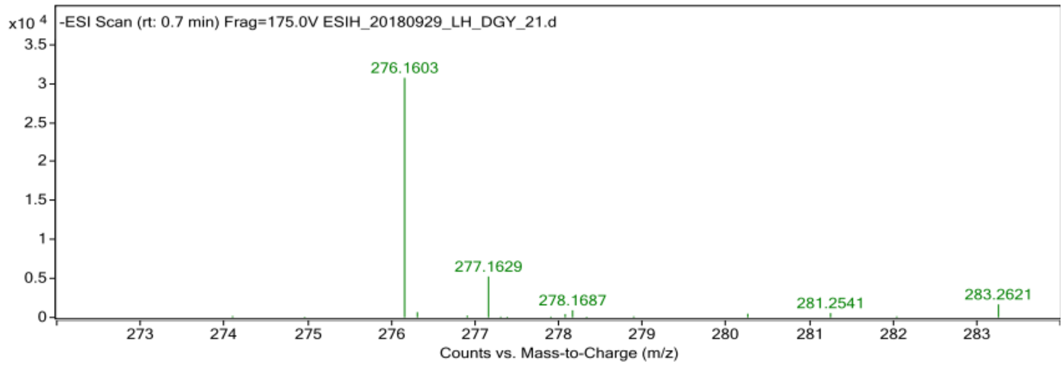

Formula Calculator Results

m/z	Calc m/z	Diff (mDa)	Diff (ppm)	Ion Formula	Ion
276.1603	276.1605	0.19	0.71	C16 H22 N O3	(M-H)-

3f

**Data Filename**	ESIH_20180929_LH_DGY_24.d	**Sample Name**	B6-0929-DW11
**Sample Type**	Sample	**Position**	P1-B6
**Instrument Name**	Agilent G6520 Q-TOF	**Acq Method**	20160322_MS_ESIH_POS_1min.m
**Acquired Time**	9/29/2018 15:16:41	**IRM Calibration Status**	Success
**DA Method**	small molecular data analysis method.m	**Comment**	ESIH by ZZY

User Spectra

Fragmentor Voltage	Collision Energy	Ionization Mode
175	0	ESI

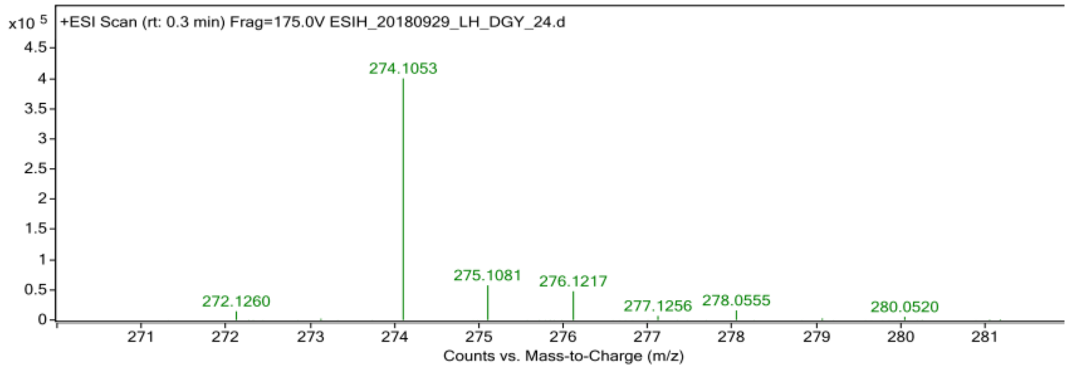

Formula Calculator Results

m/z	Calc m/z	Diff (mDa)	Diff (ppm)	Ion Formula	Ion
274.1053	274.105	-0.28	-1.02	C13 H17 N Na O4	(M+Na)+

3g

**Data Filename**	ESIH_20180929_LH_DGY_13.d	**Sample Name**	B6-0929-DW9
**Sample Type**	Sample	**Position**	P1-A4
**Instrument Name**	Agilent G6520 Q-TOF	**Acq Method**	20160322_MS_ESIH_POS_1min.m
**Acquired Time**	9/29/2018 14:56:14	**IRM Calibration Status**	Success
**DA Method**	small molecular data analysis method.m	**Comment**	ESIH by ZZY

User Spectra

Fragmentor Voltage	Collision Energy	Ionization Mode
175	0	ESI

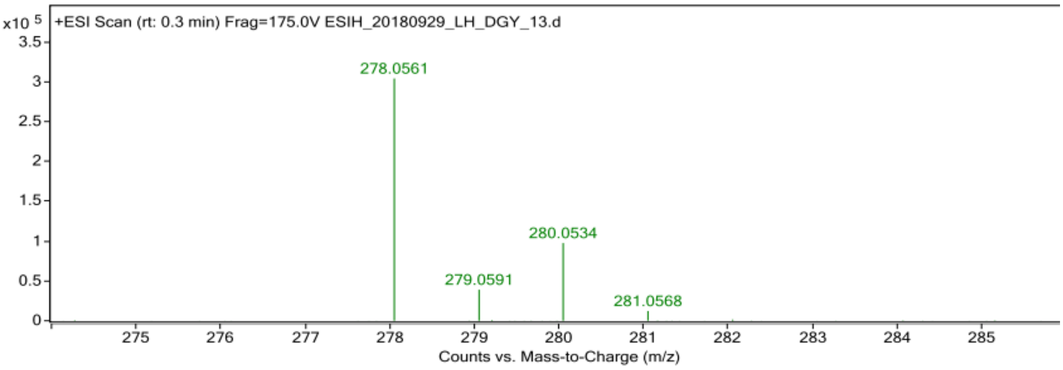

Formula Calculator Results

m/z	Calc m/z	Diff (mDa)	Diff (ppm)	Ion Formula	Ion
278.0561	278.0554	-0.65	-2.33	C12 H14 Cl N Na O3	(M+Na)+

3h

**Data Filename**	ESIH_20180929_LH_DGY_11.d	**Sample Name**	B6-0929-DW5
**Sample Type**	Sample	**Position**	P1-A2
**Instrument Name**	Agilent G6520 Q-TOF	**Acq Method**	20160322_MS_ESIH_POS_1min.m
**Acquired Time**	9/29/2018 14:52:35	**IRM Calibration Status**	Success
**DA Method**	small molecular data analysis method.m	**Comment**	ESIH by ZZY

User Spectra

Fragmentor Voltage	Collision Energy	Ionization Mode
175	0	ESI

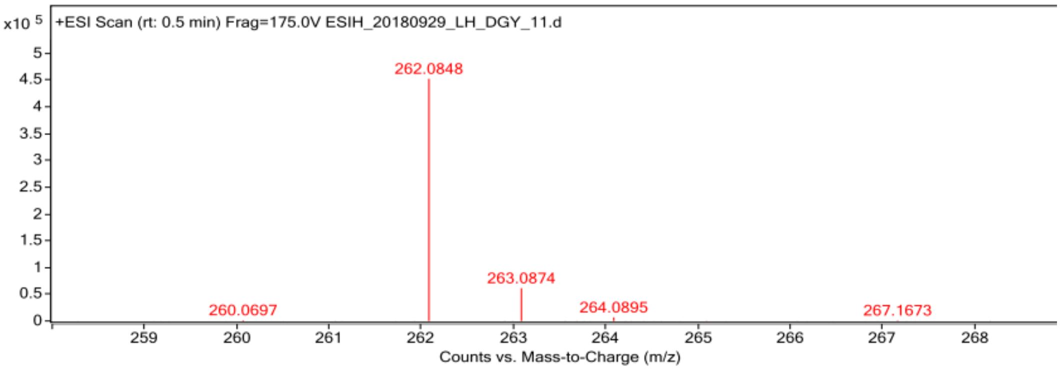

Formula Calculator Results

m/z	Calc m/z	Diff (mDa)	Diff (ppm)	Ion Formula	Ion
262.0848	262.085	0.2	0.77	C12 H14 F N Na O3	(M+Na)+

3i

**Data Filename**	ESIH_20180929_LH_DGY_12.d	**Sample Name**	B6-0929-DW8
**Sample Type**	Sample	**Position**	P1-A3
**Instrument Name**	Agilent G6520 Q-TOF	**Acq Method**	20160322_MS_ESIH_POS_1min.m
**Acquired Time**	9/29/2018 14:54:24	**IRM Calibration Status**	Success
**DA Method**	small molecular data analysis method.m	**Comment**	ESIH by ZZY

User Spectra

Fragmentor Voltage	Collision Energy	Ionization Mode
175	0	ESI

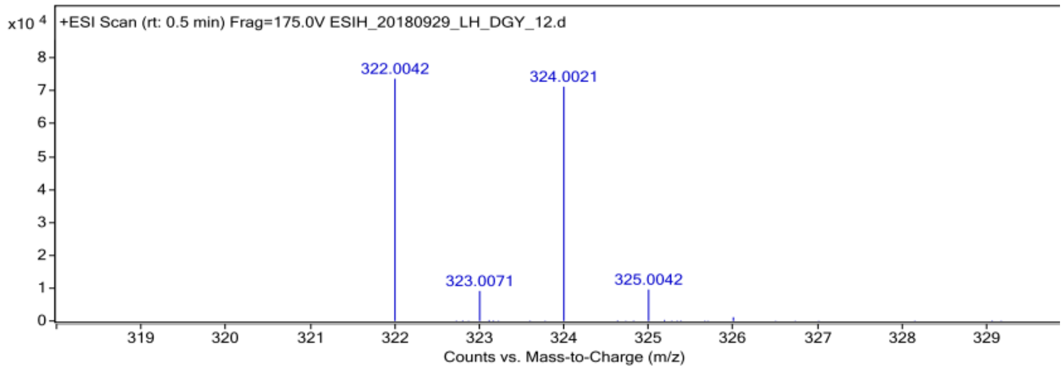

Formula Calculator Results

m/z	Calc m/z	Diff (mDa)	Diff (ppm)	Ion Formula	Ion
322.0042	322.0049	0.7	2.17	C12 H14 Br N Na O3	(M+Na)+

3j

**Data Filename**	ESIH_20181025_LH_DGY_08.d	**Sample Name**	B6-1025-DW38
**Sample Type**	Sample	**Position**	P1-A4
**Instrument Name**	Agilent G6520 Q-TOF	**Acq Method**	20160322_MS_ESIH_POS_1min.m
**Acquired Time**	10/25/2018 14:47:41	**IRM Calibration Status**	Success
**DA Method**	small molecular data analysis method.m	**Comment**	ESIH by ZZY

User Spectra

Fragmentor Voltage	Collision Energy	Ionization Mode
175	0	ESI

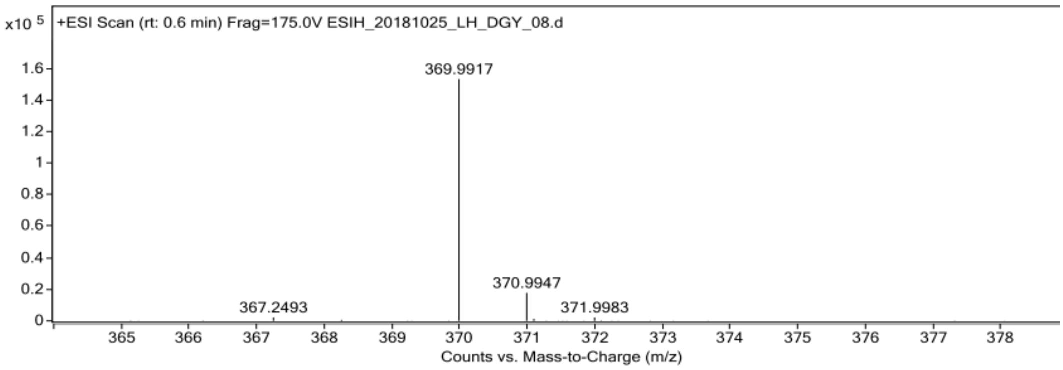

Formula Calculator Results

m/z	Calc m/z	Diff (mDa)	Diff (ppm)	Ion Formula	Ion
369.9917	369.9911	-0.63	-1.7	C12 H14 I N Na O3	(M+Na)+

3k

Data Filename	ESIH_20181017_LH_DGY_08.d	Sample Name	B6-DW-CF3
Sample Type	Sample	Position	P1-B2
Instrument Name	Agilent G6520 Q-TOF	Acq Method	20160322_MS_ESIH_POS_1min.m
Acquired Time	10/17/2018 16:37:38	IRM Calibration Status	Success
DA Method	small molecular data analysis method.m	Comment	ESIH by ZZY

User Spectra

Fragmentor Voltage	Collision Energy	Ionization Mode
175	0	ESI

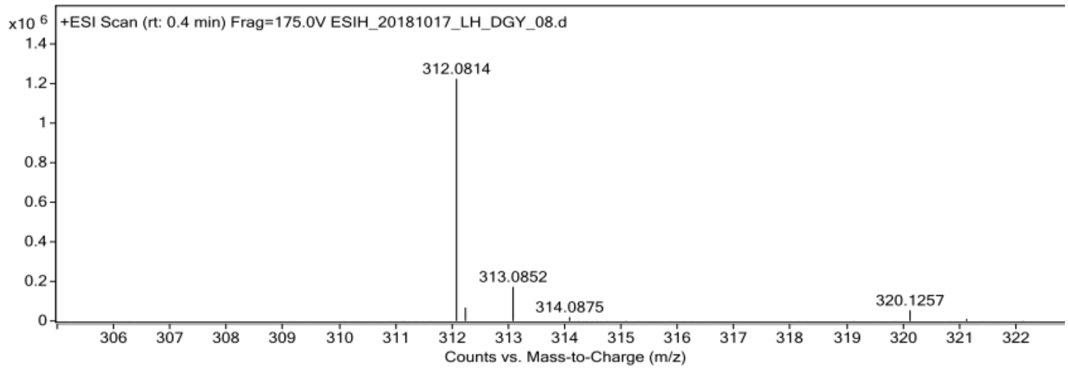

Formula Calculator Results

m/z	Calc m/z	Diff (mDa)	Diff (ppm)	Ion Formula	Ion
312.0814	312.0818	0.4	1.28	C13 H14 F3 N Na O3	(M+Na)+

3l

Data Filename	ESIH_20181126_LH_DGY_40.d	Sample Name	B6-DW-NO2
Sample Type	Sample	Position	P2-E3
Instrument Name	Agilent G6520 Q-TOF	Acq Method	20160322_MS_ESIH_POS_1min.m
Acquired Time	11/26/2018 16:23:28	IRM Calibration Status	Success
DA Method	small molecular data analysis method.m	Comment	ESIH by ZZY

User Spectra

Fragmentor Voltage	Collision Energy	Ionization Mode
175	0	ESI

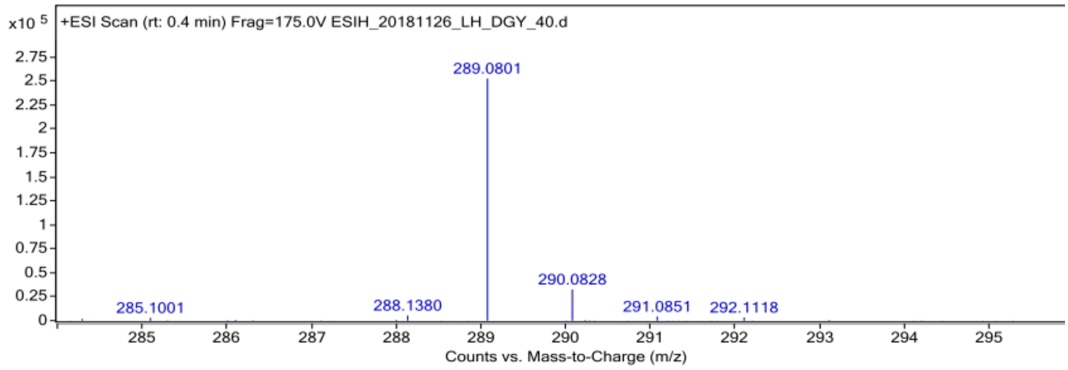

Formula Calculator Results

m/z	Calc m/z	Diff (mDa)	Diff (ppm)	Ion Formula	Ion
289.0801	289.0795	-0.6	-2.07	C12 H14 N2 Na O5	(M+Na)+

3m

**Data Filename**	ESIH_20181025_LH_DGY_09.d	**Sample Name**	B6-1025-DW39
**Sample Type**	Sample	**Position**	P1-A5
**Instrument Name**	Agilent G6520 Q-TOF	**Acq Method**	20160322_MS_ESIH_POS_1min.m
**Acquired Time**	10/25/2018 14:49:32	**IRM Calibration Status**	Success
**DA Method**	small molecular data analysis method.m	**Comment**	ESIH by ZZY

User Spectra

Fragmentor Voltage	Collision Energy	Ionization Mode
175	0	ESI

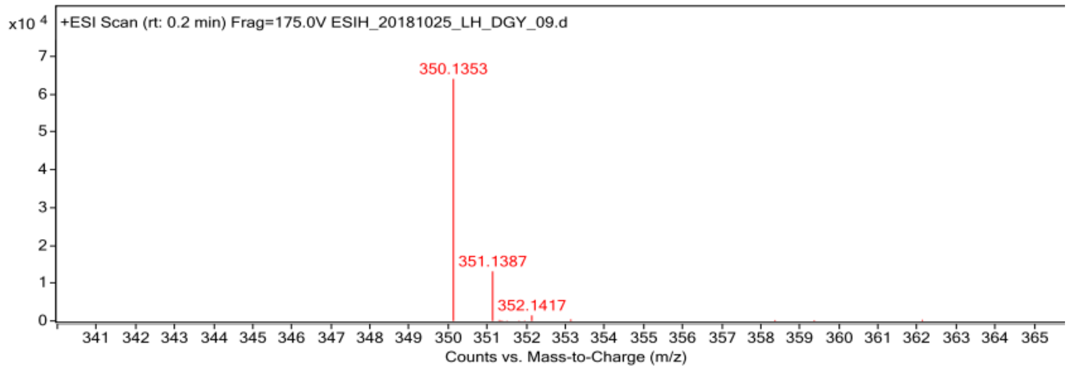

Formula Calculator Results

m/z	Calc m/z	Diff (mDa)	Diff (ppm)	Ion Formula	Ion
350.1353	350.1363	0.95	2.72	C19 H21 N Na O4	(M+Na)+

3n

**Data Filename**	ESIH_20181012_LH_DGY_07.d	**Sample Name**	B6-DW-34
**Sample Type**	Sample	**Position**	P1-B3
**Instrument Name**	Agilent G6520 Q-TOF	**Acq Method**	20160322_MS_ESIH_POS_1min.m
**Acquired Time**	10/12/2018 14:22:06	**IRM Calibration Status**	Success
**DA Method**	small molecular data analysis method.m	**Comment**	ESIH by ZZY

User Spectra

Fragmentor Voltage	Collision Energy	Ionization Mode
175	0	ESI

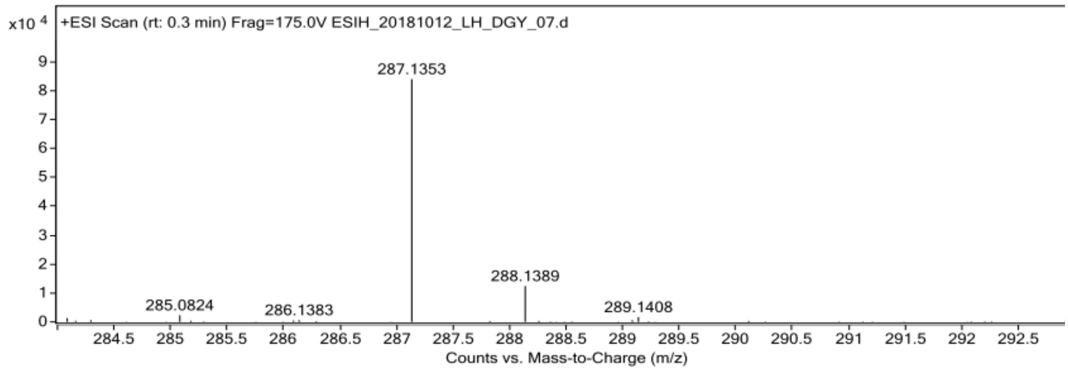

Formula Calculator Results

m/z	Calc m/z	Diff (mDa)	Diff (ppm)	Ion Formula	Ion
287.1353	287.1366	1.27	4.44	C14 H20 N2 Na O3	(M+Na)+

3o

**Data Filename**	ESIH_20181017_LH_DGY_07.d	**Sample Name**	B6-DW-19
**Sample Type**	Sample	**Position**	P1-B1
**Instrument Name**	Agilent G6520 Q-TOF	**Acq Method**	20160322_MS_ESIH_POS_1min.m
**Acquired Time**	10/17/2018 16:35:49	**IRM Calibration Status**	Success
**DA Method**	small molecular data analysis method.m	**Comment**	ESIH by ZZY

User Spectra

Fragmentor Voltage	Collision Energy	Ionization Mode
175	0	ESI

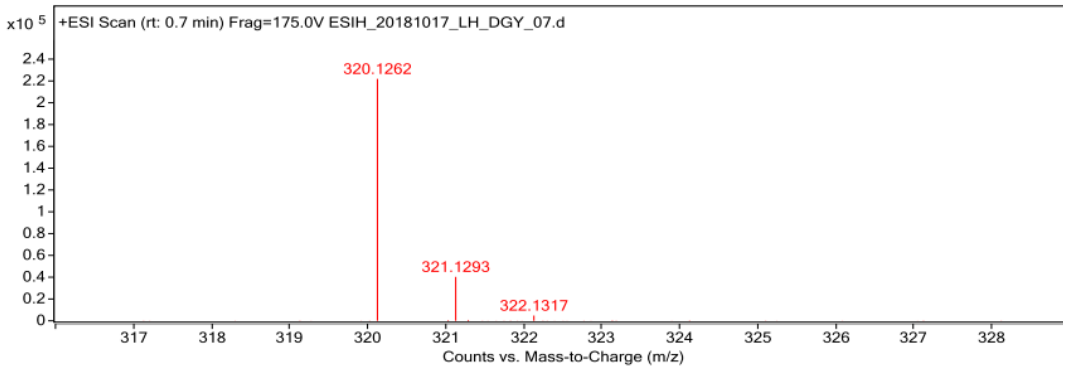

Formula Calculator Results

m/z	Calc m/z	Diff (mDa)	Diff (ppm)	Ion Formula	Ion
320.1262	320.1257	-0.46	-1.44	C18 H19 N Na O3	(M+Na)+

3p

**Data Filename**	ESIH_20180929_LH_DGY_18.d	**Sample Name**	B6-0929-DW16
**Sample Type**	Sample	**Position**	P1-A9
**Instrument Name**	Agilent G6520 Q-TOF	**Acq Method**	20160324_MS_ESIH_NEG_1min.m
**Acquired Time**	9/29/2018 15:05:36	**IRM Calibration Status**	Success
**DA Method**	small molecular data analysis method.m	**Comment**	ESIH by ZZY

User Spectra

Fragmentor Voltage	Collision Energy	Ionization Mode
175	0	ESI

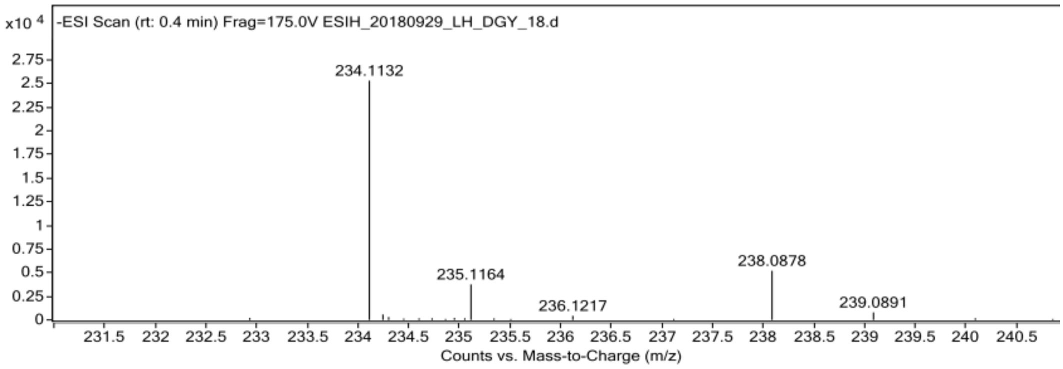

Formula Calculator Results

m/z	Calc m/z	Diff (mDa)	Diff (ppm)	Ion Formula	Ion
234.1132	234.1136	0.35	1.48	C13 H16 N O3	(M-H)-

3q

**Data Filename**	ESIH_20180929_LH_DGY_14.d	**Sample Name**	B6-0929-DW12
**Sample Type**	Sample	**Position**	P1-A5
**Instrument Name**	Agilent G6520 Q-TOF	**Acq Method**	20160324_MS_ESIH_NEG_1min.m
**Acquired Time**	9/29/2018 14:58:06	**IRM Calibration Status**	Success
**DA Method**	small molecular data analysis method.m	**Comment**	ESIH by ZZY

User Spectra

Fragmentor Voltage	Collision Energy	Ionization Mode
175	0	ESI

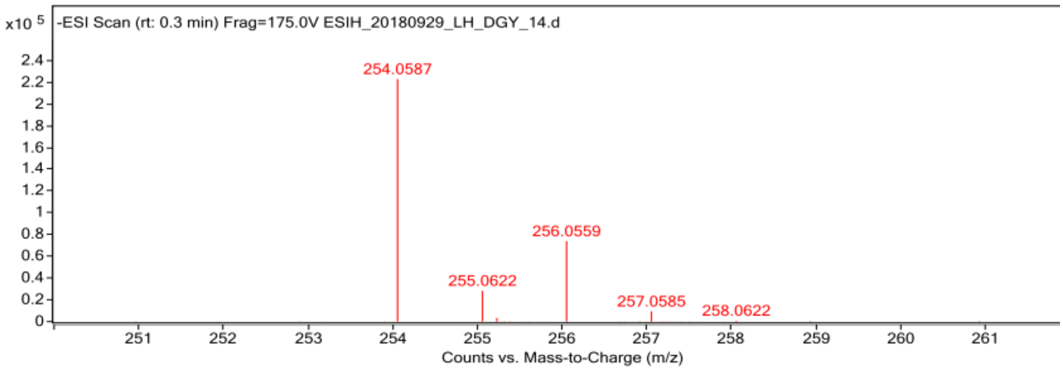

Formula Calculator Results

m/z	Calc m/z	Diff (mDa)	Diff (ppm)	Ion Formula	Ion
254.0587	254.0589	0.26	1.03	C12 H13 Cl N O3	(M-H)-

3r

**Data Filename**	ESIH_20180929_LH_DGY_16.d	**Sample Name**	B6-0929-DW14
**Sample Type**	Sample	**Position**	P1-A7
**Instrument Name**	Agilent G6520 Q-TOF	**Acq Method**	20160324_MS_ESIH_NEG_1min.m
**Acquired Time**	9/29/2018 15:01:53	**IRM Calibration Status**	Success
**DA Method**	small molecular data analysis method.m	**Comment**	ESIH by ZZY

User Spectra

Fragmentor Voltage	Collision Energy	Ionization Mode
175	0	ESI

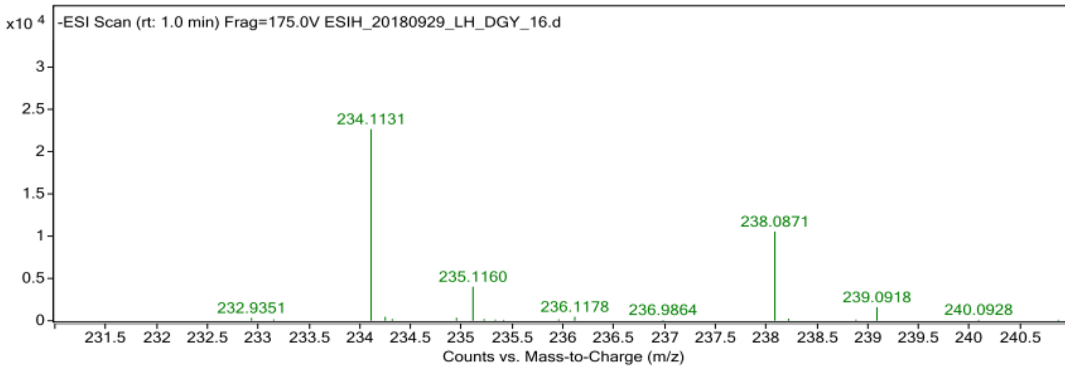

Formula Calculator Results

m/z	Calc m/z	Diff (mDa)	Diff (ppm)	Ion Formula	Ion
234.1131	234.1136	0.49	2.09	C13 H16 N O3	(M-H)-

3s

**Data Filename**	ESIH_20180929_LH_DGY_20.d	**Sample Name**	B6-0929-DW24
**Sample Type**	Sample	**Position**	P1-B2
**Instrument Name**	Agilent G6520 Q-TOF	**Acq Method**	20160324_MS_ESIH_NEG_1min.m
**Acquired Time**	9/29/2018 15:09:16	**IRM Calibration Status**	Success
**DA Method**	small molecular data analysis method.m	**Comment**	ESIH by ZZY

User Spectra

Fragmentor Voltage	Collision Energy	Ionization Mode
175	0	ESI

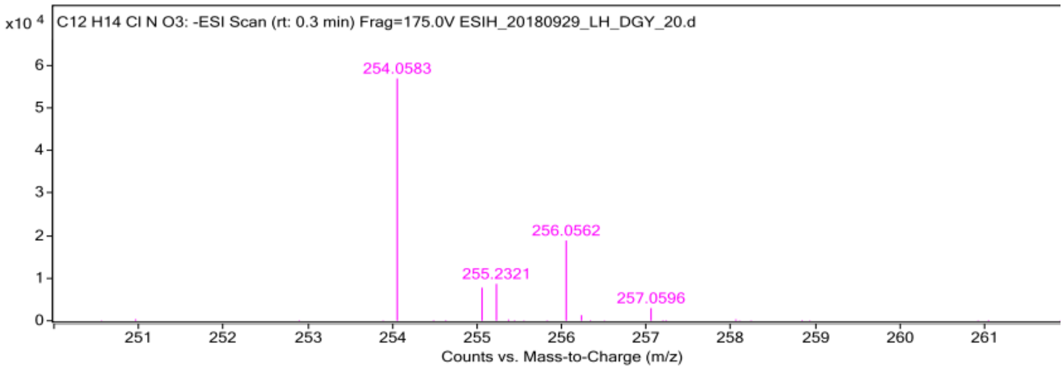

Formula Calculator Results

m/z	Calc m/z	Diff (mDa)	Diff (ppm)	Ion Formula	Ion
254.0583	254.0589	0.61	2.38	C12 H13 Cl N O3	(M-H)-

3t

**Data Filename**	ESIH_20180929_LH_DGY_19.d	**Sample Name**	B6-0929-DW23
**Sample Type**	Sample	**Position**	P1-B1
**Instrument Name**	Agilent G6520 Q-TOF	**Acq Method**	20160324_MS_ESIH_NEG_1min.m
**Acquired Time**	9/29/2018 15:07:26	**IRM Calibration Status**	Success
**DA Method**	small molecular data analysis method.m	**Comment**	ESIH by ZZY

User Spectra

Fragmentor Voltage	Collision Energy	Ionization Mode
175	0	ESI

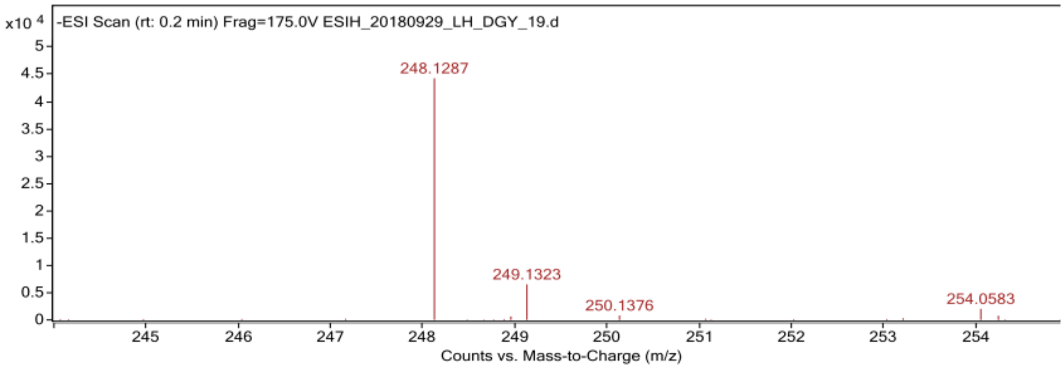

Formula Calculator Results

m/z	Calc m/z	Diff (mDa)	Diff (ppm)	Ion Formula	Ion
248.1287	248.1292	0.53	2.13	C14 H18 N O3	(M-H)-

3u

**Data Filename**	ESIH_20181024_LH_DGY_10.d	**Sample Name**	B6-DW-17
**Sample Type**	Sample	**Position**	P1-B8
**Instrument Name**	Agilent G6520 Q-TOF	**Acq Method**	20160322_MS_ESIH_POS_1min.m
**Acquired Time**	10/24/2018 19:44:50	**IRM Calibration Status**	Success
**DA Method**	small molecular data analysis method.m	**Comment**	ESIH by ZZY

User Spectra

Fragmentor Voltage	Collision Energy	Ionization Mode
175	0	ESI

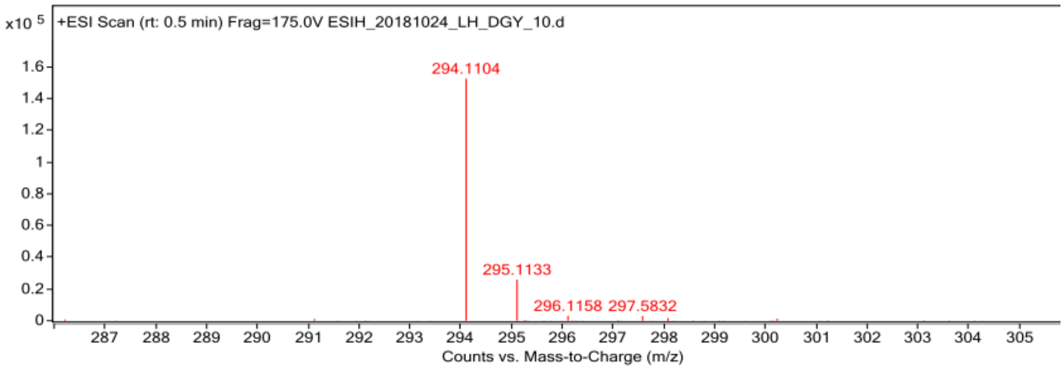

Formula Calculator Results

m/z	Calc m/z	Diff (mDa)	Diff (ppm)	Ion Formula	Ion
294.1104	294.1101	-0.3	-1.02	C16 H17 N Na O3	(M+Na)+

3v

**Data Filename**	ESIH_20180929_LH_DGY_17.d	**Sample Name**	B6-0929-DW15
**Sample Type**	Sample	**Position**	P1-A8
**Instrument Name**	Agilent G6520 Q-TOF	**Acq Method**	20160324_MS_ESIH_NEG_1min.m
**Acquired Time**	9/29/2018 15:03:43	**IRM Calibration Status**	Success
**DA Method**	small molecular data analysis method.m	**Comment**	ESIH by ZZY

User Spectra

Fragmentor Voltage	Collision Energy	Ionization Mode
175	0	ESI

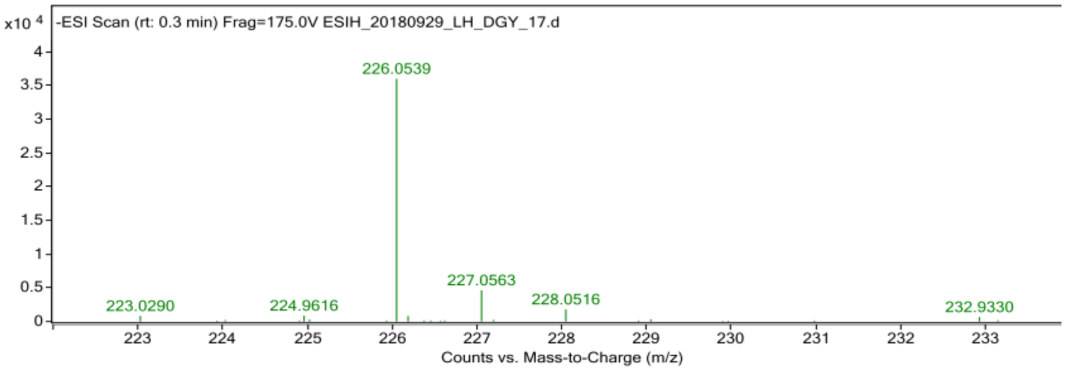

Formula Calculator Results

m/z	Calc m/z	Diff (mDa)	Diff (ppm)	Ion Formula	Ion
226.0539	226.0543	0.47	2.09	C10 H12 N O3 S	(M-H)-

6. MS Spectra of products.

3aa

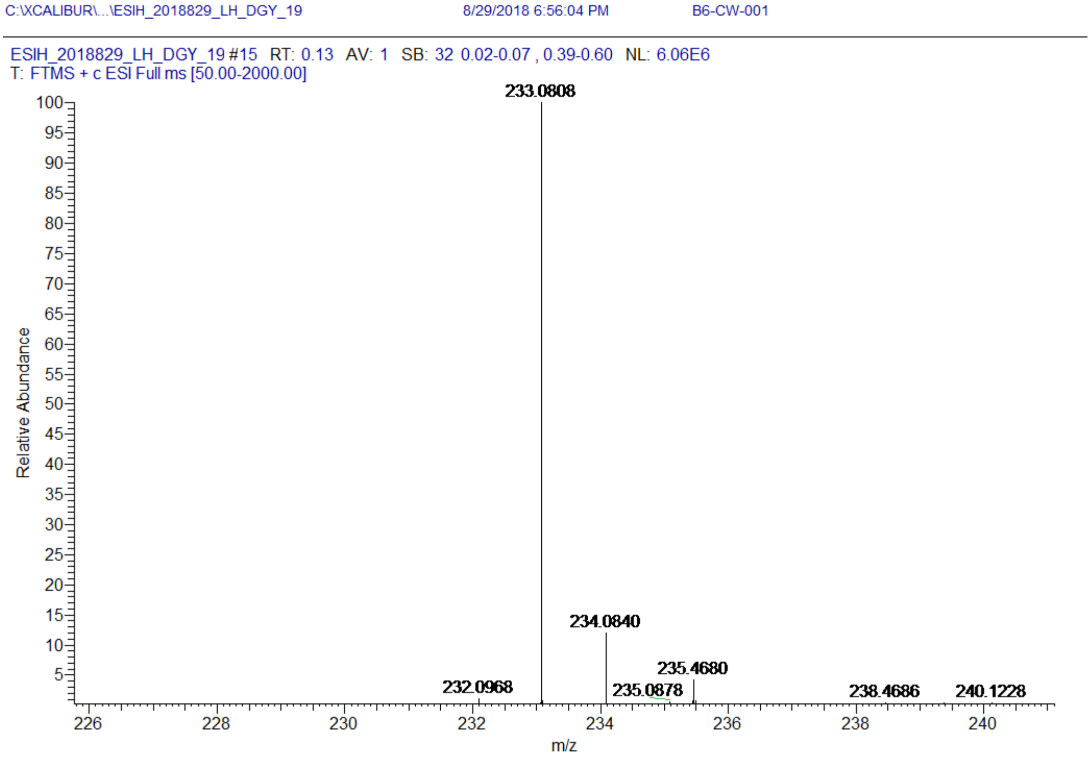

3ba

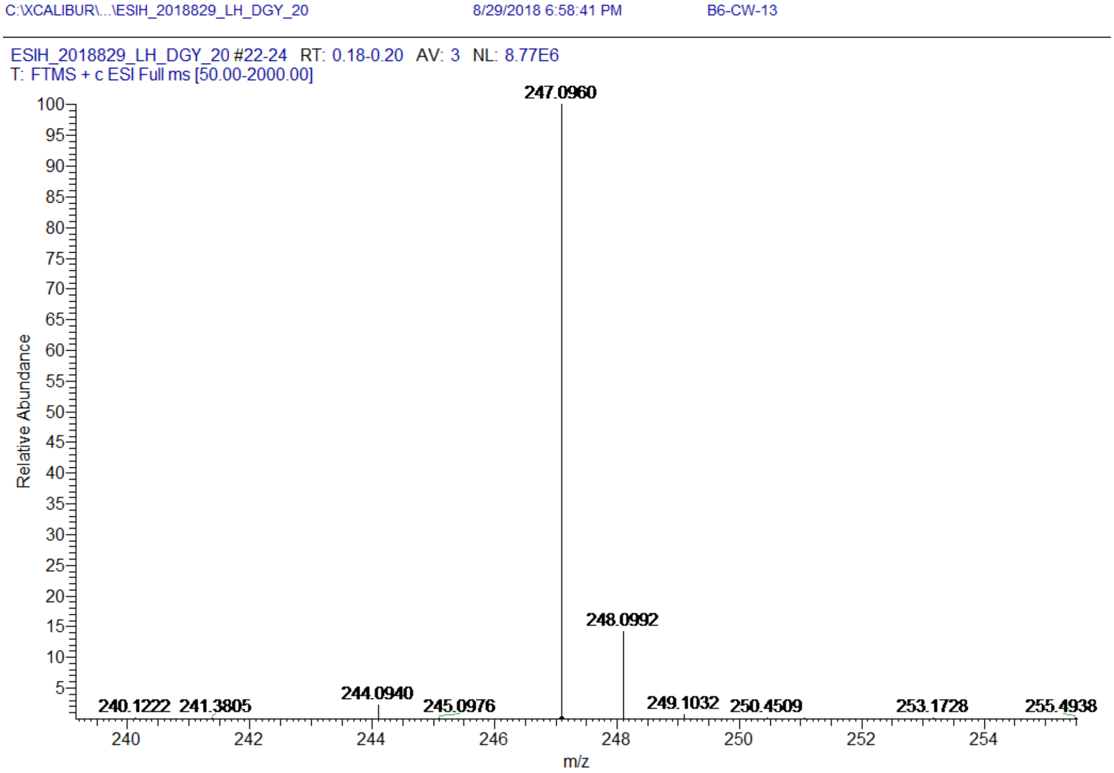

3ca

**Data Filename**	ESIH_20181017_LH_DGY_09.d	**Sample Name**	b6-CW-37
**Sample Type**	Sample	**Position**	P1-B3
**Instrument Name**	Agilent G6520 Q-TOF	**Acq Method**	20160322_MS_ESIH_POS_1min.m
**Acquired Time**	10/17/2018 16:39:28	**IRM Calibration Status**	Success
**DA Method**	small molecular data analysis method.m	**Comment**	ESIH by ZZY

User Spectra

Fragmentor Voltage	Collision Energy	Ionization Mode
175	0	ESI

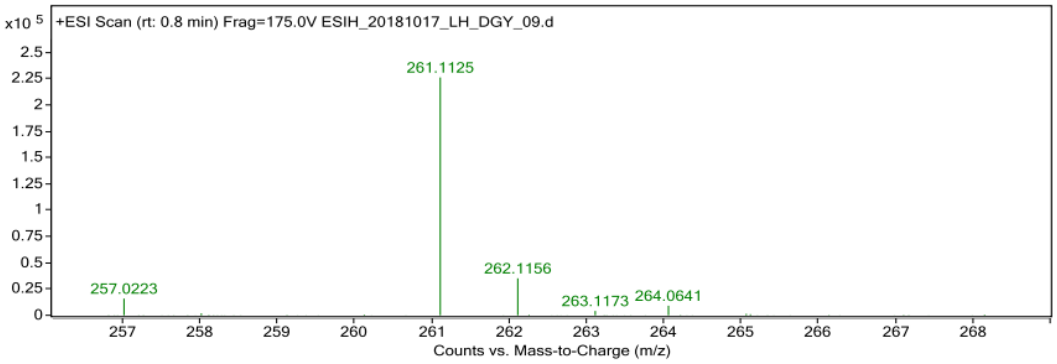

Formula Calculator Results

m/z	Calc m/z	Diff (mDa)	Diff (ppm)	Ion Formula	Ion
261.1125	261.1121	-0.4	-1.54	C15 H17 O4	(M+H)+

3da

**Data Filename**	ESIH_20181012_LH_DGY_06.d	**Sample Name**	B6-Cw-35
**Sample Type**	Sample	**Position**	P1-B2
**Instrument Name**	Agilent G6520 Q-TOF	**Acq Method**	20160322_MS_ESIH_POS_1min.m
**Acquired Time**	10/12/2018 14:20:16	**IRM Calibration Status**	Success
**DA Method**	small molecular data analysis method.m	**Comment**	ESIH by ZZY

User Spectra

Fragmentor Voltage	Collision Energy	Ionization Mode
175	0	ESI

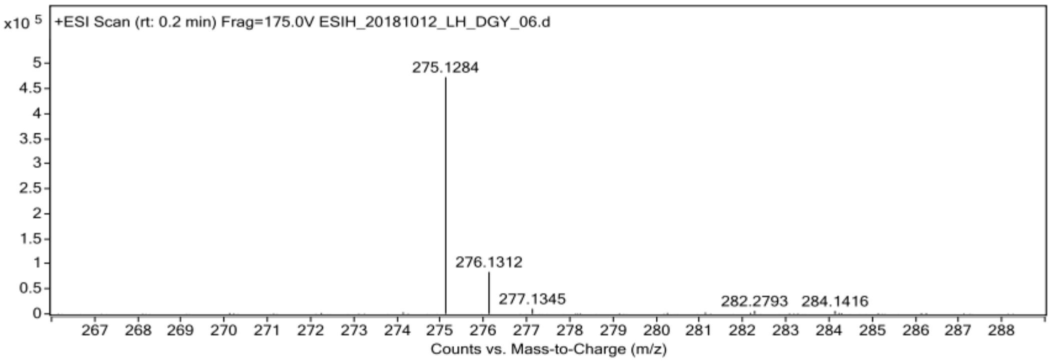

Formula Calculator Results

m/z	Calc m/z	Diff (mDa)	Diff (ppm)	Ion Formula	Ion
275.1284	275.1278	-0.63	-2.3	C16 H19 O4	(M+H)+

3ea

**Data Filename**	ESIH_20180927_LH_DGY_09.d	**Sample Name**	b6-0926-G28
**Sample Type**	Sample	**Position**	P1-A9
**Instrument Name**	Agilent G6520 Q-TOF	**Acq Method**	20160322_MS_ESIH_POS_1min.m
**Acquired Time**	9/27/2018 17:09:54	**IRM Calibration Status**	Success
**DA Method**	small molecular data analysis method.m	**Comment**	ESIH by ZZY

User Spectra

Fragmentor Voltage	Collision Energy	Ionization Mode
175	0	ESI

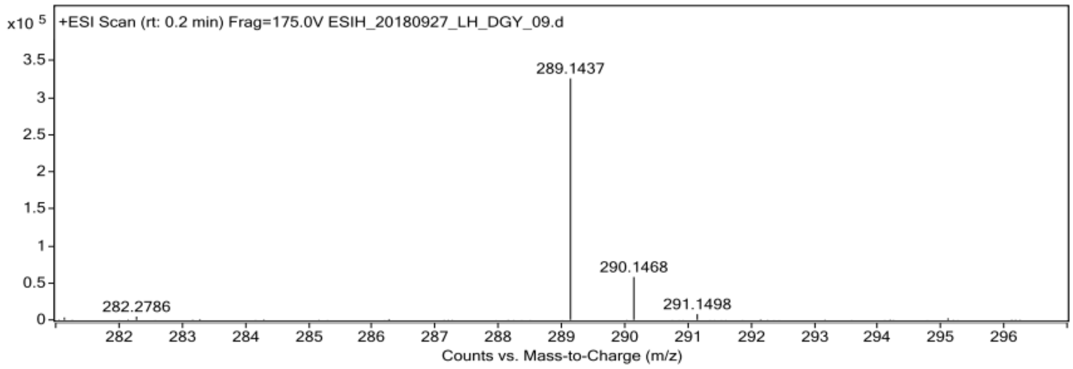

Formula Calculator Results

m/z	Calc m/z	Diff (mDa)	Diff (ppm)	Ion Formula	Ion
289.1437	289.1434	-0.22	-0.77	C17 H21 O4	(M+H)+

3fa

**Data Filename**	ESIH_20180809_LH_DGY_16.d	**Sample Name**	B6-GG-11
**Sample Type**	Sample	**Position**	P1-D2
**Instrument Name**	Agilent G6520 Q-TOF	**Acq Method**	20160322_MS_ESIH_POS_1min.m
**Acquired Time**	8/9/2018 17:40:08	**IRM Calibration Status**	Success
**DA Method**	small molecular data analysis method.m	**Comment**	ESIH by ZZY

User Spectra

Fragmentor Voltage	Collision Energy	Ionization Mode
110	0	ESI

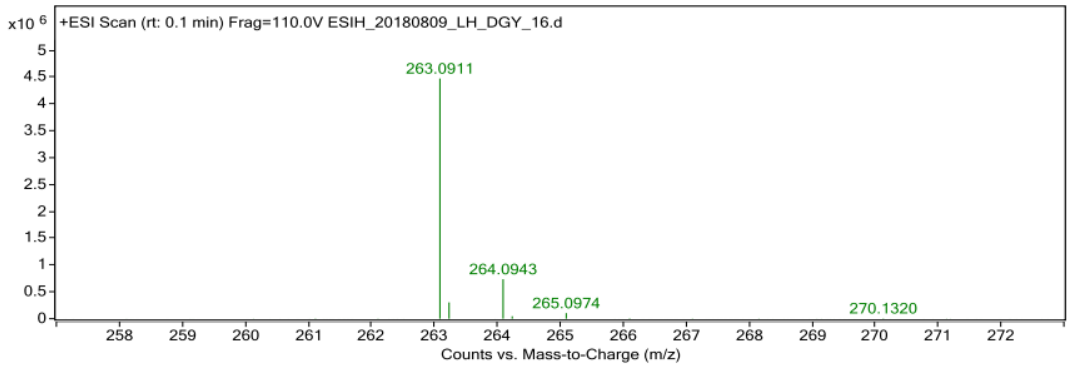

Formula Calculator Results

m/z	Calc m/z	Diff (mDa)	Diff (ppm)	Ion Formula	Ion
263.0911	263.0914	0.35	1.33	C14 H15 O5	(M+H)+

3ga

C:\XCALIBUR\...\ESIH_2018829_LH_DGY_18 8/29/2018 6:53:27 PM B6-CW-9

ESIH_2018829_LH_DGY_18 #23-31 RT: 0.19-0.26 AV: 9 NL: 1.07E5
T: FTMS + c ESI Full ms [50.00-2000.00]

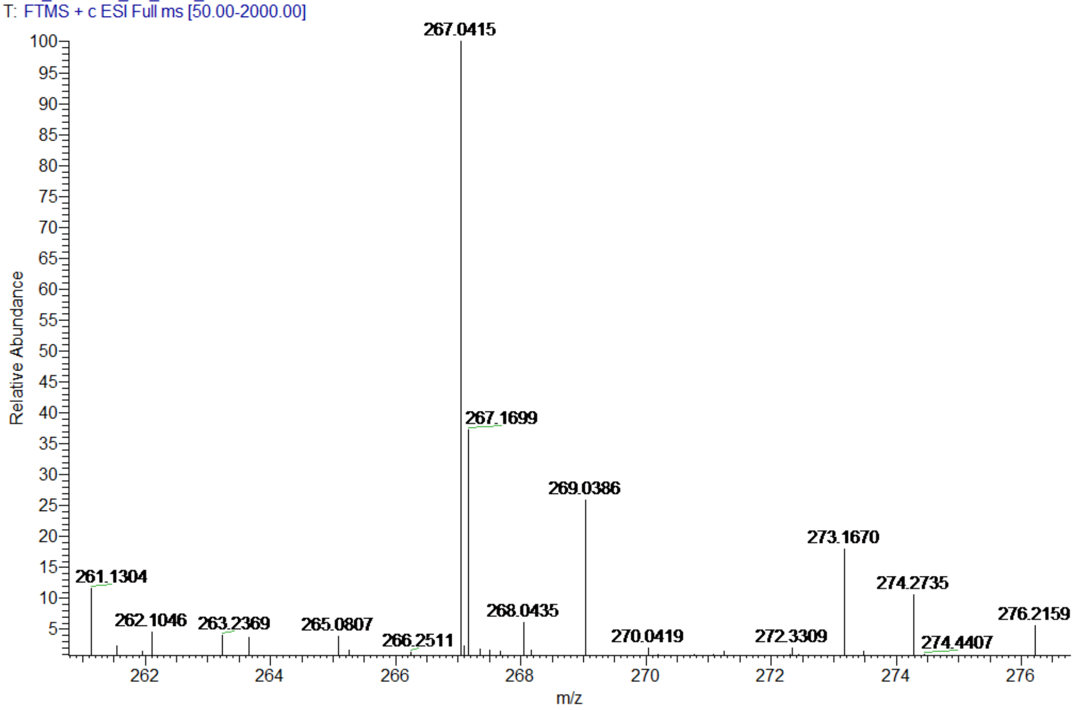

3ha

**Data Filename**	ESIH_20180927_LH_DGY_04.d	**Sample Name**	B6-0926-G5
**Sample Type**	Sample	**Position**	P1-A4
**Instrument Name**	Agilent G6520 Q-TOF	**Acq Method**	20160324_MS_ESIH_NEG_1min.m
**Acquired Time**	9/27/2018 14:56:09	**IRM Calibration Status**	Success
**DA Method**	small molecular data analysis method.m	**Comment**	ESIH by ZZY

User Spectra

Fragmentor Voltage	Collision Energy	Ionization Mode
175	0	ESI

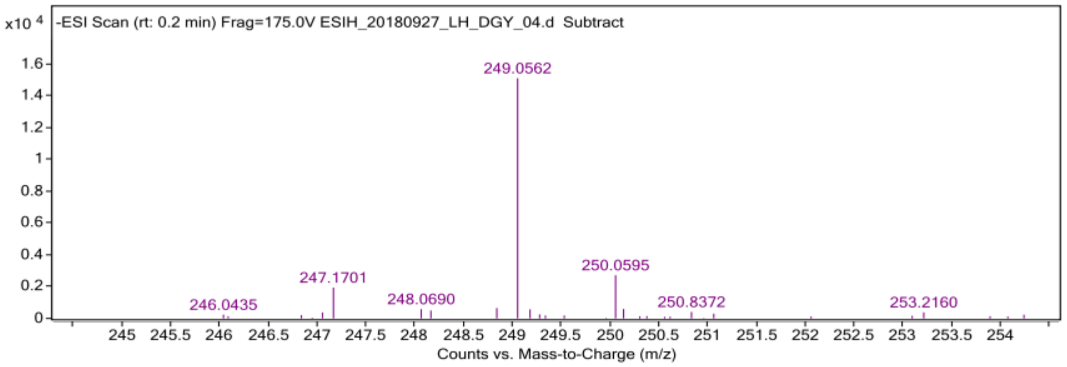

Formula Calculator Results

m/z	Calc m/z	Diff (mDa)	Diff (ppm)	Ion Formula	Ion
249.0562	249.0569	0.68	2.75	C13 H10 F O4	(M-H)-

3ia

**Data Filename**	ESIH_20180927_LH_DGY_05.d	**Sample Name**	B6-0926-G8
**Sample Type**	Sample	**Position**	P1-A5
**Instrument Name**	Agilent G6520 Q-TOF	**Acq Method**	20160322_MS_ESIH_POS_1min.m
**Acquired Time**	9/27/2018 15:51:04	**IRM Calibration Status**	Success
**DA Method**	small molecular data analysis method.m	**Comment**	ESIH by ZZY

User Spectra

Fragmentor Voltage	Collision Energy	Ionization Mode
175	0	ESI

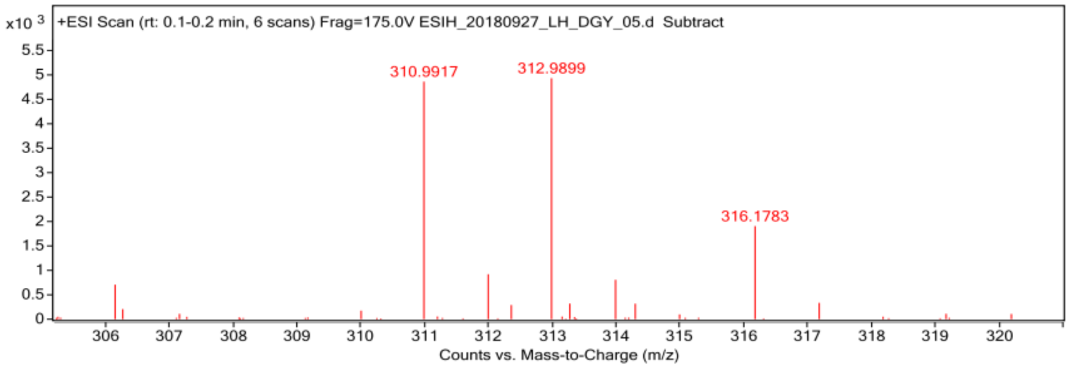

Formula Calculator Results

m/z	Calc m/z	Diff (mDa)	Diff (ppm)	Ion Formula	Ion
310.9917	310.9913	-0.39	-1.24	C13 H12 Br O4	(M+H)+

3ja

**Data Filename**	ESIH_20181025_LH_DGY_07.d	**Sample Name**	b6-1025-CW38
**Sample Type**	Sample	**Position**	P1-A3
**Instrument Name**	Agilent G6520 Q-TOF	**Acq Method**	20160322_MS_ESIH_POS_1min.m
**Acquired Time**	10/25/2018 14:45:51	**IRM Calibration Status**	Success
**DA Method**	small molecular data analysis method.m	**Comment**	ESIH by ZZY

User Spectra

Fragmentor Voltage	Collision Energy	Ionization Mode
175	0	ESI

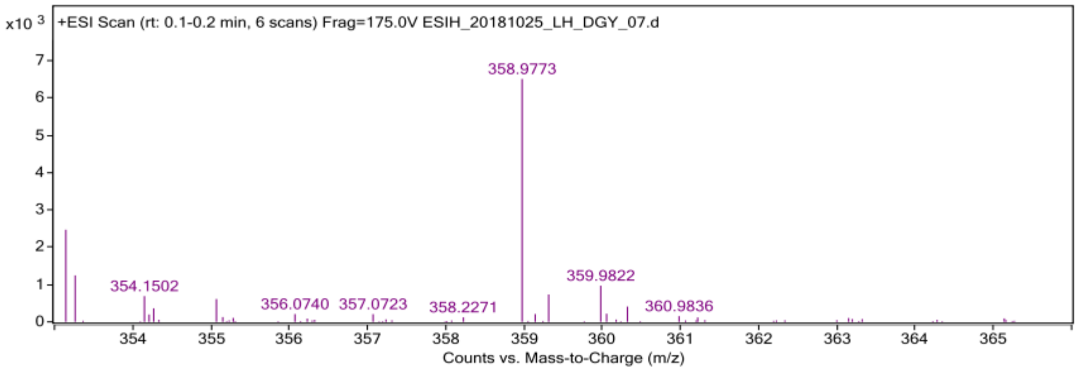

Formula Calculator Results

m/z	Calc m/z	Diff (mDa)	Diff (ppm)	Ion Formula	Ion
358.9773	358.9775	0.19	0.54	C13 H12 I O4	(M+H)+

3ka

**Data Filename**	ESIH_20181126_LH_DGY_38.d	**Sample Name**	B6-1126-CWCF3
**Sample Type**	Sample	**Position**	P2-E1
**Instrument Name**	Agilent G6520 Q-TOF	**Acq Method**	20160322_MS_ESIH_POS_1min.m
**Acquired Time**	11/26/2018 16:19:48	**IRM Calibration Status**	Success
**DA Method**	small molecular data analysis method.m	**Comment**	ESIH by ZZY

User Spectra

Fragmentor Voltage	Collision Energy	Ionization Mode
175	0	ESI

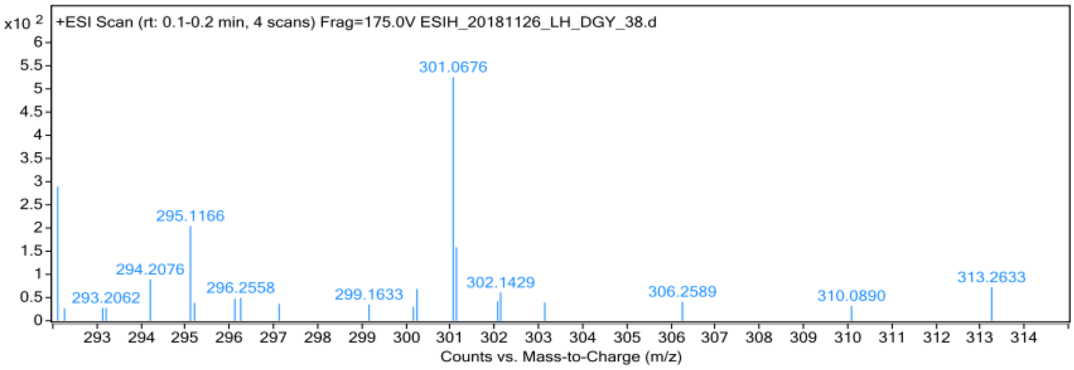

Formula Calculator Results

m/z	Calc m/z	Diff (mDa)	Diff (ppm)	Ion Formula	Ion
301.0676	301.0682	0.64	2.13	C14 H12 F3 O4	(M+H)+

3la

**Data Filename**	ESIH_20181220_LH_DGY_04.d	**Sample Name**	B6-1217-CWNO2
**Sample Type**	Sample	**Position**	P1-B1
**Instrument Name**	Agilent G6520 Q-TOF	**Acq Method**	20160324_MS_ESIH_NEG_1min.m
**Acquired Time**	12/20/2018 17:15:58	**IRM Calibration Status**	Success
**DA Method**	small molecular data analysis method.m	**Comment**	ESIH by ZZY

User Spectra

Fragmentor Voltage	Collision Energy	Ionization Mode
175	0	ESI

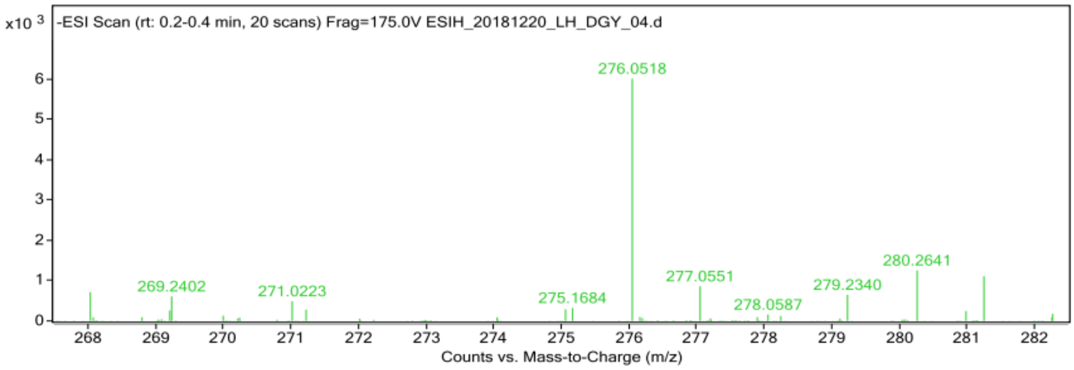

Formula Calculator Results

m/z	Calc m/z	Diff (mDa)	Diff (ppm)	Ion Formula	Ion
276.0518	276.0514	-0.43	-1.56	C13 H10 N O6	(M-H)-

3ma

**Data Filename**	ESIH_20181101_LH_DGY_02.d	**Sample Name**	B6-CW-39
**Sample Type**	Sample	**Position**	P1-A2
**Instrument Name**	Agilent G6520 Q-TOF	**Acq Method**	20160322_MS_ESIH_POS_1min.m
**Acquired Time**	11/1/2018 14:19:23	**IRM Calibration Status**	Success
**DA Method**	small molecular data analysis method.m	**Comment**	ESI_HR by ZZY

User Spectra

Fragmentor Voltage	Collision Energy	Ionization Mode
175	0	ESI

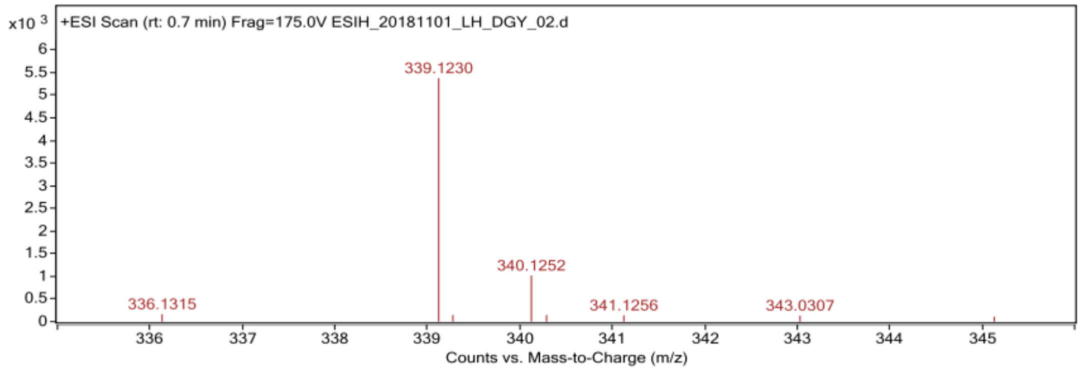

Formula Calculator Results

m/z	Calc m/z	Diff (mDa)	Diff (ppm)	Ion Formula	Ion
339.123	339.1227	-0.27	-0.79	C20 H19 O5	(M+H)+

3na

**Data Filename**	ESIH_20180929_LH_DGY_22.d	**Sample Name**	B6-0929-CW34
**Sample Type**	Sample	**Position**	P1-B4
**Instrument Name**	Agilent G6520 Q-TOF	**Acq Method**	20160322_MS_ESIH_POS_1min.m
**Acquired Time**	9/29/2018 15:13:00	**IRM Calibration Status**	Success
**DA Method**	small molecular data analysis method.m	**Comment**	ESIH by ZZY

User Spectra

Fragmentor Voltage	Collision Energy	Ionization Mode
175	0	ESI

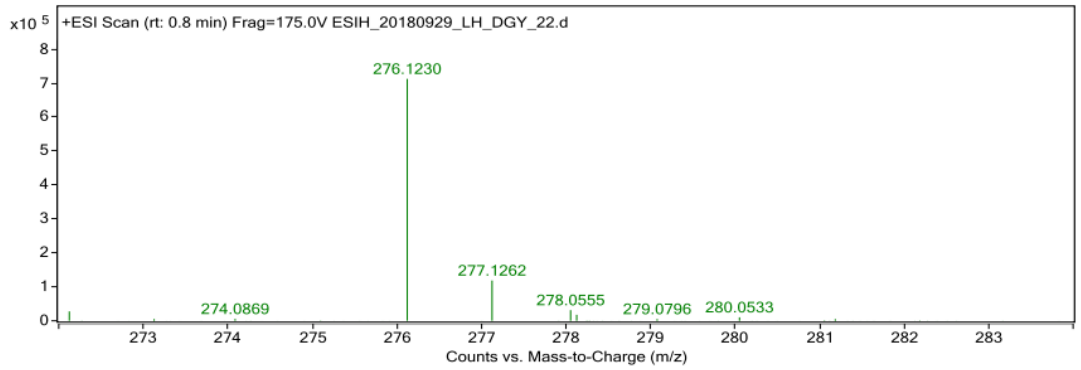

Formula Calculator Results

m/z	Calc m/z	Diff (mDa)	Diff (ppm)	Ion Formula	Ion
276.123	276.123	0.06	0.22	C15 H18 N O4	(M+H)+

3oa

**Data Filename**	ESIH_20180809_LH_DGY_11.d	**Sample Name**	B6-GG-19
**Sample Type**	Sample	**Position**	P1-C6
**Instrument Name**	Agilent G6520 Q-TOF	**Acq Method**	20160322_MS_ESIH_POS_1min.m
**Acquired Time**	8/9/2018 17:30:54	**IRM Calibration Status**	Success
**DA Method**	small molecular data analysis method.m	**Comment**	ESIH by ZZY

User Spectra

Fragmentor Voltage	Collision Energy	Ionization Mode
110	0	ESI

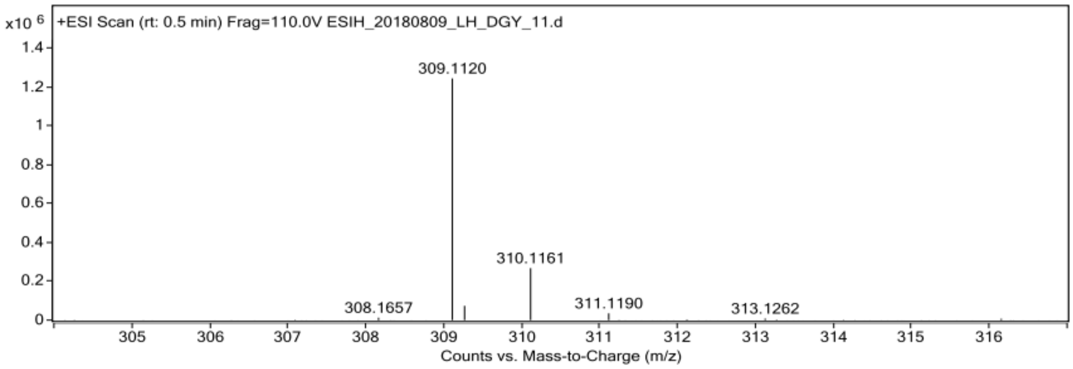

Formula Calculator Results

m/z	Calc m/z	Diff (mDa)	Diff (ppm)	Ion Formula	Ion
309.112	309.1121	0.16	0.53	C19 H17 O4	(M+H)+

3pa

**Data Filename**	ESIH_20180809_LH_DGY_13.d	**Sample Name**	B6-GG-16
**Sample Type**	Sample	**Position**	P1-C8
**Instrument Name**	Agilent G6520 Q-TOF	**Acq Method**	20160322_MS_ESIH_POS_1min.m
**Acquired Time**	8/9/2018 17:34:39	**IRM Calibration Status**	Success
**DA Method**	small molecular data analysis method.m	**Comment**	ESIH by ZZY

User Spectra

Fragmentor Voltage	Collision Energy	Ionization Mode
110	0	ESI

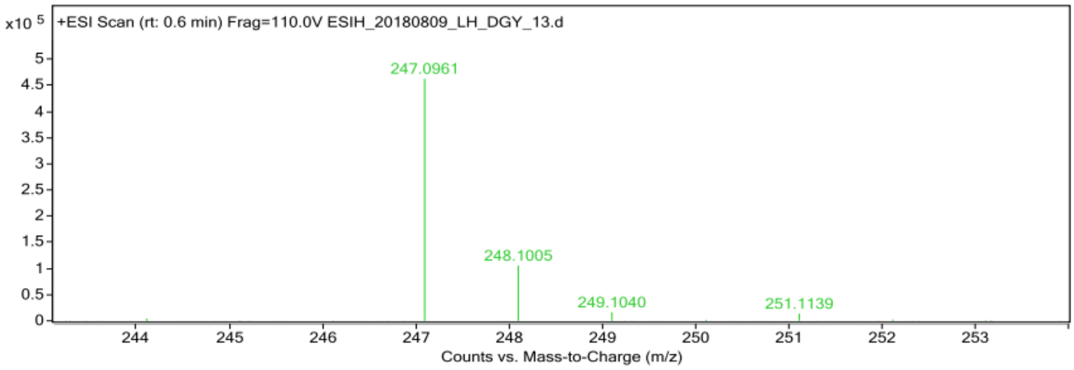

Formula Calculator Results

m/z	Calc m/z	Diff (mDa)	Diff (ppm)	Ion Formula	Ion
247.0961	247.0965	0.38	1.54	C14 H15 O4	(M+H)+

3qa

**Data Filename**	ESIH_20181025_LH_DGY_05.d	**Sample Name**	B6-1025-CW12
**Sample Type**	Sample	**Position**	P1-A1
**Instrument Name**	Agilent G6520 Q-TOF	**Acq Method**	20160322_MS_ESIH_POS_1min.m
**Acquired Time**	10/25/2018 14:42:14	**IRM Calibration Status**	Success
**DA Method**	small molecular data analysis method.m	**Comment**	ESIH by ZZY

User Spectra

Fragmentor Voltage	Collision Energy	Ionization Mode
175	0	ESI

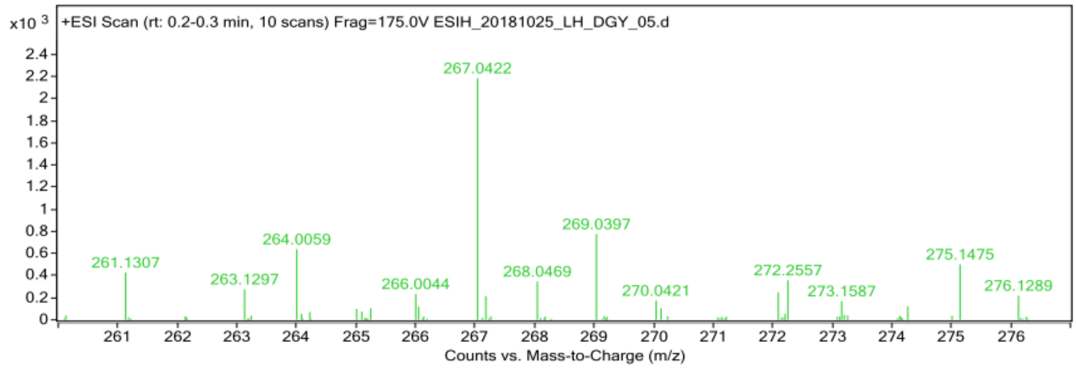

Formula Calculator Results

m/z	Calc m/z	Diff (mDa)	Diff (ppm)	Ion Formula	Ion
267.0422	267.0419	-0.39	-1.44	C13 H12 Cl O4	(M+H)+

3ra

**Data Filename**	ESIH_20180809_LH_DGY_15.d	**Sample Name**	B6-GG-14
**Sample Type**	Sample	**Position**	P1-D1
**Instrument Name**	Agilent G6520 Q-TOF	**Acq Method**	20160322_MS_ESIH_POS_1min.m
**Acquired Time**	8/9/2018 17:38:19	**IRM Calibration Status**	Success
**DA Method**	small molecular data analysis method.m	**Comment**	ESIH by ZZY

User Spectra

Fragmentor Voltage	Collision Energy	Ionization Mode
110	0	ESI

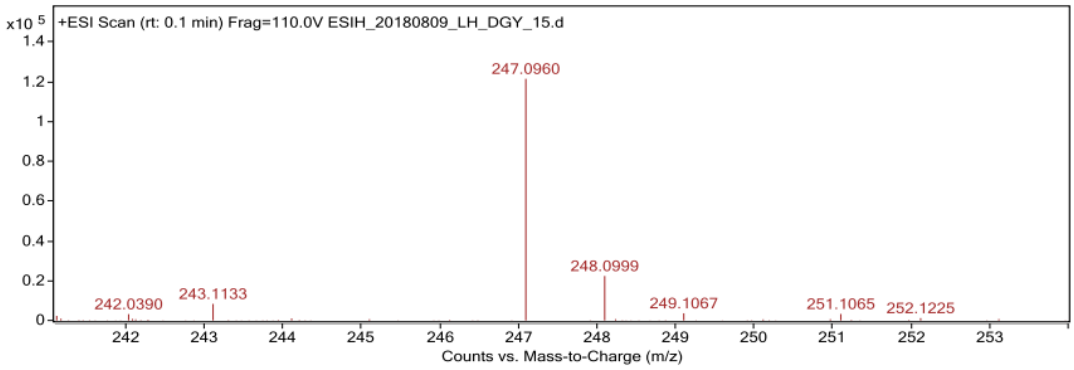

Formula Calculator Results

m/z	Calc m/z	Diff (mDa)	Diff (ppm)	Ion Formula	Ion
247.096	247.0965	0.48	1.93	C14 H15 O4	(M+H)+

3sa

Data Filename	ESIH_20180927_LH_DGY_07.d	Sample Name	B6-0926-G24
Sample Type	Sample	Position	P1-A7
Instrument Name	Agilent G6520 Q-TOF	Acq Method	20160322_MS_ESIH_POS_1min.m
Acquired Time	9/27/2018 17:06:13	IRM Calibration Status	Success
DA Method	small molecular data analysis method.m	Comment	ESIH by ZZY

User Spectra

Fragmentor Voltage	Collision Energy	Ionization Mode
175	0	ESI

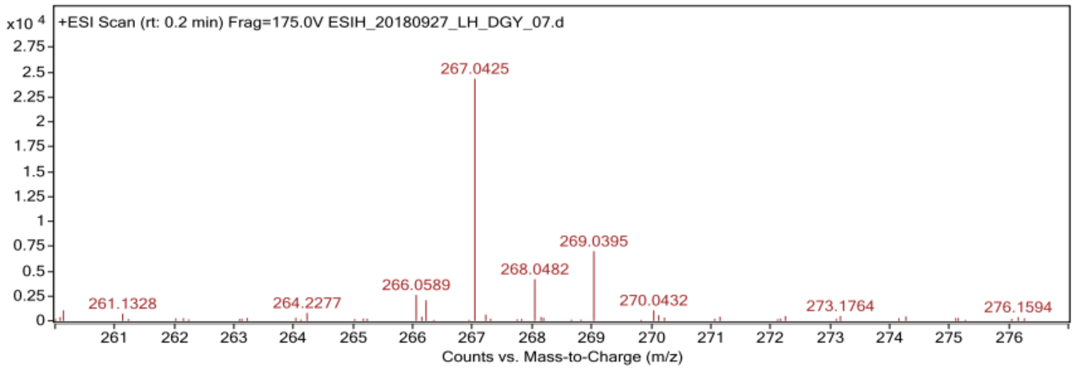

Formula Calculator Results

m/z	Calc m/z	Diff (mDa)	Diff (ppm)	Ion Formula	Ion
267.0425	267.0419	-0.68	-2.53	C13 H12 Cl O4	(M+H)+

3ta

Data Filename	ESIH_20180927_LH_DGY_06.d	Sample Name	B6-0926-G23
Sample Type	Sample	Position	P1-A6
Instrument Name	Agilent G6520 Q-TOF	Acq Method	20160322_MS_ESIH_POS_1min.m
Acquired Time	9/27/2018 17:04:19	IRM Calibration Status	Success
DA Method	small molecular data analysis method.m	Comment	ESIH by ZZY

User Spectra

Fragmentor Voltage	Collision Energy	Ionization Mode
175	0	ESI

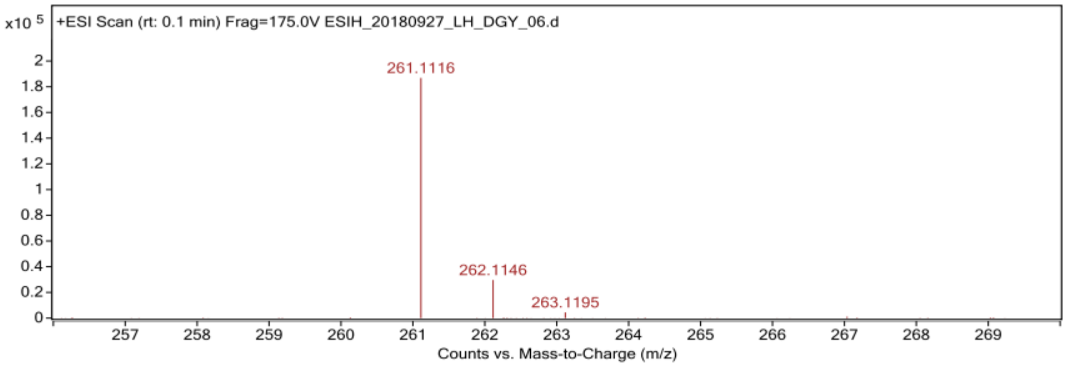

Formula Calculator Results

m/z	Calc m/z	Diff (mDa)	Diff (ppm)	Ion Formula	Ion
261.1116	261.1121	0.52	2	C15 H17 O4	(M+H)+

3ua

**Data Filename**	ESIH_20181024_LH_DGY_07.d	**Sample Name**	B6-1024-CW17
**Sample Type**	Sample	**Position**	P1-B5
**Instrument Name**	Agilent G6520 Q-TOF	**Acq Method**	20160322_MS_ESIH_POS_1min.m
**Acquired Time**	10/24/2018 19:39:20	**IRM Calibration Status**	Success
**DA Method**	small molecular data analysis method.m	**Comment**	ESIH by ZZY

User Spectra

**Fragmentor Voltage**	**Collision Energy**	**Ionization Mode**
175	0	ESI

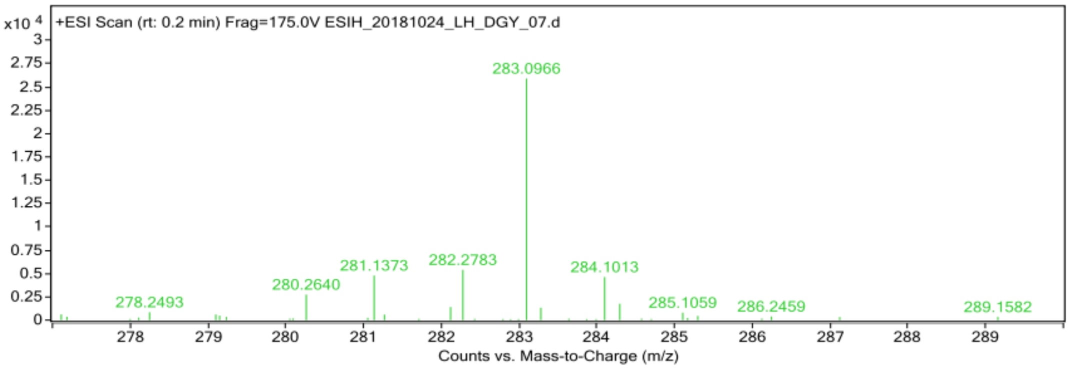

Formula Calculator Results

m/z	Calc m/z	Diff (mDa)	Diff (ppm)	Ion Formula	Ion
283.0966	283.0965	-0.09	-0.33	C17 H15 O4	(M+H)+

3va

**Data Filename**	ESIH_20180809_LH_DGY_14.d	**Sample Name**	B6-GG-15
**Sample Type**	Sample	**Position**	P1-C9
**Instrument Name**	Agilent G6520 Q-TOF	**Acq Method**	20160322_MS_ESIH_POS_1min.m
**Acquired Time**	8/9/2018 17:36:30	**IRM Calibration Status**	Success
**DA Method**	small molecular data analysis method.m	**Comment**	ESIH by ZZY

User Spectra

**Fragmentor Voltage**	**Collision Energy**	**Ionization Mode**
110	0	ESI

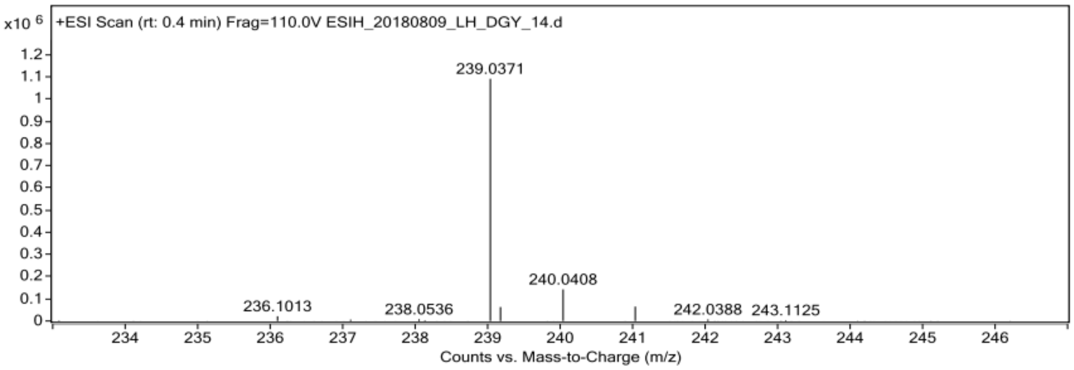

Formula Calculator Results

m/z	Calc m/z	Diff (mDa)	Diff (ppm)	Ion Formula	Ion
239.0371	239.0373	0.16	0.69	C11 H11 O4 S	(M+H)+

3ab

**Data Filename**	ESIH_20180809_LH_DGY_10.d	**Sample Name**	B6-GG-20
**Sample Type**	Sample	**Position**	P1-C5
**Instrument Name**	Agilent G6520 Q-TOF	**Acq Method**	20160322_MS_ESIH_POS_1min.m
**Acquired Time**	8/9/2018 17:29:03	**IRM Calibration Status**	Success
**DA Method**	small molecular data analysis method.m	**Comment**	ESIH by ZZY

User Spectra

Fragmentor Voltage	Collision Energy	Ionization Mode
110	0	ESI

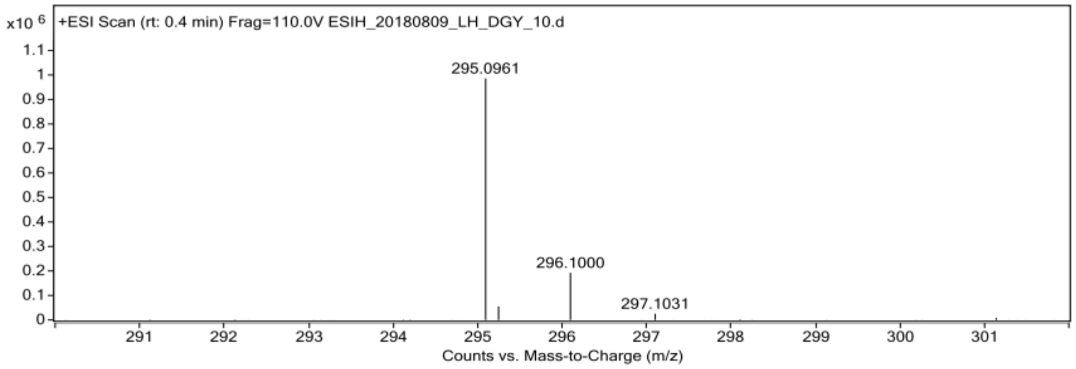

Formula Calculator Results

m/z	Calc m/z	Diff (mDa)	Diff (ppm)	Ion Formula	Ion
295.0961	295.0965	0.42	1.41	C18 H15 O4	(M+H)+

3ac

**Data Filename**	ESIH_20180809_LH_DGY_19.d	**Sample Name**	B6-GG-3
**Sample Type**	Sample	**Position**	P1-D5
**Instrument Name**	Agilent G6520 Q-TOF	**Acq Method**	20160322_MS_ESIH_POS_1min.m
**Acquired Time**	8/9/2018 17:45:38	**IRM Calibration Status**	Success
**DA Method**	small molecular data analysis method.m	**Comment**	ESIH by ZZY

User Spectra

Fragmentor Voltage	Collision Energy	Ionization Mode
110	0	ESI

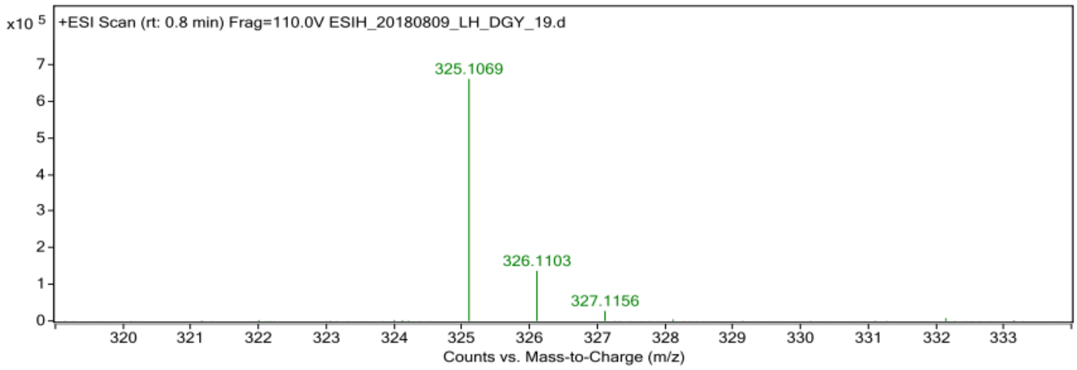

Formula Calculator Results

m/z	Calc m/z	Diff (mDa)	Diff (ppm)	Ion Formula	Ion
325.1069	325.1071	0.12	0.37	C19 H17 O5	(M+H)+

3ad

**Data Filename**	ESIH_20180929_LH_DGY_26.d	**Sample Name**	B6-0929-CW6
**Sample Type**	Sample	**Position**	P1-B8
**Instrument Name**	Agilent G6520 Q-TOF	**Acq Method**	20160322_MS_ESIH_POS_1min.m
**Acquired Time**	9/29/2018 17:20:30	**IRM Calibration Status**	Success
**DA Method**	small molecular data analysis method.m	**Comment**	ESIH by ZZY

User Spectra

Fragmentor Voltage	Collision Energy	Ionization Mode
175	0	ESI

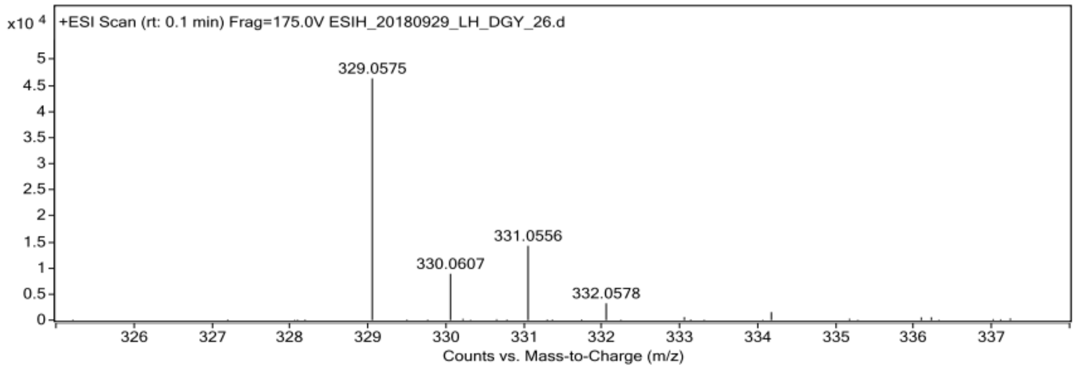

Formula Calculator Results

m/z	Calc m/z	Diff (mDa)	Diff (ppm)	Ion Formula	Ion
329.0575	329.0575	0	-0.01	C18 H14 Cl O4	(M+H)+

3ae

**Data Filename**	ESIH_20180929_LH_DGY_25.d	**Sample Name**	B6-0929-CW7
**Sample Type**	Sample	**Position**	P1-B7
**Instrument Name**	Agilent G6520 Q-TOF	**Acq Method**	20160322_MS_ESIH_POS_1min.m
**Acquired Time**	9/29/2018 15:58:43	**IRM Calibration Status**	Success
**DA Method**	small molecular data analysis method.m	**Comment**	ESIH by ZZY

User Spectra

Fragmentor Voltage	Collision Energy	Ionization Mode
175	0	ESI

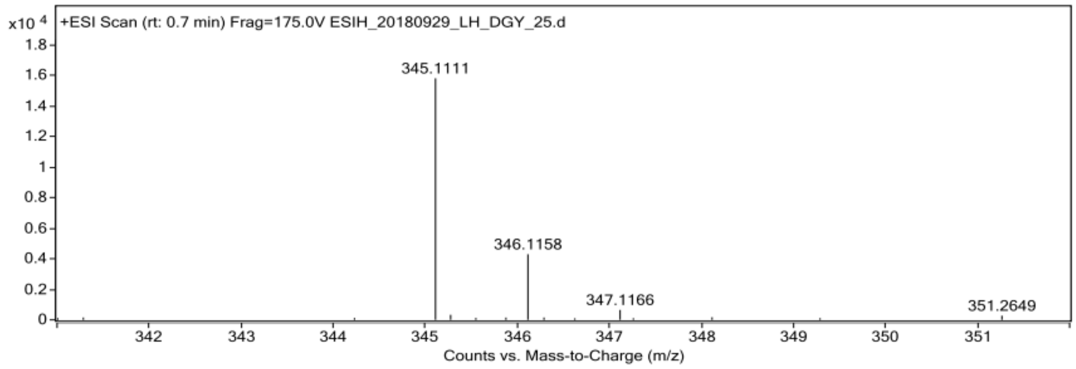

Formula Calculator Results

m/z	Calc m/z	Diff (mDa)	Diff (ppm)	Ion Formula	Ion
345.1111	345.1121	1.01	2.94	C22 H17 O4	(M+H)+

3af

**Data Filename**	ESIH_20180809_LH_DGY_18.d	**Sample Name**	B6-GG-1
**Sample Type**	Sample	**Position**	P1-D4
**Instrument Name**	Agilent G6520 Q-TOF	**Acq Method**	20160322_MS_ESIH_POS_1min.m
**Acquired Time**	8/9/2018 17:43:48	**IRM Calibration Status**	Success
**DA Method**	small molecular data analysis method.m	**Comment**	ESIH by ZZY

User Spectra

Fragmentor Voltage	Collision Energy	Ionization Mode
110	0	ESI

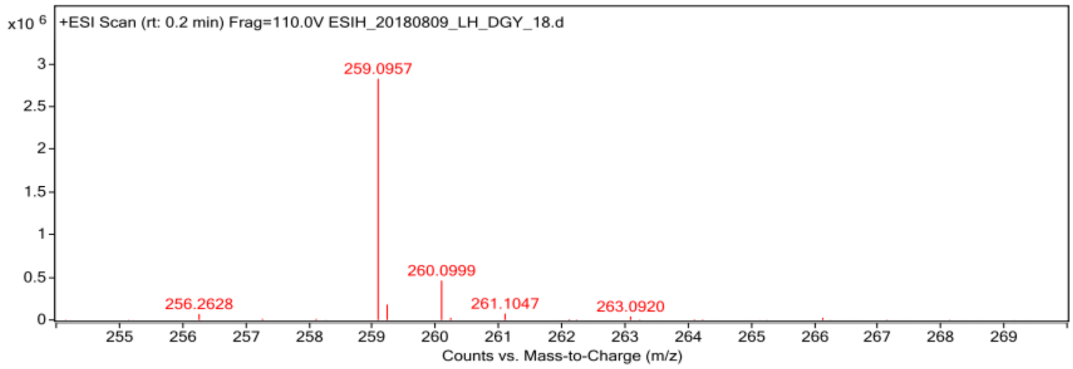

Formula Calculator Results

m/z	Calc m/z	Diff (mDa)	Diff (ppm)	Ion Formula	Ion
259.0957	259.0965	0.77	2.99	C15 H15 O4	(M+H)+

3ag

**Data Filename**	ESIH_20180927_LH_DGY_10.d	**Sample Name**	B6-0926-G29
**Sample Type**	Sample	**Position**	P1-B1
**Instrument Name**	Agilent G6520 Q-TOF	**Acq Method**	20160322_MS_ESIH_POS_1min.m
**Acquired Time**	9/27/2018 17:11:44	**IRM Calibration Status**	Success
**DA Method**	small molecular data analysis method.m	**Comment**	ESIH by ZZY

User Spectra

Fragmentor Voltage	Collision Energy	Ionization Mode
175	0	ESI

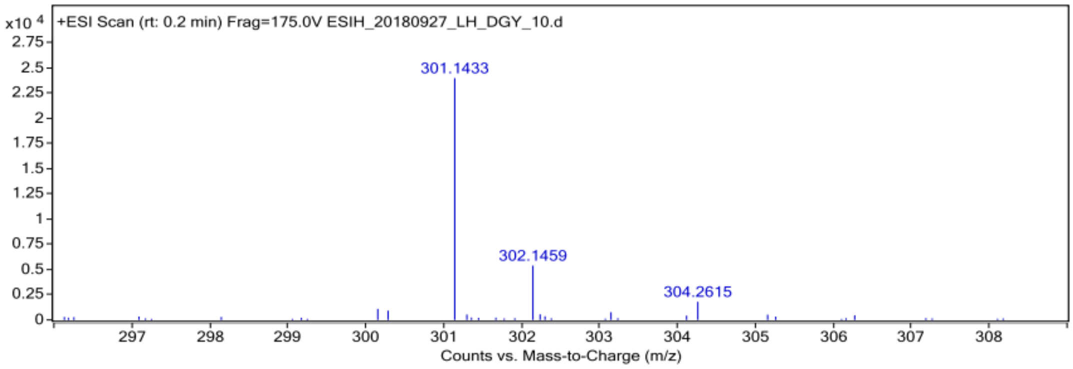

Formula Calculator Results

m/z	Calc m/z	Diff (mDa)	Diff (ppm)	Ion Formula	Ion
301.1433	301.1434	0.11	0.36	C18 H21 O4	(M+H)+

3ah

**Data Filename**	ESIH_20180927_LH_DGY_03.d	**Sample Name**	B6-0926-G4
**Sample Type**	Sample	**Position**	P1-A3
**Instrument Name**	Agilent G6520 Q-TOF	**Acq Method**	20160324_MS_ESIH_NEG_1min.m
**Acquired Time**	9/27/2018 14:54:19	**IRM Calibration Status**	Success
**DA Method**	small molecular data analysis method.m	**Comment**	ESIH by ZZY

User Spectra

Fragmentor Voltage	Collision Energy	Ionization Mode
175	0	ESI

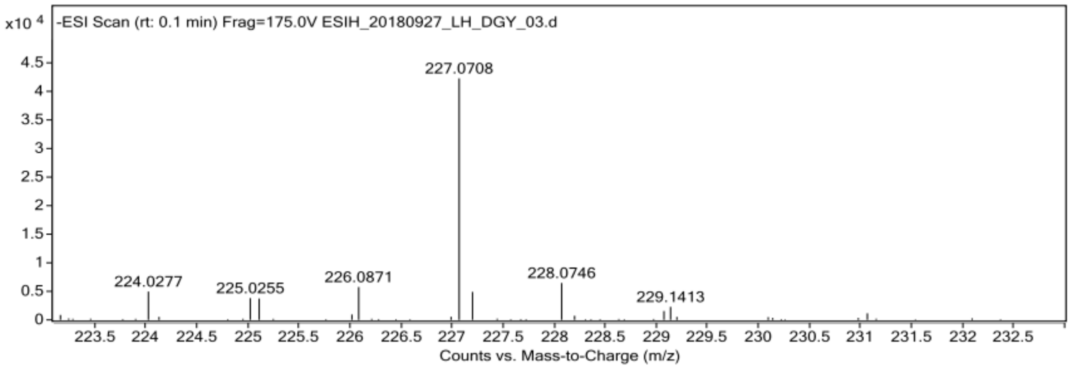

Formula Calculator Results

m/z	Calc m/z	Diff (mDa)	Diff (ppm)	Ion Formula	Ion
227.0708	227.0714	0.53	2.33	C14 H11 O3	(M-H)-

3ai

**Data Filename**	ESIH_20180809_LH_DGY_12.d	**Sample Name**	B6-GG-18
**Sample Type**	Sample	**Position**	P1-C7
**Instrument Name**	Agilent G6520 Q-TOF	**Acq Method**	20160322_MS_ESIH_POS_1min.m
**Acquired Time**	8/9/2018 17:32:48	**IRM Calibration Status**	Success
**DA Method**	small molecular data analysis method.m	**Comment**	ESIH by ZZY

User Spectra

Fragmentor Voltage	Collision Energy	Ionization Mode
110	0	ESI

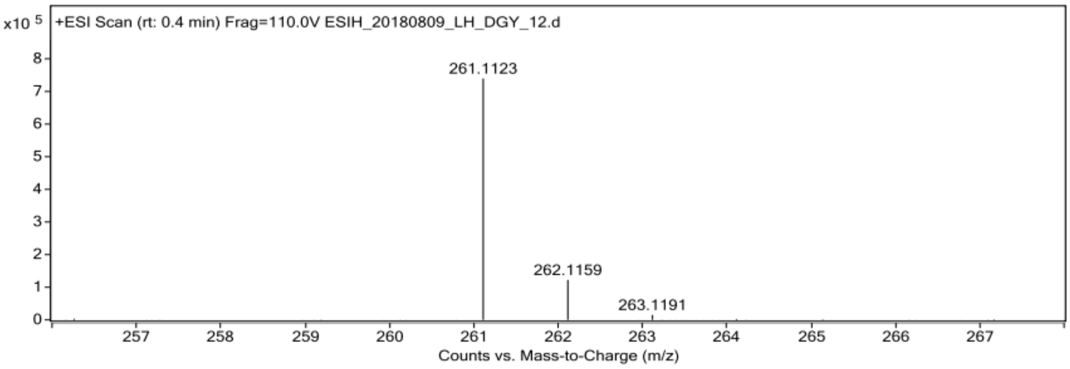

Formula Calculator Results

m/z	Calc m/z	Diff (mDa)	Diff (ppm)	Ion Formula	Ion
261.1123	261.1121	-0.14	-0.55	C15 H17 O4	(M+H)+

3aj

**Data Filename**	ESIH_20181025_LH_DGY_06.d	**Sample Name**	B6-1025-CW33
**Sample Type**	Sample	**Position**	P1-A2
**Instrument Name**	Agilent G6520 Q-TOF	**Acq Method**	20160322_MS_ESIH_POS_1min.m
**Acquired Time**	10/25/2018 14:44:02	**IRM Calibration Status**	Success
**DA Method**	small molecular data analysis method.m	**Comment**	ESIH by ZZY

User Spectra

Fragmentor Voltage	Collision Energy	Ionization Mode
175	0	ESI

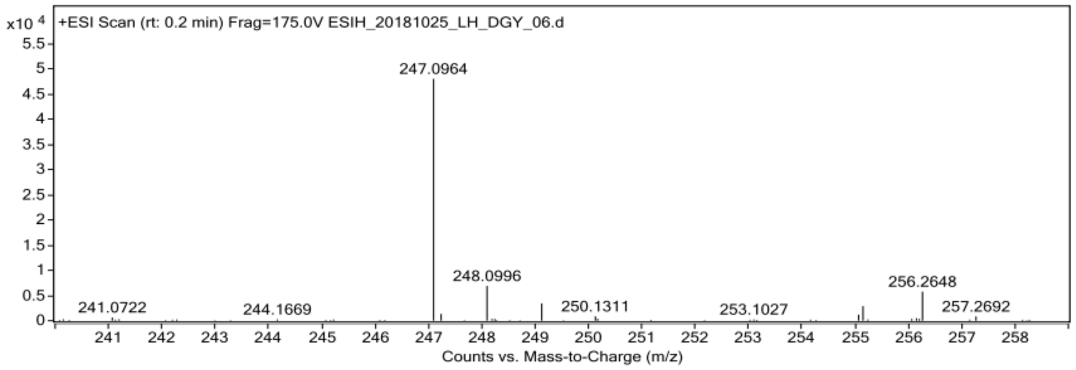

Formula Calculator Results

m/z	Calc m/z	Diff (mDa)	Diff (ppm)	Ion Formula	Ion
247.0964	247.0965	0.05	0.21	C14 H15 O4	(M+H)+

3ak

**Data Filename**	ESIH_20180809_LH_DGY_17.d	**Sample Name**	B6-GG-10
**Sample Type**	Sample	**Position**	P1-D3
**Instrument Name**	Agilent G6520 Q-TOF	**Acq Method**	20160322_MS_ESIH_POS_1min.m
**Acquired Time**	8/9/2018 17:41:58	**IRM Calibration Status**	Success
**DA Method**	small molecular data analysis method.m	**Comment**	ESIH by ZZY

User Spectra

Fragmentor Voltage	Collision Energy	Ionization Mode
110	0	ESI

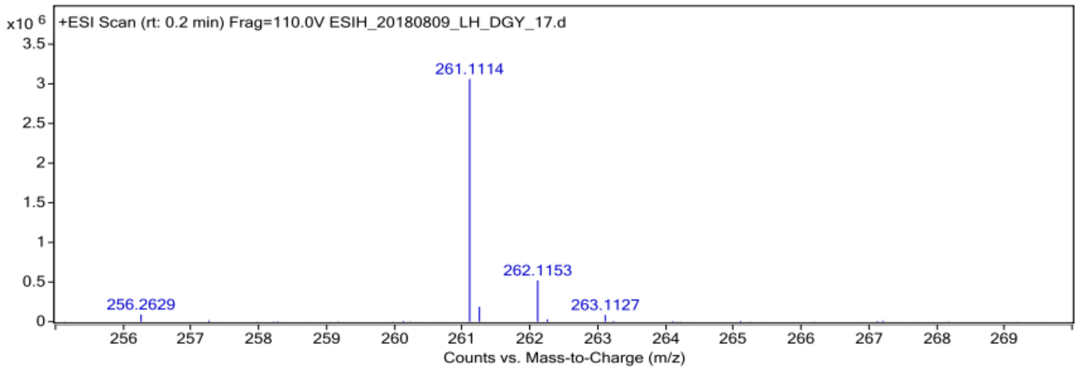

Formula Calculator Results

m/z	Calc m/z	Diff (mDa)	Diff (ppm)	Ion Formula	Ion
261.1114	261.1121	0.69	2.64	C15 H17 O4	(M+H)+

3al

**Data Filename**	ESIH_20180809_LH_DGY_12.d	**Sample Name**	B6-GG-18
**Sample Type**	Sample	**Position**	P1-C7
**Instrument Name**	Agilent G6520 Q-TOF	**Acq Method**	20160322_MS_ESIH_POS_1min.m
**Acquired Time**	8/9/2018 17:32:48	**IRM Calibration Status**	Success
**DA Method**	small molecular data analysis method.m	**Comment**	ESIH by ZZY

User Spectra

Fragmentor Voltage	Collision Energy	Ionization Mode
110	0	ESI

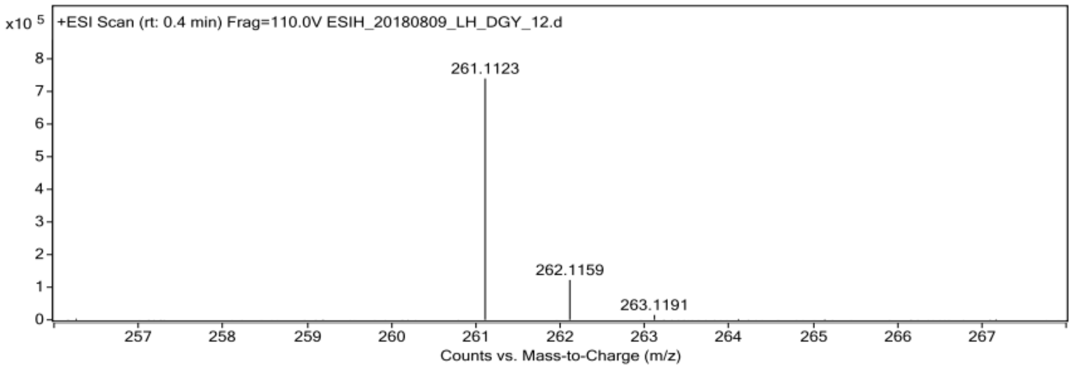

Formula Calculator Results

m/z	Calc m/z	Diff (mDa)	Diff (ppm)	Ion Formula	Ion
261.1123	261.1121	-0.14	-0.55	C15 H17 O4	(M+H)+

3am

**Data Filename**	ESIH_20181024_LH_DGY_06.d	**Sample Name**	B6-1024-CW2
**Sample Type**	Sample	**Position**	P1-B4
**Instrument Name**	Agilent G6520 Q-TOF	**Acq Method**	20160322_MS_ESIH_POS_1min.m
**Acquired Time**	10/24/2018 19:37:30	**IRM Calibration Status**	Success
**DA Method**	small molecular data analysis method.m	**Comment**	ESIH by ZZY

User Spectra

Fragmentor Voltage	Collision Energy	Ionization Mode
175	0	ESI

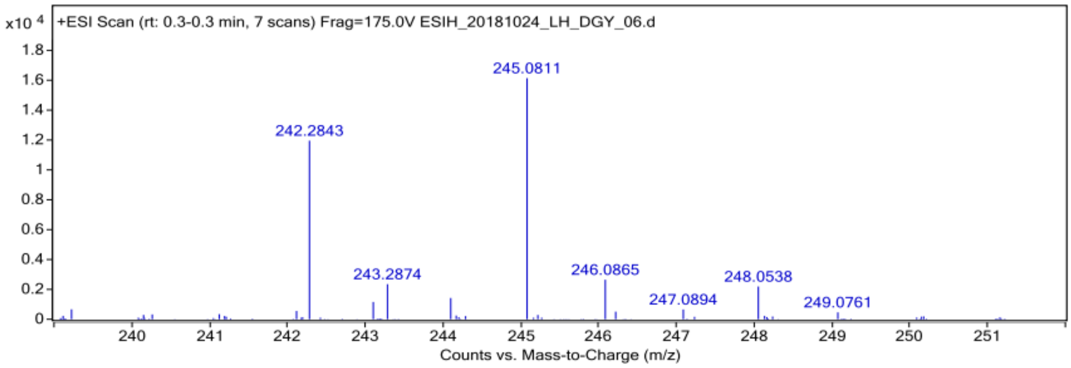

Formula Calculator Results

m/z	Calc m/z	Diff (mDa)	Diff (ppm)	Ion Formula	Ion
245.0811	245.0808	-0.28	-1.15	C14 H13 O4	(M+H)+

3an

**Data Filename**	ESIH_20181011_LH_DGY_05.d	**Sample Name**	B6-CW-G31
**Sample Type**	Sample	**Position**	P1-A4
**Instrument Name**	Agilent G6520 Q-TOF	**Acq Method**	20160322_MS_ESIH_POS_1min.m
**Acquired Time**	10/11/2018 17:42:21	**IRM Calibration Status**	Success
**DA Method**	small molecular data analysis method.m	**Comment**	ESIH by ZZY

User Spectra

**Fragmentor Voltage**	**Collision Energy**	**Ionization Mode**
175	0	ESI

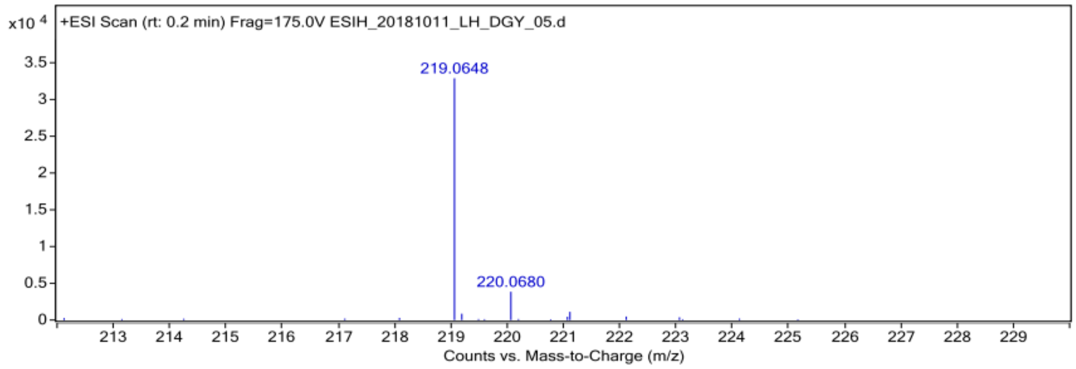

Formula Calculator Results

m/z	Calc m/z	Diff (mDa)	Diff (ppm)	Ion Formula	Ion
219.0648	219.0652	0.36	1.62	C12 H11 O4	(M+H)+

3ao

**Data Filename**	ESIH_20181012_LH_DGY_05.d	**Sample Name**	B6-Cw-32
**Sample Type**	Sample	**Position**	P1-B1
**Instrument Name**	Agilent G6520 Q-TOF	**Acq Method**	20160322_MS_ESIH_POS_1min.m
**Acquired Time**	10/12/2018 14:18:25	**IRM Calibration Status**	Success
**DA Method**	small molecular data analysis method.m	**Comment**	ESIH by ZZY

User Spectra

**Fragmentor Voltage**	**Collision Energy**	**Ionization Mode**
175	0	ESI

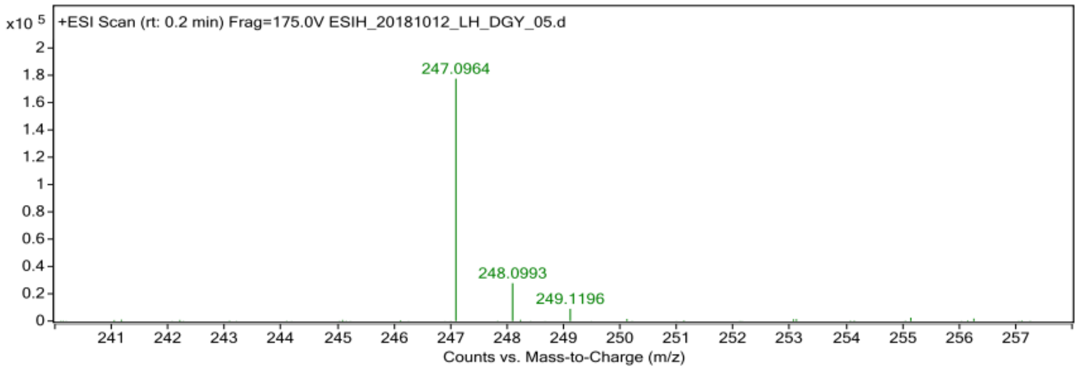

Formula Calculator Results

m/z	Calc m/z	Diff (mDa)	Diff (ppm)	Ion Formula	Ion
247.0964	247.0965	0.08	0.31	C14 H15 O4	(M+H)+

4

**Data Filename**	ESIH_20190214_LH_DGY_01.d	**Sample Name**	B6-TSNH
**Sample Type**	Sample	**Position**	P1-B1
**Instrument Name**	Agilent G6520 Q-TOF	**Acq Method**	20160322_MS_ESIH_POS_1min.m
**Acquired Time**	2/14/2019 14:26:17	**IRM Calibration Status**	Success
**DA Method**	small molecular data analysis method.m	**Comment**	ESIH by ZZY

User Spectra

Fragmentor Voltage	Collision Energy	Ionization Mode
135	0	ESI

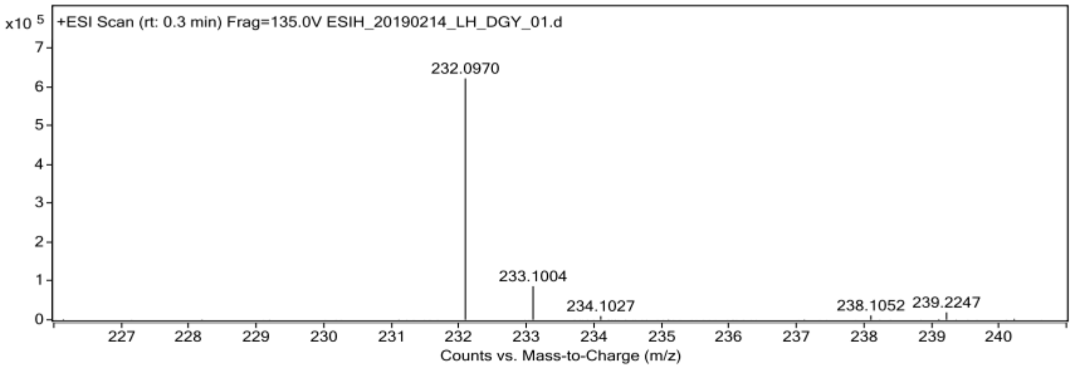

Formula Calculator Results

m/z	Calc m/z	Diff (mDa)	Diff (ppm)	Ion Formula	Ion
232.097	232.0968	-0.14	-0.62	C13 H14 N O3	(M+H)+

5

**Data Filename**	ESIH_20190215_LH_DGY_07.d	**Sample Name**	B6-TSTZ-2b
**Sample Type**	Sample	**Position**	P1-B7
**Instrument Name**	Agilent G6520 Q-TOF	**Acq Method**	20160322_MS_ESIH_POS_1min.m
**Acquired Time**	2/15/2019 14:57:11	**IRM Calibration Status**	Success
**DA Method**	small molecular data analysis method.m	**Comment**	ESIH by ZZY

User Spectra

Fragmentor Voltage	Collision Energy	Ionization Mode
135	0	ESI

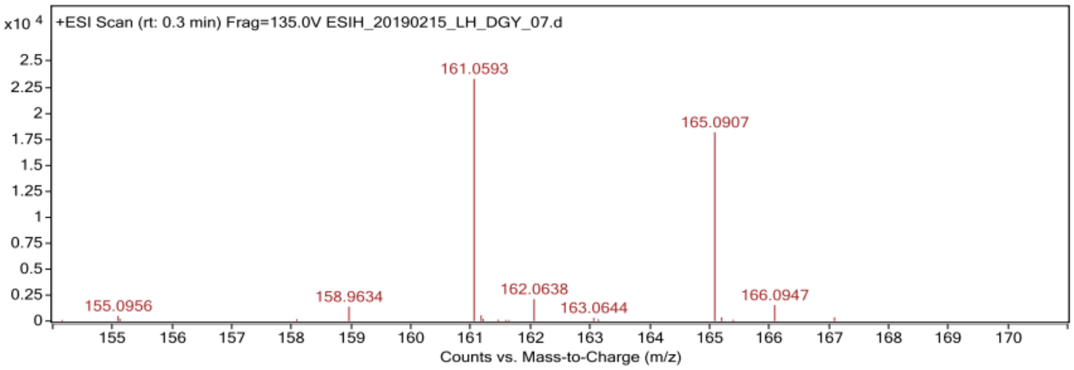

Formula Calculator Results

m/z	Calc m/z	Diff (mDa)	Diff (ppm)	Ion Formula	Ion
161.0593	161.0597	0.39	2.41	C10 H9 O2	(M+H)+
